# USP28 potentiates endothelial necroptosis via deubiquitinating NMMHC IIA in septic male mice

**DOI:** 10.1038/s41467-026-78666-8

**Published:** 2026-10-15

**Authors:** Jiahui Tang, Dingji Hu, Mengchen Xu, Haiyu Chen, Shuaishuai Gong, Fang Li, Boyang Yu, Yuanyuan Zhang, Songqiao Liu, Junping Kou

**Affiliations:** 1 Jiangsu Key Laboratory of TCM Evaluation and Translational Research, Department of Pharmacology of Chinese Materia Medica, School of Traditional Chinese Pharmacy, China Pharmaceutical University, Nanjing, Jiangsu China; 2 Jiangsu Provincial Key Laboratory of Critical Care Medicine, Department of Critical Care Medicine, Trauma Center, Zhongda Hospital, School of Medicine, Southeast University, Nanjing, Jiangsu China; 3 The First People’s Hospital of Lianyungang, The Affiliated Lianyungang Hospital of Xuzhou Medical University, Lianyungang, Jiangsu China

**Keywords:** Bacterial infection, Mechanisms of disease, Ubiquitylation

## Abstract

Endothelial dysfunction contributes to multi-organ injury in sepsis, but the underlying mechanisms remain incompletely understood. Here we show that endothelial ubiquitin-specific protease 28 (USP28) is increased in patients with sepsis and in male mice in a murine polymicrobial sepsis model. Mechanistically, USP28 stabilizes non-muscle myosin heavy chain IIA (NMMHC IIA) by removing K48-linked ubiquitination at K1413. Moreover, NMMHC IIA promotes interferon-α/β receptor 1 (IFNAR1) trafficking to the plasma membrane, enhancing IFNAR1/signal transducer and activator of transcription 1 (STAT1) signaling and endothelial necroptosis. In turn, STAT1 transcriptionally increases USP28 expression, forming a positive feedback loop. Endothelial USP28 deletion or pharmacological inhibition attenuates sepsis-induced multi-organ injury, whereas USP28 overexpression exacerbates it. Conversely, endothelial NMMHC IIA or IFNAR1 overexpression largely abrogates the protection conferred by USP28 deficiency in septic male mice. These findings identify a USP28–NMMHC IIA axis that promotes endothelial necroptosis and multi-organ injury in sepsis through IFNAR1/STAT1 signaling.

## Abstract (web summary)

Endothelial dysfunction contributes to multiorgan injury in sepsis, but the underlying mechanisms remain incompletely understood. Here, the authors show that USP28 deubiquitinates NMMHC IIA to enhance IFNAR1/STAT1 signaling, thereby driving endothelial necroptosis in sepsis in a male mouse model of sepsis.

## Introduction

Sepsis is a life-threatening syndrome of acute organ dysfunction caused by a dysregulated host response to infection, serving as the final common pathway in many fatal infectious diseases^1,2^. Globally, sepsis affects approximately 48.9 million individuals annually and causes 11 million deaths—approximately 20% of all global mortality^3^. A hallmark of sepsis is multi-organ dysfunction, predominantly characterized by acute lung injury, hepatic and renal failure, myocardial depression, and central nervous system dysfunction^4–6^. Mortality increases sharply when two or more organs are affected, with in-hospital death rates exceeding 50% in such cases^7,8^. Despite its severity, the exact mechanisms underlying sepsis-induced multi-organ dysfunction remain to be fully elucidated, and effective therapeutic interventions are currently lacking^9,10^.

Vascular endothelial cells (ECs), which line all blood vessels, play non-traditional immune roles by coordinating immune responses and maintaining vascular integrity^11,12^. Mounting evidence identifies endothelial dysfunction as a key driver of sepsis-induced multi-organ injury^13^. Under physiological conditions, ECs regulate vascular permeability, maintain anticoagulant properties, and control inflammation^14,15^. However, in sepsis, ECs are activated by pathogen-associated molecular patterns (PAMPs) and host-derived damage-associated molecular patterns (DAMPs), leading to the release of inflammatory mediators, upregulation of adhesion molecules, and even cell death. This promotes leukocyte adhesion and transmigration, amplifies inflammation^16,17^, and critically compromises the integrity of the endothelial barrier^18,19^. Barrier breakdown increases vascular permeability, causes protein-rich fluid leakage, and impairs tissue perfusion—culminating in organ edema, ischemia, and failure^20,21^. Therefore, it is important to explore the potential mechanisms of how ECs aid in the development of sepsis.

The ubiquitin-specific protease (USPs) family is the largest and most diverse deubiquitinase (DUBs) family. Numerous studies have revealed the important mechanisms by which USPs regulate biological processes by cleaving ubiquitin or ubiquitin-like peptides from substrate proteins, as exemplified in tumorigenesis, inflammation, and immune responses^22^. USP28 is a zinc-finger domain-containing deubiquitinase that primarily modulates protein stability and function by removing ubiquitin chains from target substrates^23^. USP28 has been implicated in cellular homeostasis, DNA damage repair, cell cycle regulation, and inflammatory modulation^24–26^. However, the detailed functional role of USP28 in sepsis and its underlying mechanisms remains unclear.

In this study, we used nanoproteomics to profile circulating endothelial cells (CECs) from patients with sepsis, which shows an obvious correlation between endothelial USP28 and sepsis-induced multi-organ injury. Therefore, we hypothesized that USP28 may act as a regulator of sepsis. We tested our hypothesis in vivo in USP28 conditional knockout/knockin mice, in primary mouse lung endothelial cells (MLECs), and in vitro in USP28-overexpressed and USP28-silenced human umbilical vein endothelial cells (HUVECs). We also identified the downstream target of USP28. These results support a role for USP28 in endothelial dysfunction and suggest USP28 as a potential therapeutic target in sepsis.

## Results

### Endothelial USP28 is upregulated in sepsis and positively correlates with multi-organ injury

**Fig. 1:**
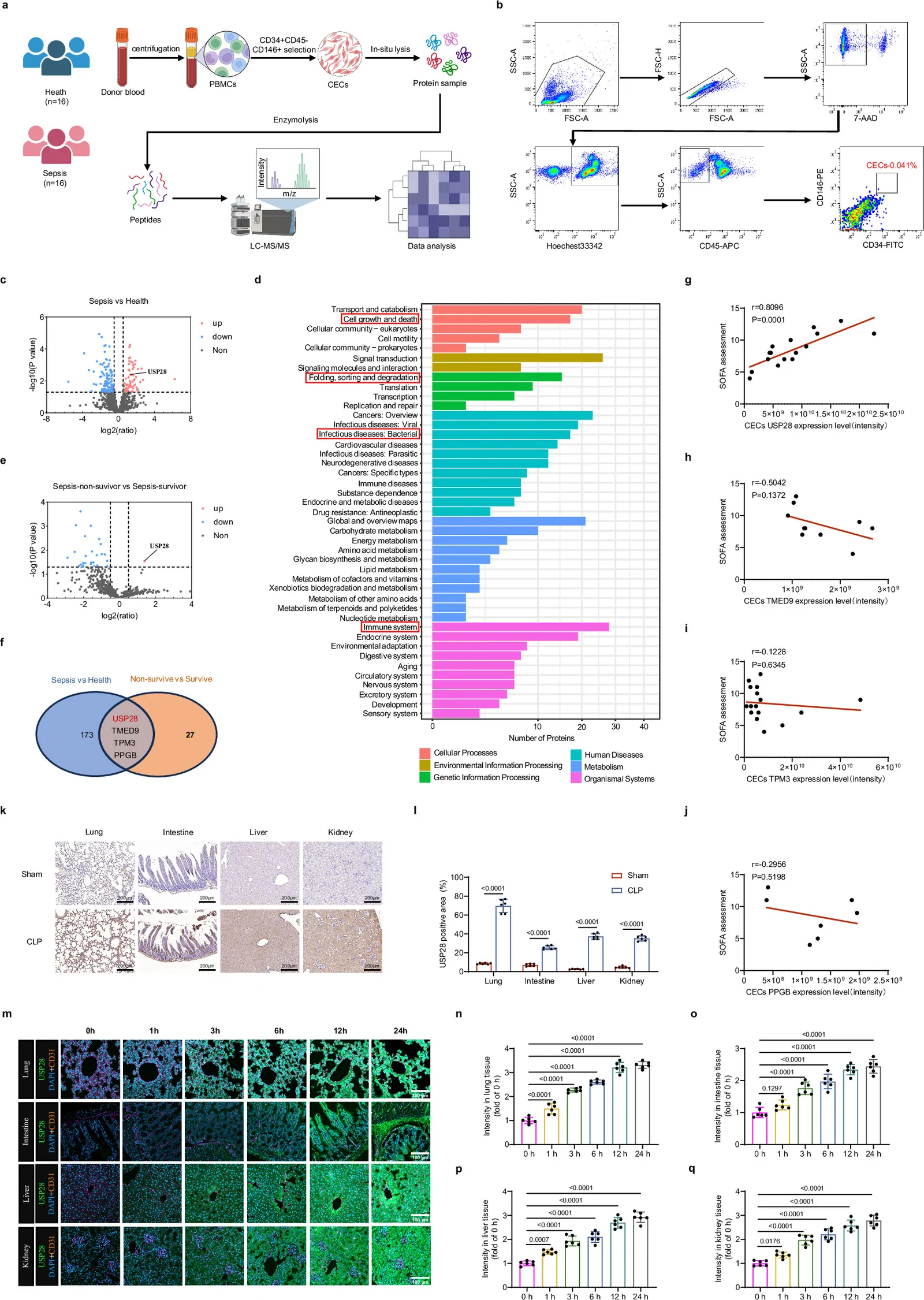
Endothelial USP28 is upregulated in sepsis and positively correlated with multi-organ injury. **a** A schematic diagram showing circulating endothelial cells (CECs) isolation and nanoproteomics sequencing in sepsis patients (*n* = 16 patients) and healthy donors (*n* = 16 healthy donors). Created in BioRender. Tang, J. (2026) https://BioRender.com/790ef18. **b** Fluorescence-activated cell sorting (FACS) showing the sorting gating of CECs. **c**,** d** Volcano plot (**c**) and Kyoto Encyclopedia of Genes and Genomes (KEGG) pathway analysis (**d**) of differently expressed genes between the sepsis group and healthy group. **e** A volcano plot of differently expressed genes between the sepsis-non-survivor group and the sepsis-survivor group. **f** A Venn diagram showing the overlapping differentially expressed genes (DEGs) from (**c**) and (**e**). **g–j** Correlation analysis between the USP28 (**g**), TMED9 (**h**), TPM3 (**i**) and PPGB (**j**) levels of CECs and the sequential organ failure assessment (SOFA) score of sepsis patients (*n* = 16 patients). **k**,** l** Representative immunohistochemical staining images and quantification of USP28 in lung, intestine, liver, and kidney tissues from mice at 24 h after cecal ligation and puncture (CLP) or sham surgery (*n* = 6 independent mice). Scale bar = 200 μm. **m–q** Representative immunofluorescent staining images and quantification of USP28 (green) in endothelium of the lung, intestine, liver, and kidney tissues from mice at 0, 1, 3, 6, 12, and 24 h after CLP (*n* = 6 independent mice). Endothelial cells were stained with CD31 (magenta), and nuclei were stained with DAPI (blue). Scale bar = 100 μm. All data were represented as the mean ± s.d. Differential protein expression in (**c**, **e**) was assessed using a two-sided Welch’s *t*-test with Benjamini–Hochberg adjustment for multiple comparisons. Statistical analysis was performed using two-tailed Student’s *t*-test (**l**), one-way ANOVA with Tukey post-hoc test (**n–q**), and correlation analysis using two-sided Spearman correlation coefficient (**g–j**). Exact *P* values are indicated in the figure. Source data are provided as a Source Data file.

CECs are endothelial cells detached from the vascular intima into the bloodstream, serve as representative biomarkers for minimally invasive detection of vascular damage, and show significantly increased numbers in patients with sepsis^27^. To trace the changes of ECs after sepsis challenge, we used nanoproteomics to profile CECs from healthy donors and patients with sepsis (Fig. 1a, b and Supplementary Table 1). A direct comparison of total protein expression between CECs from healthy donors and patients with sepsis revealed 173 differentially expressed genes (DEGs), of which 65 were upregulated and 108 downregulated (Fig. 1c). Kyoto encyclopedia of genes and genomes (KEGG) analysis of DEGs showed multiple dysregulated processes. For example, genes associated with cell growth and death, protein folding and degradation, infectious diseases and the immune system (Fig. 1d). To identify the key regulator, we further performed a comparative analysis between survivors and non-survivors of patients, which revealed one upregulated and 26 downregulated DEGs (Fig. 1e). Intersecting the DEGs from these comparation identified four recurring genes, including *USP28*, transmembrane embryonic associated protein 9 (*TMED9*), tropomyosin 3 (*TPM3*), and protective protein for β-galactosidase (*PPGB*) (Fig. 1f). We observed that USP28 was the only protein that showed a significant positive correlation with sequential organ failure assessment (SOFA) score in patients with sepsis, whereas *TMED9*, *TPM3*, and *PPGB* did not exhibit such an association (Fig. 1g–j). We found that USP28 was upregulated in the lung, intestine, liver, and kidney tissues of mice with cecal ligation and puncture (CLP)-induced polymicrobial sepsis (Fig. 1k, l). Moreover, immunofluorescence staining across multiple organs showed that USP28 expression was markedly increased in endothelial cells of the lung, intestine, liver, and kidney following CLP, with a time-dependent increase observed within 24 h (Fig. 1m–q). Further cell-type–specific analysis in lung tissues revealed that USP28 expression was selectively increased in endothelial cells (Supplementary Fig. 1a, b). Together, our data show that endothelial USP28 expression is increased during sepsis and is associated with sepsis-induced multi-organ failure and death.

### Endothelial USP28 enhances the development of polymicrobial sepsis

**Fig. 2:**
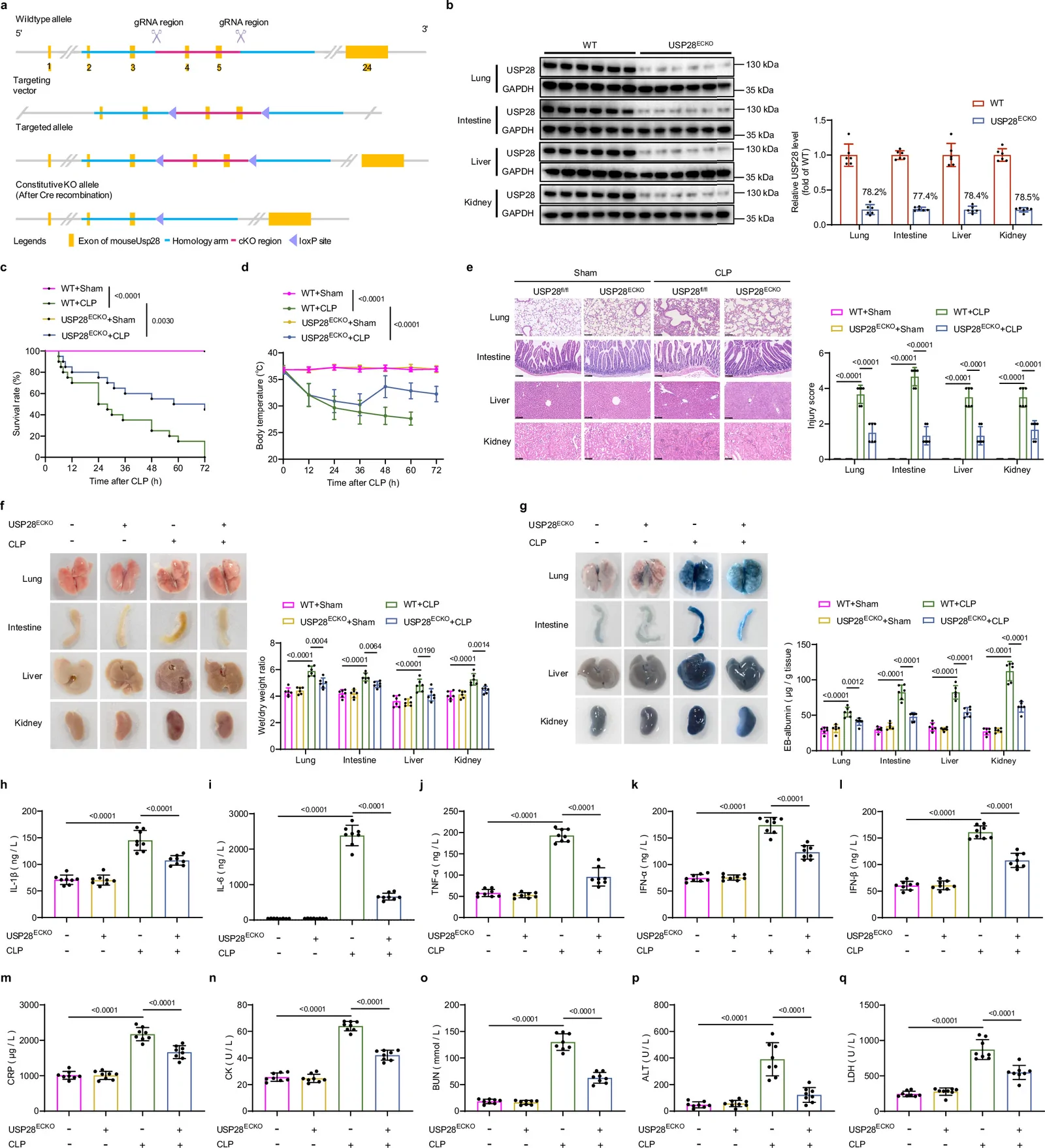
Endothelial-specific USP28 knockout ameliorates multi-organ injury in polymicrobial sepsis. Wild-type (WT) and *USP28*^ECKO^ mice were treated with cecal ligation and puncture (CLP) or sham surgery. **a** A schematic illustrating the generation of *USP28*^ECKO^ mice. **b** Immunoblot analysis and quantification of USP28 in primary endothelial cells (ECs) isolated from lung, intestine, liver, and kidney of WT or *USP28*^ECKO^ mice (*n* = 6 independent mice). **c** Survival rate (*n* = 20 independent mice). **d** Body temperature (*n* = 6 independent mice). **e** Hematoxylin and eosin (H&E) staining and injury score of lung, intestine, liver, and kidney tissues (*n* = 6 independent mice). Scale bar = 100 μm. **f** Representative gross morphology and wet/dry ratio of lung, intestine, liver, and kidney tissue (*n* = 6 independent mice). **g** Evaluation of vascular leakages using Evans blue tracer. Leakage degree was quantified by detecting the Evans blue dye contents in lung, intestine, liver, and kidney homogenate (*n* = 6 independent mice). **h–l** Quantitation of IL-1β (**h**), IL-6 (**i**), TNF-α (**j**), IFN-α (**k**), and IFN-β (**l**) in plasma (*n* = 8 independent mice). **m–q** Quantitation of CRP (**m**), CK (**n**), BUN (**o**), ALT (**p**), and LDH (**q**) in plasma (*n* = 8 independent mice). All data were represented as the mean ± s.d. Statistical analysis was performed using two-way ANOVA with Tukey post hoc test (**d–q**), and survival analysis using the two-sided log-rank (Mantel–Cox) test (**c**). Exact *P* values are indicated in the figure. Source data are provided as a Source Data file.

To assess the role of endothelial USP28 on the development of sepsis, we generated endothelial-specific USP28 knockout (*USP28*^ECKO^) mice (Fig. 2a, b). *USP28*^ECKO^ did not affect baseline physiological conditions in sham-treated mice (Fig. 2c–q). Compared with that of WT mice, *USP28*^ECKO^ improved the survival and hypothermia of CLP-treated mice (Fig. 2c, d). Moreover, *USP28*^ECKO^ significantly attenuated pathological injury, tissue edema, and vascular leakage of the lung, intestine, liver, and kidney in CLP-treated mice (Fig. 2e–g). The elevated levels of plasma pro-inflammatory cytokines, including interleukin-1β (IL-1β), IL-6, tumor necrosis factor (TNF)-α, and IFN-α/β in CLP-treated mice were reduced by *USP28*^ECKO^ (Fig. 2h–l). Consistent with this, the elevated levels of DAMPs associated with multi-organ injury in CLP-treated mice, including C-reactive protein (CRP), creatine kinase (CK), blood urea nitrogen (BUN), alanine aminotransferase (ALT), and lactate dehydrogenase (LDH), were reduced by *USP28*^ECKO^ (Fig. 2m–q).

To investigate the potential of USP28 inhibition as a therapeutic approach for treating sepsis, we intragastrically administered the USP28 inhibitor (AZ-1, 20 mg/kg) to mice immediately prior to CLP surgery. Similar to what we observed in *USP28*^ECKO^ mice, starting AZ-1 therapy early helped reduce mortality and increase the reduced body temperature of CLP-treated mice (Supplementary Fig. 2a, b). In addition, early AZ-1 treatment reduced the elevated pro-inflammatory cytokines and the progression of multi-organ injury in CLP-induced septic mice (Supplementary Fig. 2c–o). Consistently, delayed administration of AZ-1 at 1 h after CLP surgery produced comparable protective effects, including improved survival, attenuated inflammatory responses, and reduced multi-organ injury (Supplementary Fig. 3c–p), supporting its efficacy in a post-insult setting. To further determine whether these effects are dependent on endothelial USP28, endothelial-specific USP28-deficient mice were generated using an adeno-associated virus type 9 (AAV9) carrying USP28 shRNA under the endothelial-specific promoter Tie1 (Supplementary Fig. 3a, b). *USP28*^AAV9-Tie1-sh^ did not affect baseline physiological conditions in sham-treated mice (Supplementary Fig. 3c–p). Notably, AZ-1 treatment failed to confer additional protection in *USP28*^AAV9-Tie1-sh^ mice, as reflected by the absence of further improvement in survival, inflammatory responses, and multi-organ injury (Supplementary Fig. 3c–p), suggesting that the protective effects of AZ-1 are largely dependent on endothelial USP28.

To provide further evidence for the involvement of endothelial USP28 in sepsis, endothelial-specific USP28 overexpression mice were generated under the endothelial-specific promoter Tie1 by the AAV9 system (Supplementary Fig. 4a, b). *USP28*^AAV9-Tie1^ did not affect baseline physiological conditions in sham-treated mice (Supplementary Fig. 4c–o). Consistent with previous results, *USP28*^AAV9-Tie1^ exacerbated the mortality and hypothermia of CLP-treated mice (Supplementary Fig. 4c, d). Moreover, *USP28*^AAV9-Tie1^ led to more severe pathological injury, tissue edema, and vascular leakage of multiple organs (lung, intestine, liver, and kidney), as well as a higher level of pro-inflammatory cytokines and DAMPs in CLP-treated mice (Supplementary Fig. 4e–o). Overall, these findings demonstrate that endothelial USP28 promotes the development of sepsis.

### USP28 mediates EC necroptosis via IFNAR1/STAT1 signaling

**Fig. 3:**
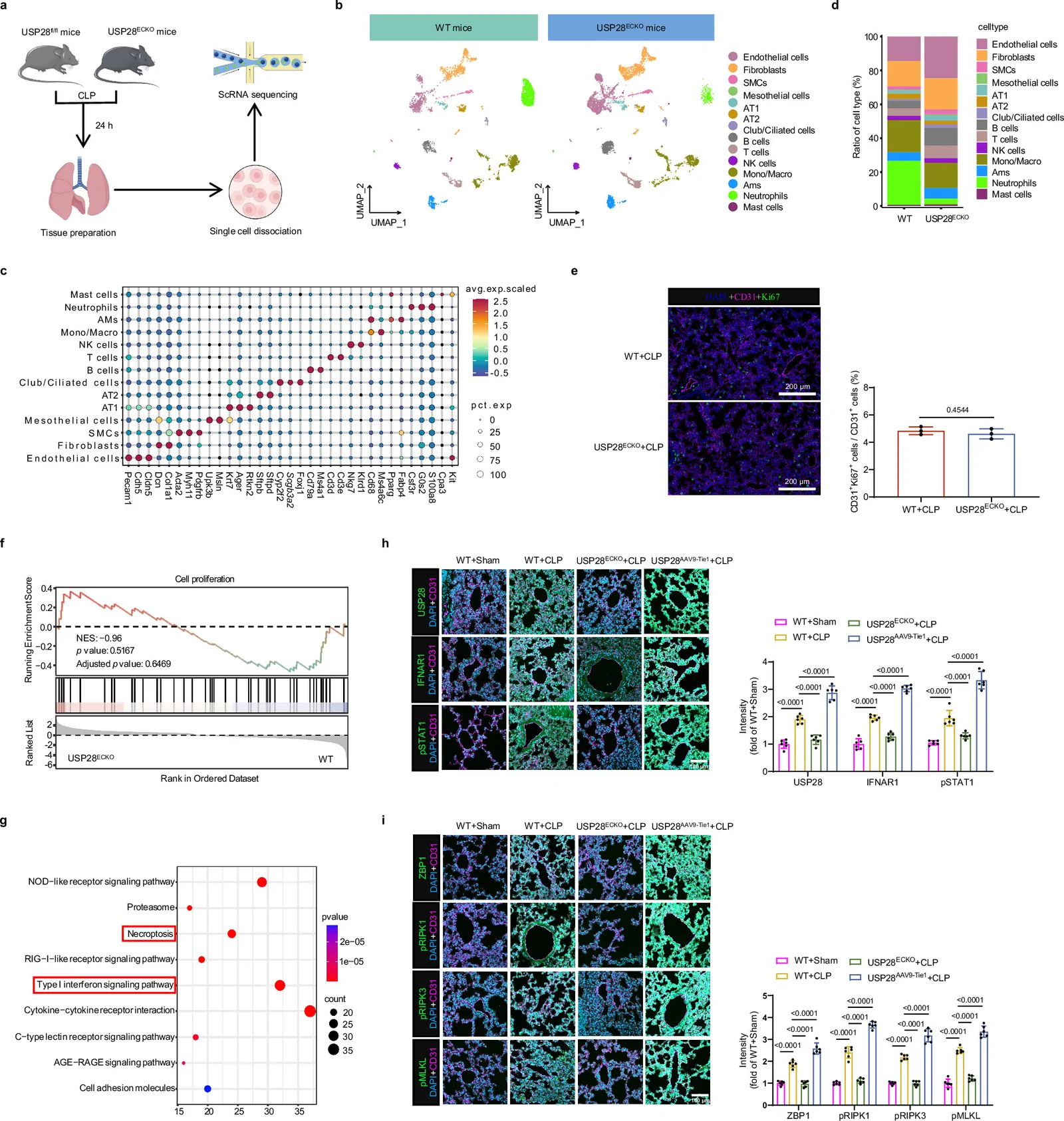
USP28 mediates endothelial necroptosis via IFNAR1/STAT1 signaling in polymicrobial sepsis. **a** A schematic diagram showing the experimental design for single-cell RNA sequencing (scRNA-seq) of lung tissue from WT and *USP28*^ECKO^ mice at 24 h after cecal ligation and puncture (CLP) surgery. Created in BioRender. Tang, J. (2026) https://BioRender.com/mj5m2ey. **b** Uniform manifold approximation and projection (UMAP) plot of 14 cell types identified from the lung tissue of wild-type (WT) and *USP28*^ECKO^ mice. **c** Dot plot showing the representative marker genes across the 14 clusters. **d** Proportions of the clusters from different groups. **e** Immunofluorescence staining of Ki67 (green) and CD31 (magenta) in lung tissues from WT and *USP28*^ECKO^ mice following CLP. Nuclei were stained with DAPI (blue). Scale bar = 200 μm. Quantification of the proportion of Ki67-positive CD31^+^ cells is shown on the right (*n* = 3 independent mice). **f** Gene set enrichment analysis (GSEA) analysis of cell proliferation-related pathways in endothelial cells (ECs) cluster. **g** Kyoto Encyclopedia of Genes and Genomes (KEGG) enrichment analysis for differentially expressed genes (DEGs) in endothelial cells comparing CLP-treated WT and *USP28*^ECKO^ mice. **h**,** i** WT, *USP28*^ECKO^ and *USP28*^AAV9-Tie1^ mice were detected at 24 h after CLP surgery. Representative immunofluorescent staining images and quantification of USP28, IFNAR1, pSTAT1, ZBP1, pRIPK1, pRIPK3, and pMLKL in the endothelium of the lung tissues (*n* = 6 independent mice). The target proteins were stained in green. Endothelial cells were stained with CD31 (magenta), and nuclei were stained with DAPI (blue). Scale bar = 100 μm. All data were represented as the mean ± s.d. Statistical analysis was performed using two-way ANOVA with Tukey post hoc test (**h–i**). Exact *P* values are indicated in the figure. Source data are provided as a Source Data file.

To further explore the role of USP28 in sepsis, we performed single-cell RNA sequencing (scRNA-seq) on lung tissues from WT and *USP28*^ECKO^ mice after 24 h post CLP surgery (Fig. 3a). After rigorous quality control, 10,951 cells were analyzed for transcriptional profiling, including 4620 cells from the CLP-treated WT mice and 6097 cells from the CLP-treated *USP28*^ECKO^ mice. Total cells were grouped and identified as 14 different cell types by uniform manifold approximation and projection (UMAP) (Fig. 3b, c). We found that *USP28*^ECKO^ significantly increased the proportion of endothelial cells in the lung tissues of septic mice (Fig. 3d).

To explore the mechanism underlying this increase, we next assessed EC proliferation. However, no significant difference in the number of proliferating ECs was observed between WT and *USP28*^ECKO^ mice following CLP (Fig. 3e). Consistently, Gene set enrichment analysis (GSEA) of ECs clusters showed no enrichment of proliferation-related signatures (Fig. 3f), suggesting that the increased ECs proportion is unlikely driven by enhanced proliferation. KEGG analysis revealed that USP28 markedly regulated necroptosis and IFN-I signaling in ECs (Fig. 3g). To further dissect endothelial heterogeneity, pulmonary ECs were re-clustered into distinct subtypes, including arterial, venous, capillary, and lymphatic ECs based on canonical marker genes (Supplementary Fig. 5a, b). Notably, IFN-I signaling and necroptosis-related transcriptional signatures were consistently enriched across arterial, venous, and capillary EC subsets following USP28 deletion, whereas lymphatic ECs showed no significant changes (Supplementary Fig. 5c–j), indicating a subtype-specific response predominantly in vascular ECs.

Reportedly, IFN-I specifically induces necroptosis by activating the Z-DNA binding protein 1 (ZBP1)–mixed lineage kinase domain-like (MLKL) signaling^28–30^. Therefore, we hypothesize that USP28 may potentiate IFN-1 signaling-induced endothelial necroptosis in sepsis. Consistent with our hypothesis, the expression of IFNAR1 and pSTAT1, along with necroptotic markers including ZBP1, Phospho-receptor-interacting serine/threonine kinase 1 (pRIPK1), pRIPK3, and pMLKL, was significantly upregulated in pulmonary ECs of CLP-treated mice (Fig. 3h, i). However, *USP28*^ECKO^ reduced the elevated expression of IFN-I signaling-associated proteins and necroptosis-associated proteins in CLP-treated mice, whereas *USP28*^AAV9-Tie1^ exacerbated the expression (Fig. 3h, i). Consistently, USP28 deficiency reduced the elevated expression of IFN-1 signaling-associated proteins and necroptosis-associated proteins in IFN-β-treated MLECs, whereas USP28 overexpression enhanced these responses (Supplementary Fig. 6a–c). In contrast, IFN-β stimulation did not alter the levels of pyroptotic (GSDMD-N/GSDMD) or apoptotic (cleaved caspase-3/caspase-3) markers in MLECs, and these markers remained unchanged upon USP28 deficiency or overexpression (Supplementary Fig. 6d–g).

To further test the hypothesis, we performed in vitro experiments using IFN-β-stimulated HUVECs. Under IFN-β stimulation, the impaired cell viability and trans-endothelial electrical resistance (TEER) alongside enhanced Evans blue (EB) leakage in HUVECs were ameliorated by USP28 silencing, but exacerbated by USP28 overexpression (Supplementary Fig. 7a–c). Consistent with these results, USP28 silencing reduced the elevated expression of IFN-I signaling-associated proteins and necroptosis-associated proteins in IFN-β-treated HUVECs, whereas USP28 overexpression exacerbated these effects (Supplementary Fig. 7d–g). Similarly, IFN-β stimulation did not affect the levels of cleaved caspase-3/caspase-3 and GSDMD-N/GSDMD in HUVECs, and these proteins remained unchanged upon USP28 silencing or overexpression (Supplementary Fig. 7i–l). Moreover, silencing of MLKL or RIPK3 abolished IFN-β-induced cell death in HUVECs, as reflected by restored cell viability (Supplementary Fig. 7m–o). In summary, these results showed that endothelial USP28 initiates the expression of necroptosis-related genes via IFNAR1/STAT1 signaling.

### IFNAR1 overexpression largely abrogates the protective effects of USP28 deficiency in sepsis

**Fig. 4:**
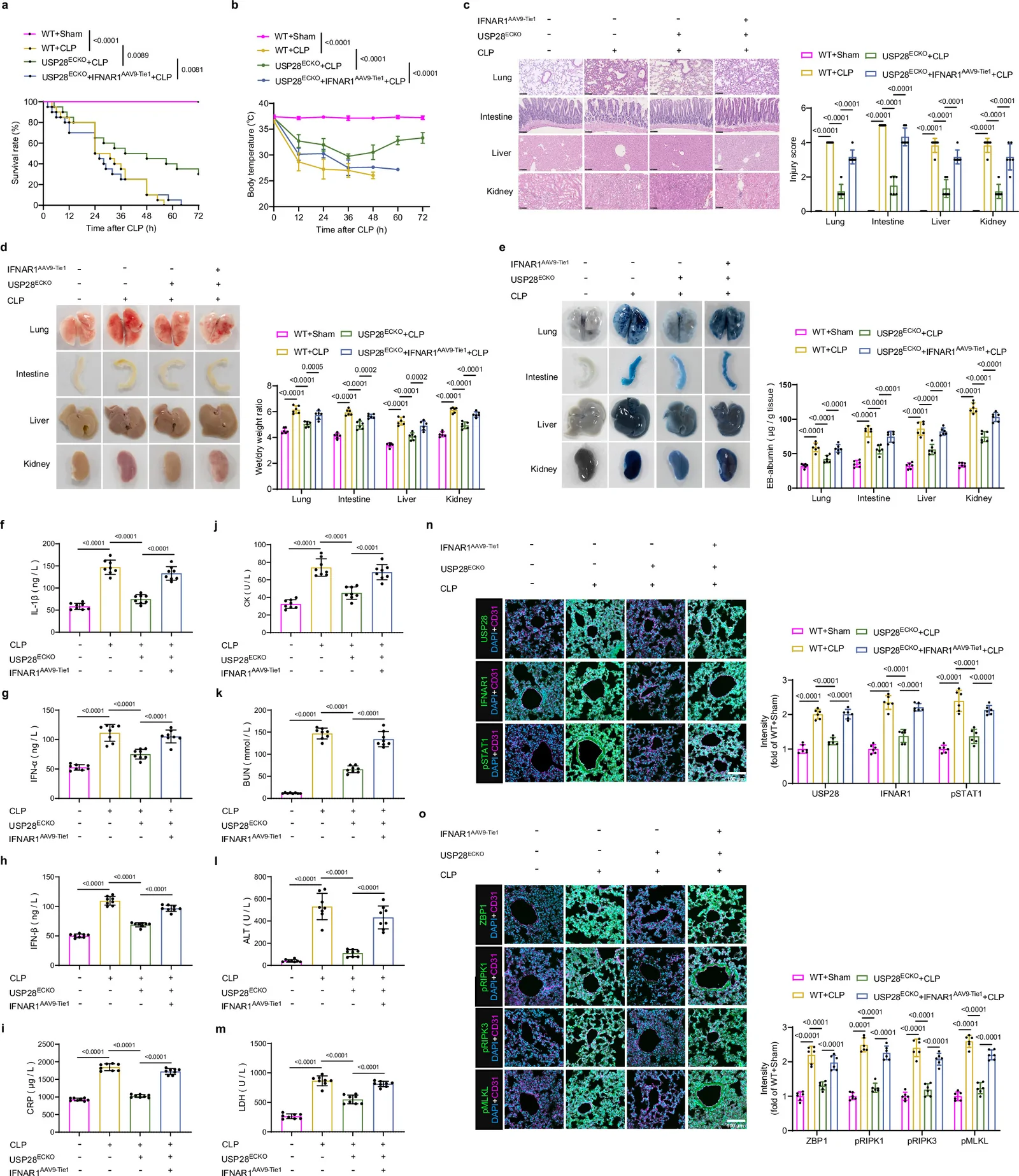
Endothelial-specific IFNAR1 overexpression largely abrogates the protective effect of USP28 deficiency in polymicrobial sepsis. Wild-type (WT) and *USP28*^ECKO^ mice were intravenously injected with rAAV9-tie1-vector, and *USP28*^ECKO^ + *IFNAR1*^AAV9-Tie1^ mice with rAAV9-tie1-IFNAR1. All groups were then treated with cecal ligation and puncture (CLP) or sham surgery. **a** Survival rate (*n* = 20 independent mice). **b** Body temperature (*n* = 6 independent mice). **c** Hematoxylin and eosin (H&E) staining and injury score of lung, intestine, liver, and kidney tissues (*n* = 6 independent mice). Scale bar = 100 μm. **d** Representative gross morphology and wet/dry ratio of lung, intestine, liver, and kidney tissue (*n* = 6 independent mice). **e** Evaluation of vascular leakages using Evans blue tracer. Leakage degree was quantified by detecting the Evans blue dye contents in lung, intestine, liver, and kidney homogenate (*n* = 6 independent mice). **f–h** Quantitation of IL-1β (**f**), IFN-α (**g**), and IFN-β (**h**) in plasma (*n* = 8 independent mice). **i–m** Quantitation of CRP (**i**), CK (**j**), BUN (**k**), ALT (**l**), and LDH (**m**) in plasma (*n* = 8 independent mice). **n**,** o** Representative immunofluorescent staining images and quantification of USP28, IFNAR1, pSTAT1, ZBP1, pRIPK1, pRIPK3, and pMLKL in endothelium of the lung tissues (*n* = 6 independent mice). The target proteins were stained in green. Endothelial cells were stained with CD31 (magenta), and nuclei were stained with DAPI (blue). Scale bar = 100 μm. All data were represented as the mean ± s.d. Statistical analysis was performed using two-way ANOVA with Tukey post hoc test (**b–o**), and survival analysis using the two-sided log-rank (Mantel–Cox) test (**a**). Exact *P* values are indicated in the figure. Source data are provided as a Source Data file.

To verify whether USP28 deficiency improves sepsis through regulating IFNAR1/STAT1 signaling, we constructed AAV9 vector carrying IFNAR1 under the endothelial-specific promoter Tie1 (Supplementary Fig. 8a, b), and administered to WT or *USP28*^ECKO^ mice via tail vein injection. *IFNAR1*^AAV9-Tie1^ did not affect baseline physiological conditions in sham-treated mice (Supplementary Fig. 8c–n). As anticipated, *USP28*^ECKO^ improved the survival and hypothermia of septic mice, while *IFNAR1*^AAV9-Tie1^ abrogated the protective effects of USP28 deficiency (Fig. 4a, b). Likewise, *USP28*^ECKO^ significantly attenuated pathological injury, tissue edema, and vascular leakage of multiple organs (lung, intestine, liver, and kidney), as well as higher levels of pro-inflammatory cytokines and DAMPs in septic mice, while these protective effects were abolished by *IFNAR1*^AAV9-Tie1^ (Fig. 4c–m). Moreover, *USP28*^ECKO^ inhibited the activation of IFN-I signaling and necroptosis in pulmonary ECs of septic mice but not in *IFNAR1*^AAV9-Tie1^ mice (Fig. 4n, o). Consistently, in IFN-β-stimulated MLECs, *USP28*^ECKO^ inhibited the activation of IFN-I signaling and necroptosis, whereas *IFNAR1*^AAV9-Tie1^ abrogated these effects (Supplementary Fig. 9a–c). Collectively, these results suggest that USP28 deficiency preserves endothelial function and attenuates sepsis via IFNAR1/STAT1 signaling.

### USP28 interacts with NMMHC IIA to modulate deubiquitinated modification and stability of NMMHC IIA

**Fig. 5:**
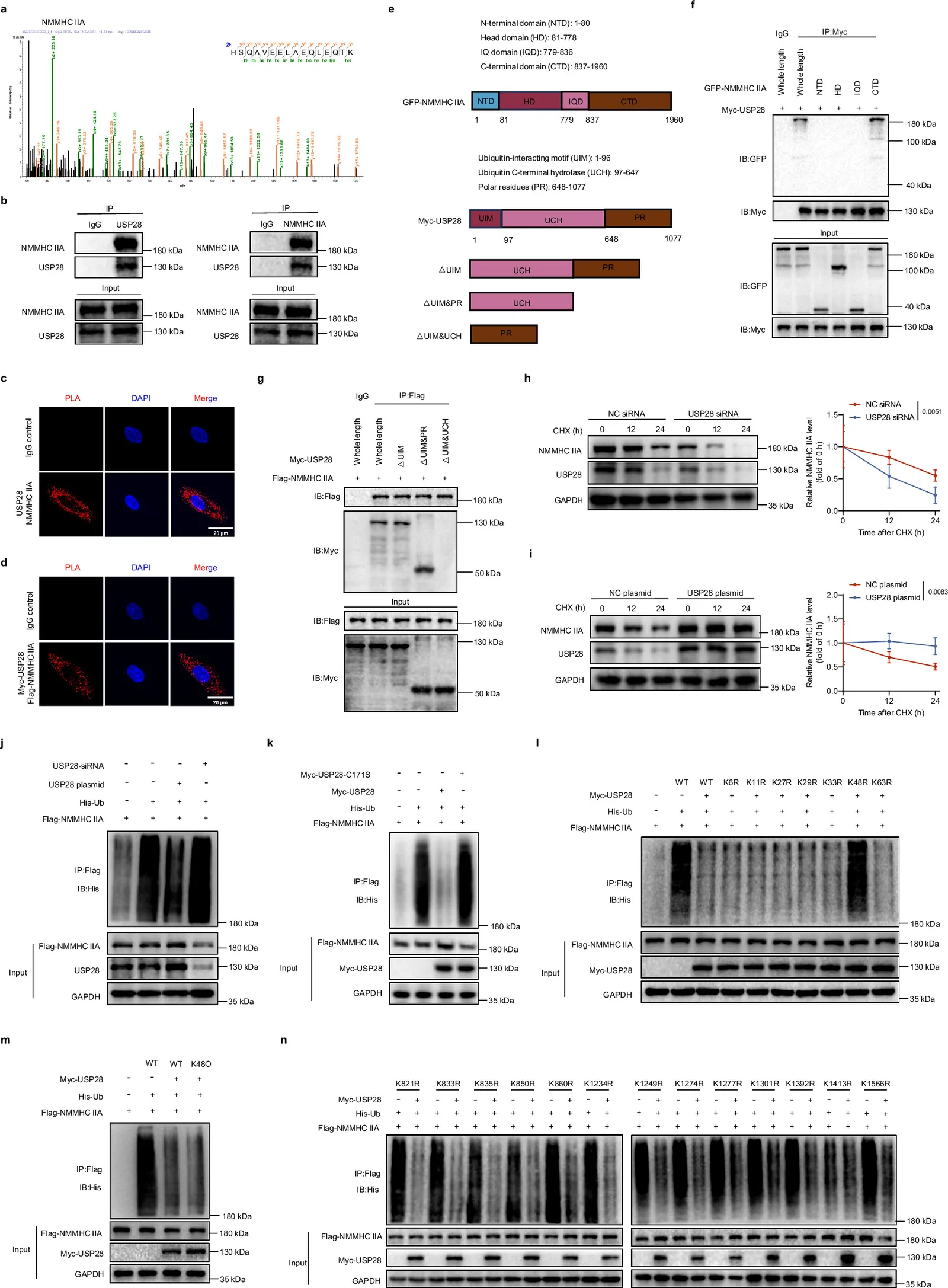
USP28 interacts with NMMHC IIA to modulate deubiquitinated modification and stability of NMMHC IIA. **a** An immunoprecipitation–mass spectrometry (IP-MS) analysis showed NMMHC IIA was identified to be a putative protein binding to USP28. **b** Co-immunoprecipitation (Co-IP) analysis between USP28 and NMMHC IIA in human umbilical vein endothelial cells (HUVECs) (*n* = 6 independent cultures). **c**,** d** Proximity ligation assay (PLA) (red) showing the interaction between USP28 and NMMHC IIA in HUVECs (**c**), and between Myc-USP28 and Flag-NMMHC IIA in human embryonic kidney 293 T (HEK-293T) cells (**d**) (*n* = 6 independent cultures). Nuclei were stained with DAPI (blue). Scale bar = 20 μm. **e** A graphical presentation for the N-terminal, head, IQ, C-terminal domain of NMMHC IIA and the ubiquitin-interacting motif, ubiquitin C-terminal hydrolase, polar residues domain of USP28. **f**,** g** HEK-293T cells were transfected with various combinations of plasmids. IP analysis was performed to examine the specific binding domains of USP28 and NMMHC IIA (*n* = 3 independent cultures). **h**,** i** HUVECs were transfected with control siRNA, *USP28* siRNA, control vector, or *USP28* plasmid, and then treated with cycloheximide (CHX, 10 μmol/L) for the indicated time periods. Representative immunoblot analysis of USP28 and NMMHC IIA protein levels for each group, and quantification of NMMHC IIA level (*n* = 6 independent cultures). **j** Ubiquitination levels of NMMHC IIA were assessed in HEK-293T cells co-transfected with Flag-NMMHC IIA, His-Ub, *USP28* siRNA and *USP28* plasmid (*n* = 6 independent cultures). **k** Ubiquitination levels of NMMHC IIA were assessed in HEK-293T cells co-transfected with Myc-USP28, Myc-USP28-C171S, Flag-NMMHC IIA and His-Ub (*n* = 6 independent cultures). **l**,** m** IP analysis was conducted in HEK-293T cells co-transfected with Myc-USP28, Flag-NMMHC IIA, His-Ub (WT) and His-tagged various Ub mutants to determine NMMHC IIA ubiquitination levels (*n* = 6 independent cultures). **n** An IP analysis was conducted in HEK-293T cells co-transfected with Myc-USP28, His-Ub and different Flag-NMMHC IIA mutants to determine NMMHC IIA ubiquitination levels (*n* = 6 independent cultures). All data were represented as the mean ± s.d. Statistical analysis was performed using a two-tailed Student’s *t*-test (**h, i**). Exact *P* values are indicated in the figure. Source data are provided as a Source Data file.

To further investigate the potential mechanism by which USP28 potentiates endothelial necroptosis in sepsis, we used immunoprecipitation-coupled mass spectrometry (IP/MS) to determine the proteins that interact with USP28. Non-muscle myosin heavy chain IIA (NMMHC IIA) was identified as the top-ranked protein that interacts with USP28 (Fig. 5a). Complementarily, we found that endogenous USP28 co-precipitated with NMMHC IIA in HUVECs (Fig. 5b). Consistently, proximity ligation assay (PLA) further confirmed the interaction between USP28 and NMMHC IIA (Fig. 5c, d). Mapping USP28–NMMHC IIA interaction motifs revealed that the ubiquitin C-terminal hydrolase domain (97–647) of USP28, as well as the C-terminal domain (837–1960) of NMMHC IIA, were required for their interaction (Fig. 5e–g).

We then explored the effect of USP28 expression on the protein stability of endogenous NMMHC IIA in the presence of the protein synthesis inhibitor cycloheximide (CHX). We observed that USP28 silencing markedly promoted NMMHC IIA degradation, whereas USP28 overexpression suppressed NMMHC IIA degradation (Fig. 5h, i). Next, we sought to determine the effect of USP28 on the ubiquitination of NMMHC IIA. Forced expression of USP28 instead of the deubiquitinase-dead mutant USP28-C171S decreased the ubiquitination of NMMHC IIA, whereas USP28 knockdown increased NMMHC IIA ubiquitination (Fig. 5j, k). A ubiquitination assay indicated that USP28 could efficiently remove the K48-linked ubiquitin chain from the NMMHC IIA protein (Fig. 5l, m). We further mapped the USP28-mediated deubiquitination sites of NMMHC IIA. By screening the online ubiquitination site prediction database for NMMHC IIA with a high-throughput publication count threshold of ≥3^31^, we identified 13 potential sites in the C-terminal domain of NMMHC IIA protein (Supplementary Table 2). We generated various mutants of NMMHC IIA for these 13 sites and found that K1413 was the key site on NMMHC IIA deubiquitinated by USP28 (Fig. 5n). Taken together, these data indicate that USP28 regulates the stability of NMMHC IIA by deubiquitinating it in ECs.

### NMMHC IIA is essential for USP28-mediated EC necroptosis

To further test the hypothesis that USP28 induces EC necroptosis via NMMHC IIA, we performed rescue experiments by restoring NMMHC IIA in USP28-silencing HUVECs. We found that forced overexpression of NMMHC IIA reversed the protective effects of USP28 silencing on cell viability and endothelial permeability in IFN-β-treated HUVECs (Supplementary Fig. 10a–c). Consistent with this, forced overexpression of NMMHC IIA restored the expression of IFNAR1, pSTAT1/STAT1, ZBP1, pRIPK1/RIPK1, pRIPK3/RIPK3 and pMLKL/MLKL in the absence of USP28 (Supplementary Fig. 10d–h).

**Fig. 6:**
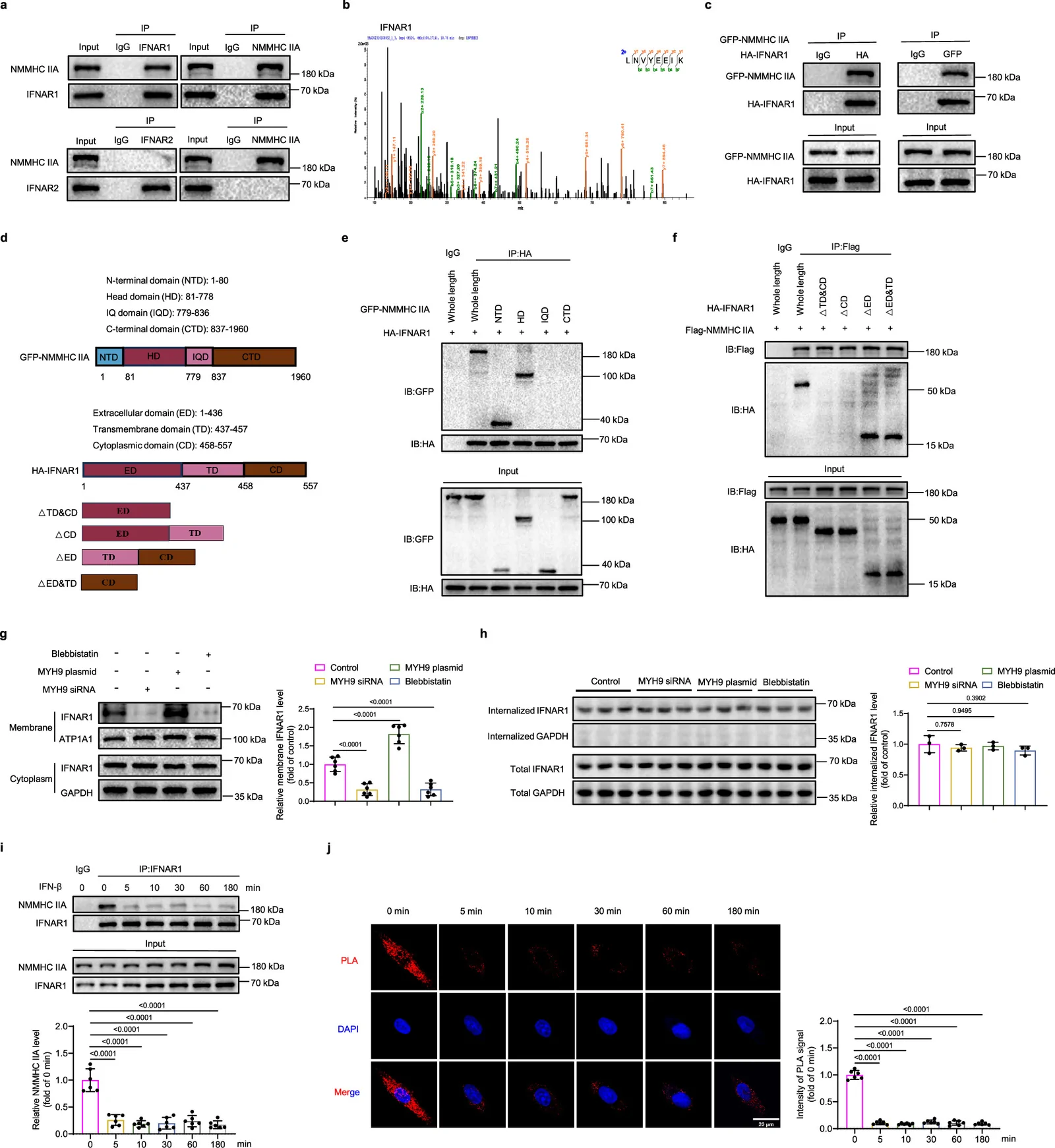
NMMHC IIA interacts with and facilitates IFNAR1 trafficking to the membrane. **a** Co-IP analysis between NMMHC IIA and IFNAR1/2 in human umbilical vein endothelial cells (HUVECs) (*n* = 6 independent cultures). **b** Immunoprecipitation–mass spectrometry (IP-MS) analysis showing IFNAR1 was identified to be a putative protein binding to NMMHC IIA. **c** Co-immunoprecipitation (Co-IP) analysis was performed to examine the interaction between GFP-NMMHC IIA and HA-IFNAR1 in human embryonic kidney 293 T (HEK-293T) cells (*n* = 6 independent cultures). **d** Graphical presentation for the extracellular, transmembrane, cytoplasmic domain of IFNAR1. **e**,** f** HEK-293T cells were transfected with various combinations of plasmids. Co-IP analysis was performed to examine the specific binding domains of NMMHC IIA and IFNAR1 (*n* = 3 independent cultures). **g** Immunoblot for IFNAR1 in membrane and cytoplasm of HUVECs treated with *MYH9* siRNA, plasmid or inhibitor (blebbistatin) (*n* = 6 independent cultures). **h** IP analysis for internalized IFNAR1 in HUVECs treated with *MYH9* siRNA, plasmid or inhibitor (blebbistatin) (*n* = 6 independent cultures). **i** Co-IP analysis and quantification of the interaction between NMMHC IIA and IFNAR1 after IFN-β treatment for various durations in HUVECs (*n* = 6 independent cultures). **j** Visualization and quantification of the interaction between NMMHC IIA and IFNAR1 after IFN-β treatment for various durations in HUVECs using the proximity ligation assay (PLA) (red) (*n* = 6 independent cultures). Nuclei were stained with DAPI (blue). Scale bar = 20 μm. All data were represented as the mean ± s.d. Statistical analysis was performed using one-way ANOVA with Tukey post-hoc test (**g–j**). Exact *P* values are indicated in the figure. Source data are provided as a Source Data file.

NMMHC IIA interacts with various proteins to exert its functions^32–34^. To understand how NMMHC IIA mediates USP28-potentiated endothelial necroptosis, we performed co-immunoprecipitation (co-IP) assays to examine whether NMMHC IIA interacts with IFNAR1/2. We found that in HUVECs endogenous NMMHC IIA co-precipitated with IFNAR1, but not with IFNAR2 (Fig. 6a); this was further confirmed by IP/MS analysis (Fig. 6b). Complementarily, we also confirmed an interaction between ectopically expressed Flag-tagged NMMHC IIA and HA-tagged IFNAR1 in HEK-293T cells (Fig. 6c). Mapping NMMHC IIA-IFNAR1 interaction motifs revealed that the N-terminal (1–80) and head domain (81–778) of NMMHC IIA as well as the cytoplasmic domain (458–557) of IFNAR1 were required for their interaction (Fig. 6d–f). As a membrane receptor, IFNAR1 requires trafficking to the plasma membrane after synthesis to exert its function^35^. We found that NMMHC IIA silencing or pharmacological inhibition reduced the membrane expression of IFNAR1, whereas NMMHC IIA overexpression enhanced it (Fig. 6g). In contrast, IFNAR1 internalization was not affected by NMMHC IIA silencing, pharmacological inhibition, or overexpression (Fig. 6h). Notably, we observed that NMMHC IIA binds to IFNAR1 under physiological conditions but undergoes rapid dissociation upon IFN-I stimulation (Fig. 6i, j). Overall, these results indicate that USP28–NMMHC IIA axis potentiates endothelial necroptosis via IFNAR1/STAT1 signaling.

### NMMHC IIA overexpression abolishes the protective effects of USP28 deficiency in sepsis

**Fig. 7:**
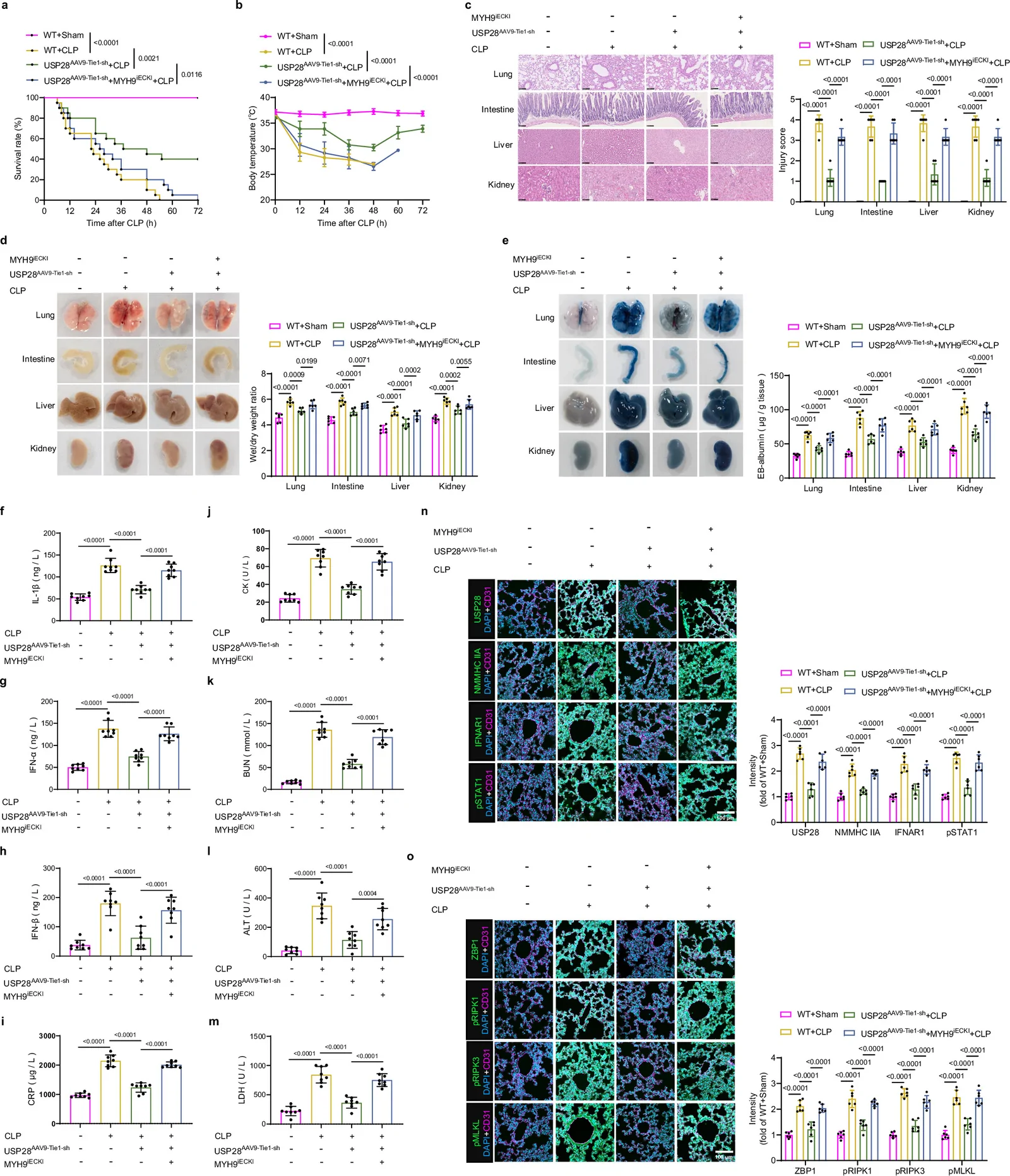
Endothelial-specific NMMHC IIA overexpression largely abrogates the protective effect of USP28 deficiency in polymicrobial sepsis. Wild-type (WT) and *MYH9*^iECKI^ mice were intravenously injected with rAAV9-tie1-vector, and *MYH9*^iECKI^ + *USP28*^AAV9-Tie1-sh^ mice with rAAV9-tie1-shUSP28. All groups were then treated with cecal ligation and puncture (CLP) or sham surgery. **a** Survival rate (*n* = 20 independent mice). **b** Body temperature (*n* = 6 independent mice). **c** Hematoxylin and eosin (H&E) staining and injury score of lung, intestine, liver, and kidney tissues (*n* = 6 independent mice). Scale bar = 100 μm. **d** Representative gross morphology and wet/dry ratio of lung, intestine, liver, and kidney tissue (*n* = 6 independent mice). **e** Evaluation of vascular leakages using Evans blue tracer. Leakage degree was quantified by detecting the Evans blue dye contents in lung, intestine, liver, and kidney homogenate (*n* = 6 independent mice). **f–h** Quantitation of IL-1β (**f**), IFN-α (**g**), and IFN-β (**h**) in plasma (*n* = 8 independent mice). **i–m** Quantitation of CRP (**i**), CK (**j**), BUN (**k**), ALT (**l**), and LDH (**m**) in plasma (*n* = 8 independent mice). **n, o** Representative immunofluorescent staining images and quantification of USP28, NMMHC IIA, IFNAR1, pSTAT1, ZBP1, pRIPK1, pRIPK3, and pMLKL in endothelium of the lung tissues (*n* = 6 independent mice). The target proteins were stained in green. Endothelial cells were stained with CD31 (magenta), and nuclei were stained with DAPI (blue). Scale bar = 100 μm. All data were represented as the mean ± s.d. Statistical analysis was performed using two-way ANOVA with Tukey post hoc test (**b–o**), and survival analysis using the two-sided log-rank (Mantel–Cox) test (**a**). Exact *P* values are indicated in the figure. Source data are provided as a Source Data file.

To further verify whether USP28 deficiency improves sepsis through regulating NMMHC IIA in mice, we constructed endothelial-specific NMMHC IIA overexpression (*MYH9*^iECKI^, Supplementary Fig. 11a, b) and administered *USP28*^AAV9-Tie1-sh^ via tail vein injection. *MYH9*^*iECKI*^ did not affect baseline physiological conditions in sham-treated mice (Supplementary Fig. 11c–n). As anticipated, *USP28*^AAV9-Tie1-sh^ improved the survival and hypothermia of septic mice, while *MYH9*^iECKI^ reversed the protective effects of USP28 deficiency (Fig. 7a, b). Likewise, *USP28*^AAV9-Tie1-sh^ significantly attenuated pathological injury, tissue edema, and vascular leakage of multiple organs (lung, intestine, liver, and kidney), as well as plasma pro-inflammatory cytokines and DAMPs in septic mice, while these protective effects were reversed by *MYH9*^iECKI^ (Fig. 7c–m). Moreover, *USP28*^AAV9-Tie1-sh^ inhibited the activation of IFN-I signaling and necroptosis in pulmonary ECs of septic mice, while *MYH9*^iECKI^ reversed these effects (Fig. 7n, o). Consistently, in IFN-β-treated MLECs, *USP28*^AAV9-Tie1-sh^ reduced the elevated expression of IFN-I signaling-associated proteins and necroptosis-associated proteins, whereas NMMHC IIA overexpression restored these responses (Supplementary Fig. 12a–c). Collectively, these results suggest that USP28 deficiency preserves endothelial function and attenuates sepsis via regulating NMMHC IIA.

### USP28–NMMHC IIA axis is subject to a positive feedback regulation by IFN-I signaling

**Fig. 8:**
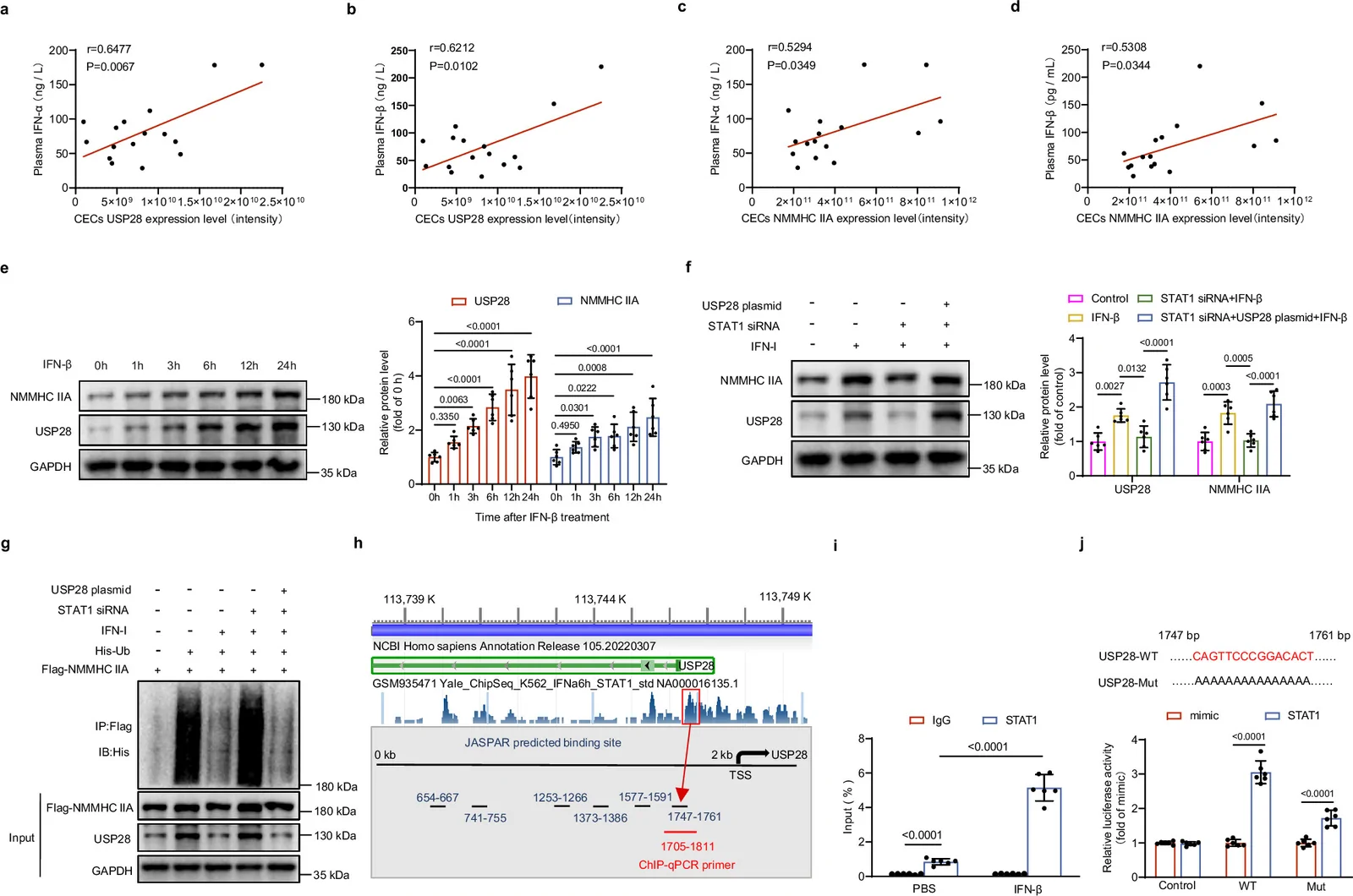
IFN-I signaling has a positive feedback regulation on USP28–NMMHC IIA axis. **a–d** Correlation analysis for the circulating endothelial cells (CECs) USP28 and NMMHC IIA level with the IFN-α and IFN-β level of sepsis patients (*n* = 16 patients). **e** Immunoblot and quantification of USP28 and NMMHC IIA in HUVECs at 0, 3, 6, 12 and 24 h after IFN-β stimulation (*n* = 6 independent cultures). **f** HUVECs were transfected with *STAT1* siRNA or *USP28* plasmid, and then treated with IFN-β. Immunoblot and quantification of USP28 and NMMHC IIA in HUVECs (*n* = 6 independent cultures). **g** HUVECs were pretreated with Flag-NMMHC IIA, His-Ub, *STAT1* siRNA or *USP28* plasmid and further stimulated with IFN-β. NMMHC IIA ubiquitination levels were detected by immunoprecipitation (IP) analysis (*n* = 6 independent cultures). **h** Potential binding motifs for STAT1 in *USP28* promoter regions were predicted by using the JASPAR database. The accurate binding sites were also spotted via STAT1 chromatin immunoprecipitation sequencing (ChIP-Seq) data and used to design the chromatin immunoprecipitation–quantitative PCR (ChIP-qPCR) primer (red). **i** ChIP-qPCR quantification of DNA sequences from the STAT1-binding region of the *USP28* promoter in HUVECs (*n* = 6 independent cultures). **j** Graphical presentation showing the sequence of the mutated STAT1 binding site of the *USP28* promoter region and dual luciferase reporter assay showing STAT1 promotes transcriptional activation of USP28 through binding the specific region (*n* = 6 independent cultures). All data were represented as the mea*n* ± s.d. Statistical analysis was performed using one-way ANOVA (**e**,** f**) and two-way ANOVA with Tukey post-hoc test (**i**,** j**), and correlation analysis using two-sided Spearman correlation coefficient (**a–d**). Exact *P* values are indicated in the figure. Source data are provided as a Source Data file.

Finally, we further investigated the mechanism underlying the upregulation of endothelial USP28 during sepsis. We observed that the expression of USP28 and NMMHC IIA in CECs was positively correlated with plasma IFN-α/β concentrations in patients with sepsis (Fig. 8a–d). Consistent with this, the expression of USP28 and NMMHC IIA in HUVECs was increased in response to IFN-β in a time-dependent manner (Fig. 8e). Based on our previous results demonstrating that USP28 inhibits NMMHC IIA degradation (Fig. 5), we hypothesized that IFN-I signaling upregulates NMMHC IIA through USP28-mediated stabilization. As expected, the IFN-β-induced upregulation of NMMHC IIA and reduction of NMMHC IIA ubiquitination were restored by STAT1 silencing, while STAT1 overexpression further enhanced these effects (Supplementary Fig. 13a–d). Forced overexpression of USP28 reversed the inhibitory effect of STAT1 silencing on IFN-β-induced reduction of NMMHC IIA ubiquitination (Fig. 8f, g).

Subsequently, we sought to elucidate the mechanism by which IFN-I signaling upregulates USP28. Notably, the IFN-β-induced upregulation of USP28 expression was suppressed by STAT1 silencing, while STAT1 overexpression further enhanced this effect (Supplementary Fig. 13c). Since STAT1 is a transcription factor (TF), we further hypothesized that STAT1 transcriptionally activates USP28 to enhance its expression. Guided by the integration of STAT1 motif prediction (JASPAR, https://jaspar.elixir.no/) and chromatin immunoprecipitation (ChIP)-seq data (GSM935471), we designed specific primers for ChIP-qPCR analysis (Fig. 8h). ChIP-qPCR analysis showed that STAT1 directly bound to the promoter region (1705–1811) of USP28, and IFN-β stimulation significantly increased the binding (Fig. 8i). To further define the STAT1-binding site within *USP28* promoter region, we constructed psiCHECK-2 plasmids carrying specific mutations (1747–1761) in the *USP28* promoter (Fig. 8j). Dual-luciferase assay revealed a significant decrease in luciferase activity after mutation (Fig. 8j). Overall, these results indicate that IFN-I signaling has a positive feedback regulation on USP28–NMMHC IIA axis.

## Discussion

In the present study, our results delineated USP28 as a detrimental deubiquitinating enzyme in sepsis. Endothelial USP28 expression is upregulated in septic mice and patients with sepsis, showing a positive correlation with the SOFA score of patients. Endothelial-specific USP28 deficiency alleviated multi-organ injury in septic mice, whereas endothelial-specific USP28 overexpression exacerbated it. A comprehensive scRNA-seq analysis revealed that USP28 deficiency suppressed IFN-I signaling and necroptosis in pulmonary ECs of septic mice. Mechanistically, we showed that USP28 K48-linked deubiquitinates NMMHC IIA at the K1413 site, which enhances the NMMHC IIA-IFNAR1 interaction and consequently exacerbates IFN-I signaling activation, ultimately potentiating endothelial necroptosis. Conversely, the upregulation of USP28 and NMMHC IIA in sepsis is driven by STAT1-mediated transcriptional activation of USP28 following IFN-I stimulation. Finally, we demonstrated that endothelial-specific IFNAR1 or NMMHC IIA overexpression reverses the protective effects of USP28 deficiency in sepsis. Consequently, our findings identify a USP28–NMMHC IIA axis that contributes to EC necroptosis and multi-organ injury during sepsis and suggest that targeting USP28 may represent a potential therapeutic target in sepsis (Supplementary Fig. 14).

Endothelial dysfunction is a major contributor to multi-organ failure and poor prognostic outcomes in sepsis^13,36^. During the early stages of sepsis, excessive immune responses cause extensive damage to ECs, with studies showing a 20% loss of pulmonary vascular ECs at 24 h and 40% at 48 h post-CLP^36,37^. However, the precise mechanisms underlying early-phase endothelial injury remain incompletely understood, with limited access to vascular tissues in patients with sepsis. CECs are mature endothelial cells that detach from the vessel wall into the bloodstream due to vascular injury or physiological remodeling. As such, they serve as indicators of local vascular status, are recognized as reliable markers of endothelial dysfunction in pathological conditions, and provide a potential window into the molecular status of the vasculature in vivo^38,39^. However, the scarcity of CECs in peripheral blood has made it challenging to characterize their protein profile using conventional methods. Nanoproteomics refers to mass-spectrometry–based proteomic strategies designed to analyze extremely small amounts of protein material, enabling the characterization of low-abundance proteoforms and rare cell populations^40–44^. Here, our nanoproteomics analysis revealed that USP28 is markedly upregulated in CECs from patients with sepsis, particularly in non-survivors, and positively correlates with SOFA scores, identifying USP28 as a clinically relevant mediator of sepsis-induced multi-organ injury.

DUBs and DUB-mediated deubiquitinating modification play important roles in modulating intracellular protein stability and function, then regulating a series of pathophysiological processes^45^. Maintaining the intracellular protein quality control by ubiquitination modification is crucial in determining sepsis pathophysiology^46–48^. However, so far, the role of USP28 in sepsis remains entirely unknown. This study identified EC-derived USP28 as an important factor involved in sepsis-induced multi-organ injury. Consistent with our nanoproteomics findings, we found that USP28 was markedly upregulated in the lung, intestine, liver, and kidney tissues of CLP-induced septic mice. Importantly, the upregulated USP28 was predominantly localized to ECs in the lungs of septic mice. Endothelial-specific USP28 deficiency or pharmacological inhibition of USP28 alleviated multi-organ injury in septic mice, whereas endothelial-specific USP28 overexpression exacerbated the injury. Considering the pathogenic role of USP28 in septic ECs, we believe that endothelial-specific gene therapy or small-molecule inhibitors targeting USP28 may represent a potential strategy for therapeutic intervention in sepsis.

Given the inherent complexity of sepsis pathophysiology, the role of IFN-I signaling in sepsis remains highly controversial. While IFN-I signaling promotes pathogen clearance, excessive early activation can exacerbate inflammation and tissue injury. IFN-I signaling blockade exerts protective effects in murine sepsis models and ameliorates sepsis-induced endothelial dysfunction^49–51^. In contrast, IFN-β administration has been reported to improve outcomes in certain settings, such as sepsis-induced acute respiratory distress syndrome (ARDS)^52–54^, although clinical trials have failed to demonstrate significant benefits in mortality or ventilator-free days^55,56^. These seemingly contradictory findings highlight the dual and context-dependent nature of IFN-I signaling. In this study, we found that IFN-I signaling mediates USP28-driven EC necroptosis, thereby contributing to multi-organ injury during sepsis. Through scRNA-seq analysis, we observed that *USP28*^ECKO^ markedly alleviated pulmonary EC loss in septic mice, a process associated with IFN-I signaling and necroptotic pathways. Previous studies have demonstrated that IFN-I specifically induces necroptosis through activation of the ZBP1–MLKL signaling pathway^28–32^. Consistent with these findings, our results indicate that activation of IFN-I signaling specifically triggers EC necroptosis, and this process is tightly regulated by USP28. Thus, our findings indicate that sustained IFN-I activation in the context of CLP-induced sepsis promotes endothelial necroptosis and contributes to multi-organ injury, which may help explain the conflicting outcomes observed in experimental and clinical studies of IFN-I-targeted therapies.

NMMHC IIA belongs to the myosin superfamily and regulates cell migration, adhesion, and vesicle trafficking^57,58^. Our previous work showed that NMMHC IIA regulates pulmonary endothelial integrity during LPS-induced lung injury^32^, but its role in sepsis remains unclear. In this study, we found that NMMHC IIA serves as the key downstream molecule through which USP28 amplifies IFN-I signaling in sepsis. Specifically, we show that USP28 directly binds to NMMHC IIA and removes the K48-linked ubiquitin from the K1413 site of the NMMHC IIA protein. In this process, the ubiquitin C-terminal hydrolase domain of USP28 and the C-terminal domain of NMMHC IIA are crucial for the interaction. Subsequently, NMMHC IIA further enhances the activation of the IFNAR1/STAT1 signaling by interacting with IFNAR1, which is mediated by the N-terminal and head domain of NMMHC IIA and the cytoplasmic domain of IFNAR1. Finally, endothelial-specific IFNAR1 or NMMHC IIA overexpression abolishes the protective effects of USP28 deficiency in sepsis. In conclusion, these findings identify a USP28–NMMHC IIA regulatory axis that links deubiquitination-dependent stabilization of NMMHC IIA to IFNAR1/STAT1-driven endothelial necroptosis during sepsis.

Our study had some limitations. First, experiments were performed in young male mice, whereas both age and sex are important determinants of sepsis susceptibility and outcomes. Therefore, further investigations in aged and female animals are required to determine the generalizability of the USP28–NMMHC IIA axis in sepsis. Second, although our study was primarily focused on the role of the USP28–NMMHC IIA axis in ECs during sepsis, and mechanistic validation was mainly performed in pulmonary ECs, whether this mechanism operates in other cell types or exhibits organ-specific features across different vascular beds remains to be elucidated. Third, this study was conducted in a relatively small clinical cohort. Although the data support the clinical relevance of our findings, the limited sample size restricts the statistical power and precludes comprehensive multivariable analyses to control for potential confounding factors such as age, comorbidities, or clinical interventions. In addition, the ECs analyzed in this study were not directly isolated from patient tissues but were instead derived from CECs obtained from peripheral blood. Although the applied sorting strategy was designed to enrich endothelial-lineage cells, the extremely low abundance of CECs and the limitations of rare-cell isolation may introduce low-level contamination that cannot be completely excluded. Therefore, the consistency of these findings with tissue-resident ECs warrants further validation.

In conclusion, this study showed that USP28 exacerbates sepsis-induced multi-organ injury by inhibiting ubiquitination and degradation of NMMHC IIA. The USP28–NMMHC IIA axis further activates IFNAR1/STAT1/necroptosis signaling, leading to EC necroptosis in sepsis. Therefore, these findings suggest that the USP28 may represent a potential target for therapeutic intervention in sepsis.

## Methods

### Ethics statement

All research was conducted in accordance with relevant ethical regulations. The human study protocol was approved by the Independent Ethics Committee for Clinical Research of Zhongda Hospital, Affiliated to Southeast University (2023ZDSYLL468-P01). Written informed consent was obtained directly from participants who were able to provide consent. For participants who were unable to provide informed consent at the time of enrollment because they were unconscious or under medically required sedation, including sedation administered during invasive mechanical ventilation, written informed consent was obtained from their legally authorized representatives prior to enrollment. All animal experiments followed protocols approved by the Animal Ethics Committee of China Pharmaceutical University (Certificate No. 2024-12-031).

### Blood specimen collection from human participants

The study was conducted in accordance with the ethical principles of the Declaration of Helsinki. No participant compensation was provided. All enrolled patients met the Sepsis-3 clinical criteria^1^. Patients who were pregnant or breastfeeding, diagnosed with malignancy, organ transplantation, HIV infection, autoimmune disease, or who were using immunosuppressive medications were excluded from the study^59^. The study included 16 patients with sepsis (10 males and 6 females; age, 63.9 ± 14.8 years) and 16 healthy controls (6 males and 10 females; age, 55.8 ± 11.5 years). Additional population characteristics are provided in Supplementary Table 1. Participant sex was recorded from medical records as male or female. Sex was not used as an inclusion or exclusion criterion, and both male and female participants were enrolled. The trial recruited female and male participants without any prejudice. Sex-specific subgroup analyses were not performed because the relatively small cohort size did not provide sufficient statistical power for meaningful sex-stratified analyses. Blood samples were collected from sepsis patients within 24 h of enrollment in EDTA tubes, and peripheral blood mononuclear cells (PBMCs) were isolated. The first harvested 3 mL tube was excluded from the analysis to avoid the effects of the vascular damage caused by venipuncture. PBMCs were stained with cell surface and intracellular activation markers to define CEC populations using high-dimensional spectral flow cytometry.

### Mice

All mouse lines were Mus musculus on the C57BL/6 J genetic background. Male mice aged 8–10 weeks were used for all animal experiments unless otherwise indicated. Only male mice were used in this study to minimize sex-related variability and maintain consistency across experiments; therefore, sex was not included as a biological variable in the study design or statistical analysis. The number of animals used in each individual experiment is provided in the corresponding figure legends and Source Data. Endothelial-specific NMMHC IIA knockin (*MYH9*^iECKI^) mice were obtained from Nanjing Biomedical Research Institute of Nanjing University (Certificate number was SCXK Jiangsu 2023–0009). USP28 endothelial-specific knockout (*USP28*^ECKO^) mice were obtained from Cyagen Bioscience (Certificate SCXK Jiangsu 2020-0006). C57BL/6 J male mice were obtained from Jiangsu Huachuang Xinnuo Pharmaceutical Technology (Certificate SCXK 2020–0009). To generate *USP28*^AAV9-Tie1^, *USP28*^AAV9-Tie1-sh^ and *IFNAR1*^AAV9-Tie1^ mice, AAV9 vectors carrying USP28, USP28 shRNA, or IFNAR1 under the Tie1 promoter were administered to mice via tail vein injection three weeks before experiments. All mice were allowed free access to food (Jiangsu Xietong Pharmaceutical Bio-engineering Co., Ltd., Cat#XTI01JX-002) and water and were housed under 12 h light and dark cycles at room temperature (23–25 °C) with relative humidity around 40–60%. All mice were maintained in a specific-pathogen-free (SPF) facility. Mice were randomly assigned to experimental groups, and sample sizes were determined based on previous studies and pilot experiments^60^. Investigators were not blinded to group allocation due to the use of genetically modified mouse models; however, data analysis was performed in a blinded manner where feasible to minimize bias. Humane endpoints were predefined, and mice were euthanized if they exhibited severe hypothermia, inability to access food or water, or moribund condition. All animal experiments followed the protocols approved by the Animal Ethics Committee of China Pharmaceutical University (Certificate No. 2024-12-031).

### CECs isolation

CEC isolation was performed using the following procedure^61,62^. The blood drawn from the subjects was mixed with Ficoll solution and centrifuged at 500 × *g* at 20 °C for 25 min. After centrifugation, the PBMCs were carefully aspirated and transferred to new centrifuge tubes, and the plasma was preserved for subsequent research. PBMCs were incubated with Fc receptor blockers (1 µg/10^6^ cells, BD Biosciences) after washing with PBS. Subsequently, PBMCs were stained in the dark with Hoechst 33342 (1:100, Beyotime, Cat#C1029), 7-AAD (1:1000, Beyotime, Cat#ST515), anti-CD45-APC (10 µl/10^6^ cells, Proteintech, Cat#APC-65064), anti-CD34-FITC (20 µl/10^6^ cells, Proteintech, Cat#FITC-65183) and anti-CD146-PE (10 µl/10^6^ cells, Proteintech, Cat#PE-65181). Prior to marker-based gating, standard FSC/SSC-based singlet discrimination was applied to exclude cell aggregates and doublets. The CEC populations were defined as Hoechst 33342^+^/7-AAD^−^/CD45^−^/CD34^+^/CD146^+^ cells and were sorted using a fluorescence-activated cell sorter (FACS Aria Fusion, Becton Dickinson and Company).

### Adeno-associated virus and viral delivery protocol

To specifically overexpress USP28 and IFNAR1 in the ECs, mice received a single intravenous injection of AAV9 encoding the mouse *USP28* and *IFNAR1* gene under the Tie1 promoter (rAAV9-Tie1-USP28 and rAAV9-Tie1-IFNAR1) via the tail vein. AAV9 carrying without USP28 or IFNAR1 gene (rAAV9-Tie1-Vector) was used as a control vector. To specifically knock down USP28 in ECs, mice received a single intravenous injection of AAV9 vectors containing shRNA sequences targeting the coding sequence (CDS) region of the *USP28* gene, driven by the Tie1 promoter (rAAV9-Tie1-shUSP28). A AAV9 vector carrying shRNA targeting a scrambled sequence (AAV9-Tie1-Vector) was used as a control. The viral vectors were manufactured by Genomeditech (Shanghai, China). The experimental group received tail vein injections of rAAV9-Tie1-USP28 (5 × 10^11^ vg, 200 μl), rAAV9-Tie1-IFNAR1 (5 × 10^11^ vg, 200 μl), or rAAV9-Tie1-Vector (200 μl).

### CLP-induced polymicrobial sepsis model

The CLP-induced polymicrobial sepsis model was established as follows^63^. Mice were anesthetized with 2–3% isoflurane (Reward Life Technology, Cat#R510-22-10) in 100% oxygen with a delivery rate of 1 L/min through a vaporizer. After confirming the loss of reflexes, the abdomen was shaved and disinfected with alcohol. A midline incision of 1–2 cm was performed with a scalpel blade, and the cecum was delivered to a sterile operative field on the abdominal surface. A 4–0 silk suture was used to ligate approximately 75% of the cecum measured from the ileocecal valve. In contrast, for the experiments presented in Supplementary Fig. 4, a milder CLP protocol was used, in which approximately 50% of the cecum was ligated to avoid excessive mortality and ensure that disease severity could be evaluated within an appropriate dynamic range^64^. A 20 G needle was used to puncture a single hole in the ligated cecum without damaging the mesenteric blood vessels. The cecum was then squeezed to expel a small bead of fecal material and returned to the peritoneal cavity. The abdominal incision was sutured in two layers, using 6–0 silk suture for muscles and 4–0 silk suture for skin. Following surgery, mice were administered a subcutaneous injection of 1 mL pre-warmed saline to assist in recovery. Sham-operated mice underwent a similar procedure but without CLP. The survival rate and body temperature of mice were monitored until 72 h. For all assays, mice were euthanized at 24 h post-CLP.

### Isolation of primary vascular endothelial cells

Primary endothelial cells were isolated from mouse lung, liver, kidney, and intestine tissues using the following procedure^65–67^. Briefly, mice were euthanized and transcardially perfused with ice-cold PBS to remove circulating blood. Tissues (lung, liver, kidney, and intestine) were harvested, minced into small fragments, and subjected to enzymatic digestion in KnockOut DMEM (Thermo Fisher Scientific, Cat#10829018) supplemented with collagenase type I, II, or IV (0.1%, Thermo Fisher Scientific, Cat#17018029, Cat#17101015, or Cat#17104019) and DNase I (7.5 mg/mL, Sigma-Aldrich, Cat#D4527). The digested tissues were filtered through 70 μm cell strainers (Corning, Cat#352350), and single-cell suspensions were collected and washed with PBS containing 0.5% bovine serum albumin (Sigma-Aldrich, Cat#10735086001) and 2 mM EDTA (Sigma-Aldrich, Cat#ED2P). Endothelial cells were enriched using CD31 MicroBeads (Miltenyi Biotec, Cat#130-097-418) according to the manufacturer’s instructions. The CD31^+^ cell fraction was collected using LS columns (Miltenyi Biotec, Cat#130-042-401) and subsequently used for downstream experiments. The purity of endothelial cells was confirmed by immunofluorescence analysis.

### Vascular leakage analysis

Mice were intravenously injected with 50 mg/kg Evans blue (EB)-albumin (Sigma-Aldrich, Cat#E2129) via the tail vein at 24 h after CLP and perfused 2 h later with PBS containing 5 mM EDTA-2Na on the right ventricle to remove the intravascular dye. Lung, intestine, liver, and kidney tissues were collected, flash-frozen in liquid nitrogen, and homogenized in a tenfold volume of formamide. The homogenate was incubated at 60 °C for 18 h. After centrifugation at 5000 × *g* for 30 min, the absorption of EB in the supernatant was measured at 620 nm, and the EB content was measured with a standard curve.

### Determination of organ edema

The lung, intestine, liver, and kidney tissues were taken out quickly at 24 h after CLP and their wet weights were accurately measured. After dried in an oven at 60 °C for 72 h, recording the dry weight. The wet weight to dry weight ratio was calculated to determine the degree of multi-organ edema.

### Histopathological evaluation

The lung, intestine, liver, and kidney samples were immersed in the 4% formaldehyde solution at 4 °C overnight and then processed through graded alcohols and xylene. Subsequently, the tissues were embedded in paraffin and cut into 5 μm sections with a vibratome (Leica, HistoCore BIOCUT). The sections were deparaffinized in xylene, rehydrated in 100–70% gradient ethanol, and stained with hematoxylin-eosin (H&E) staining. Pathological images were examined using an optical microscope (Hamamatsu, NanoZoomer 2.0-RS), and the extent of the injury was scored by pathologists.

### Immunohistochemical analysis

For immunohistochemical analysis, tissue sections were prepared as described above for histological evaluation. After dewaxing and hydration, sections were subjected to antigen repair by microwave heating (10–15 min) in citrate buffer. Sections were incubated with 3% hydrogen peroxide for 15 min to suppress endogenous peroxidase and blocked with 5% Goat Serum for an hour at room temperature to prevent non-specific background staining. Sections were then incubated with USP28 antibodies (Thermo Fisher Scientific, Cat#PA5-52347) in PBS at 4 °C overnight. Next, Sections were further processed with anti-rabbit EnVision™+/HRP reagent (Gene Technology, China) at 37 °C. One hour later, the samples were visualized by using diaminobenzidine (Gene Technology, China). Images were captured with an Olympus BX41 microscope (Olympus, Milan, Italy) equipped with an Olympus DP50 camera at a magnification of 20×.

### Plasma pro-inflammatory factors and DAMPs contents

Plasma IL-1β (Proteintech, Cat#KE10003), IL-6 (NeoBioscience, Cat#EMC004.96), TNF-α (NeoBioscience, Cat#EMC102a.96), IFN-α (Solarbio, Cat#SEKM-0359), IFN-β (Solarbio, Cat#SEKM-0032), CRP (Proteintech, Cat#KE10128), CK (Nanjing Jiancheng Bioengineering Institute, Cat#A032-1-1), LDH (Nanjing Jiancheng Bioengineering Institute, Cat#A020-2-2), BUN (Nanjing Jiancheng Bioengineering Institute, Cat#C013-2-1), and ALT (Nanjing Jiancheng Bioengineering Institute, Cat#C009-2-1) were measured by colorimetric assays or enzyme-linked immunosorbent assay (ELIZA) in accordance with the manufacturer’s instruction.

### Immunofluorescence

After perfusion with PBS and 4% paraformaldehyd, tissues were placed into 4% paraformaldehyde at 4 °C overnight. Tissues were dehydrated in 30% sucrose for 3 days, then embedded in OCT and frozen at −70 °C. Tissues were sliced to a thickness of 10 μm with a cryotome (CM1950, Leica) and placed onto adhesive microscope slides (Citoglas, Cat#80313-7161-16). Following permeabilization with 0.3% Triton X-100 in PBS, samples were blocked with 5% normal donkey serum for 30 min at room temperature. Subsequently, primary antibody incubation was performed overnight at 4 °C, followed by three times of washing with PBS containing 0.1% Tween-20. Fluorescent secondary antibodies were then applied for 1 h at room temperature under light-protected conditions. DAPI (Beyotime, Cat#C1006) was used to stain nuclei. Fluorescent images were observed with a confocal laser scanning microscope (LSM800, Zeiss). The primary antibodies utilized were: NMMHC IIA (Proteintech, Cat#11128-1-AP), USP28 (Proteintech, Cat#17707-1-AP), IFNAR1 (Proteintech, Cat#13083-1-AP), pSTAT1 (Affinity Biosciences, Cat#AF3300), ZBP1 (ABclonal, Cat#A28338), pRIPK1 (Thermo Fisher Scientific, Cat#PA5-104645), pRIPK3 (Cell Signaling Technology, Cat#91702), pMLKL (Cell Signaling Technology, Cat#37333), CD31 (R&D Systems, Cat#AF3628), Ki67 (Proteintech, Cat#28074-1-AP).

### Western blot and immunoprecipitation

Samples were lysed by using RIPA buffer (Beyotime, Cat#P0013E) with an inhibitor cocktail (Beyotime, Cat#P1006) and boiled 10 min with SDS loading buffer (Beyotime, Cat#P0015). Protein samples (20 μg per well) were then fractionated using SDS-PAGE and transferred to PVDF membranes (Merck Millipore, Cat#HVLP00010). The membranes were treated with 5% BSA at room temperature for 2 h to block, and then incubated with primary antibodies: NMMHC IIA (Proteintech, Cat#11128-1-AP), USP28 (Proteintech, Cat#17707-1-AP), IFNAR1 (Proteintech, Cat#13083-1-AP), pSTAT1 (Affinity Biosciences, Cat#AF3300), ZBP1 (ABclonal, Cat#A28338), RIPK1 (Abcam, Cat#ab300617), pRIPK1 (Thermo Fisher Scientific, Cat#PA5-104645), RIPK3 (Thermo Fisher Scientific, Cat#703750), pRIPK3 (Cell Signaling Technology, Cat#91702), MLKL (Proteintech, Cat#66675-1-Ig), pMLKL (Cell Signaling Technology, Cat#37333), GSDMD-N/GSDMD (ABclonal, Cat#A20197SP), Cleaved Caspase 3 (Proteintech, Cat#82707-13-RR), Caspase 3 (Proteintech, Cat#66470-2-Ig), ubiquitin (Proteintech, Cat#10201-2-AP), His tag (Proteintech, Cat# 66005-1-Ig), HA tag (Proteintech, Cat#51064-2-AP), Flag tag (Proteintech, Cat#20543-1-AP), GFP tag (Proteintech, Cat#50430-2-AP), Myc tag (Proteintech, Cat# 60003-2-Ig), GAPDH (Bioworld, Cat#MB001), β-actin (Proteintech, Cat#66009-1-Ig), ATP1A1 (Proteintech, Cat#14418-1-AP). After three times of washing with fresh TBS-T for 15 min each, the membranes were incubated with secondary anti-rabbit (Bioworld, Cat#BS20241-Y) or anti-mouse (Bioworld, Cat#BS20242-Y) IgG conjugated with horseradish peroxidase (1:5000 dilution). Enhanced chemiluminescence (ECL) reagents were used to visualize the protein membranes, and signal quantification was performed with the detection system (Bio-Rad Laboratories, USA). Densitometric analysis of the gels was done using Image Lab software. Uncropped and unprocessed scans of the blots are provided in the Source Data file and, for Supplementary Figs., at the end of the Supplementary Information.

For IP/co-IP analysis, samples were harvested and lysed in lysis buffer (Beyotime, Cat#P0037) for 30 min on ice and centrifuged at 4 °C for 20 min at 12,000 × *g*. Then supernatants were incubated with anti- primary antibodies (1:50) antibody or IgG (1 μg) at 4 °C overnight on a shaker. The next day, Protein A/G PLUS-Agarose (Santa Cruz Biotechnology, Cat#sc-2003) beads were rinsed 5 times with lysis buffer (Beyotime, Cat#P0037). Then added SDS loading buffer to the samples and boiled 10 min. Samples were analyzed by Western blot.

### IP-MS analysis

For each IP-MS experiment, one biological sample was analyzed with one technical replicate, with a matched IgG immunoprecipitation serving as the negative control. Samples were harvested and lysed in lysis buffer (Beyotime, Cat#P0037) for 30 min on ice and centrifuged at 12,000 × *g* for 20 min at 4 °C. The supernatants were incubated with the indicated primary antibodies (2 μg) or control IgG (2 μg) overnight at 4 °C with gentle rotation. On the following day, Protein A/G PLUS-Agarose beads (Santa Cruz Biotechnology, Cat#sc-2003) were washed five times with lysis buffer and then incubated with the antibody–protein complexes. After extensive washing, bound proteins were eluted by resuspending the beads in SDS–DTT–Tris lysis buffer, followed by boiling for 10 min. Eluates were centrifuged, and the supernatants were collected for downstream proteomic analysis. The eluates were subjected to filter-aided sample preparation (FASP). Protein samples were reduced with DTT to a final concentration of 100 mM at 95 °C for 5 min, diluted with UA buffer (8 M urea, 150 mM Tris-HCl, pH 8.5), and transferred to 10-kDa centrifugal filter units. After repeated washing with UA buffer, proteins were alkylated with 100 mM iodoacetamide for 30 min at room temperature in the dark. The filters were subsequently washed with UA buffer and 25 mM ammonium bicarbonate, and proteins were digested with 4 μg trypsin at 37 °C for 16–18 h. Peptides were collected by centrifugation and subjected to LC–MS/MS analysis.

Peptide samples were subjected to LC-MS/MS analysis on a Q Exactive mass spectrometer (Thermo Fisher Scientific) coupled to an Easy nLC system (Thermo Fisher Scientific). Peptides were loaded onto a reverse-phase trap column (Thermo Fisher Scientific Acclaim PepMap100, nanoViper C18) and separated on a C18 analytical column (Thermo Fisher Scientific Easy Column) using solvent A (0.1% formic acid in water) and solvent B (84% acetonitrile with 0.1% formic acid) at a flow rate of 300 nL/min. Peptides were separated using a 60-min gradient: 0–55 min, 0–40% solvent B; 55–58 min, 40–100% solvent B; and 58–60 min, 100% solvent B. The mass spectrometer was operated in positive ion mode using data-dependent acquisition. Full MS scans were acquired over an m/z range of 350–1800 at a resolution of 70,000, with an automatic gain control (AGC) target of 3 × 10^6^ and a maximum injection time of 50 ms. The 20 most intense precursor ions from each full MS scan were selected for higher-energy collisional dissociation (HCD) with a normalized collision energy of 27. MS/MS spectra were acquired at a resolution of 17,500 with an AGC target of 1 × 10^5^ and a maximum injection time of 50 ms. Dynamic exclusion was set to 30 s.

Raw MS data files were processed using MaxQuant software (version 2.1.3.0) with the integrated Andromeda search engine. Database searches were performed against the UniProt human reviewed protein database (nr_uniprot-taxonomy_Homo_sapiens_swiss.fasta, 20,308 sequences). Trypsin was specified as the proteolytic enzyme, allowing up to two missed cleavages, with a minimum peptide length of 7 amino acids. Carbamidomethylation of cysteine was set as a fixed modification, whereas oxidation of methionine and acetylation of protein N-termini were specified as variable modifications. The precursor ion mass tolerance was set to 20 ppm, and the fragment ion mass tolerance was set to 0.1 Da. Peptide-spectrum matches and protein identifications were filtered using an FDR threshold of 1% at both the peptide and protein levels. Proteins were required to contain at least one unique peptide for identification. Protein quantification was performed using the iBAQ algorithm implemented in MaxQuant. Identified proteins were further filtered to remove nonspecific binders based on IgG control samples, and the resulting interaction candidates were used for downstream analyses.

### Nanoproteomics of CECs

Nanoproteomics analysis of CECs was performed using the following workflow^68^. A total of 32 CEC samples were analyzed, including 16 samples from healthy controls and 16 samples from patients with sepsis. Among the patients with sepsis, 13 were survivors, and 3 were non-survivors. Each CEC sample represented an independent biological replicate and was analyzed once by LC–MS/MS (technical replicate, *n* = 1). Briefly, FACS-sorted CECs were collected into 0.2 mL maximum-recovery PCR tubes (Axygen). Ammonium bicarbonate buffer and dithiothreitol were added for cell lysis and reduction using water-bath sonication for 15 min. Samples were then alkylated with iodoacetamide for 30 min in the dark and digested with trypsin at 37 °C for 2 h prior to LC–MS/MS analysis. Peptides were analyzed using a Fusion Lumos Tribrid mass spectrometer (Thermo Fisher Scientific) coupled to an UltiMate 3000 UHPLC system. Peptides were separated on an analytical column at 500 nL/min using a gradient of 5–25% buffer B (0.1% formic acid in 98% acetonitrile) over 90 min, 25–35% over 10 min, and 35–80% over 5 min, followed by column washing and equilibration. MS data were acquired in data-dependent acquisition mode with MS1 resolution of 120,000, scan range 350–1500 m/z, AGC target 1 × 10^5^, and maximum injection time 50 ms. MS2 spectra were acquired at 30,000 resolution with AGC target 2 × 10^4^, maximum injection time 50 ms, isolation window 1.6 m/z, and top-N value of 20. Bulk HUVECs samples (*n* = 3) were analyzed to generate a spectral library that assisted peptide identification in the nanoproteomics samples.

Raw MS data were processed using MaxQuant (v2.1.3.0) with the integrated Andromeda search engine against the UniProt human reference proteome database (nr_uniprot-taxonomy_Homo_sapiens_swiss.fasta, 20,308 sequences). Trypsin was specified as the digestion enzyme with a minimum peptide length of 7 amino acids. Up to two missed cleavages were allowed. The precursor mass tolerance was set to 20 ppm for the first search and 4.5 ppm for the main search, and the fragment ion mass tolerance was set to 20 ppm. Proteins were required to contain at least one unique peptide for identification. Carbamidomethyl (C) was set as a fixed modification, while oxidation (M), acetyl (protein N-terminus), and deamidation (NQ) were included as variable modifications. Peptide-spectrum matches were filtered at a false discovery rate (FDR) of 1% at the PSM level, and protein identifications were further filtered at 1% FDR at the protein level. Protein quantification was performed in MaxQuant using label-free quantification (LFQ) with iBAQ enabled. LFQ intensities were used for downstream analysis and internally normalized by the MaxQuant algorithm. Missing values were not imputed, and proteins were analyzed based on the detected values. Differential protein expression between groups was assessed by calculating fold changes and performing Welch’s *t*-test. Proteins with fold change >1.5 and adjusted *P* < 0.05 were considered significantly differentially expressed, where adjusted *P* values were calculated using the Benjamini–Hochberg method to control the FDR.

### Single-cell RNA sequencing

Mouse lung tissue was obtained from wild-type mice and *USP28*^ECKO^ mice at 24 h after CLP surgery. After placing a sterile RNase-free culture dish with ice-cold, calcium-free, and magnesium-free PBS on ice, the tissue was transferred into the dish, minced into 0.5 mm^2^ pieces, and washed with PBS to remove blood clots and adipose tissue. Tissues were dissociated into single cells in dissociation solution (0.35% collagenase IV, 2 mg/ml papain, 120 Units/ml DNase I) in a 37 °C water bath with shaking for 20 min at 100 × rpm. Digestion was terminated with 1× PBS containing 10% fetal bovine serum (FBS, V/V), then pipetting 5–10 times with a Pasteur pipette. The resulting cell suspension was filtered bypassing through 70–30 um stacked cellstrainer and centrifuged at 300 g for 5 min at 4 °C. The cell pellet was resuspended in 100 ul PBS (0.04% BSA) and added with 1 ml 1× red blood cell lysis buffer (Miltenyi Biotec, Cat#130-094-183) and incubated at room temperature or on ice for 2–10 min to lyse remaining red blood cells. After incubation, the suspension was centrifuged at 300 g for 5 min at room temperature. The suspension was resuspended in 100 μl Dead Cell Removal MicroBeads (Miltenyi Biotec, Cat#130-090-101) and dead cells were removed using the Miltenyi ® Dead Cell Removal Kit (Miltenyi Biotec, Cat#130-090-101). Then the suspension was resuspended in 0.04% BSA-PBS and centrifuged at 300 g for 3 min at 4 °C (repeat twice). The cell pellet was resuspended in 50 μl of 0.04% BSA-PBS. The overall cell viability was confirmed by trypan blue exclusion, which needed to be above 85%, single cell suspensions were counted using a hemocytometer/Countess II Automated Cell Counter and concentration adjusted to 700–1200 cells/μl. Single-cell suspensions were loaded onto a DNBelab C-TaiM 4 platform to capture approximately 5000 single cells per sample according to the manufacturer’s instructions. Subsequent cDNA amplification and library construction were performed following the standard protocol. The amplified cDNA was used to prepare short-read sequencing libraries according to the 10x Genomics protocol. Finally, the libraries were sequenced on a DNBSEQ-T7 system (PE100, multiplexed run) with a minimum sequencing depth of 20,000 reads per cell.

Raw sequencing data were processed using Cell Ranger (v8.0.1) for alignment and quantification. Quality control retained cells with 200–10,000 detected genes and mitochondrial UMI proportion <20%, and doublets were removed using DoubletFinder (v2.0.4), yielding 4620 and 6097 high-quality cells for the WT and *USP28*^ECKO^ groups, respectively. Downstream analysis was performed using Seurat (v4.4.0), with normalization by LogNormalize (scale factor = 10000), identification of highly variable genes using the “vst” method, and selection of 3000 shared variable features for PCA. Batch effects were corrected using Harmony (v1.2.1), followed by graph-based clustering with FindNeighbors and FindClusters (resolution = 0.5), and visualization using UMAP. Cell types were annotated using SingleR (v2.4.1) with the MouseRNAseqData reference and further refined based on canonical marker genes. Differential expression analysis was performed using FindAllMarkers with the Wilcoxon rank-sum test, with thresholds of min.pct > 0.1, log2 fold change > 0.25, and adjusted *P* value < 0.05 (Bonferroni correction). Functional enrichment analysis was conducted using clusterProfiler (v4.15.0.1) based on KEGG pathways with a hypergeometric test and adjusted *P* value < 0.05. GSEA analysis was further performed using clusterProfiler based on ranked gene lists derived from differential expression analysis, with pathways showing adjusted *P* value < 0.05 considered significantly enriched.

### Chromatin immunoprecipitation

ChIP was performed using the Pierce™ Magnetic ChIP Kit (Thermo Fisher Scientific, Cat#26157) according to the manufacturer’s instructions. Briefly, HUVECs were crosslinked with 1% formaldehyde for 10 min and quenched with 1× glycine. After washing with ice-cold PBS, cells were harvested and lysed, followed by micrococcal nuclease (MNase) digestion and sonication to obtain chromatin fragments ranging from 200 to 1000 bp. The chromatin was diluted and incubated overnight at 4 °C with an anti-STAT1 antibody (Proteintech, Cat#10144-2-AP) and Protein A/G magnetic beads. After extensive washing, protein-DNA complexes were eluted, and crosslinking was reversed with Proteinase K. DNA was purified using the DNA Clean-Up Columns provided in the kit and analyzed by qPCR. HiScript® Q RT SuperMix (Vazyme, Cat#R223) was employed for RNA transcription, and ChamQTM SYBR® qPCR Master Mix (Vazyme, Cat#Q331-02) was used for quantitative real-time PCR, adhering to the instructions provided by the reagent manufacturers. The ChIP-qPCR signals were normalized to those from the input DNA. The primer sequences for the USP28 promoter region (forward primer: 5’-GCGGAGAAGACTCAGGAACG-3’, and reverse primer: 5’-GTCGCTGTTGGTCAGCTTCA-3’).

### Proximity ligation assay

Detected interactions between NMMHC IIA and IFNAR1 using a PLA kit (Sigma-Aldrich, Cat#DUO92002, Cat#DUO92004, and Cat#DUO92008) according to the manufacturer’s protocols. After incubating samples with primary antibodies against NMMHC IIA and IFNAR1, secondary antibodies coupled with oligonucleotides (PLA probes) were applied for ligation and rolling circle amplification. The images were then examined using a confocal laser scanning microscope (LSM800, Zeiss) and processed using ZEN imaging software.

### Cell culture

HUVECs and HEK-293T cells were purchased from Shanghai Institute of Cell Biology, Chinese Academy of Sciences. Cells were cultured in DMEM (Gibco, Cat#C11965500BT) supplemented with 10% fetal bovine serum (Gibco, Cat#A5669701) and 100 U/mL penicillin/streptomycin (Gibco, Cat#15140122) at 37 °C in a humidified atmosphere of 5% CO^2^. The cells in the model group were stimulated with 300 U/mL IFN-β (Proteintech, Cat#HZ-1298) for 12 h. The control group was cultured with normal medium.

### Plasmid and siRNA transfection

All plasmids used in this study, including expression constructs for USP28, NMMHC IIA, IFNAR1, ubiquitin and their indicated deletion or site-directed mutant derivatives, were generated and supplied by Genomeditech Co. Ltd. (Shanghai, China). The USP28-C171S mutant, NMMHC IIA site-directed mutants, domain-deletion constructs, and IFNAR1 domain constructs were generated by site-directed mutagenesis or molecular cloning based on the corresponding wild-type constructs. The wild-type and STAT1-binding-site mutant USP28 promoter fragments used for the dual-luciferase reporter assay were cloned into the psiCHECK2 vector by Genomeditech Co. Ltd. *MYH9* siRNA (forward, 5’-GAGGCAAUGAUCACUGACU-3’; reverse, 5’-AGUCAGUGAUCAUUGCCUC-3’), *USP28* siRNA (forward, 5’-UUCGGAACAAACUAUAAUCUUC-3’; reverse, 5’- GAAGAUUAUAGUUUGUUCCGAA-3’), *STAT1* siRNA (forward, 5’-GCUGGCCCUGAUGGUCUUA-3’; reverse, 5’-UAAGACCAUCAGGGCCAGC-3’), *MLKL* siRNA (forward, 5’- GGUGUGAAGAGAUGAAAUU-3’; reverse, 5’- AAUUUCAUCUCUUCACACC-3’) and *RIPK3* siRNA (forward, 5’-CUGAGAGACAAGGCAUGAACU-3’; reverse, 5’-AGUUCAUGCCUUGUCUCUCAG-3’) were synthesized by Biomics Biotechnologies (Nantong, China). Cell transfections with plasmids or siRNAs were carried out using Lipofectamine 3000 (Thermo Fisher Scientific, Cat#L3000015) or Lipofectamine RNAiMAX (Thermo Fisher Scientific, Cat#13778150) following the manufacturer’s guidelines. Plasmids and other newly generated materials described in this study are available from the corresponding authors upon reasonable request.

### Membrane protein fractionation

Membrane and cytosolic protein fractions were isolated using a membrane protein extraction kit (Beyotime, Cat#P0033) according to the manufacturer’s instructions. Briefly, HUVECs were lysed in extraction buffer A supplemented with PMSF and homogenized on ice. After removal of nuclei and debris by differential centrifugation, the supernatant was further processed to separate cytosolic and membrane fractions. The membrane pellet was subsequently extracted using buffer B to obtain the membrane protein fraction. The obtained fractions were subjected to Western blot analysis.

### Cell surface biotinylation and internalization assay

For the internalization assay^69^, HUVECs were washed with ice-cold PBS and incubated with Sulfo-NHS-SS-Biotin sodium (1 mg/mL, MedChemExpress, Cat#HY-111496) for 1 h at 4 °C with gentle shaking to label surface proteins. Excess biotin was quenched by washing with PBS containing 100 mM glycine. Cells were then returned to 37 °C for the indicated times to allow internalization. After internalization, remaining surface biotin was removed using a membrane-impermeable reducing buffer containing 100 mM sodium 2-mercaptoethanesulfonate (MesNa, MedChemExpress, Cat#HY-13679). HUVECs were subsequently lysed in RIPA buffer (Beyotime, Cat#P0013D) and incubated with NeutrAvidin agarose beads (Thermo Fisher Scientific, Cat#29200) at 4 °C overnight to capture biotinylated proteins.

### Dual-luciferase reporter assay

Dual-luciferase reporter assays were performed in HEK-293T cells using the Dual-Luciferase® Reporter Assay Kit (Beyotime, Cat#RG009S) according to the manufacturer’s instructions. The *USP28* promoter region (wild-type or STAT1 binding site mutant) was cloned into the psiCHECK2 vector (Genomeditech Co. Ltd). HEK-293T cells were seeded in 24-well plates and transfected with 500 ng of the respective reporter plasmids using Lipofectamine 3000 (Thermo Fisher Scientific, Cat#L3000015). After 24–48 h, firefly and Renilla luciferase activities were measured using a microplate luminometer. Renilla luciferase activity, driven by the *USP28* promoter constructs, was normalized to firefly luciferase activity to control for transfection efficiency.

### Transendothelial electrical resistance assays and EB-albumin leakage assays

HUVECs were seeded on transwell inserts (0.4 μm pore, 6.5 mm diameter, Millipore, Cat#PTHT24H48) for 7 days. The TEER of the monolayer was measured with the Millicell-ERS voltohmeter (Millipore, Cat#MERS00002). Resistance values of multiple transwell inserts of an experimental group were measured sequentially, and the mean was expressed in the common unit (Ω · cm^2^) after subtraction of the value of a blank cell-free filter. The TEER of the monolayers was recorded when a stable resistance reading was achieved with triplicate measurements that were taken for each transwell. IFN-β (300 U/mL) was added to the upper and lower chambers for 12 h. The inner chamber fluid was changed to 200 μL of EB-albumin solution as the tracer, and the outer chamber fluid was changed to 600 μL of 4% BSA solution and incubated in an incubator for 1 h. Collect the extracellular fluid, add 200 μL per well to the 96-well plate, measure the absorbance at 620 nm, and calculate the content of EB-albumin leakage according to the EB curve.

### Statistical analysis

GraphPad Prism software (version 8, GraphPad Software Inc.) was used for statistical analysis. Data are shown as mean ± standard deviation (SD), with biological replicates indicated by the number of individuals (n). Significance was measured using Student’s *t*-test (two-tailed) for comparisons between two groups and the Mann–Whitney U test for data that were not normally distributed. For comparisons involving three or more groups, one- or two-way analysis of variance was employed, followed by the Tukey post-hoc test. Survival curves were analyzed using the Kaplan–Meier method and compared using the log-rank (Mantel–Cox) test. The Spearman correlation coefficient was used to evaluate the correlations. All tests were two-sided, and statistical significance was set at *P* < 0.05.

### Reporting summary

Further information on research design is available in the Nature Portfolio Reporting Summary linked to this article.

## Supplementary information

Supplementary Information

Reporting Summary

Transparent Peer Review file

## Source data

Source Data

## Data Availability

The single-cell RNA-sequencing raw data generated in this study have been deposited in the NCBI Sequence Read Archive (SRA) under BioProject accession number PRJNA1379272. The raw nanoproteomics mass spectrometry data have been deposited in the iProX repository (a ProteomeXchange partner repository) under accession code PXD071841. The raw IP-MS data have also been deposited in the iProX repository under accession code PXD071842. The previously published STAT1 ChIP-seq data used in this study are available in the Gene Expression Omnibus under accession code GSM935471. Source data are provided with this paper. All other data supporting the findings of this study are available from the corresponding authors upon request, without restrictions. Source data are provided with this paper.
