# Supplementary material for: USP28 potentiates endothelial necroptosis via deubiquitinating NMMHC IIA in septic male mice
Source: Nat Commun. 2026 Oct 15;17:11667. doi: 10.1038/s41467-026-78666-8 (PMC13759511; doi:10.1038/s41467-026-78666-8)
Supplement: Supplementary file 1 [file 41467_2026_78666_MOESM1_ESM.pdf]

Supplementary Information

USP28 potentiates endothelial necroptosis via deubiquitinating NMMHC IIA in septic male mice

Authors: Jiahui Tang[1], Dingji Hu[2], Mengchen Xu[1], Haiyu Chen[1], Shuaishuai Gong[1], Fang Li[1], Boyang Yu[1], Yuanyuan Zhang[1,*], Songqiao Liu[2,3,*], Junping Kou[1,*]

Affiliations:

[1]Jiangsu Key Laboratory of TCM Evaluation and Translational Research, Department of Pharmacology of Chinese Materia Medica, School of Traditional Chinese Pharmacy, China Pharmaceutical University, Nanjing 211198, Jiangsu, China

[2]Jiangsu Provincial Key Laboratory of Critical Care Medicine, Department of Critical Care Medicine, Trauma Center, Zhongda Hospital, School of Medicine, Southeast University, Nanjing 210009, Jiangsu, China

[3]The First People's Hospital of Lianyungang, The Affiliated Lianyungang Hospital of Xuzhou Medical University, Lianyungang 222000, Jiangsu, China

[*]**Corresponding authors:** Junping Kou, e-mail: junpingkou@cpu.edu.cn;

Songqiao Liu, e-mail: liusongqiao@ymail.com;

Yuanyuan Zhang, e-mail: yuanyuanzhang@cpu.edu.cn.

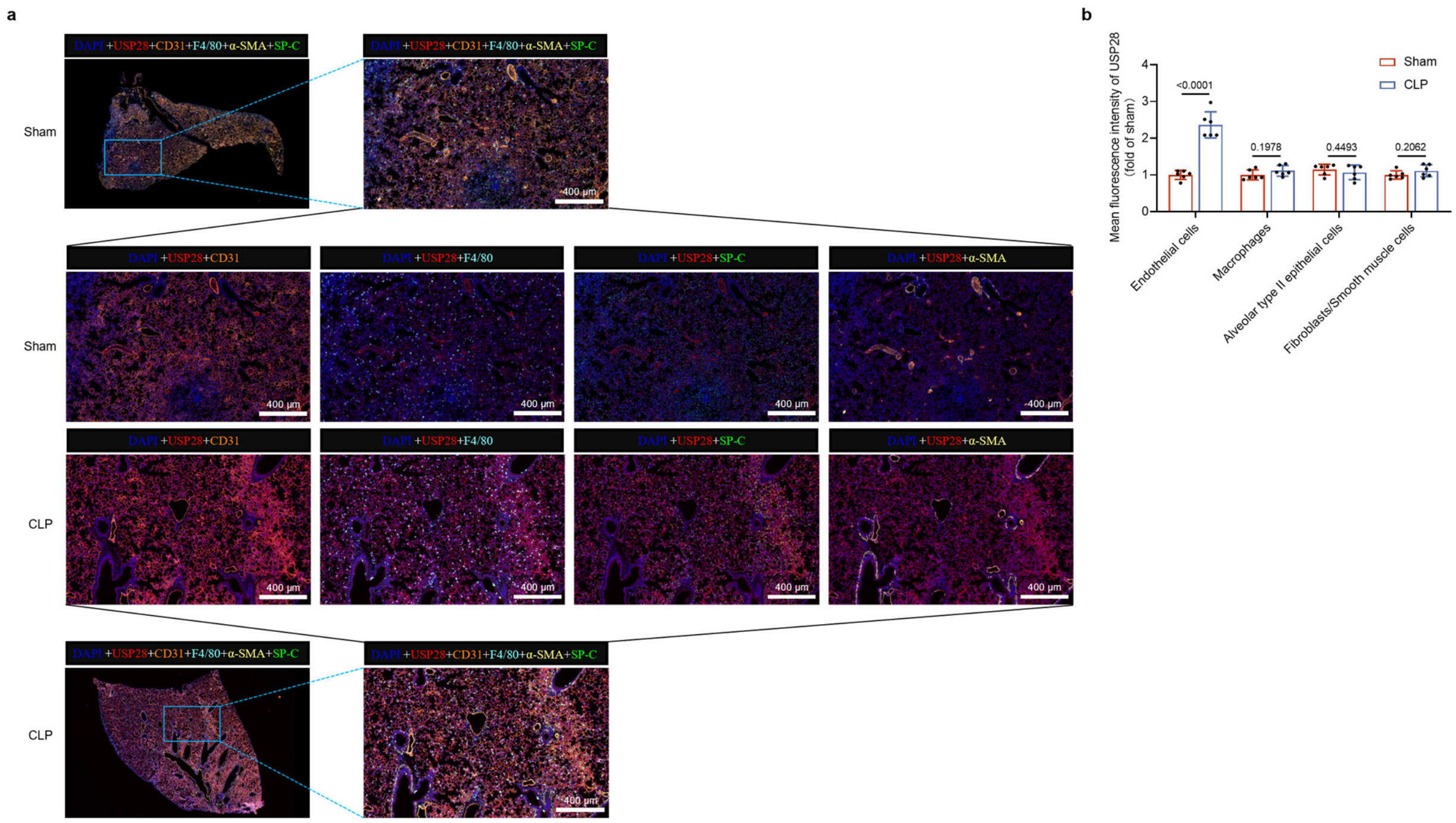

Supplementary Figure 1. Endothelial-specific upregulation of USP28 in CLP-induced septic mice. a, b Representative immunofluorescence staining images and quantification of USP28 expression in pulmonary CD31$^+$ endothelial cells, F4/80$^+$ macrophages, SP-C$^+$ alveolar type II epithelial cells and α-SMA$^+$ fibroblasts/smooth muscle cells from sham- or CLP-treated mice (n = 6 independent mice). Sections were stained for USP28 (red), CD31 (orange), F4/80 (cyan), α-SMA (yellow), SP-C (green), and DAPI (blue). Scale bar = 400 μm. All data were represented as the mean ± standard deviation. Statistical analysis was performed using two-tailed Student's t-test (**b**). Exact *P* values are indicated in the figure. Source data are provided as a Source Data file.

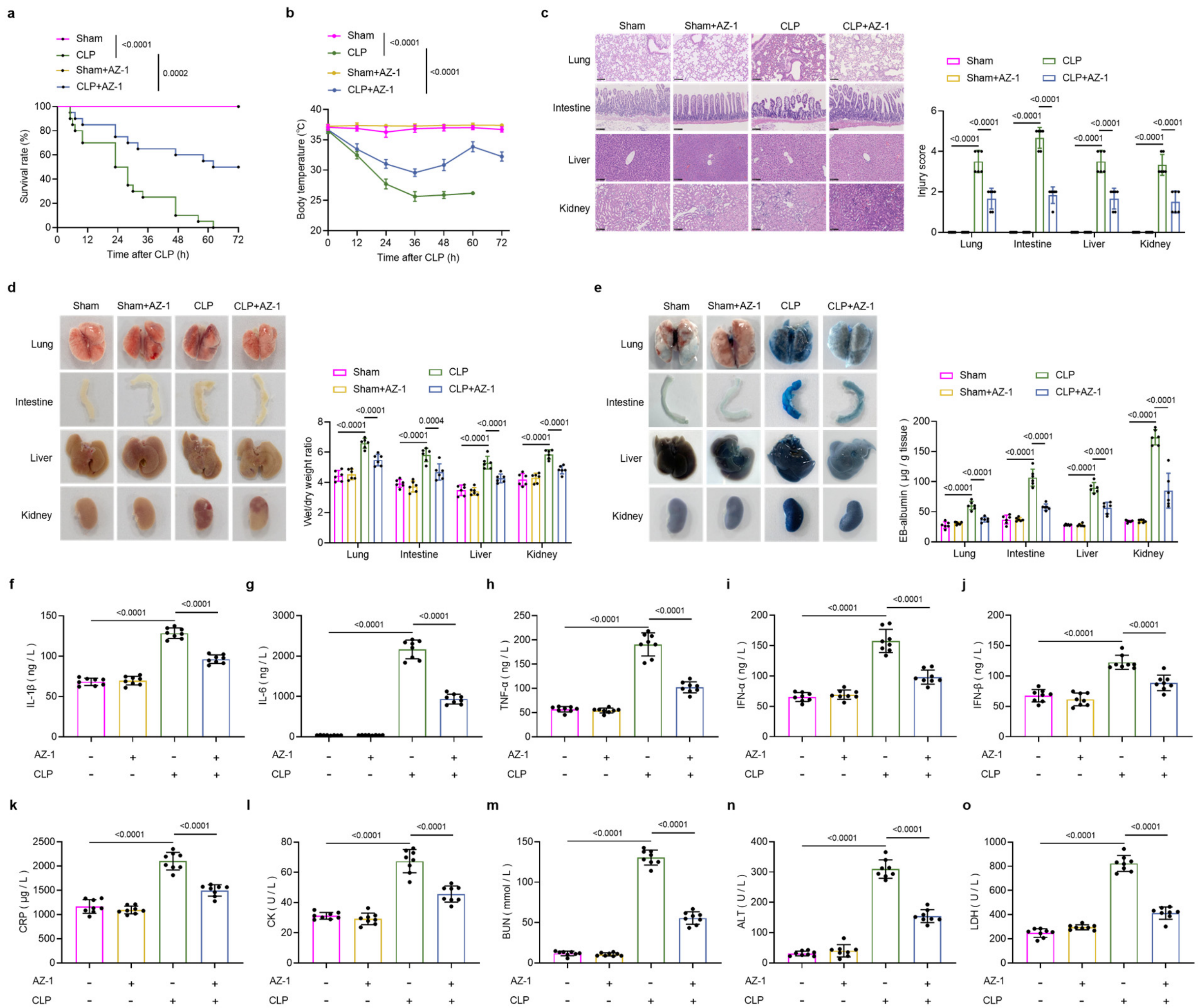

Supplementary Figure 2. Inhibition of USP28 ameliorates multi-organ injury in polymicrobial sepsis. Mice were administered AZ-1 (20 mg/kg) by intragastric gavage immediately prior to cecal ligation and puncture (CLP) or sham surgery. **a** Survival rate (n = 20 independent mice). **b** Body temperature (n = 6 independent mice). **c** Hematoxylin and eosin (H&E) staining and injury score of lung, intestine, liver, and kidney tissues (n = 6 independent mice). Scale bar = 100 μm. **d** Representative gross morphology and wet/dry ratio of lung, intestine, liver, and kidney tissue (n = 6 independent mice). **e** Evaluation of vascular leakages using Evans blue tracer. Leakage degree was quantified by detecting the Evans blue dye contents in lung, intestine, liver, and kidney homogenate (n = 6 independent mice). **f–j** Quantitation of IL-1β (**f**), IL-6 (**g**),

TNF-α (**h**), IFN-α (**i**), and IFN-β (**j**) in plasma (n = 8 independent mice). **k–o** Quantitation of CRP (**k**), CK (**l**), BUN (**m**), ALT (**n**), and LDH (**o**) in plasma (n = 8 independent mice). All data were represented as the mean ± standard deviation. Statistical analysis was performed using two-way ANOVA with Tukey post hoc test (**b–o**), and survival analysis using the two-sided log-rank (Mantel–Cox) test (**a**). Exact *P* values are indicated in the figure. Source data are provided as a Source Data file.

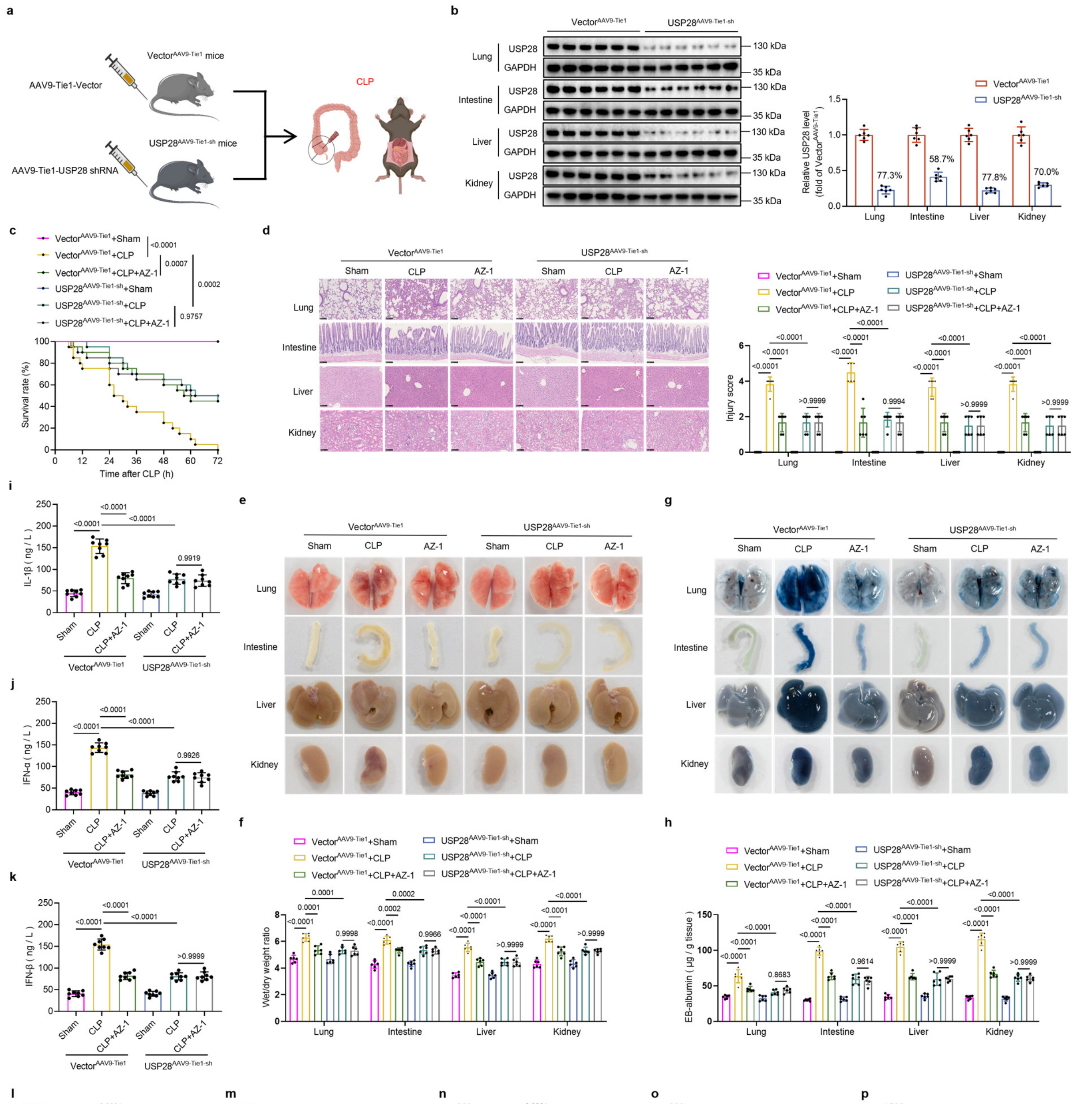

Supplementary Figure 3. **Endothelial-specific USP28 deficiency abrogates the protective effects of AZ-1 in sepsis-induced multi-organ injury.** Vector$^{\text{AAV9-Tie1}}$ mice were intravenously injected with rAAV9-tie1-vector and *USP28*$^{\text{AAV9-Tie1-sh}}$ mice with rAAV9-tie1-shUSP28. Vector$^{\text{AAV9-Tie1}}$ and *USP28*$^{\text{AAV9-Tie1-sh}}$ mice were administered AZ-1 (20 mg/kg) by intragastric

gavage 1 h after cecal ligation and puncture (CLP) or sham surgery. **a** Schematic illustrating generation of $\text{Vector}^{\text{AAV9-Tie1}}$ and $\textit{USP28}^{\text{AAV9-Tie1-sh}}$ mice. Created in BioRender. Tang, J. (2026) https://BioRender.com/vsus0ps. **b** Immunoblot analysis and quantification of USP28 in primary endothelial cells (ECs) isolated from lung, intestine, liver, and kidney of $\text{Vector}^{\text{AAV9-Tie1}}$ and $\textit{USP28}^{\text{AAV9-Tie1-sh}}$ mice (n = 6 independent mice). **c** Survival rate (n = 20 independent mice). **d** Hematoxylin and eosin (H&E) staining and injury score of lung, intestine, liver, and kidney tissues (n = 6 independent mice). Scale bar = 100 μm. **e, f** Representative gross morphology and wet/dry ratio of lung, intestine, liver, and kidney tissue (n = 6 independent mice). **g, h** Evaluation of vascular leakages using Evans blue tracer. Leakage degree was quantified by detecting the Evans blue dye contents in lung, intestine, liver, and kidney homogenate (n = 6 independent mice). **i–k** Quantitation of IL-1β (**i**), IFN-α (**j**), and IFN-β (**k**) in plasma (n = 8 independent mice). **l–p** Quantitation of CRP (**l**), CK (**m**), BUN (**n**), ALT (**o**), and LDH (**p**) in plasma (n = 8 independent mice). All data were represented as the mean ± standard deviation. Statistical analysis was performed using two-way ANOVA with Tukey post hoc test (**d, f, h–p**), and survival analysis using the two-sided log-rank (Mantel–Cox) test (**c**). Exact *P* values are indicated in the figure. Source data are provided as a Source Data file.

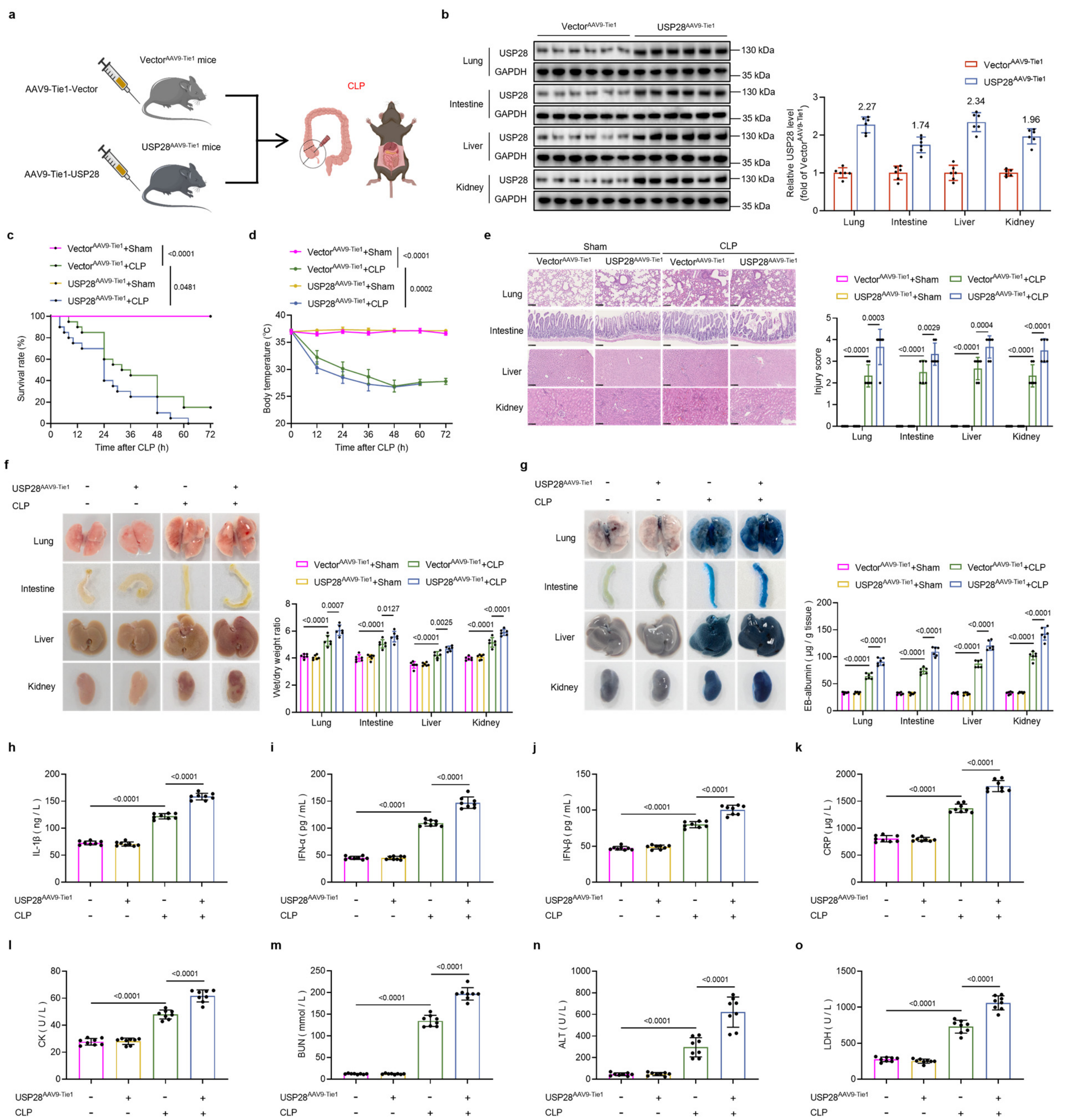

Supplementary Figure 4. **Endothelial-specific USP28 overexpression exacerbates multi-organ injury in polymicrobial sepsis.** Vector$^{AAV9\text{-}Tie1}$ mice were intravenously injected with rAAV9-tie1-vector and *USP28*$^{AAV9\text{-}Tie1}$ mice with rAAV9-tie1-USP28. Vector$^{AAV9\text{-}Tie1}$ and *USP28*$^{AAV9\text{-}Tie1}$ mice were treated with cecal ligation and puncture (CLP) or sham surgery. **a** Schematic illustrating generation of Vector$^{AAV9\text{-}Tie1}$ and *USP28*$^{AAV9\text{-}Tie1}$ mice. Created in BioRender. Tang, J. (2026) https://BioRender.com/o6fc9sz. **b** Immunoblot analysis and

quantification of USP28 in primary endothelial cells (ECs) isolated from lung, intestine, liver, and kidney of Vector$^{AAV9\text{-}Tie1}$ and *USP28*$^{AAV9\text{-}Tie1}$ mice (n = 6 independent mice). **c** Survival rate (n = 20). **d** Body temperature (n = 6 independent mice). **e** Hematoxylin and eosin (H&E) staining and injury score of lung, intestine, liver, and kidney tissues (n = 6 independent mice). Scale bar, 100 μm. **f** Representative gross morphology and wet/dry ratio of lung, intestine, liver, and kidney tissue (n = 6 independent mice). **g** Evaluation of vascular leakages using Evans blue tracer. Leakage degree was quantified by detecting the Evans blue dye contents in lung, intestine, liver, and kidney homogenate (n = 6 independent mice). **h–j** Quantitation of IL-1β (**h**), IFN-α (**i**), and IFN-β (**j**) in plasma (n = 8 independent mice). **k–o** Quantitation of CRP (**k**), CK (**l**), BUN (**m**), ALT (**n**), and LDH (**o**) in plasma (n = 8 independent mice). All data were represented as the mean ± standard deviation. Statistical analysis was performed using two-way ANOVA with Tukey post hoc test (**d–o**), and survival analysis using the two-sided log-rank (Mantel–Cox) test (**c**). Exact *P* values are indicated in the figure. Source data are provided as a Source Data file.

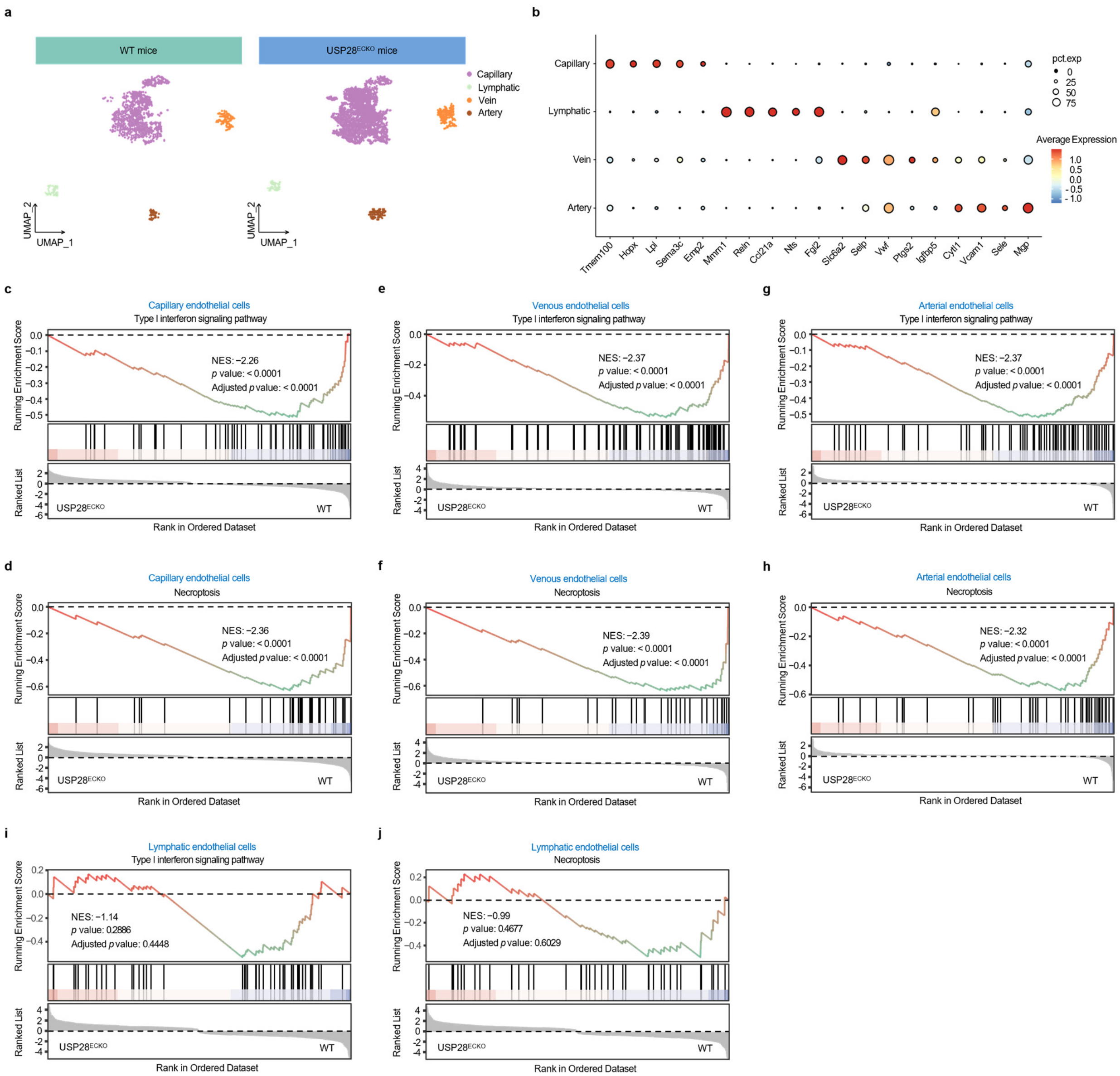

Supplementary Figure 5. **Endothelial-specific USP28 knockout suppresses sepsis-induced necroptosis and IFN-I signaling in vascular endothelial cells. a** Uniform manifold approximation and projection (UMAP) plot of endothelial cells (ECs) subpopulations from the lung tissues of wild-type (WT) and *USP28*^{ECKO} mice after cecal ligation and puncture (CLP) surgery, capillary, venous, arterial, and lymphatic ECs. **b** Dot plot showing the representative marker genes across the capillary, venous, arterial, and lymphatic ECs subpopulations. **c–j** Gene set enrichment analysis (GSEA) analysis of necroptosis and IFN-I signaling pathway across

capillary (**c, d**), venous (**e, f**), arterial (**g, h**), and lymphatic (**i, j**) ECs subpopulations. Exact *P* values are indicated in the figure.

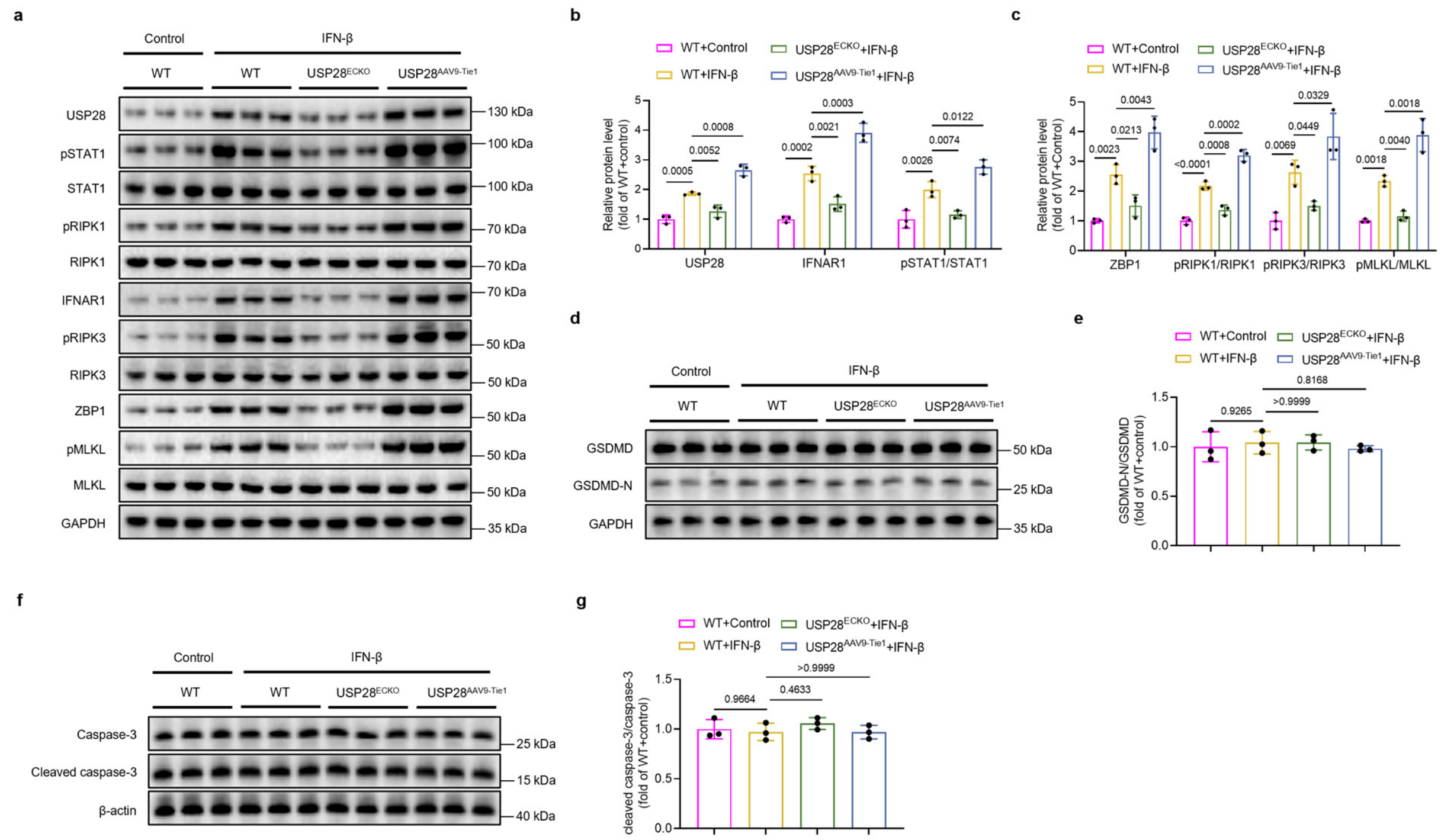

Supplementary Figure 6. **USP28 mediates endothelial necroptosis via IFNAR1/STAT1 signaling in MLECs.** Primary mouse lung endothelial cells (MLECs) isolated from wild-type (WT), *USP28*^{ECKO} and *USP28*$^{\text{AAV9-Tie1}}$ mice were treated with PBS or IFN-β for 12 h. **a–c** Immunoblot analysis and quantification of USP28, IFNAR1, pSTAT1/STAT1, ZBP1, pRIPK1/RIPK1, pRIPK3/RIPK3, and pMLKL/MLKL in MLECs (n = 3 independent cultures). **d, e** Immunoblot analysis and quantification of GSDMD-N/GSDMD in MLECs (n = 3 independent cultures). **f, g** Immunoblot analysis and quantification of cleaved caspase-3/caspase-3 in MLECs (n = 3 independent cultures). All data were represented as the mean ± standard deviation. Statistical analysis was performed using two-way ANOVA with Tukey post hoc test (**b, c, e, g**). Exact *P* values are indicated in the figure. Source data are provided as a Source Data file.

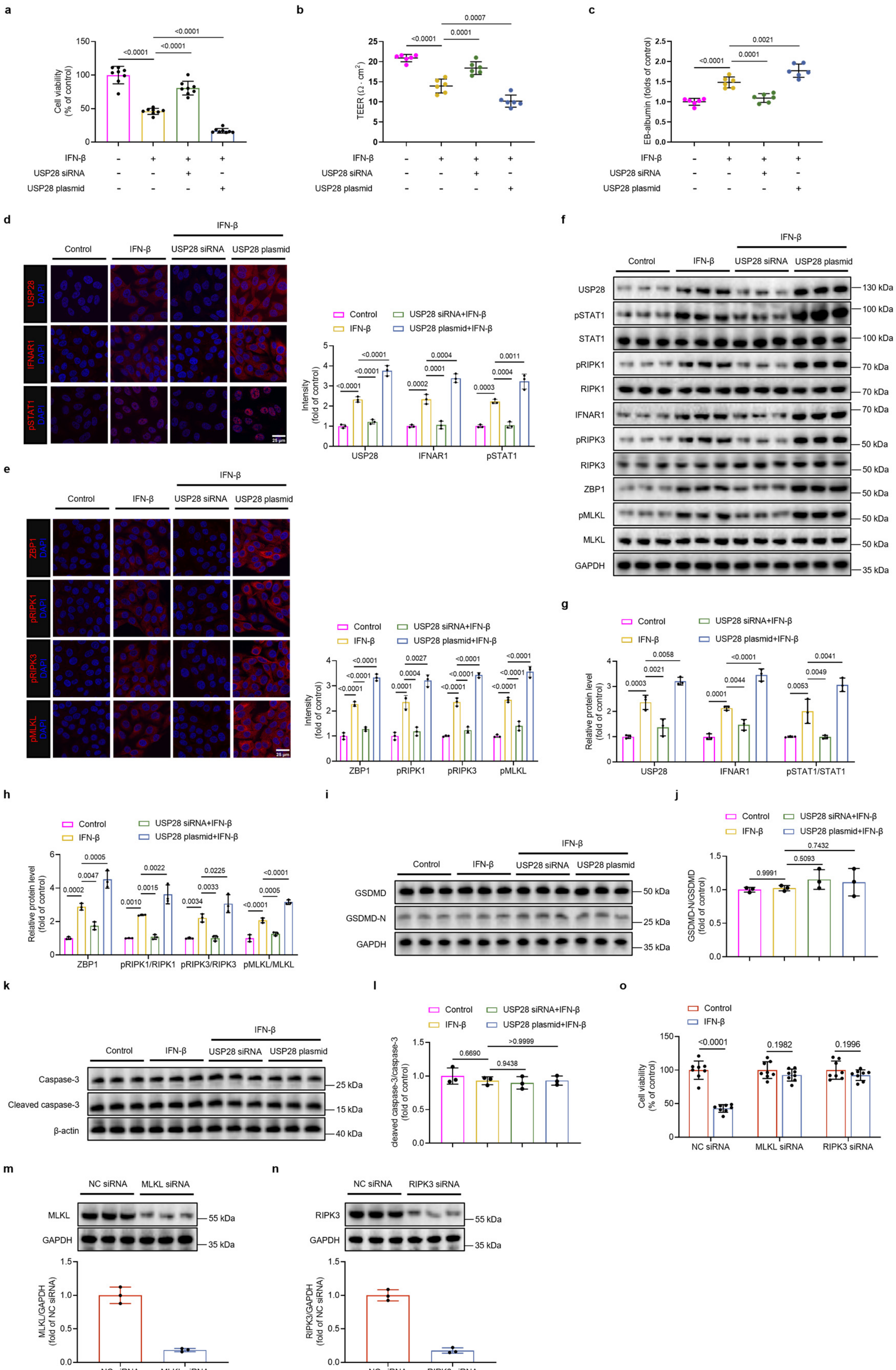

a
b
c
d
e
f
g
h
i
j
k
l
o
m
n
Cell viability (% of control)
TEER (Ω · cm²)
EB-albumin (folds of control)
IFN-β
USP28 siRNA
USP28 plasmid
Control
USP28 siRNA+IFN-β
USP28 plasmid+IFN-β
Intensity (fold of control)
Relative protein level (fold of control)
USP28
IFNAR1
pSTAT1
STAT1
pRIPK1
RIPK1
pRIPK3
RIPK3
ZBP1
pMLKL
MLKL
GAPDH
pSTAT1/STAT1
pRIPK1/RIPK1
pRIPK3/RIPK3
pMLKL/MLKL
GSDMD
GSDMD-N
GSDMD-N/GSDMD (fold of control)
Caspase-3
Cleaved caspase-3
β-actin
cleaved caspase-3/caspase-3 (fold of control)
NC siRNA
MLKL siRNA
RIPK3 siRNA
MLKL/GAPDH (fold of NC siRNA)
RIPK3/GAPDH (fold of NC siRNA)
130 kDa
100 kDa
70 kDa
50 kDa
35 kDa
25 kDa
15 kDa
40 kDa
55 kDa
25 μm

Supplementary Figure 7. **USP28 mediates endothelial necroptosis via IFNAR1/STAT1 signaling in HUVECs.** Human umbilical vein endothelial cells (HUVECs) were pretreated with *USP28* siRNA or plasmid, and further stimulated with PBS or IFN-β for 12 h. **a–c** Quantification of 3-(4,5-dimethylthiazol-2-yl)-2,5-diphenyltetrazolium bromide (MTT) assay (n = 8 independent cultures) (**a**), transendothelial electrical resistance (TEER) (n = 6 independent cultures) (**b**) and Evans blue leakage (n = 6 independent cultures) (**c**) in HUVECs. **d, e** Representative immunofluorescent staining images and quantification of USP28, IFNAR1, pSTAT1, ZBP1, pRIPK1, pRIPK3, and pMLKL in HUVECs (n = 3 independent cultures). The target proteins were stained in red and nuclei were stained with DAPI (blue). Scale bar = 25 μm. **f–h** Immunoblot analysis and quantification of USP28, IFNAR1, pSTAT1/STAT1, ZBP1, pRIPK1/RIPK1, pRIPK3/RIPK3, and pMLKL/MLKL in HUVECs (n = 3 independent cultures). **i, j** Immunoblot analysis and quantification of GSDMD-N/GSDMD in HUVECs (n = 3 independent cultures). **k, l** Immunoblot analysis and quantification of cleaved caspase-3/caspase-3 in HUVECs (n = 3 independent cultures). **m** HUVECs were treated with negative control (NC) siRNA or *MLKL* siRNA. Immunoblot analysis and quantification of MLKL in HUVECs (n = 3 independent cultures). **n** HUVECs were treated with NC siRNA or *RIPK3* siRNA. Immunoblot analysis and quantification of RIPK3 in HUVECs (n = 3 independent cultures). **o** MTT assay for HUVECs were pretreated with NC siRNA, *MLKL* siRNA or *RIPK3* siRNA, and further stimulated with PBS or IFN-β for 12 h. Cell viability was assessed by MTT assay (n = 8 independent cultures). All data were represented as the mean ± standard deviation. Statistical analysis was performed using two-tailed Student's t-test (**m, n**) and two-way ANOVA with Tukey post hoc test (**a–e, g, h, j, l, o**). Exact *P* values are indicated in the figure. Source data are provided as a Source Data file.

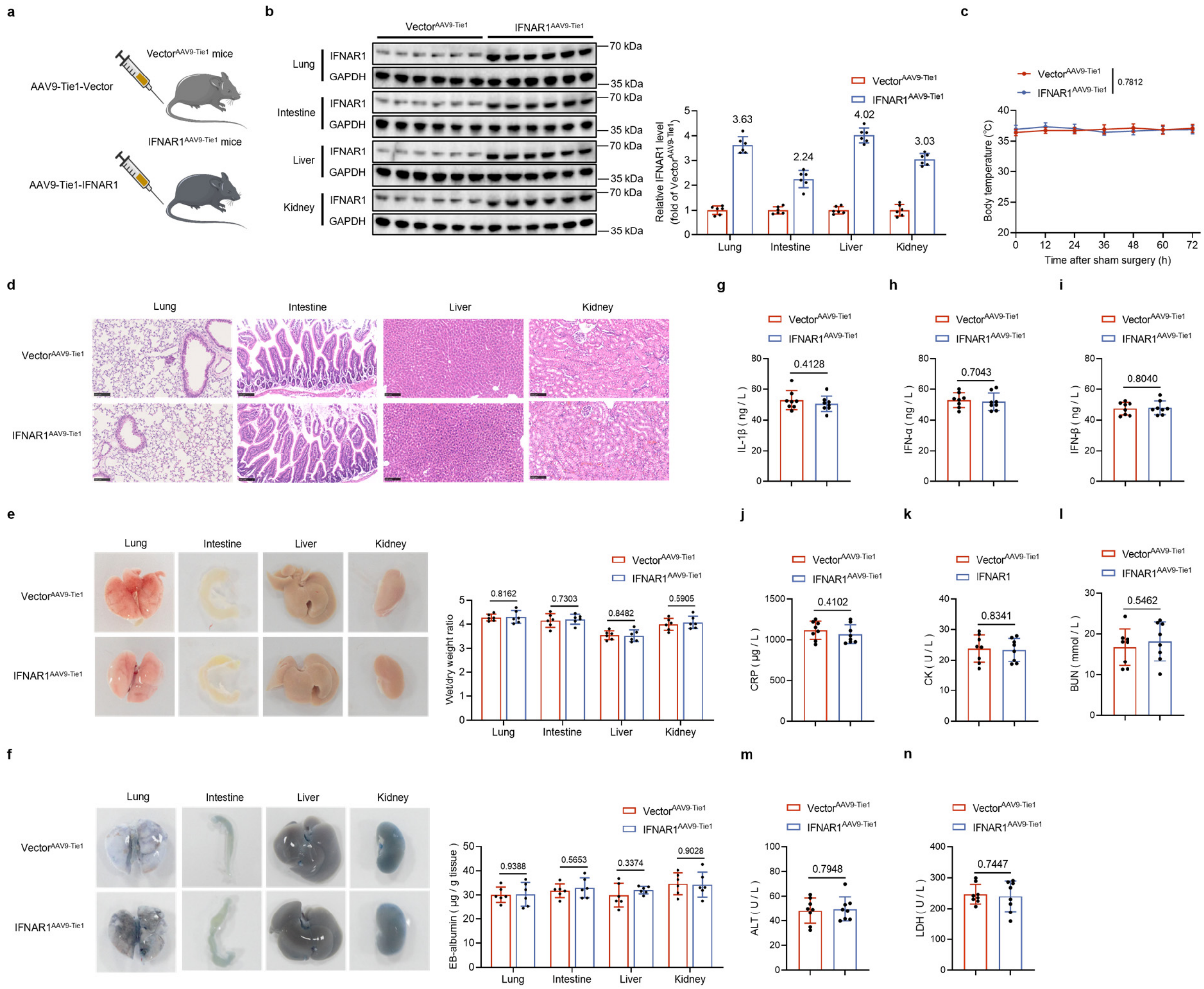

Supplementary Figure 8. **Endothelial-specific IFNAR1 overexpression does not affect basal physiological homeostasis in mice.** Vector$^{AAV9\text{-}Tie1}$ mice were intravenously injected with rAAV9-tie1-vector and *IFNAR1*$^{AAV9\text{-}Tie1}$ mice with rAAV9-tie1-IFNAR1. Vector$^{AAV9\text{-}Tie1}$ and *IFNAR1*$^{AAV9\text{-}Tie1}$ mice were treated with sham surgery. **a** Schematic illustrating generation of Vector$^{AAV9\text{-}Tie1}$ and *IFNAR1*$^{AAV9\text{-}Tie1}$ mice. Created in BioRender. Tang, J. (2026) https://BioRender.com/8jpxj9v. **b** Immunoblot analysis and quantification of IFNAR1 in primary endothelial cells (ECs) isolated from lung, intestine, liver, and kidney of Vector$^{AAV9\text{-}Tie1}$ and *IFNAR1*$^{AAV9\text{-}Tie1}$ mice (n = 6 independent mice). **c** Body temperature (n = 6 independent mice). **d** Hematoxylin and eosin (H&E) staining and injury score of lung, intestine, liver, and kidney tissues (n = 6 independent mice). Scale bar = 100 μm. **e** Representative gross morphology and wet/dry

ratio of lung, intestine, liver, and kidney tissue (n = 6 independent mice). **f** Evaluation of vascular leakages using Evans blue tracer. Leakage degree was quantified by detecting the Evans blue dye contents in lung, intestine, liver, and kidney homogenate (n = 6 independent mice). **g–i** Quantitation of IL-1β (**g**), IFN-α (**h**), and IFN-β (**i**) in plasma (n = 8 independent mice). **j–n** Quantitation of CRP (**j**), CK (**k**), BUN (**l**), ALT (**m**), and LDH (**n**) in plasma (n = 8 independent mice). All data were represented as the mean ± standard deviation. Statistical analysis was performed using two-tailed Student's t-test (**c–n**). Exact *P* values are indicated in the figure. Source data are provided as a Source Data file.

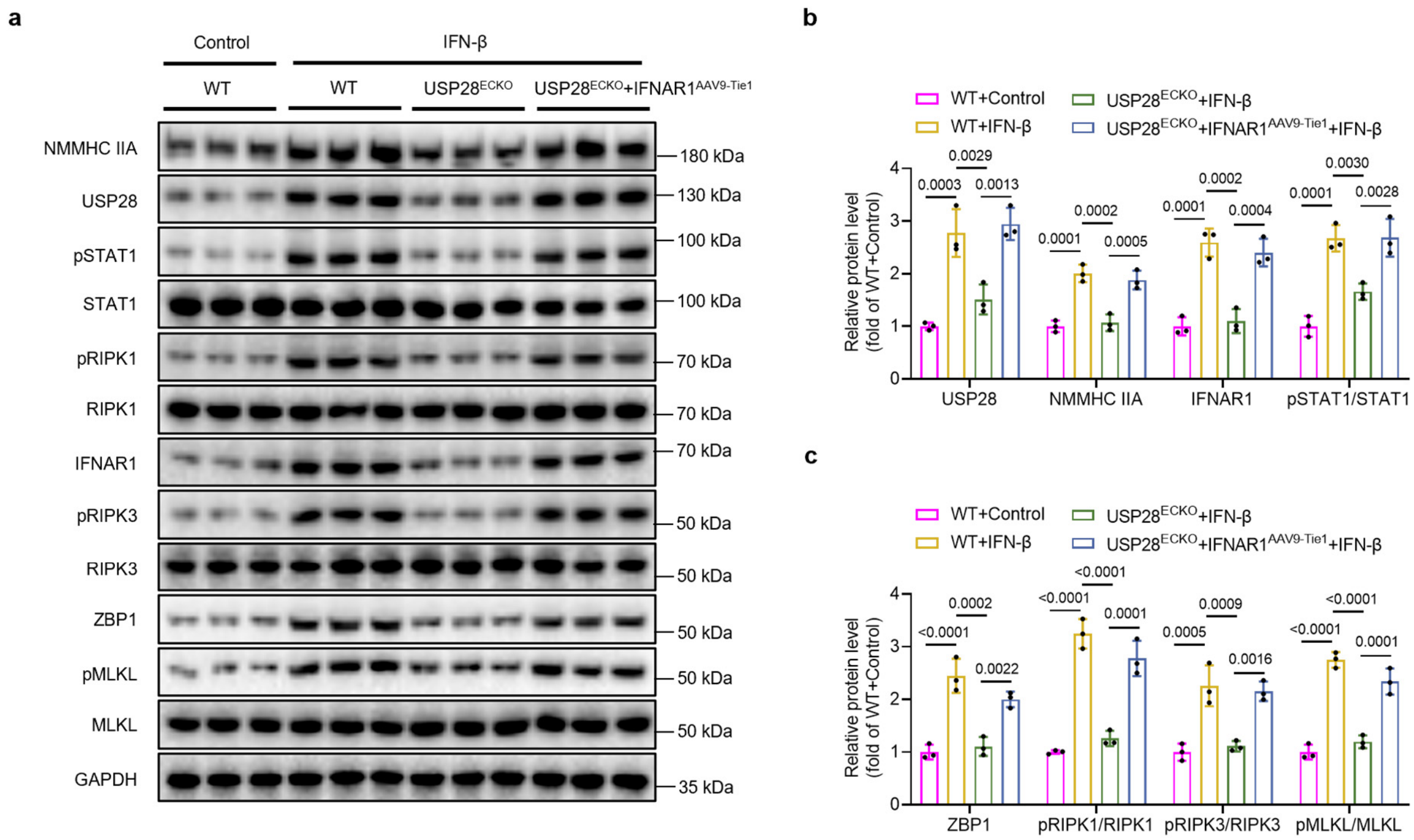

Supplementary Figure 9. Endothelial-specific IFNAR1 overexpression largely abrogates the protective effect of USP28 deficiency on endothelial necroptosis in MLECs. Primary mouse lung endothelial cells (MLECs) isolated from wild-type (WT), *USP28*^{ECKO} and *USP28*$^{\mathrm{AAV9\text{-}Tie1}}$+ *IFNAR1*$^{\mathrm{AAV9\text{-}Tie1}}$ mice were treated with PBS or IFN-β for 12 h. **a–c** Immunoblot analysis and quantification of USP28, NMMHC IIA, IFNAR1, pSTAT1/STAT1, ZBP1, pRIPK1/RIPK1, pRIPK3/RIPK3, and pMLKL/MLKL in MLECs (n = 3 independent cultures). All data were represented as the mean ± standard deviation. Statistical analysis was performed using two-way ANOVA with Tukey post hoc test (**b, c**). Exact *P* values are indicated in the figure. Source data are provided as a Source Data file.

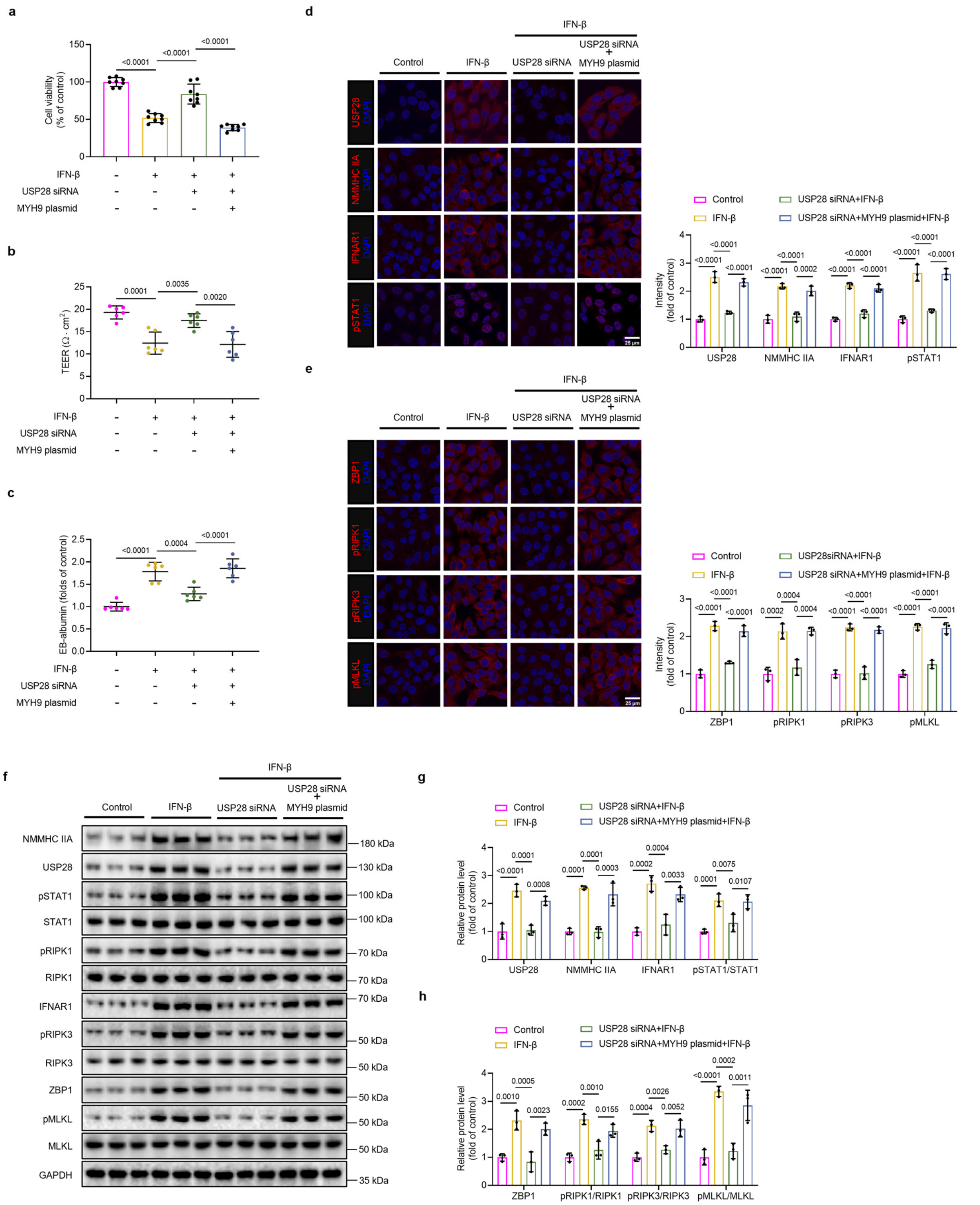

Supplementary Figure 10. **NMMHC IIA overexpression largely abrogates the protective effect of USP28 inhibition on endothelial necroptosis in HUVECs.** Human umbilical vein endothelial cells (HUVECs) were pretreated with *USP28* siRNA or *MYH9* plasmid, and further

stimulated with IFN-β. **a–c** Quantification of 3-(4,5-dimethylthiazol-2-yl)-2,5-diphenyltetrazolium bromide (MTT) assay (n = 8 independent cultures) (**a**), transendothelial electrical resistance (TEER) (n = 6 independent cultures) (**b**) and Evans blue leakage (n = 6 independent cultures) (**c**) in HUVECs. **d, e** Representative immunofluorescent staining images and quantification of USP28, NMMHC IIA, IFNAR1, pSTAT1, ZBP1, pRIPK1, pRIPK3, and pMLKL in HUVECs (n = 3 independent cultures). The target proteins were stained in red and nuclei were stained with DAPI (blue). Scale bar = 25 μm. **f–h** Immunoblot analysis and quantification of USP28, NMMHC IIA, IFNAR1, pSTAT1/STAT1, ZBP1, pRIPK1/RIPK1, pRIPK3/RIPK3, and pMLKL/MLKL in HUVECs (n = 3 independent cultures). All data were represented as the mean ± standard deviation. Statistical analysis was performed using two-way ANOVA with Tukey post hoc test (**a–e, g, h**). Exact *P* values are indicated in the figure. Source data are provided as a Source Data file.

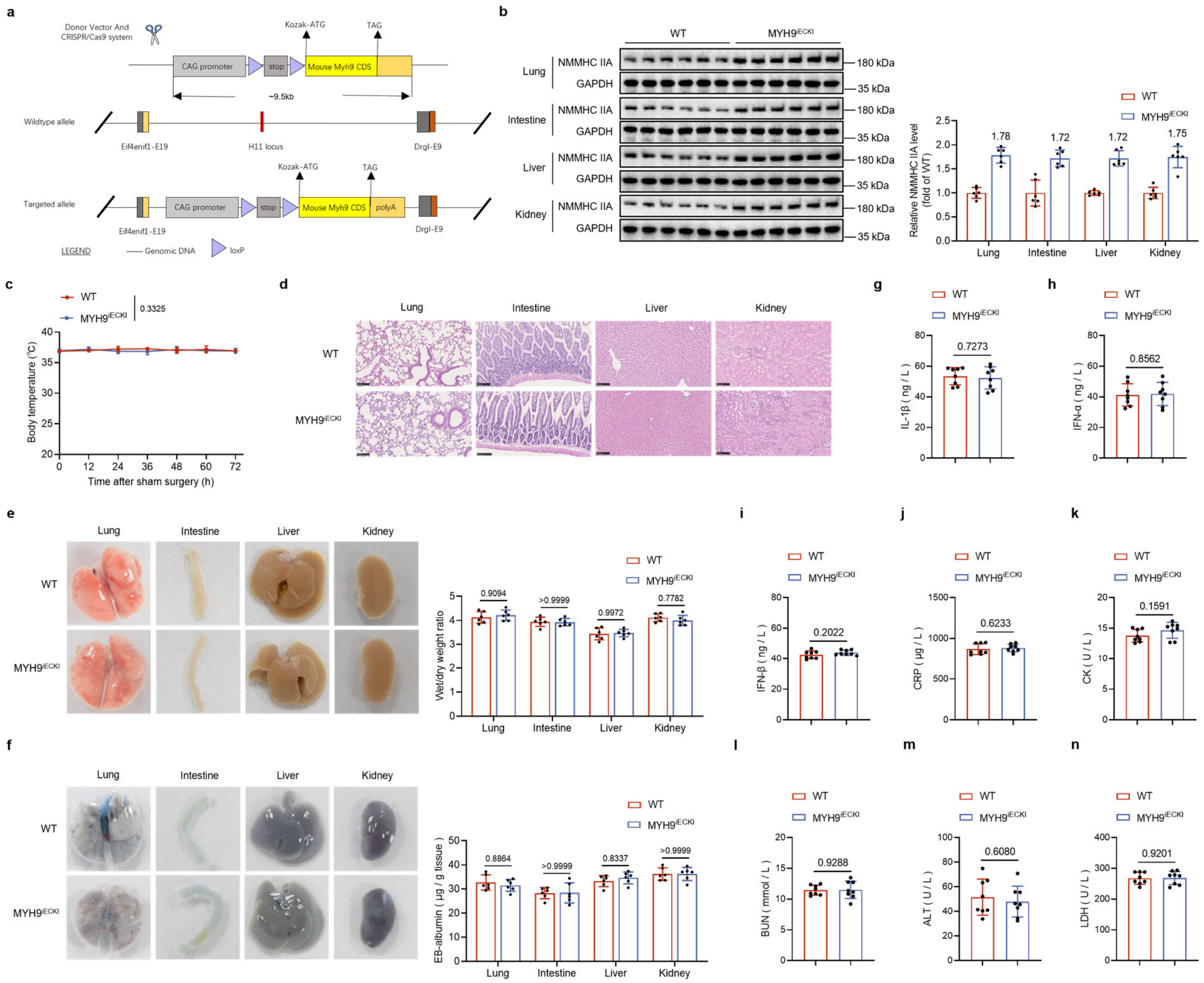

Supplementary Figure 11. **Endothelial-specific NMMHC IIA overexpression does not affect basal physiological homeostasis in mice.** Wild-type (WT) and *MYH9*^{iECKI} mice were treated with sham surgery. **a** Schematic illustrating generation of *MYH9*^{iECKI} mice. **b** Immunoblot analysis and quantification of NMMHC IIA in primary endothelial cells (ECs) isolated from lung, intestine, liver, and kidney of WT and *MYH9*^{iECKI} mice (n = 6 independent mice). **c** Body temperature (n = 6 independent mice). **d** Hematoxylin and eosin (H&E) staining and injury score of lung, intestine, liver, and kidney tissues (n = 6 independent mice). Scale bar = 100 µm. **e** Representative gross morphology and wet/dry ratio of lung, intestine, liver, and kidney tissue (n = 6 independent mice). **f** Evaluation of vascular leakages using Evans blue tracer. Leakage degree was quantified by detecting the Evans blue dye contents in lung, intestine, liver, and kidney homogenate (n = 6

independent mice). **g–i** Quantitation of IL-1β (**g**), IFN-α (**h**), and IFN-β (**i**) in plasma (n = 8 independent mice). **j–n** Quantitation of CRP (**j**), CK (**k**), BUN (**l**), ALT (**m**), and LDH (**n**) in plasma (n = 8 independent mice). All data were represented as the mean ± standard deviation. Statistical analysis was performed using two-tailed Student's t-test (**c–n**). Exact *P* values are indicated in the figure. Source data are provided as a Source Data file.

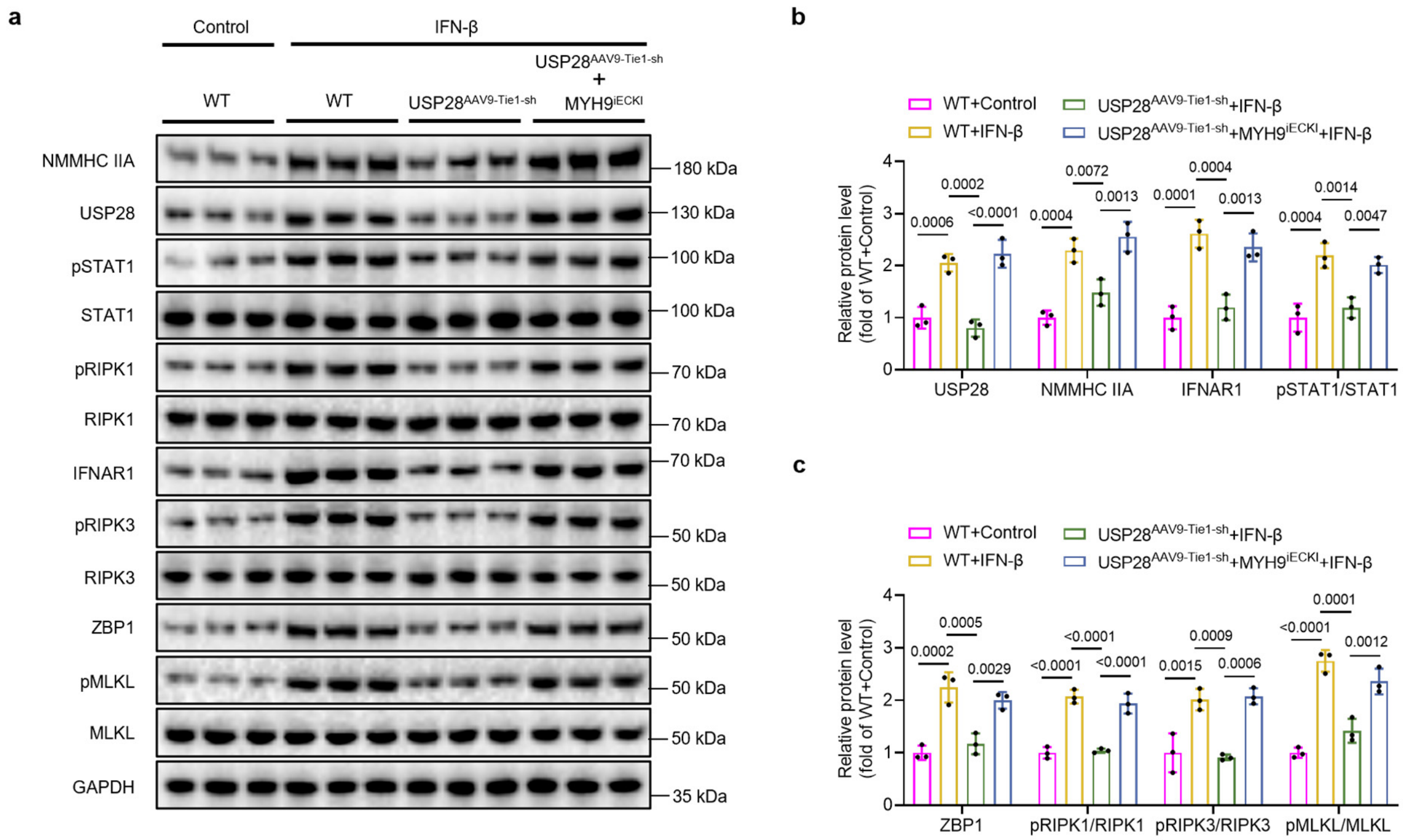

Supplementary Figure 12. Endothelial-specific NMMHC IIA overexpression largely abrogates the protective effect of USP28 deficiency on endothelial necroptosis in MLECs. Primary mouse lung endothelial cells (MLECs) isolated from wild-type (WT), *USP28*$^{\text{AAV9-Tie1-sh}}$ and *USP28*$^{\text{AAV9-Tie1-sh}}$ + *MYH9*^{iECKI} mice were treated with PBS or IFN-β for 12 h. **a–c** Immunoblot analysis and quantification of USP28, NMMHC IIA, IFNAR1, pSTAT1/STAT1, ZBP1, pRIPK1/RIPK1, pRIPK3/RIPK3, and pMLKL/MLKL in MLECs (n = 3 independent cultures). All data were represented as the mean ± standard deviation. Statistical analysis was performed using two-way ANOVA with Tukey post hoc test (**b, c**). Exact *P* values are indicated in the figure. Source data are provided as a Source Data file.

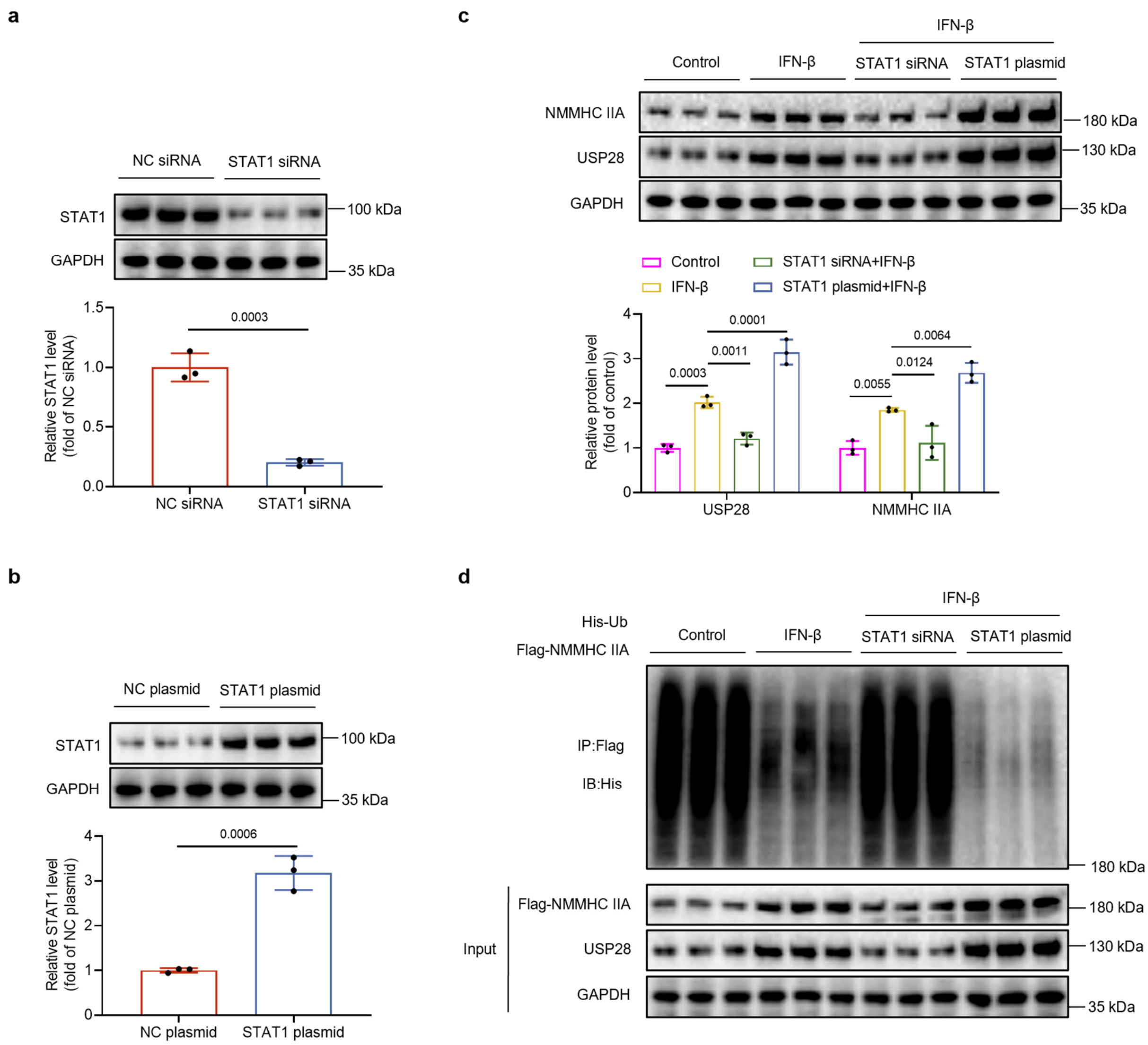

Supplementary Figure 13. **STAT1 promotes USP28-mediated deubiquitination of NMMHC IIA in HUVECs.** Human umbilical vein endothelial cells (HUVECs) were pretreated with *STAT1* siRNA or plasmid, and further stimulated with PBS or IFN-β for 12 h. **a, b** Immunoblot analysis and quantification of STAT1 in HUVECs (n = 3 independent cultures). **c** Immunoblot analysis and quantification of USP28 and NMMHC IIA in HUVECs (n = 3 independent cultures). **d** Ubiquitination levels of NMMHC IIA were assessed in HUVECs (n = 3 independent cultures). All data were represented as the mean ± standard deviation. Statistical analysis was performed using

two-tailed Student's t-test (**a, b**) and two-way ANOVA with Tukey post hoc test (**c**). Exact *P* values are indicated in the figure. Source data are provided as a Source Data file.

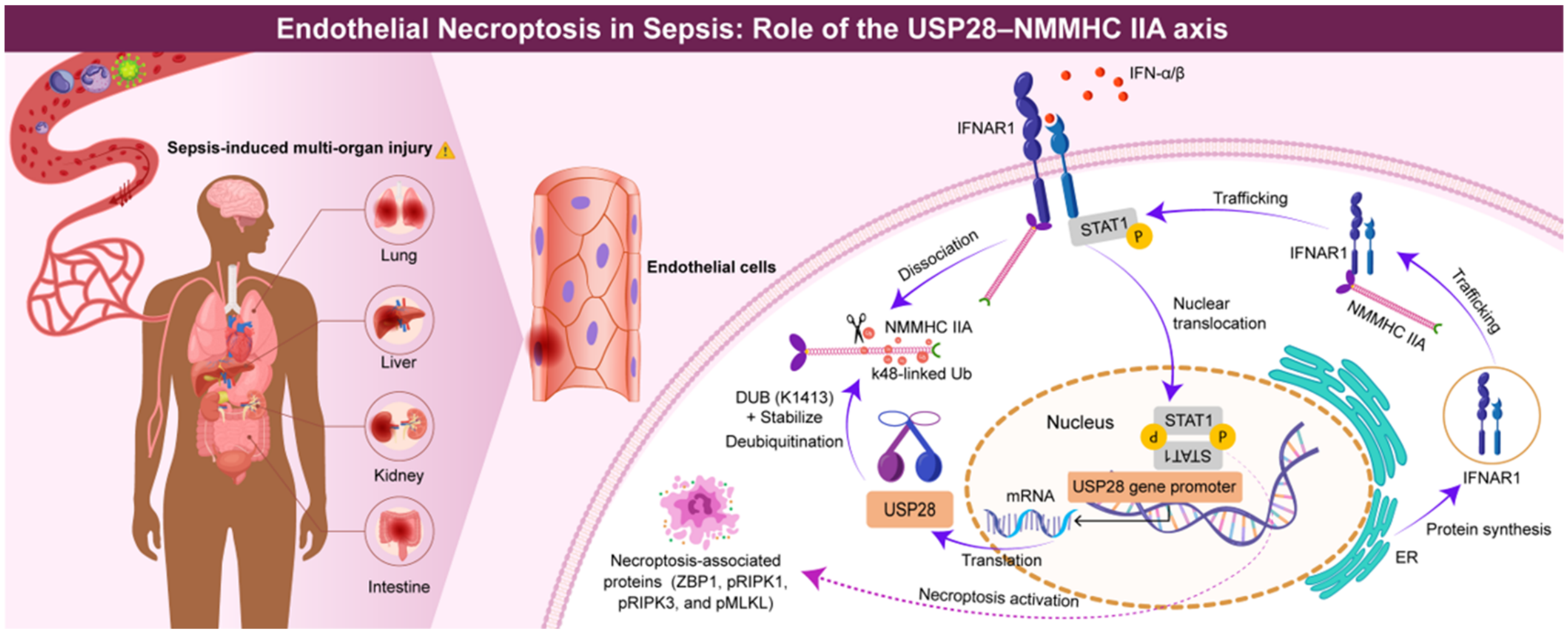

Supplementary Figure 14. **Schematic summary of the mechanism by which NMMHC IIA–USP28 axis exacerbates sepsis.** In sepsis, excessive and sustained type I interferon activates IFNAR1/STAT1 signaling in endothelial cells. Activated STAT1 translocates into the nucleus and promotes USP28 transcription, leading to increased USP28 expression. Mechanistically, USP28 directly binds to NMMHC IIA and removes K48-linked ubiquitination from NMMHC IIA at the K1413 site through its catalytic ubiquitin-specific protease domain, thereby preventing proteasomal degradation and stabilizing NMMHC IIA. Stabilized NMMHC IIA further interacts with IFNAR1 and facilitates IFNAR1 trafficking and accumulation at the plasma membrane, which enhances IFNAR1/STAT1 signaling. Thus, STAT1-dependent USP28 upregulation and USP28–NMMHC IIA-mediated amplification of IFNAR1/STAT1 signaling form a positive feedback loop. Sustained activation of this loop promotes ZBP1/RIPK1/RIPK3/MLKL-dependent endothelial necroptosis, impairs endothelial barrier integrity, increases vascular leakage, and ultimately aggravates sepsis-induced multi-organ injury.

Supplementary Table 1. Demographic information of donors

Characteristic	Sepsis patients (n = 16)	Healthy controls (n = 16)
Age (years, mean ± SD)	63.9 ± 14.8	55.8 ± 11.5
Male (n)	10	6
Female (n)	6	10
Lung infection (n)	3	N/A
Urinary system infection (n)	3	N/A
Peritoneal cavity infection (n)	8	N/A
Soft tissue infection (n)	1	N/A
Other infection site (n)	1	N/A
Mechanical ventilation (n)	15	N/A
SOFA score (median, interquartile range)	8.0 (7.0–10.3)	N/A
Survivors (n)	13	N/A
Non-survivors (n)	3	N/A

Supplementary Table 2. Candidate ubiquitination sites in the C-terminal domain of NMMHC IIA

Site	NMMHC IIA (human)	HTP (high throughput papers)
K821-ub	RNCAAYL**K**LRNWQWW	7
K833-ub	QWWRLFT**K**VKPLLQV	4
K835-ub	WRLFTKV**K**PLLQVSR	6
K850-ub	QEEEMMA**K**EEELVKV	3
K860-ub	ELVKVRE**K**QLAAENR	28
K1234-ub	GELANEV**K**VLLQGKG	3
K1249-ub	DSEHKRK**K**VEAQLQE	3
K1274-ub	VRTELAD**K**VTKLQVE	7
K1277-ub	ELADKVT**K**LQVELDN	5
K1301-ub	SKSSKLT**K**DFSALES	5
K1392-ub	EVKRKLQ**K**DLEGLSQ	4
K1413-ub	AAYDKLE**K**TKTRLQQ	9
K1566-ub	EVNLQAM**K**AQFERDL	3
