# Supplementary material for: USP28 potentiates endothelial necroptosis via deubiquitinating NMMHC IIA in septic male mice
Source: Nat Commun. 2026 Oct 15;17:11667. doi: 10.1038/s41467-026-78666-8 (PMC13759511; doi:10.1038/s41467-026-78666-8)
Supplement: Supplementary file 2 [file 41467_2026_78666_MOESM2_ESM.pdf]

nature portfolio

Corresponding author(s): Junping Kou, Songqiao Liu, Yuanyuan Zhang

Last updated by author(s): Sep 29, 2026

Reporting Summary

Nature Portfolio wishes to improve the reproducibility of the work that we publish. This form provides structure for consistency and transparency in reporting. For further information on Nature Portfolio policies, see our Editorial Policies and the Editorial Policy Checklist.

Statistics

For all statistical analyses, confirm that the following items are present in the figure legend, table legend, main text, or Methods section.

n/a	Confirmed	
☐	☒	The exact sample size (n) for each experimental group/condition, given as a discrete number and unit of measurement
☐	☒	A statement on whether measurements were taken from distinct samples or whether the same sample was measured repeatedly
☐	☒	The statistical test(s) used AND whether they are one- or two-sided<br>*Only common tests should be described solely by name; describe more complex techniques in the Methods section.*
☒	☐	A description of all covariates tested
☐	☒	A description of any assumptions or corrections, such as tests of normality and adjustment for multiple comparisons
☐	☒	A full description of the statistical parameters including central tendency (e.g. means) or other basic estimates (e.g. regression coefficient) AND variation (e.g. standard deviation) or associated estimates of uncertainty (e.g. confidence intervals)
☐	☒	For null hypothesis testing, the test statistic (e.g. F, t, r) with confidence intervals, effect sizes, degrees of freedom and P value noted<br>*Give P values as exact values whenever suitable.*
☒	☐	For Bayesian analysis, information on the choice of priors and Markov chain Monte Carlo settings
☒	☐	For hierarchical and complex designs, identification of the appropriate level for tests and full reporting of outcomes
☐	☒	Estimates of effect sizes (e.g. Cohen's d, Pearson's r), indicating how they were calculated

Our web collection on statistics for biologists contains articles on many of the points above.

Software and code

Policy information about availability of computer code

Data collection	Raw scRNA-seq data: DNBSEQ-T7 system (MGI Tech Co., Ltd.), Cell Ranger (version 8.0.1)<br>Raw mass spectrometry data: MaxQuant software (version 2.1.3.0)<br>HE and Immunohistochemistry images: NanoZoomer 2.0RS (Hamamatsu)<br>Fluorescent images: Zeiss LSM800<br>Western blot images: Bio-Rad Universal Hood II Gel Documentation and Imaging System<br>Fluorescence-activated cell sorting: BD FACSAria Fusion cell sorter (BD biosciences) with BD FACSDiva software (version 8.0)
Data analysis	Statistical analysis: GraphPad Prism (version 8.0)<br>Flow-cytometric data: BD FACSDiva software (version 8.0), FlowJo software (version 10.8.1)<br>Western blot images: Image Lab software (version 6.1)<br>HE and Immunohistochemistry images: NDP.view2 (version 2.9.22), Fiji ImageJ (version 2.14.0)<br>Fluorescent images: Fiji Image J (version 2.14.0)<br>scRNA-seq and nanoproteomics data: Cell Ranger (version 8.0.1), DoubletFinder (v2.0.4), Seurat (v4.4.0), MaxQuant (version 2.1.3.0)

For manuscripts utilizing custom algorithms or software that are central to the research but not yet described in published literature, software must be made available to editors and reviewers. We strongly encourage code deposition in a community repository (e.g. GitHub). See the Nature Portfolio guidelines for submitting code & software for further information.

Data

Policy information about availability of data

All manuscripts must include a data availability statement. This statement should provide the following information, where applicable:

- Accession codes, unique identifiers, or web links for publicly available datasets
- A description of any restrictions on data availability
- For clinical datasets or third party data, please ensure that the statement adheres to our policy

The single-cell RNA-sequencing raw data generated in this study have been deposited in the NCBI Sequence Read Archive (SRA) under BioProject accession number PRJNA1379272. The raw nanoproteomics mass spectrometry data have been deposited in the iProX repository (a ProteomeXchange partner repository) under accession code PXD071841. The raw IP-MS data have also been deposited in the iProX repository under accession code PXD071842. Source data are provided with this paper. All other data supporting the findings of this study are available from the corresponding authors upon request, without restrictions.

Research involving human participants, their data, or biological material

Policy information about studies with human participants or human data. See also policy information about sex, gender (identity/presentation), and sexual orientation and race, ethnicity and racism.

Reporting on sex and gender	Participant sex was recorded from medical records as male or female. Sex was not used as an inclusion or exclusion criterion, and both male and female participants were enrolled. The trial recruited female and male participants without any prejudice. Specifically, among the patients with sepsis (age, 63.9 ± 14.8 years), ten were male and six were female. Among the non-sepsis donors (age, 55.8 ± 11.5 years), there were six males and ten females. Sex-specific subgroup analyses were not performed because the relatively small cohort size did not provide sufficient statistical power for meaningful sex-stratified analyses.
Reporting on race, ethnicity, or other socially relevant groupings	There was no bias of recruitment in terms of race, ethnicity, or other socially relevant.
Population characteristics	Population characteristics were indicated in Supplementary Table 1.
Recruitment	Septic and non-septic participants were recruited sequentially from the Critical Care Medicine of Zhongda Hospital, Affiliated to Southeast University. Study candidates were identified by treating physicians at Critical Care Medicine of Zhongda Hospital, Affiliated to Southeast University. Written informed consent was obtained directly from participants who were able to provide consent. For participants who were unable to provide informed consent at the time of enrollment because they were unconscious or under medically required sedation, including sedation administered during invasive mechanical ventilation, written informed consent was obtained from their legally authorized representatives prior to enrollment. No participant compensation was provided.
Ethics oversight	This project was reviewed and proved by the Independent Ethics Committee for Clinical Research of Zhongda Hospital, Affiliated to Southeast University (2023ZDSYLL468-P01).

Note that full information on the approval of the study protocol must also be provided in the manuscript.

Field-specific reporting

Please select the one below that is the best fit for your research. If you are not sure, read the appropriate sections before making your selection.

☒ Life sciences ☐ Behavioural & social sciences ☐ Ecological, evolutionary & environmental sciences

For a reference copy of the document with all sections, see nature.com/documents/nr-reporting-summary-flat.pdf

Life sciences study design

All studies must disclose on these points even when the disclosure is negative.

Sample size	No sample-size calculations were performed. Sample size was determined to be adequate based on the magnitude and consistency of measurable differences between groups. For in vitro and in vivo experiments, the minimum of sample size was 3 in all the cases. Exact sample sizes for all experimental groups are provided in the corresponding figure legends and Source Data.
Data exclusions	Inclusion and exclusion criteria were specified in the protocol and followed. No data were excluded from the analysis.
Replication	All attempts at replication were successful. Data were representative of at least three independent experiments.
Randomization	Patient samples were included based on predefined diagnostic criteria, and no randomization was applied. For animal experiments, mice were allocated to experimental groups based on genotype; therefore, no formal randomization was performed. However, mice were randomly housed in cages throughout the experiment. For in vitro studies, samples were randomly assigned to experimental groups.

Blinding	Investigators were not blinded to mouse genotypes or cell line conditions during the experiments, as these were required for experimental design and execution. Blinding was not applied during data collection and analysis. However, histological analyses and image quantification were performed in a blinded manner.

Reporting for specific materials, systems and methods

We require information from authors about some types of materials, experimental systems and methods used in many studies. Here, indicate whether each material, system or method listed is relevant to your study. If you are not sure if a list item applies to your research, read the appropriate section before selecting a response.

Materials & experimental systems

n/a	Involved in the study
☐	☒ Antibodies
☐	☒ Eukaryotic cell lines
☒	☐ Palaeontology and archaeology
☐	☒ Animals and other organisms
☒	☐ Clinical data
☒	☐ Dual use research of concern
☒	☐ Plants

Methods

n/a	Involved in the study
☒	☐ ChIP-seq
☐	☒ Flow cytometry
☒	☐ MRI-based neuroimaging

Antibodies

Antibodies used	All the used antibodies for CECs and isolation were summarized as following: anti-CD45-APC (10 μl/10^6 cells; Proteintech, Cat#APC-65064), anti-CD34-FITC (20 μl/10^6 cells; Proteintech, Cat#FITC-65183) and anti-CD146-PE (10 μl/10^6 cells; Proteintech, Cat#PE-65181).<br>All the used antibodies for immunofluorescence were summarized as following: NMMHC IIA (1:200; Proteintech, Cat#11128-1-AP), USP28 (1:200; Proteintech, Cat#17707-1-AP), IFNAR1 (1:100; Proteintech, Cat#13083-1-AP), pSTAT1 (1:100; Affinity Biosciences, Cat#AF3300), ZBP1 (1:100; ABclonal, Cat#A28338), pRIPK1 (1:100; Thermo Fisher Scientific, Cat#PA5-104645), pRIPK3 (1:200; Cell Signaling Technology, Cat#91702), pMLKL (1:1600; Cell Signaling Technology, Cat#37333), CD31 (1:200; R&D Systems, Cat#AF3628), Ki67 (1:2000; Proteintech, Cat#28074-1-AP).<br>The antibodies used for western blotting are as followed: NMMHC IIA (1:8000; Proteintech, Cat#11128-1-AP), USP28 (1:8000; Proteintech, Cat#17707-1-AP), IFNAR1 (1:1000; Proteintech, Cat#13083-1-AP), pSTAT1 (1:1000; Affinity Biosciences, Cat#AF3300), ZBP1 (1:5000; ABclonal, Cat#A28338), RIPK1 (1:1000; Abcam, Cat#ab300617), pRIPK1 (1:1000; Thermo Fisher Scientific, Cat#PA5-104645), RIPK3 (1:2000; Thermo Fisher Scientific, Cat#703750), pRIPK3 (1:1000; Cell Signaling Technology, Cat#91702), MLKL (1:10000; Proteintech, Cat#66675-1-Ig), pMLKL (1:1000; Cell Signaling Technology, Cat#37333), GSDMD-N/GSDMD (1:5000; ABclonal, Cat#A20197SP), Cleaved Caspase 3 (1:10000; Proteintech, Cat#82707-13-RR), Caspase 3 (1:2000; Proteintech, Cat#66470-2-Ig), ubiquitin (1:5000; Proteintech, Cat#10201-2-AP), His tag (1:10000; Proteintech, Cat# 66005-1-Ig), HA tag (1:10000; Proteintech, Cat#51064-2-AP), Flag tag (1:20000; Proteintech, Cat#20543-1-AP), GFP tag (1:2000; Proteintech, Cat#50430-2-AP), Myc tag (1:10000; Proteintech, Cat# 60003-2-Ig), GAPDH (1:8000; Bioworld, Cat#MB001), β-actin (1:50000; Proteintech, Cat#66009-1-Ig), ATP1A1 (1:10000; Proteintech, Cat#14418-1-AP).<br>The antibody used for immunohistochemistry is as followed: USP28 (1:200; Thermo Fisher Scientific, Cat#PA5-52347)
Validation	All antibodies are commercially available and have been verified by the manufacturers according to the immunoblots and/or images on their websites.

Eukaryotic cell lines

Policy information about cell lines and Sex and Gender in Research

Cell line source(s)	HUVECs (Cat#PSC-01; female donor) and HEK293T cells (Cat#PSC-01GNHu17; female) were obtained from the Shanghai Institute of Cell Biology, Chinese Academy of Sciences.
Authentication	Cell lines were obtained from the Shanghai Institute of Cell Biology, Chinese Academy of Sciences, where they were authenticated by short tandem repeat (STR) analysis. No additional authentication was performed in this study.
Mycoplasma contamination	All the cell lines were tested negative for mycoplasma contamination.
Commonly misidentified lines (See ICLAC register)	No misidentified cell lines were used.

Animals and other research organisms

Policy information about studies involving animals; ARRIVE guidelines recommended for reporting animal research, and Sex and Gender in Research

Laboratory animals	All mouse lines were Mus musculus on the C57BL/6J genetic background. Male mice aged 8–10 weeks were used for all animal

Laboratory animals	experiments unless otherwise indicated. The number of animals used in each individual experiment is provided in the corresponding figure legends and Source Data. Endothelial-specific NMMHC IIA knockin (MYH9iECKI) mice were obtained from Nanjing Biomedical Research Institute of Nanjing University (Certificate number was SCXK Jiangsu 2023–0009). USP28 endothelial-specific knockout (USP28ECKO) mice were obtained from Cyagen Bioscience (Certificate SCXK Jiangsu 2020-0006). C57BL/6J male mice were obtained from Jiangsu Huachuang Xinnuo Pharmaceutical Technology (Certificate SCXK 2020–0009). All mice were housed and maintained in specific pathogen-free conditions with a 12:12 light/dark schedule, controlled ambient temperature, and humidity, and were given free access to food and water.
Wild animals	No wild animals were used.
Reporting on sex	All animal experiments in this study were conducted using male mice exclusively to eliminate potential confounding effects of sex hormones on experimental outcomes, consistent with standard practices in this field of research.
Field-collected samples	The study did not involve samples collected from the field.
Ethics oversight	All animal experiments followed the protocols approved by the Animal Ethics Committee of China Pharmaceutical University (Certificate No. 2024-12-031).

Note that full information on the approval of the study protocol must also be provided in the manuscript.

Plants

Seed stocks	The study did not involve in Plants.
Novel plant genotypes	The study did not involve in Plants.
Authentication	The study did not involve in Plants.

Flow Cytometry

Plots

Confirm that:

☒ The axis labels state the marker and fluorochrome used (e.g. CD4-FITC).

☒ The axis scales are clearly visible. Include numbers along axes only for bottom left plot of group (a 'group' is an analysis of identical markers).

☒ All plots are contour plots with outliers or pseudocolor plots.

☒ A numerical value for number of cells or percentage (with statistics) is provided.

Methodology

Sample preparation	The blood drawn from the subjects was mixed with ficoll solution and centrifuged at 500×g at 20 ? for 25 min. After centrifugation, the PBMCs were carefully aspirated and transferred to new centrifuge tubes, and the plasma was preserved for subsequent research. PBMCs were incubated with Fc receptor blockers (1 µg/106 cells, BD Biosciences) after washing by PBS. Subsequently, PBMCs were stained in the dark by Hoechest 33342 (1:100, Beyotime, Cat#C1029), 7-AAD (1:1000, Beyotime, Cat#ST515), anti-CD45-APC (10 µl/106 cells, Proteintech, Cat#APC-65064), anti-CD34-FITC (20 µl/106 cells, Proteintech, Cat#FITC-65183) and anti-CD146-PE (10 µl/106 cells, Proteintech, Cat#PE-65181).
Instrument	BD FACSAria Fusion cell sorter (BD biosciences)
Software	BD FACSDiva software (version 8.0), FlowJo software (version 10.8.1)
Cell population abundance	Circulating endothelial cells (CECs) were defined as Hoechst 33342^{+}/7-AAD^{-}/CD45^{-}/CD34^{+}/CD146^{+} cells following flow cytometric sorting, accounting for approximately 0.041 % of total events. Due to the extremely low abundance of CECs as a rare cell population, post-sort purity was not further assessed.
Gating strategy	Hoechst positivity excludes non-nucleated events such as platelets and debris, 7-AAD negativity excludes non-viable cells, and CD45 negativity excludes hematopoietic cells. In addition, FSC/SSC-based singlet discrimination was applied prior to marker-based selection to reduce doublets and platelet–leukocyte aggregates.

☒ Tick this box to confirm that a figure exemplifying the gating strategy is provided in the Supplementary Information.
