# Supplementary material for: USP28 potentiates endothelial necroptosis via deubiquitinating NMMHC IIA in septic male mice
Source: Nat Commun. 2026 Oct 15;17:11667. doi: 10.1038/s41467-026-78666-8 (PMC13759511; doi:10.1038/s41467-026-78666-8)
Supplement: Supplementary file 3 [file 41467_2026_78666_MOESM3_ESM.pdf]

nature portfolio

Peer Review File

USP28 potentiates endothelial necroptosis via deubiquitinating NMMHC IIA in septic male mice

Corresponding Author: Professor Junping Kou

This file contains all reviewer reports in order by version, followed by all author rebuttals in order by version.

Version 0:

Reviewer comments:

Reviewer #1

(Remarks to the Author)
In this manuscript, Tang et al. investigates the role of the deubiquitinase USP28 in regulating endothelial cell necroptosis during sepsis. Specifically, the authors isolate circulating endothelial cells (CECs) from sepsis patients and healthy donors to perform comparative proteomic profiling, with the goal of identifying key molecular drivers of sepsis-induced endothelial dysfunction. Through this approach, they identify USP28 as significantly upregulated in septic patients (especially non-survivors). They then use a combination of cell culture and animal models to demonstrate that elevated endothelial USP28 promotes necroptotic cell death in sepsis, ostensibly via a novel signaling axis involving the substrate NMMHC IIA and downstream interferon (IFN) pathway activation.

A notable strength of the study is its use of advanced proteomics methodologies to tackle a clinically relevant problem. The authors employ “nanoproteomics” for the single-cell proteomics analysis of rare CECs, enabling detection of over 1,000 proteins from limited samples. Using nanoproteomics to profile CECs, they demonstrate an interesting correlation between endothelial USP28 expression and sepsis-induced multi-organ injury. In addition, the authors perform additional immunoprecipitation-coupled mass spectrometry (IP-MS) to uncover proteins interacting with USP28. The combination of unbiased proteomics analysis for discovery with targeted validation is a powerful aspect of the work. That said, the proteomics techniques used here are inherently complex, and certain technical details (e.g. data analysis pipelines) are only briefly described. A more thorough discussion of these methodological aspects would strengthen the manuscript by helping readers appreciate the robustness and limitations of the data.

Overall, the study’s findings appear novel and significant. Prior to this work, the involvement of deubiquitinating enzymes in endothelial necroptosis during sepsis was not well defined. Tang et al. reveal a new USP28–NMMHC IIA molecular axis that links protein ubiquitination status to necroptotic signaling. Their data suggest that USP28 stabilizes NMMHC IIA by removing K48-linked ubiquitination at Lys1413, preventing its proteasomal degradation. Stabilized NMMHC IIA then enhances IFNAR1/STAT1 pathway signaling, thereby exacerbating inflammatory cell death. This mechanistic insight is supported by both their proteomics data and functional assays, and it points to USP28 as a potential therapeutic target. To this end, the authors showed that endothelial-specific USP28 deficiency (using USP28AAV9-Tie1-sh) improved the survival of septic mice. Moreover, the authors demonstrated that intragastrically administration of USP25/28 inhibitor AZ1immmediatly prior to CLP surgery led to reduced mortality and increased body temperature of cecal ligation and puncture (CLP)-treated mice, suggesting that pharmacological inhibition of USP28 can be protective in sepsis models.

Ultimately, this manuscript tackles an important question with innovative proteomic approaches and provides compelling evidence for a novel mechanism in sepsis pathology. The authors should be commended for bringing together cutting-edge proteomics and mechanistic biology in a challenging clinical context. With appropriate revisions it has the potential to make a valuable contribution to the scientific community. Therefore, I recommend the manuscript be considered for publication only after addressing my concerns raised above (vide supra) and the following specific concerns presented below:

1. While the manuscript is generally well written, the authors do not fully leverage their Figures as major tools for effective scientific communication. Several Figures illustrate the raw data well but do not see to effectively convey their key conclusions clearly. In particular, the results shown in Figures 1B-D, 3C, 5B, 6B, and 8H are crowded, and the text and data are difficult to interpret. Given the complexity of the relationships among USP28, NMMHC IIA, IFNAR1/STAT1 signaling, IFN-I, and sepsis, the authors are strongly encouraged to include a schematic model summarizing these interactions as a

part of the discussions (perhaps even as a new Figure).

2. The proteomics approach is exciting and clearly a key strength of the study. However, to help readers fully understand these results, the manuscript would benefit from additional methodological detail and clarity. For example, it should be clarified how the data analysis for all the proteomics results were actually performed: software, settings, tolerances for MS1/MS2, etc.. For instance, in the IP-MS results, MaxQuant is mentioned for “identification and quantification” without any mention of how the analysis was performed. For the nanoproteomics results, it appears no mentions were made to how the data was eventually processed. Providing this information will bolster confidence that the proteomic findings are robust and reproducible. I encourage the authors to also supply the complete list of their search results showing the peptides/proteins identified and their differential expression statistics, so that readers can appreciate the full dataset underlying the focus on USP28.

3. Line 52, Should the abbreviation of “pathogen-associated molecular patterns” be “PAMPs” instead of the used “DAMPs”? If so, the authors should carefully address this wording throughout the manuscript.

4. Line 327, the concluding statement “USP28 inhibits sepsis progression by inhibiting ubiquitination …” seems to contradict all the authors results. Did the authors mean to phrase this differently?

5. In this manuscript the authors introduce the term “nanoproteomics” as an “emerging sequencing technology”, but this term has been used in prior literature to describe interdisciplinary proteomics technologies that enable the identification and characterization of low-abundance proteoforms (see PMCID: PMC7411019; PMC6656398; PMC10728164). I recommend the authors briefly clarify this point in the manuscript and cite the relevant prior studies, thereby acknowledging the existing work in this emerging area.

6. The manuscript is focused on endothelial USP28, have the authors consider whether immune-cell USP28 indirectly contributes to cytokine production or IFN-I signaling, which can affect the in vivo data?

Reviewer #2

(Remarks to the Author)
This manuscript reports a substantial and technically sophisticated body of work, but several issues in design, interpretation, and presentation justify a recommendation of major revision before it can be considered further.

1. Novelty and conceptual advance
The central USP28–NMMHC IIA–IFNAR1–STAT1–necroptosis axis is interesting, but the authors should more clearly delineate what is conceptually new compared with prior work on NMMHC IIA in endothelial integrity and IFN-I–induced necroptosis (e.g., ZBP1–MLKL axis and NMMHC IIA in LPS-induced lung injury) and explicitly discuss overlapping pathways rather than largely restating background.

The claim that the USP28–NMMHC IIA axis “promotes sepsis pathology” and represents a “promising therapeutic target” feels somewhat overstated relative to the modest sample size of human data and the constraints of a single CLP model; these translational claims should be toned down or more rigorously qualified.

2. Human cohort design and characterization
The clinical cohort of sepsis patients used for CEC nanoproteomics appears relatively small, and the criteria for inclusion, exclusion, timing of sampling, infection source, and organ support (vasopressors, ventilation, RRT) are not fully described; more detailed clinical characterization and a table comparing survivors vs non-survivors are needed to interpret the correlation with SOFA and IFN-I levels.

It is unclear whether potential confounders (age, comorbidities, immunosuppression, steroid or IFN therapy, baseline endothelial disease) were controlled in the analysis linking CEC USP28/NMMHC IIA expression to outcomes; multivariable or at least stratified analyses should be presented or limitations clearly acknowledged.

3. Definition and validation of CECs
The gating strategy (Hoechst$^+$7-AAD$^-$CD45$^-$CD34$^+$CD146$^+$) is plausible but the manuscript does not show rigorous validation that these events are bona fide mature CECs rather than progenitors or platelet–leukocyte aggregates; additional phenotypic markers, imaging cytometry, or parallel endothelial-specific transcripts/proteins would strengthen this aspect.

Because nanoproteomics is performed on extremely rare cells, the authors need to discuss and, where possible, quantify purity, doublet rates, and potential leukocyte contamination, and how these may bias the identification of USP28 as a top hit.

4. Nanoproteomics analysis and statistics
The proteomic pipeline (single-cell or nano-scale) is only briefly sketched; details about normalization, batch correction, missing-value imputation, statistical models, and multiple-testing correction (e.g., FDR thresholds) for DEGs and KEGG analyses should be provided, preferably in Methods and figure legends.

The intersection strategy that narrows 173 DEGs to four recurring genes and then prioritizes USP28 as “the key regulator” seems somewhat arbitrary; the rationale for focusing on USP28 over other candidates (TMED9, TPM3, PPGB) and whether

they were systematically evaluated should be more transparently described.

5. Mouse models and sepsis relevance
All in vivo work appears restricted to young male C57BL/6J mice; given the clinical relevance of age and sex in sepsis, the authors should at least acknowledge this more explicitly and, if feasible, provide data or a plan for older and/or female mice.

The CLP protocol (75% ligation, single puncture) yields a very severe phenotype; justification of this severity and discussion of how it compares to human sepsis dynamics (particularly in relation to timing of interventions like AZ-1) would help interpret the therapeutic implications.

6. Specificity and timing of USP28 targeting
The timing of AZ-1 administration is essentially prophylactic (immediately prior to CLP), which does not mimic realistic clinical scenarios; the authors should include delayed-treatment experiments (e.g., 6–12 h post-CLP) or temper claims about therapeutic potential and explicitly label AZ-1 as proof-of-concept rather than a clinically viable regimen.

USP28 is expressed in multiple cell types; while endothelial-specific knockout/overexpression is used, AZ-1 will act systemically. The authors should clarify whether AZ-1 effects are EC-specific (e.g., through conditional models ± AZ-1) and discuss possible off-target or non-endothelial contributions.

7. Necroptosis readouts and cell death specificity
Necroptosis is inferred mainly from increased pRIPK1/pRIPK3/pMLKL and ZBP1, but classic genetic or pharmacologic validation (e.g., $RIPK3^{-/-}$, $MLKL^{-/-}$ mice, or necrostatin-1) is lacking; at minimum, more stringent evidence that endothelial death is necroptotic rather than apoptotic/pyroptotic (e.g., cleaved caspase-3, GSDMD, TUNEL) should be provided.

In HUVECs, cell viability and barrier assays under IFN-I are not accompanied by direct necroptosis-specific functional readouts; adding rescue with necroptosis inhibitors or genetic silencing of RIPK3/MLKL would make the mechanistic conclusion more robust.

8. Mechanistic depth of the USP28–NMMHC IIA–IFNAR1 axis
While the mapping of USP28 and NMMHC IIA interaction domains is thorough, the functional necessity of K1413 deubiquitination is primarily inferred from mutant overexpression; the authors should test whether a deubiquitinase-dead USP28 mutant fails to stabilize NMMHC IIA and to promote IFNAR1 trafficking and necroptosis in ECs, ideally in vivo or in primary endothelial cells.

The link between NMMHC IIA and IFNAR1 membrane trafficking remains largely descriptive; mechanistic insight into trafficking pathways (e.g., endosomal markers, live-cell imaging, biotinylation assays) and whether NMMHC IIA affects IFNAR1 internalization vs anterograde transport would substantially strengthen this part of the story.

9. IFN-I signaling and feedback loop
The proposed positive feedback loop (IFN-I → STAT1 → USP28 transcription → NMMHC IIA stabilization → IFNAR1 trafficking → amplified IFN-I signaling) is appealing but only partially supported; for example, the dependence of IFNAR1 surface levels and downstream STAT1 activation on USP28/NMMHC IIA in vivo is not rigorously quantified (e.g., phospho-STAT1 and ISG expression in sorted ECs).

Given the complex and sometimes protective roles of IFN-I in sepsis and ARDS, the discussion should better integrate conflicting literature and explicitly clarify that the data primarily address excessive, sustained IFN-I in a specific CLP context rather than all sepsis settings.

10. Overexpression rescue models and physiological relevance
The EC-specific overexpression of IFNAR1 or NMMHC IIA via AAV9 may produce supra-physiological levels that do not reflect human sepsis; the authors should show absolute or relative expression levels compared to endogenous in septic ECs and discuss whether the phenotypes might partly reflect non-physiologic receptor/myosin load.

In both IFNAR1AAV9-Tie1 and MYH9iECKI rescue experiments, overexpression/knockin appears to fully abrogate the protective effects of USP28 deficiency; the possibility of ceiling effects or pathway saturation should be addressed, and partial-rescue phenotypes or dose–response experiments would be informative.

11. Single-cell RNA-seq analysis of lung ECs
The scRNA-seq lung dataset is a major mechanistic anchor, but analysis of endothelial subclusters (arterial, venous, capillary, lymphatic) and their differential response to USP28 deletion is limited; more granular analysis of EC heterogeneity and necroptosis/IFN-I signatures across subtypes would strengthen the mechanistic claims.

Methods for quality control, doublet removal, integration, and differential expression in scRNA-seq (e.g., thresholds for mitochondrial reads, minimum genes, batch correction) are not fully detailed and should be expanded, with appropriate statistics and multiple-testing correction clearly stated.

12. Data presentation and quantification
Several key figures rely on representative histology or immunofluorescence with limited quantitative data in the main text; numerical data (e.g., necroptosis markers in lung ECs, vascular leakage, wet/dry ratios) should be fully plotted with sample

sizes, effect sizes, and P values clearly indicated, ideally in the main figures or expanded data.

For many western blots and co-IP experiments, quantification (densitometry) is not consistently shown, and the number of biological replicates is sometimes unclear; the authors should standardize reporting of n, independent experiments, and statistical tests throughout.

13. Pharmacological tool AZ-1
The selectivity profile of AZ-1 for USP28 versus other USPs and deubiquitinases is not discussed; the authors should either provide or cite biochemical specificity data and acknowledge potential off-target effects that might contribute to the observed phenotypes.

If feasible, using an independent USP28-targeting modality (e.g., a structurally distinct inhibitor or a genetic knockdown approach in adult mice) would help exclude AZ-1–specific artifacts; otherwise, this limitation should be clearly stated.

14. Ethical, reporting, and ARRIVE considerations
The animal experiments section lacks some important details (randomization, allocation concealment, a priori power calculations, humane endpoints) that are increasingly expected for high-impact journals; these details should be added and aligned with current ARRIVE guidelines.

For human subjects, the consent process and handling of severely ill or incapacitated patients (e.g., surrogate consent) should be clarified, and the registration of the cohort or biobank, if any, should be indicated.

15. Writing, consistency, and minor issues
There are numerous typographical and formatting issues (missing hyphens, inconsistent spacing, ambiguous symbols, occasional apparent word omissions) that impede clarity and should be carefully corrected by thorough language editing.

Some statements in the Discussion seem internally inconsistent with earlier sections (e.g., one sentence suggests USP28 “inhibits sepsis progression” while the overall conclusion is that USP28 is detrimental), and the final paragraphs should be revised to maintain logical consistency and avoid contradictory phrasing.

Reviewer #3

(Remarks to the Author)
Thank you for the invitation to review the manuscript. The authors propose a mechanism wherein endothelial USP28 stabilizes NMMHC IIA to promote IFNAR1 trafficking and necroptosis, thereby driving sepsis-induced multi-organ injury. The study presents a substantial amount of work, including clinical correlation, genetic mouse models, and molecular biology assays. However, despite the potential novelty of the proposed USP28-NMMHC IIA axis, I have significant concerns regarding the experimental design, the quality of the data, and the strength of the evidence provided. The current findings are insufficient to support the major conclusions drawn. While the hypothesis is interesting, the study in its current form suffers from fundamental design flaws and a lack of robust evidence. Therefore, I cannot recommend its publication.

Major concerns:
1. Organ Heterogeneity and Sampling Bias (Figure 1)
While the IHC results in Figure 1H suggest ubiquitous expression of USP28 across the lung, intestine, liver, and kidney, the co-localization analysis in Figure 1I is restrictively performed only on pulmonary endothelial cells. Why were other cell types and organs excluded? Given that sepsis-induced dysfunction follows a distinct temporal sequence across different organs, it is scientifically precarious to assume the pulmonary expression pattern represents the systemic condition. Preliminary verification of USP28 in other cell types is required. Furthermore, the immunofluorescence (IF) images provided have an unacceptably small field of view, which introduces significant selection bias. Larger fields of view are necessary to ensure data representativeness.

2. Inadequate Validation of Genetic Models (Figure S1 & S3)
Relying solely on IF (Figure S1B) to validate endothelial-specific knockout or overexpression is methodologically insufficient. The authors must isolate primary pulmonary endothelial cells from the respective mouse models and confirm the efficiency via Western blotting and qPCR. Additionally, the overexpression efficiency shown in Figure S3B is underwhelming (less than 2-fold increase). This raises serious concerns regarding the titer of the AAV virus or the efficacy of the injection protocol, questioning the validity of the subsequent phenotypic observations.

3. Poor Histological Quality (Lung H&E)
The quality of the lung H&E staining images is substandard and requires immediate replacement. Crucially, the control group exhibits evident structural damage and alveolar collapse, which is biologically implausible for healthy tissue. This suggests either improper sample processing or pre-existing pathology in the control mice.

4. Anomalous Cytokine Profiles (Figure 2 & S2)
The cytokine data presented is highly questionable for several reasons:
Timing: Measuring cytokines at 24 hours post-CLP is suboptimal, as levels of IL-1β, IL-6, and TNF-α typically peak at 6–12 hours and decline significantly by 24 hours.
Magnitude Discrepancy: Physiologically, serum IL-6 levels (typically ng/mL range) should exceed IL-1β and TNF-α (pg/mL range) in sepsis, yet the reported data contradicts this well-established pattern.

Baseline Levels: The baseline TNF-α level in control mice is reported as ~500 pg/mL (500 ng/L), which is aberrantly high (normal range <30 pg/mL).
Incomplete Data: Why are cytokine and CK data omitted in subsequent figures? This selective reporting undermines the credibility of the study.

5. Flawed Drug Intervention Strategy
Administering drug intervention prior to CLP surgery violates Minimum Quality Threshold in Pre-Clinical Sepsis Studies (Mqtipss), which require post-insult treatment to mimic clinical reality. Furthermore, AZ-1 is a non-specific inhibitor that also targets USP25, a homolog expressed in endothelial cells (PMID: 40370563). The authors failed to rule out the off-target contribution of USP25 inhibition to the observed protective effects.

6. Limitations of AAV Tropism (Figure S3)
While AAV9-Tie1 effectively targets the lung and liver via tail vein injection, its transduction efficiency in the kidney and intestine is historically poor. The authors did not assess the overexpression efficiency in these organs. Therefore, attributing the systemic multi-organ dysfunction solely to endothelial USP28 overexpression is speculative without verifying target engagement in the kidney and gut.

7. Survival vs. Organ Damage Discrepancy (Figure S3C)
If endothelial USP28 overexpression exacerbates multi-organ damage, why does it fail to accelerate mortality in the survival analysis? This discrepancy suggests a "ceiling effect" caused by an overly severe CLP model. I recommend repeating this experiment using a milder CLP protocol (e.g., fewer punctures or smaller needle size) to determine if USP28 overexpression truly impacts survival.

8. Irrational Vector Control Data (Figure S3)
The "Vector-AAV9-Tie1 + CLP" group exhibits significantly milder tissue injury and lower inflammatory markers compared to the "WT + CLP" group in Figure 2 or the "CLP" group in Figure S2. It is biologically irrational for an empty vector to confer protection against sepsis. This inconsistency indicates serious issues with experimental reproducibility or batch effects.

9. Lack of Biochemical Verification for Signaling (Figures 3, 4, 7, S4, S5)
Relying exclusively on immunofluorescence to demonstrate the impact of USP28 on IFN-I signaling is insufficient. The authors must provide Western blot evidence from isolated mouse pulmonary endothelial cells (in vivo) and HUVEC lysates (in vitro) to quantitatively validate these signaling changes.

10. p-STAT1 Localization and Imaging (HUVECs)
Single-cell imaging is anecdotal and does not represent the population. Moreover, phosphorylated STAT1 (p-STAT1) is a transcription factor that should translocate to the nucleus. The provided images show predominant cytoplasmic localization, which is anomalous. The authors must explain this observation—is it an antibody specificity issue, a fixation artifact, or a biological peculiarity?

11. Validation of AAV-IFNAR1 Rescue
The authors must demonstrate the transduction efficiency of AAV9-Tie1-IFNAR1 across all relevant organs (lung, liver, kidney, intestine). The experimental grouping in Figure 4 is incomplete; a "IFNAR-AAV9-Tie1 + CLP" group should be compared directly with the "USP28 + IFNAR-AAV9-Tie1 + CLP" group. Additionally, the baseline effect of AAV9-Tie1-IFNAR1 on normal (Sham) mice must be assessed.

12. Ubiquitination Specificity
Does USP28 exclusively target K48-linked ubiquitination on NMMHC IIA? The authors should investigate whether it affects other linkage types (e.g., K63) to provide a complete mechanistic picture.

13. Direct Interaction Confirmation (Figures 5 & 6)
Co-IP alone is insufficient to claim "direct" binding. The interaction between USP28 and NMMHC IIA, as well as NMMHC IIA and IFNAR1, must be confirmed using GST-pull down assays (using purified proteins) and Proximity Ligation Assays (PLA) in cells.

14. Validation of AAV-MYH9 Rescue
Similar to Point 11, the authors must validate the efficiency of AAV9-Tie1-MYH9 in all organs. The grouping in Figure 4 is incomplete (missing "MYH9-AAV + CLP" vs. "USP28 + MYH9-AAV + CLP") and lacks a baseline assessment in normal mice.

15. Validation of STAT1 Feedback Loop (Figure 8)
Western blotting is required to confirm the knockdown efficiency of si-STAT1 in Figure 8F. To rigorously prove the feedback loop, the authors should also perform a STAT1 overexpression experiment and measure the resulting changes in USP28 expression and NMMHC IIA ubiquitination levels.

16. Over-interpretation of Lung-Specific Data
The conclusion that USP28 drives septic endothelial necroptosis via IFN-I signaling is predominantly based on scRNA-seq analysis derived exclusively from lung tissue (WT vs. USP28ECKO). However, the authors extrapolate these findings to explain systemic multi-organ dysfunction, a generalization that lacks sufficient logical support. Endothelial heterogeneity is a fundamental biological principle; both under physiological conditions and during sepsis, the molecular mechanisms and

biological behaviors of endothelial cells differ significantly across distinct vascular beds. It is highly probable that the abundance of USP28 and the activation threshold of IFN-I signaling vary among organs, yet the authors only validated this pathway in lung tissue via immunofluorescence, leaving other critical organs unexamined. Furthermore, the use of HUVECs (macrovascular endothelial cells) in vitro fails to adequately represent the specialized phenotype of pulmonary microvascular endothelial cells. Consequently, relying solely on pulmonary and HUVEC data is insufficient to demonstrate the universal applicability of this mechanism in the context of systemic multi-organ injury.

Minor concerns：

1.Selection Criteria for DEGs (Figures 1C & 1E):
The authors appear to have used a relaxed threshold for identifying differentially expressed genes (DEGs). Conventionally, a cutoff of log2(Fold Change) ≥ 1 is recommended to ensure biological significance. While a lower threshold yields a higher number of candidates, it deviates from standard screening criteria and may include noise.

2.Missing Statistical Annotations:
The graphs depicting mouse survival rates and body temperature changes lack statistical significance annotations (e.g., asterisks or p-values). These must be added to allow for proper interpretation of the data.

3.Inconsistency in CRP Levels (Figures 2, S2, & S3):
There is a substantial discrepancy in the average serum CRP levels reported across Figures 2, S2, and S3. The magnitude of these differences is concerning. The authors should verify whether this is due to inconsistencies in the detection assay, batch effects, or variations in the experimental system.

4.Graph Formatting:
The formatting style of the statistical graphs is inconsistent throughout the manuscript. Please standardize the layout, font sizes, and axis styles for a uniform presentation.

5.Alternative Explanations for Cell Numbers (Figure 3D):
Regarding Figure 3D, the observed increase in endothelial cell numbers could theoretically result from self-proliferation or differentiation from progenitor cells. Although investigating these specific pathways may be outside the scope of this study, the authors should briefly discuss these possibilities as potential contributing factors.

6.Contextualizing NMMHC IIA Function:
The functional role of NMMHC IIA in IFN-I signaling is currently mentioned only in the Abstract. To enhance readability and flow, this background information should also be elaborated upon in the Introduction and explicitly reintroduced in the text corresponding to Result sect

Reviewer #4

(Remarks to the Author)
This manuscript presents a comprehensive and mechanistically well-supported study elucidating the role of USP28 in sepsis-induced endothelial necroptosis and multi-organ injury. The study initiated with nanoproteomics from patients to identify USP28 as a key deubiquitinase upregulated in sepsis and demonstrate that endothelial USP28 promotes vascular injury and mortality in a polymicrobial sepsis model. Mechanistically, USP28 stabilizes NMMHC IIA through deubiquitination, thereby sustaining IFNAR1 membrane localization and amplifying IFN-I signaling, which ultimately drives endothelial necroptosis and barrier dysfunction. Functionally, endothelial-specific overexpression of IFNAR1 or NMMHC IIA largely reversed the protective effects of USP28 inhibition on sepsis-induced multi-organ injury. This study provides mechanistic insight into endothelial regulation of IFN-I signaling during polymicrobial sepsis and helps explain how sustained interferon responses contribute to vascular injury in severe infection. As such, these original findings should appeal to a broad audience, including clinicians, infectious diseases researchers, and immunologists. The overall conclusions are well supported by the data, and the study design is elegant. But there some issues should be addressed prior to publication.
1.The study exclusively utilized male mice for all in vivo experiments. The authors are encouraged to briefly justify this experimental choice and to comment on whether sex-specific effects might influence the interpretation of the findings.
2.In the Methods section, the description of the Evans blue–albumin leakage assay includes a wavelength of “20 nm”, which appears inconsistent with typical UV–Vis absorbance measurements. The authors should check and correct this value.
3.In Figure 1H, the immunohistochemical images labeled as liver and kidney appear inconsistent with the expected tissue morphology. Please verify whether the labeling of these panels is correct.
4.In Figure 5B, during the IP/MS-based identification of USP28-interacting proteins, the authors focused on NMMHC IIA for subsequent mechanistic analyses. The authors are encouraged to clarify the criteria used to prioritise this candidate, such as peptide counts or enrichment fold.
5.In Figure 5L, the identification of K1413 as the key deubiquitination site is convincing. Please clarify whether this site is evolutionarily conserved, which would strengthen the biological significance.
6.In Figure 6G, IFNAR1 membrane expression is presented. Please clarify whether this result was obtained using membrane protein fractionation, as this method does not appear to be explicitly described in the Methods section.
7.Cell apoptosis, pyroptosis and other forms of programmed cell death have also been reported to play important roles in the pathogenesis of sepsis. The authors are encouraged to further discuss why the present study specifically focuses on the role of USP28 in endothelial necroptosis.
8.A few grammatical inconsistencies and typographical errors are present (e.g., “IFANR1” instead of “IFNAR1” in one section) and should be corrected during revision.

9.Overall, the study is carefully executed and the data are of high quality. Because of the wealth of data, the arguments are not always easy to follow. A graphical abstract detailing the main molecular findings described in the manuscript would improve clarity for the reader.

Version 1:

Reviewer comments:

Reviewer #1

(Remarks to the Author)
The authors should be commended for their thoughtful and thorough revisions. The revised manuscript has addressed all of my concerns and is suitable for publication as is.

Reviewer #3

(Remarks to the Author)
The authors' previously submitted manuscript suffered from substantial data quality issues, which was my primary reason for raising concerns and recommending against publication. I note significant improvements in the data quality of this revised submission, and the authors' efforts during the revision process are highly commendable. While most of my initial concerns have been addressed with reasonable explanations, several points still require further clarification.

1. Issues remain with some of the immunofluorescence (IF) images. Specifically, proteins with distinctly different subcellular localizations—such as ZBP1 (cytoplasm), pRIPK1 (cytoplasm), pRIPK3 (cytoplasm), IFNAR1 (cell membrane and cytoplasm), and pSTAT1 (nucleus)—all appear to be predominantly localized to the nucleus in the IF images of lung tissues. This is highly unusual and anomalous. The authors must address this issue, as it directly impacts the validity and biological rationale of the experimental data. Furthermore, when quantifying these IF results, the authors should evaluate the fluorescence intensity specifically within lung endothelial cells rather than calculating the overall intensity across the entire lung tissue.

2. Although the authors have resolved the quality issues regarding the tissue section data, it must be clarified that while perfusion can indeed cause alveolar collapse or minor structural deformation, it should not result in massive structural destruction. The lung pathology images presented in the original manuscript were largely unacceptable from the perspective of pulmonary pathologists.

3. While it is common practice to employ CLP models of varying severities when independently assessing survival rates versus organ function, applying different severities of CLP across different experimental groups within the same study/comparison seems quite unusual. Could the authors provide references to other studies or researchers that have utilized a similar experimental design?

Reviewer #4

(Remarks to the Author)
All concerns have been addressed.

Reviewer #5

(Remarks to the Author)
All questions of reviewer 2 were adequately answered

Suppl figure 14 is an informative graphical abstract, but I miss what all purple arrows mean. Are all relations positive feedback loops ? The legend to suppl figure 14 is to short to be a stand-alone figure. Please expand and explain

Version 2:

Reviewer comments:

Reviewer #3

(Remarks to the Author)
The author has resolved all my queries and concerns. The manuscript is now of publishable quality. I appreciate the hard work put in by the author and their team in revising the paper.

Open Access This Peer Review File is licensed under a Creative Commons Attribution 4.0 International License, which permits use, sharing, adaptation, distribution and reproduction in any medium or format, as long as you give appropriate credit to the original author(s) and the source, provide a link to the Creative Commons license, and indicate if changes were made.
In cases where reviewers are anonymous, credit should be given to 'Anonymous Referee' and the source.
The images or other third party material in this Peer Review File are included in the article's Creative Commons license, unless indicated otherwise in a credit line to the material. If material is not included in the article's Creative Commons license and your intended use is not permitted by statutory regulation or exceeds the permitted use, you will need to obtain permission directly from the copyright holder.
To view a copy of this license, visit https://creativecommons.org/licenses/by/4.0/

Responses to the Reviewers' comments

Title: USP28 potentiates endothelial necroptosis via deubiquitinating NMMHC IIA in sepsis

Manuscript Number: NCOMMS-25-86271

We sincerely thank the reviewers for their time and effort in carefully evaluating our manuscript. Their constructive and insightful comments have greatly helped improve the quality and clarity of this work. We have taken every comment with the utmost seriousness and have dedicated substantial time to addressing each point comprehensively. In response to the reviewers' suggestions, we have performed multiple additional experiments, added nine supplementary figures, revised all main figures, and extensively revised the manuscript text. Our responses reflect a thorough consideration of the reviewers' suggestions, including additional experiments where warranted. We believe that these revisions have significantly strengthened our study, and we would be honored to have the manuscript reevaluated for potential publication in *Nature Communications*.

The following is a point-by-point response to the reviewers' comments. Due to file size limitations, key data are included within this document, and high-resolution of all figures are provided in the accompanying files for clarity.

Reviewer Comments

Reviewer #1 (Remarks to the Author):

In this manuscript, Tang et al. investigates the role of the deubiquitinase USP28 in regulating endothelial cell necroptosis during sepsis. Specifically, the authors isolate circulating endothelial cells (CECs) from sepsis patients and healthy donors to perform comparative proteomic profiling, with the goal of identifying key molecular drivers of sepsis-induced endothelial dysfunction. Through this approach, they identify USP28 as significantly upregulated in septic patients (especially non-survivors). They then use a combination of cell culture and animal models to demonstrate that elevated endothelial USP28 promotes necroptotic cell death in sepsis, ostensibly via a novel signaling axis involving the substrate NMMHC IIA and downstream interferon (IFN) pathway activation.
A notable strength of the study is its use of advanced proteomics methodologies to tackle a clinically relevant problem. The authors employ "nanoproteomics" for the single-cell proteomics analysis of rare CECs, enabling detection of over 1,000 proteins from limited samples. Using nanoproteomics to profile CECs, they demonstrate an interesting correlation between endothelial USP28 expression and sepsis-induced

multi-organ injury. In addition, the authors perform additional immunoprecipitation-coupled mass spectrometry (IP-MS) to uncover proteins interacting with USP28. The combination of unbiased proteomics analysis for discovery with targeted validation is a powerful aspect of the work. That said, the proteomics techniques used here are inherently complex, and certain technical details (e.g. data analysis pipelines) are only briefly described. A more thorough discussion of these methodological aspects would strengthen the manuscript by helping readers appreciate the robustness and limitations of the data.

Overall, the study's findings appear novel and significant. Prior to this work, the involvement of deubiquitinating enzymes in endothelial necroptosis during sepsis was not well defined. Tang et al. reveal a new USP28–NMMHC IIA molecular axis that links protein ubiquitination status to necroptotic signaling. Their data suggest that USP28 stabilizes NMMHC IIA by removing K48-linked ubiquitination at Lys1413, preventing its proteasomal degradation. Stabilized NMMHC IIA then enhances IFNAR1/STAT1 pathway signaling, thereby exacerbating inflammatory cell death. This mechanistic insight is supported by both their proteomics data and functional assays, and it points to USP28 as a potential therapeutic target. To this end, the authors showed that endothelial-specific USP28 deficiency (using USP28AAV9-Tie1-sh) improved the survival of septic mice. Moreover, the authors demonstrated that intragastrically administration of USP25/28 inhibitor AZ1immmediatly prior to CLP surgery led to reduced mortality and increased body temperature of cecal ligation and puncture (CLP)-treated mice, suggesting that pharmacological inhibition of USP28 can be protective in sepsis models.

Ultimately, this manuscript tackles an important question with innovative proteomic approaches and provides compelling evidence for a novel mechanism in sepsis pathology. The authors should be commended for bringing together cutting-edge proteomics and mechanistic biology in a challenging clinical context. With appropriate revisions it has the potential to make a valuable contribution to the scientific community. Therefore, I recommend the manuscript be considered for publication only after addressing my concerns raised above (vide supra) and the following specific concerns presented below:

Response: We sincerely thank the reviewer for the careful evaluation of our manuscript and for the constructive and encouraging comments. We are grateful for the reviewer's positive assessment of the novelty and significance of our work, as well as the recognition of our proteomics-based approach to investigate endothelial dysfunction in sepsis. We have carefully addressed all of the reviewer's specific comments below and revised the manuscript accordingly.

1. While the manuscript is generally well written, the authors do not fully leverage their Figures as major tools for effective scientific communication. Several Figures illustrate the raw data well but do not see to effectively convey their key conclusions clearly. In particular, the results shown in Figures 1B-D, 3C, 5B, 6B, and 8H are crowded, and the text and data are difficult to interpret. Given the complexity of the relationships among USP28, NMMHC IIA, IFNAR1/STAT1 signaling, IFN-I, and sepsis, the authors are strongly encouraged to include a schematic model summarizing these interactions as a part of the discussions (perhaps even as a new Figure).

Response: We thank the reviewer for this helpful suggestion regarding figure clarity and data presentation. To improve readability and facilitate interpretation of the data, we have carefully reorganized the layout of several figures in the revised manuscript. Specifically, Figures 1B–D, 3C, 5B, 6B, and 8H have been reformatted with enlarged panels, improved spacing, and clearer labeling to ensure that the text and key features of the data are easily readable.

In addition, following the reviewer's suggestion, we have added a new schematic model summarizing the proposed USP28–NMMHC IIA–IFNAR1/STAT1 signaling axis and its role in endothelial necroptosis during sepsis **(updated Supplementary Fig. 14)**. This model is included to provide readers with an integrated overview of the mechanistic relationships described throughout the study. We believe these revisions substantially improve the clarity of the figures and enhance the overall presentation of the study.

2. The proteomics approach is exciting and clearly a key strength of the study. However, to help readers fully understand these results, the manuscript would benefit from additional methodological detail and clarity. For example, it should be clarified how the data analysis for all the proteomics results were actually performed: software, settings, tolerances for MS1/MS2, etc. For instance, in the IP-MS results, MaxQuant is mentioned for "identification and quantification" without any mention of how the analysis was performed. For the nanoproteomics results, it appears no mentions were made to how the data was eventually processed. Providing this information will bolster confidence that the proteomic findings are robust and reproducible. I encourage the authors to also supply the complete list of their search results showing the peptides/proteins identified and their differential expression statistics, so that readers can appreciate the full dataset underlying the focus on USP28.

Response: We thank the reviewer for this constructive suggestion and for recognizing the proteomics analysis as an important strength of the study. To improve methodological transparency, we have expanded the Methods section in the revised manuscript to provide a more detailed description of the proteomics data analysis

workflow. Specifically, we now clarify the database search strategy and identification parameters, including the use of MaxQuant (v2.1.3.0) with the integrated Andromeda search engine, the reference database, enzyme specificity, peptide length threshold, fixed and variable modifications, as well as the false discovery rate (FDR) control applied at both the peptide-spectrum match and protein levels. In addition, the procedures used for protein quantification (LFQ and iBAQ) and the statistical criteria for differential expression analysis have been explicitly described **(Pages 25–26, Lines 570–597)**.

Regarding the availability of the complete proteomics results, the raw mass spectrometry data and processed search results have been deposited in the ProteomeXchange partner repository iProX, where they are publicly accessible under the accession number PXD071841. This repository contains the full identification and quantification results, enabling readers to examine the complete dataset underlying the discovery of USP28 and related proteins **(Page 34, Lines 763–768)**. These revisions improve the clarity of the analytical pipeline and ensure that the proteomics data supporting this study are fully transparent and reproducible.

3. Line 52, Should the abbreviation of "pathogen-associated molecular patterns" be "PAMPs" instead of the used "DAMPs"? If so, the authors should carefully address this wording throughout the manuscript.

Response: We thank the reviewer for pointing out this issue. The abbreviation in line 52 was incorrect and has been corrected from "DAMPs" to "PAMPs" to accurately refer to pathogen-associated molecular patterns **(Page 3, Lines 51–54)**. We have carefully reviewed the manuscript and ensured that this terminology is used correctly and consistently throughout the text.

4. Line 327, the concluding statement "USP28 inhibits sepsis progression by inhibiting ubiquitination …" seems to contradict all the authors results. Did the authors mean to phrase this differently?

Response: We apologize for the oversight and appreciate the reviewer bringing this to our attention. The original sentence was incorrectly phrased and did not accurately reflect the conclusions of our study. We have corrected this statement in the revised manuscript **(Page 18, Lines 390–391)**.

5. In this manuscript the authors introduce the term "nanoproteomics" as an "emerging sequencing technology", but this term has been used in prior literature to describe interdisciplinary proteomics technologies that enable the identification and characterization of low-abundance proteoforms (see PMCID: PMC7411019; PMC6656398; PMC10728164). I recommend the authors briefly clarify this point in

the manuscript and cite the relevant prior studies, thereby acknowledging the existing work in this emerging area.

Response: We thank the reviewer for this helpful comment. We agree that the term nanoproteomics has been used in prior studies to describe mass-spectrometry–based proteomic approaches designed to analyze extremely small amounts of protein material and to characterize low-abundance proteoforms. In the revised manuscript, we have clarified the description of nanoproteomics and replaced the phrase "emerging sequencing technology" with more accurate wording describing it as a mass-spectrometry–based proteomics approach for low-input samples **(Page 125, Lines 323–325)**. In addition, we have cited the relevant prior studies suggested by the reviewer to acknowledge previous work in this area **(Page 39, Lines 868–874)**. These revisions help place our study within the broader context of emerging low-input proteomics technologies.

6. The manuscript is focused on endothelial USP28, have the authors consider whether immune-cell USP28 indirectly contributes to cytokine production or IFN-I signaling, which can affect the in vivo data?

Response: We thank the reviewer for raising this important point. To better define the cellular distribution of USP28, we have now performed multiplex immunofluorescence staining in lung tissue, including markers for endothelial cells (CD31), macrophages (F4/80), alveolar epithelial cells (SP-C), and smooth muscle/myofibroblasts (α-SMA), together with USP28. These analyses demonstrate that, although USP28 is detectable in multiple cell types, its upregulation following CLP is most prominently observed in endothelial cells, supporting a preferential induction of USP28 in this compartment under septic conditions **(updated Supplementary Fig. 1)**. Moreover, the experimental strategy in the present study was specifically designed to investigate the role of endothelial USP28. Importantly, our initial discovery originated from nanoproteomics analysis of circulating endothelial cells (CECs) isolated from patients with sepsis, in which USP28 was identified as a significantly upregulated protein in endothelial cells, and this observation motivated our subsequent focus on the endothelial function of USP28. *In vivo* manipulation was achieved using an endothelial-targeting AAV9-Tie1 and CDH5-Cre system for endothelial cell-specific genetic manipulation. The key mechanistic findings were consistently validated in primary mouse lung endothelial cells **(updated Supplementary Fig. 6, updated Supplementary Fig. 9, updated Supplementary Fig. 12)**. These experiments demonstrate that USP28 directly regulates NMMHC IIA stability and downstream IFNAR1/STAT1 signaling in endothelial cells. Therefore, although we cannot completely exclude the possibility that USP28 expressed in immune cells may influence inflammatory signaling during sepsis,

the combination of endothelial-targeted manipulation in vivo and endothelial cell-based mechanistic experiments strongly supports a predominant endothelial cell–intrinsic role for USP28 in the phenotypes observed in this study. We have now added a brief discussion in the revised manuscript to acknowledge the potential contribution of other cell types **(Page 17, Lines 377–380)**.

updated Supplementary Fig. 1

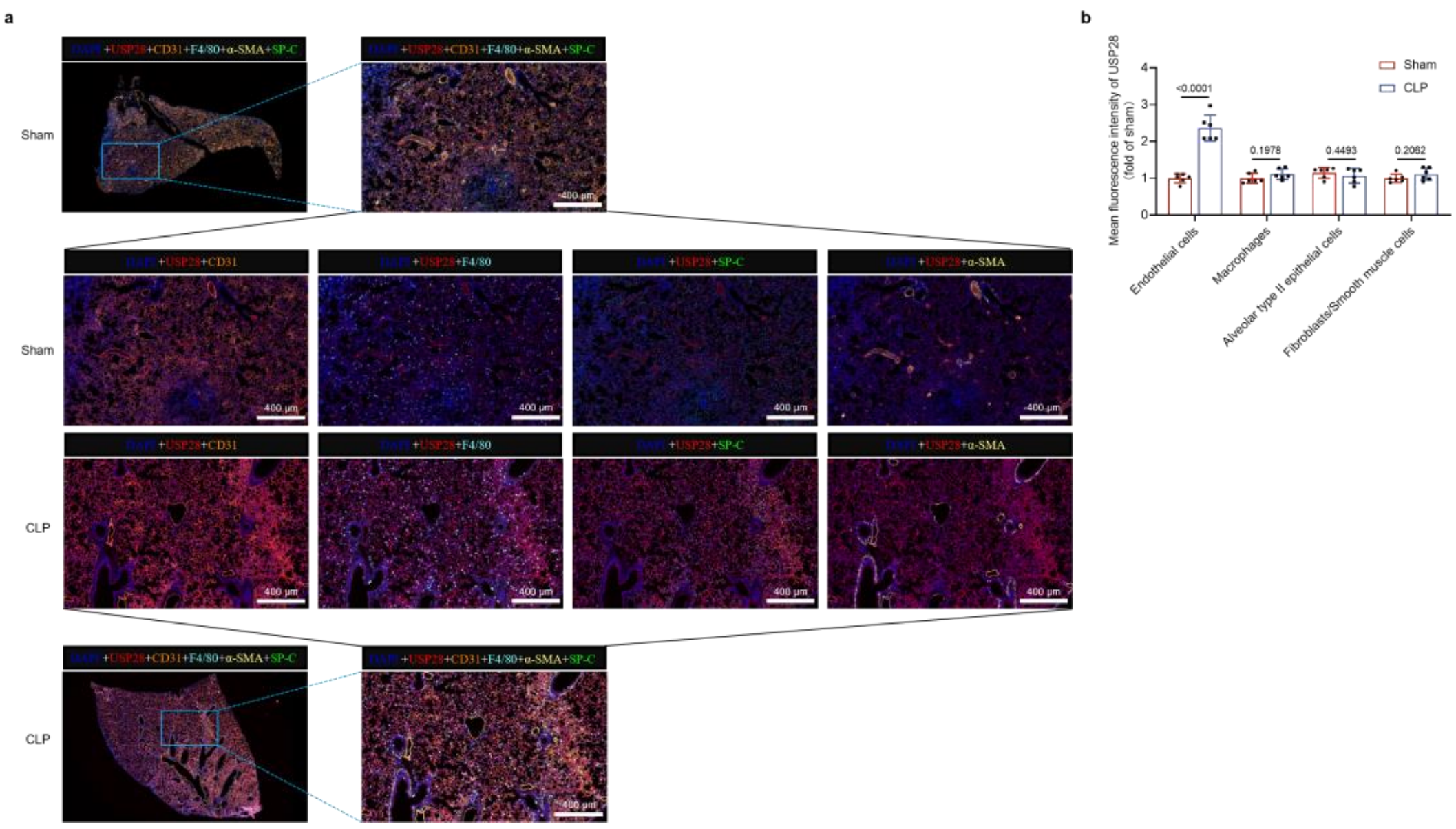

Reviewer #2 (Remarks to the Author):

This manuscript reports a substantial and technically sophisticated body of work, but several issues in design, interpretation, and presentation justify a recommendation of major revision before it can be considered further.

Response: We thank the reviewer for the careful and thoughtful evaluation of our manuscript. We appreciate the reviewer's recognition of the substantial and technically sophisticated work presented in this study. We have carefully addressed the concerns regarding study design, interpretation, and presentation raised by the reviewer, and have revised the manuscript accordingly. A detailed point-by-point response to each comment is provided below.

1. Novelty and conceptual advance

The central USP28–NMMHC IIA–IFNAR1–STAT1–necroptosis axis is interesting, but the authors should more clearly delineate what is conceptually new compared with prior work on NMMHC IIA in endothelial integrity and IFN-I–induced necroptosis (e.g., ZBP1–MLKL axis and NMMHC IIA in LPS-induced lung injury) and explicitly discuss overlapping pathways rather than largely restating background.

The claim that the USP28–NMMHC IIA axis "promotes sepsis pathology" and represents a "promising therapeutic target" feels somewhat overstated relative to the modest sample size of human data and the constraints of a single CLP model; these translational claims should be toned down or more rigorously qualified.

Response: We thank the reviewer for this thoughtful comment regarding the conceptual positioning of our work. We agree that both NMMHC IIA and IFN-I–associated necroptosis pathways have been studied previously in endothelial biology and inflammatory injury. In the revised manuscript, we have expanded the Discussion and removed some repetitive background to more clearly delineate the conceptual advance of our study relative to these prior findings **(Page 16, Lines 349–359; Pages 16–17, Lines 362–364)**. Specifically, our work identifies USP28 as a previously unrecognized deubiquitinase that stabilizes NMMHC IIA, thereby linking ubiquitin-mediated protein regulation to endothelial inflammatory signaling during sepsis. Furthermore, our data reveal that stabilized NMMHC IIA promotes IFNAR1 membrane trafficking and activation of IFNAR1/STAT1 signaling, establishing a mechanistic connection between the ubiquitin system and interferon-driven endothelial necroptosis. These findings therefore extend previous work by defining a USP28–NMMHC IIA regulatory axis upstream of IFN-I signaling, which has not been described in the context of sepsis-associated endothelial dysfunction.

We also agree that the original wording may have overstated the translational implications of our findings. In the revised manuscript, we have moderated the relevant statements to better reflect the scope of our data. Specifically, we replaced broad expressions such as "promoting sepsis pathology" with more precise descriptions focused on endothelial injury and necroptosis during sepsis. In addition, the statement describing the USP28–NMMHC IIA axis as a "promising therapeutic target for treating sepsis" has been revised to a more cautious formulation, indicating that USP28 may represent a potential target for therapeutic intervention in sepsis. These revisions ensure that the conclusions remain consistent with the mechanistic evidence presented in this study while avoiding overstatement of the translational implications **(Page 14, Lines 310–312; Pages 15–16, Lines 339–341; Page 17, Lines 371–373; Page 18, Lines 390–394)**.

2. Human cohort design and characterization

The clinical cohort of sepsis patients used for CEC nanoproteomics appears relatively small, and the criteria for inclusion, exclusion, timing of sampling, infection source, and organ support (vasopressors, ventilation, RRT) are not fully described; more detailed clinical characterization and a table comparing survivors vs non-survivors are needed to interpret the correlation with SOFA and IFN-I levels.

It is unclear whether potential confounders (age, comorbidities, immunosuppression, steroid or IFN therapy, baseline endothelial disease) were controlled in the analysis linking CEC USP28/NMMHC IIA expression to outcomes; multivariable or at least stratified analyses should be presented or limitations clearly acknowledged.

Response: We thank the reviewer for underscoring the importance of comprehensive clinical characterization of the human sepsis cohort and careful consideration of potential confounding factors. In response to these comments, we have revised the manuscript to provide a more detailed description of the clinical cohort. Specifically, we have clarified the patient inclusion and exclusion criteria (all enrolled patients met Sepsis-3 diagnostic criteria, and patients with pregnancy or breastfeeding, malignancy, organ transplantation, HIV infection, autoimmune disease, or immunosuppressive therapy were excluded) and specified the timing of blood sampling, which was performed within 24 h after patient enrollment **(Page 18, Lines 397–405)**. In addition, we have expanded the clinical characterization of the cohort by including demographic and clinical information such as sex, age, infection source, major treatments and organ support measures (e.g., mechanical ventilation), SOFA scores, and clinical outcomes **(updated Supplementary Table 1)**.
We also appreciate the reviewer's concern regarding potential confounding factors. Due to the relatively small size of this exploratory clinical cohort, comprehensive multivariable analyses adjusting for confounders, including age, comorbidities, immunosuppression status, exposure to steroids or interferon therapy, and baseline endothelial disease, were not feasible. These limitations are now explicitly acknowledged in the revised Discussion section **(Page 17, Lines 380–384)**. Importantly, the clinical analyses presented here were intended to provide clinically relevant context and generate testable hypotheses. The overall conclusions of this study are not driven by the human cohort correlations alone but are supported by independent experimental analyses demonstrating the functional relevance of USP28 in sepsis-associated endothelial dysfunction and multi-organ injury.

3. Definition and validation of CECs
The gating strategy (Hoechst^{+}7-AAD^{-}CD45^{-}CD34^{+}CD146^{+}) is plausible but the manuscript does not show rigorous validation that these events are bona fide mature CECs rather than progenitors or platelet–leukocyte aggregates; additional phenotypic markers, imaging cytometry, or parallel endothelial-specific transcripts/proteins would strengthen this aspect.
Because nanoproteomics is performed on extremely rare cells, the authors need to discuss and, where possible, quantify purity, doublet rates, and potential leukocyte contamination, and how these may bias the identification of USP28 as a top hit.

Response: We thank the reviewer for the thoughtful comments regarding the definition and validation of circulating endothelial cells (CECs), as well as quality control considerations associated with nanoproteomic analysis of extremely rare cell populations. With respect to CEC identification, the gating strategy (Hoechst^{+}7-AAD^{-}CD45^{-}CD34^{+}CD146^{+}) used in this study is widely adopted and well-established in previous studies for the enrichment of CECs **(doi: 10.1038/s41598-018-24234-0; doi: 10.1002/ajh.25881; doi: 10.1038/s41598-018-36442-9; doi: 10.1158/1078-0432.CCR-08-0432)**. This strategy was designed to maximize enrichment of bona fide endothelial cells while minimizing non-endothelial contamination. Specifically, Hoechst positivity excludes non-nucleated events such as platelets and debris, 7-AAD negativity excludes non-viable cells, and CD45 negativity excludes hematopoietic cells. In addition, FSC/SSC-based singlet discrimination was applied prior to marker-based selection to reduce doublets and platelet–leukocyte aggregates. These methodological details have now been clarified in the revised Methods section **(Pages 19–20, Lines 429–440)**.
Because nanoproteomics was performed on an extremely rare cell population, we have further expanded the discussion of sorting purity and potential contamination. Although our gating strategy was designed to minimize contamination from leukocytes, platelets, and cell aggregates, we acknowledge that low-level contamination cannot be completely excluded. Given the extremely limited number of sorted cells, further phenotypic validation is technically challenging. These limitations, together with their potential impact on data interpretation, are now explicitly discussed in the revised manuscript **(Pages 17–18, Lines 384–389)**. Importantly, we note that the identification of USP28 as a key candidate is supported not only by the CECs nanoproteomics dataset but also by independent experimental validation, including endothelial-specific genetic manipulation and mechanistic studies *in vitro* and *in vivo*. Therefore, while the rare-cell proteomic analysis serves as a discovery platform, the overall conclusions of this study are supported by multiple complementary lines of evidence.

4. Nanoproteomics analysis and statistics
The proteomic pipeline (single-cell or nano-scale) is only briefly sketched; details about normalization, batch correction, missing-value imputation, statistical models, and multiple-testing correction (e.g., FDR thresholds) for DEGs and KEGG analyses should be provided, preferably in Methods and figure legends.
The intersection strategy that narrows 173 DEGs to four recurring genes and then prioritizes USP28 as “the key regulator” seems somewhat arbitrary; the rationale for focusing on USP28 over other candidates (TMED9, TPM3, PPGB) and whether they were systematically evaluated should be more transparently described.

Response: We thank the reviewer for this important comment and apologize for the lack of clarity in our original presentation regarding the candidate selection strategy. In this study, the identification of USP28 was not arbitrary but was based on a stepwise and data-driven prioritization process. First, differential expression analyses were performed between patients with sepsis and healthy donors, as well as between survivors and non-survivors. By intersecting these datasets, we identified four consistently altered proteins (USP28, TMED9, TPM3, and PPGB) as candidate molecules associated with sepsis severity. To further prioritize these candidates, we performed correlation analyses with clinical severity (SOFA score). Among the four proteins, USP28 showed a significant positive correlation with SOFA score, whereas TMED9, TPM3, and PPGB did not exhibit significant associations with disease severity **(updated Fig.1g–j)**. Given that SOFA score is a well-established indicator of multi-organ dysfunction in sepsis, this result suggested that USP28 is more closely linked to disease progression. Based on this combined evidence from differential expression and clinical correlation analyses, we selected USP28 for subsequent mechanistic investigation. We have now clarified this selection strategy in the revised manuscript **(Page 5, Lines 91–94)**.

updated Fig.1g–j

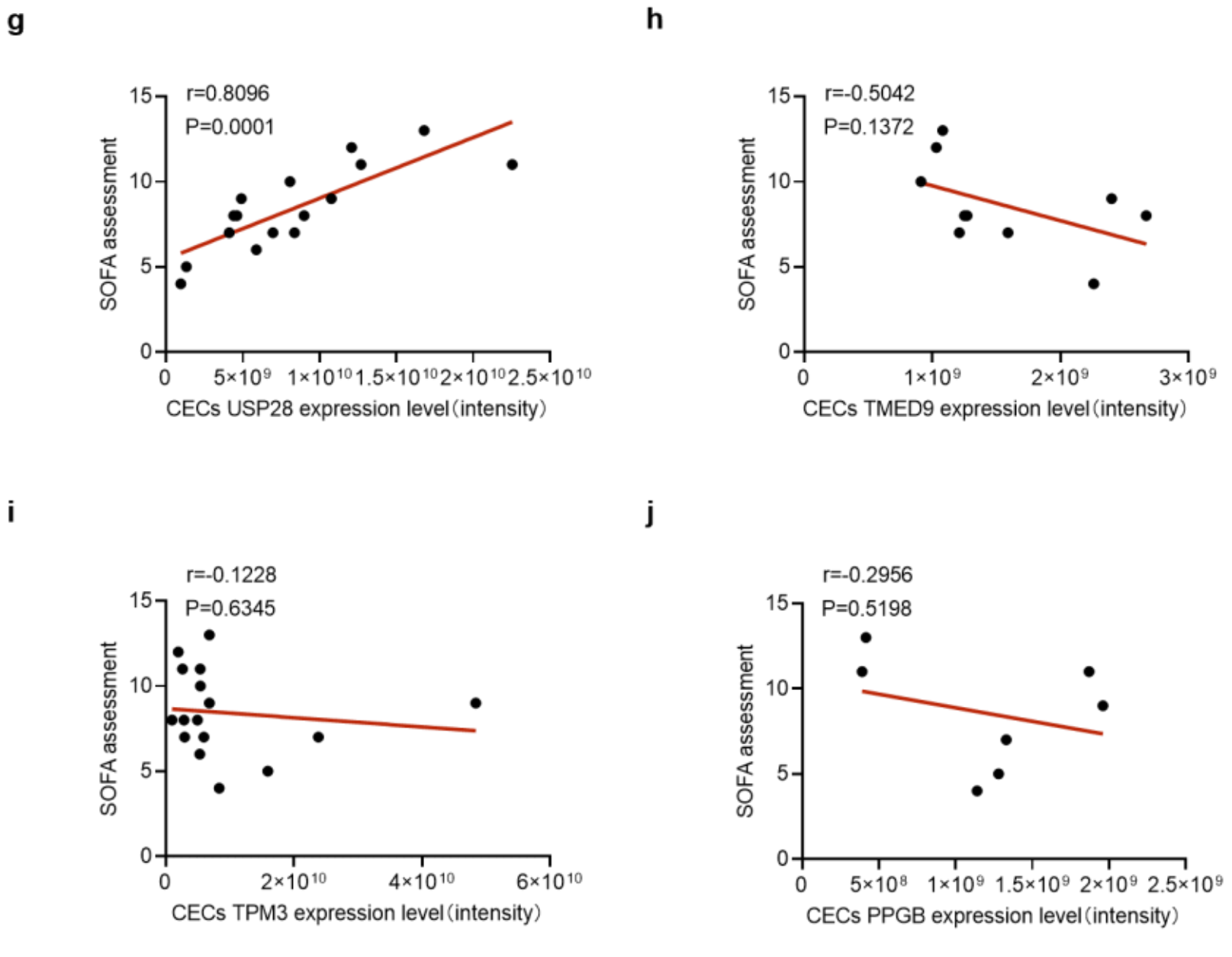

5. Mouse models and sepsis relevance

All in vivo work appears restricted to young male C57BL/6J mice; given the clinical relevance of age and sex in sepsis, the authors should at least acknowledge this more explicitly and, if feasible, provide data or a plan for older and/or female mice.

The CLP protocol (75% ligation, single puncture) yields a very severe phenotype;

justification of this severity and discussion of how it compares to human sepsis dynamics (particularly in relation to timing of interventions like AZ-1) would help interpret the therapeutic implications.

Response: We thank the reviewer for this important comment regarding the use of mouse models and their relevance to human sepsis. With respect to age and sex, we have now explicitly acknowledged this as a limitation in the revised manuscript **(Page 17, Lines 374–377)**. In this study, male mice were selected because of their lower variability in the caecal ligation and puncture (CLP) model compared with female mice. Sepsis is characterized by systemic inflammation and immune dysregulation, whereas female mice exhibit cyclic fluctuations in sex hormone levels that can influence inflammatory responses and tolerance to CLP-induced injury, thereby introducing additional variability **(doi: 10.1038/nprot.2008.214; doi: 10.1172/jci.insight.180132)**. In addition, female sex hormones have been reported to exert protective effects in sepsis-associated cardiac injury **(doi: 10.1038/s41598-022-08889-4)**. Therefore, the use of male mice is consistent with the majority of experimental sepsis studies and facilitates more stable and reproducible phenotypic assessment **(doi: 10.1016/j.phrs.2022.106473; doi: 10.1002/ptr.8093)**.

Regarding the CLP model, we employed a relatively severe protocol (75 % ligation with a single puncture) to ensure robust and reproducible induction of sepsis. The CLP model is a well-established and widely accepted preclinical model of polymicrobial sepsis, as it recapitulates key features of human sepsis, including systemic inflammation, immune dysregulation, and multi-organ dysfunction **(doi: 10.1038/nprot.2008.214; doi: 10.1038/s41418-020-0524-1; doi: 10.1038/s41467-020-20577-3)**. We agree that this represents a severe phenotype; therefore, to mitigate potential ceiling effects, we performed experiments using a milder CLP protocol (50 % ligation) in the context of endothelial-specific USP28 overexpression **(updated Supplementary Fig 4)**, and this has now been clearly described in the Methods section **(Page 21, Lines 460–462)**. In addition, in response to the reviewer's concern regarding the timing of intervention, we have now supplemented our study with experiments in which AZ-1 was administered after CLP surgery. These results further support the protective effect of pharmacological inhibition of USP28 under a more clinically relevant intervention paradigm **(updated Supplementary Fig 3; Page 6, Lines 122–132)**. Together, these additions and clarifications improve the interpretation and translational relevance of our *in vivo* findings.

updated Supplementary Fig 3

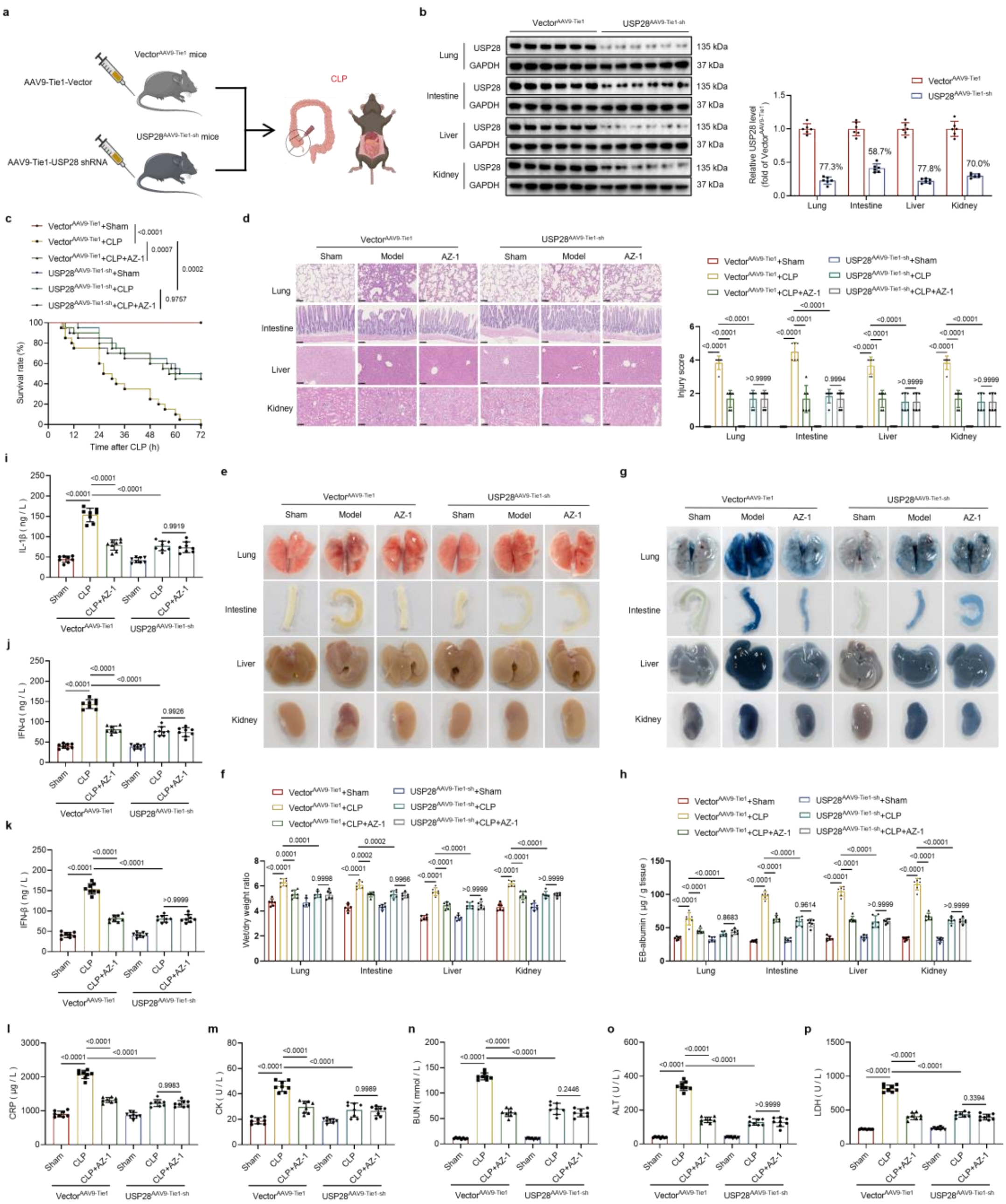

6. Specificity and timing of USP28 targeting

The timing of AZ-1 administration is essentially prophylactic (immediately prior to CLP), which does not mimic realistic clinical scenarios; the authors should include delayed-treatment experiments (e.g., 6–12 h post-CLP) or temper claims about therapeutic potential and explicitly label AZ-1 as proof-of-concept rather than a clinically viable regimen.

USP28 is expressed in multiple cell types; while endothelial-specific knockout/overexpression is used, AZ-1 will act systemically. The authors should clarify whether AZ-1 effects are EC-specific (e.g., through conditional models ± AZ-1) and discuss possible off-target or non-endothelial contributions.

Response: We thank the reviewer for this insightful comment regarding the timing of AZ-1 administration and the cell-type specificity of USP28 targeting. We fully agree that prophylactic treatment does not adequately reflect clinical scenarios. In response, we have now supplemented our study with additional experiments in which AZ-1 was administered at 1 h after CLP surgery to better approximate an early therapeutic intervention paradigm. Notably, post-CLP administration of AZ-1 produced protective effects comparable to those observed with pre-treatment, including improved survival, reduced levels of systemic pro-inflammatory cytokines, and alleviation of multi-organ injury **(updated Supplementary Fig. 3; Page 6, Lines 122–132)**. These findings indicate that pharmacological inhibition of USP28 remains effective after sepsis onset, thereby strengthening the translational relevance of our study.

Regarding the concern that AZ-1 acts systemically while USP28 is expressed in multiple cell types, we performed additional experiments using endothelial-specific USP28 knockout mice to determine whether the protective effects of AZ-1 are dependent on endothelial USP28. Importantly, we found that AZ-1 treatment failed to confer further protection in $USP28^{\text{AAV9-Tie1-sh}}$ mice, as reflected by the absence of additional improvements in survival, inflammatory cytokine production, and multi-organ injury. These results provide strong genetic evidence that the beneficial effects of AZ-1 are largely mediated through endothelial USP28 **(updated Supplementary Fig. S3; Page 6, Lines 122–132)**. Together, these additional experiments and clarifications demonstrate that USP28 inhibition exerts protective effects under a post-onset treatment paradigm and that these effects are predominantly dependent on endothelial USP28, thereby substantially strengthening both the mechanistic rigor and translational relevance of our study.

7. Necroptosis readouts and cell death specificity

Necroptosis is inferred mainly from increased pRIPK1/pRIPK3/pMLKL and ZBP1, but classic genetic or pharmacologic validation (e.g., $RIPK3^{-/-}$, $MLKL^{-/-}$ mice, or necrostatin-1) is lacking; at minimum, more stringent evidence that endothelial death is necroptotic rather than apoptotic/pyroptotic (e.g., cleaved caspase-3, GSDMD, TUNEL) should be provided.

In HUVECs, cell viability and barrier assays under IFN-I are not accompanied by direct necroptosis-specific functional readouts; adding rescue with necroptosis inhibitors or genetic silencing of RIPK3/MLKL would make the mechanistic conclusion more robust.

Response: We thank the reviewer for this important comment regarding the specificity of necroptosis and the need for more rigorous validation of cell death modalities. In response, we have performed additional experiments to more comprehensively

characterize the mode of endothelial cell death. Specifically, we examined representative markers of apoptosis and pyroptosis in both primary mouse lung endothelial cells (MLECs) and HUVECs under IFN-I stimulation. We found that IFN-I did not alter the expression of cleaved caspase-3 or GSDMD-N in MLECs, and neither USP28 deficiency nor USP28 overexpression affected the levels of these markers. Consistent results were obtained in HUVECs, in which IFN-I likewise failed to induce cleaved caspase-3 or GSDMD-N, and modulation of USP28 expression did not affect either apoptotic or pyroptotic marker levels. These data indicate that, under our experimental conditions, IFN-I-induced endothelial injury is not accompanied by detectable activation of apoptosis or pyroptosis **(updated Supplementary Fig. S6d–g; updated Supplementary Fig. S7i–l)**. In addition, to provide more direct functional evidence for necroptosis, we further performed loss-of-function experiments targeting key downstream mediators of the necroptotic pathway in HUVECs. Notably, silencing of MLKL or RIPK3 using siRNA almost completely blocked the IFN-I-induced reduction in cell viability, indicating that the endothelial injury observed in this setting is highly dependent on the canonical RIPK3-MLKL necroptotic axis **(updated Supplementary Fig. S7m–o)**. When considered together with our original data showing activation of IFNAR1/STAT1 signaling and increased expression of ZBP1, pRIPK1, pRIPK3, and pMLKL, these new findings provide much stronger evidence that USP28 regulates IFN-I-driven endothelial necroptosis rather than apoptosis or pyroptosis.

updated Supplementary Fig. S6d–g

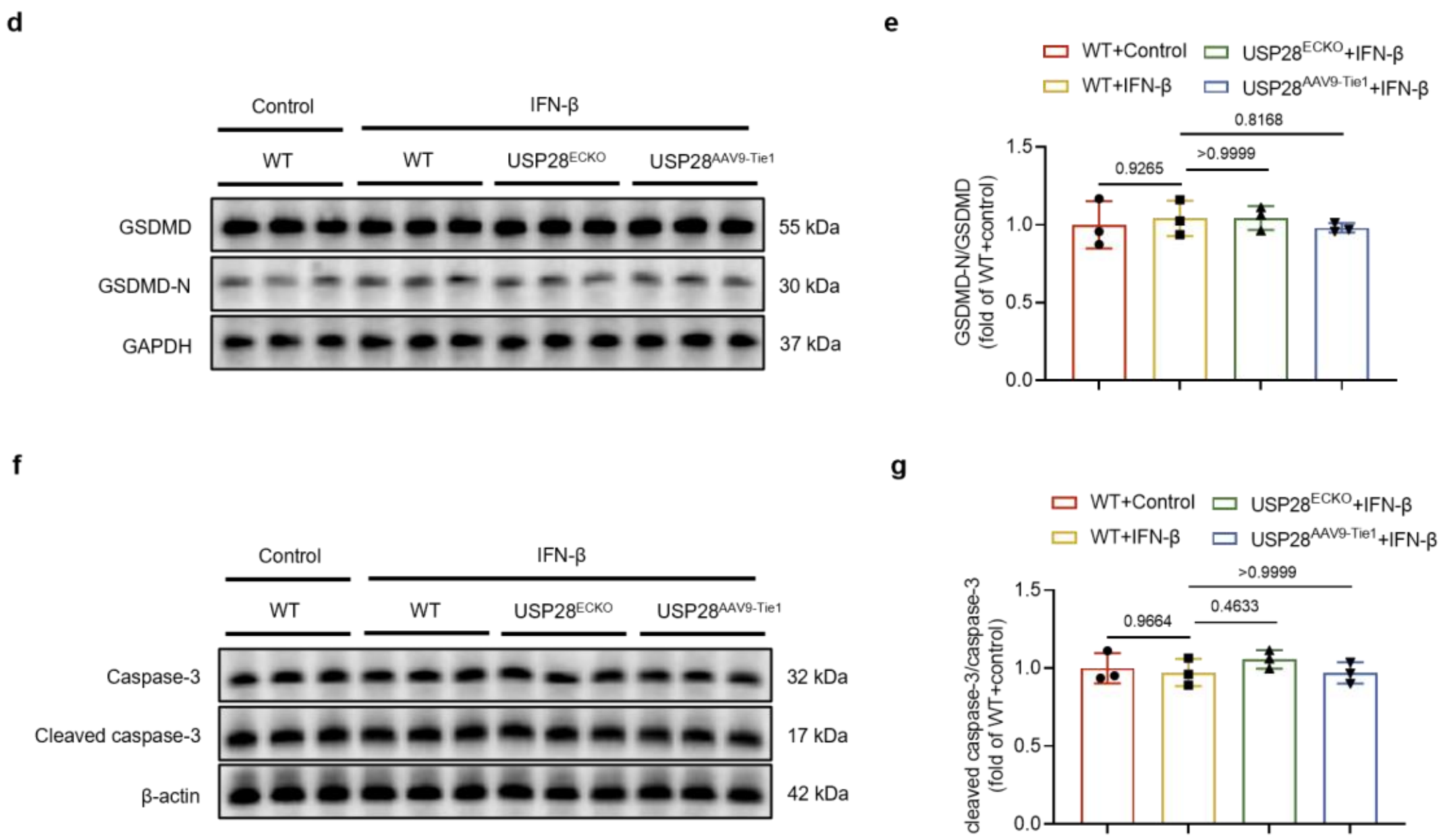

updated Supplementary Fig. S7i–o

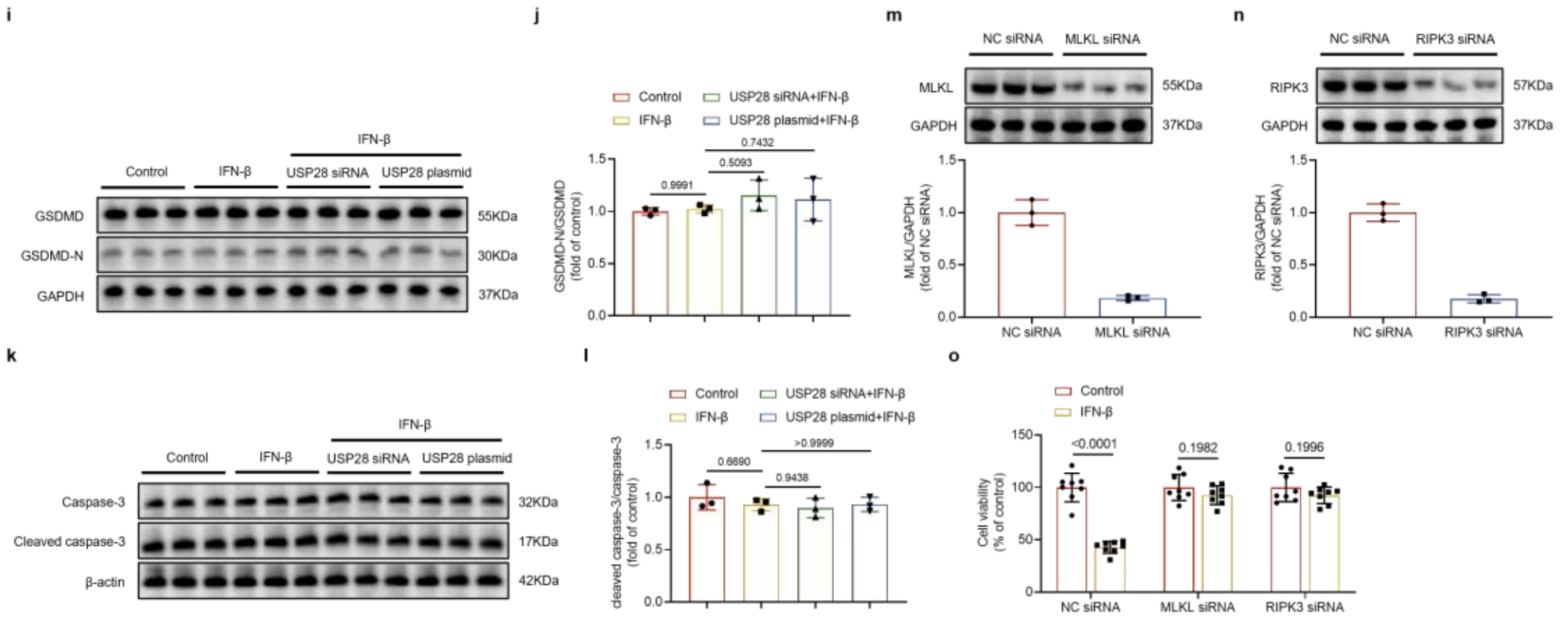

8. Mechanistic depth of the USP28–NMMHC IIA–IFNAR1 axis

While the mapping of USP28 and NMMHC IIA interaction domains is thorough, the functional necessity of K1413 deubiquitination is primarily inferred from mutant overexpression; the authors should test whether a deubiquitinase-dead USP28 mutant fails to stabilize NMMHC IIA and to promote IFNAR1 trafficking and necroptosis in ECs, ideally in vivo or in primary endothelial cells.

The link between NMMHC IIA and IFNAR1 membrane trafficking remains largely descriptive; mechanistic insight into trafficking pathways (e.g., endosomal markers, live-cell imaging, biotinylation assays) and whether NMMHC IIA affects IFNAR1 internalization vs anterograde transport would substantially strengthen this part of the story.

Response: We sincerely appreciate the reviewer's valuable suggestions regarding the mechanistic depth of the USP28–NMMHC IIA–IFNAR1 axis. We agree that additional evidence addressing the enzymatic requirement of USP28 and the trafficking mechanism of IFNAR1 would substantially strengthen this part of the study. In response, we have now performed additional experiments to further refine the mechanism. With respect to the functional necessity of USP28 deubiquitinase activity, we generated a deubiquitinase-dead USP28 mutant, USP28-C171S, and examined its effect on NMMHC IIA ubiquitination. Consistent with our original model, wild-type USP28 markedly reduced the ubiquitination level of NMMHC IIA, whereas the USP28-C171S mutant failed to do so, indicating that the deubiquitinating activity of USP28 is required for the removal of ubiquitin chains from NMMHC IIA. These new results provide direct evidence that the catalytic activity of USP28 is essential for its regulation of NMMHC IIA ubiquitination, thereby further strengthening our conclusion that USP28 stabilizes NMMHC IIA through its deubiquitinase function **(updated Fig. 5k)**. Regarding the mechanism by which NMMHC IIA regulates IFNAR1 membrane

trafficking, we further performed IFNAR1 biotinylation-based internalization assays. We found that neither NMMHC IIA silencing nor NMMHC IIA overexpression affected the internalization rate of IFNAR1 **(updated Fig. 6h)**. Therefore, when considered together with our previous data showing that NMMHC IIA positively regulates membrane IFNAR1 abundance **(updated Fig. 6g)**, these findings suggest that NMMHC IIA does not control IFNAR1 internalization, but rather promotes its anterograde trafficking to the plasma membrane.

updated Fig. 5k

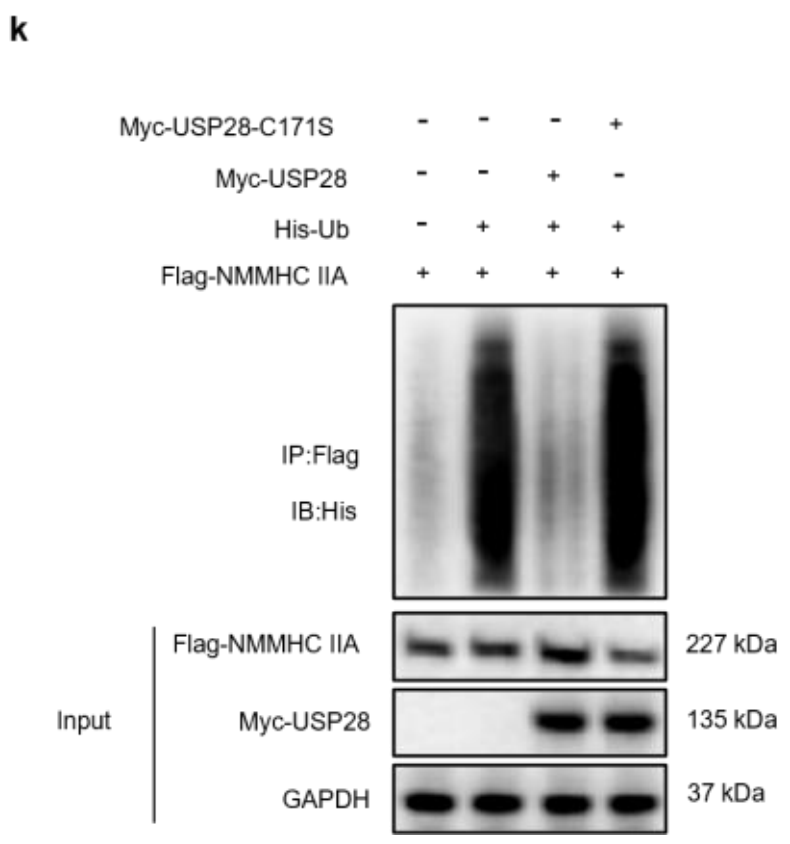

updated Fig. 6h

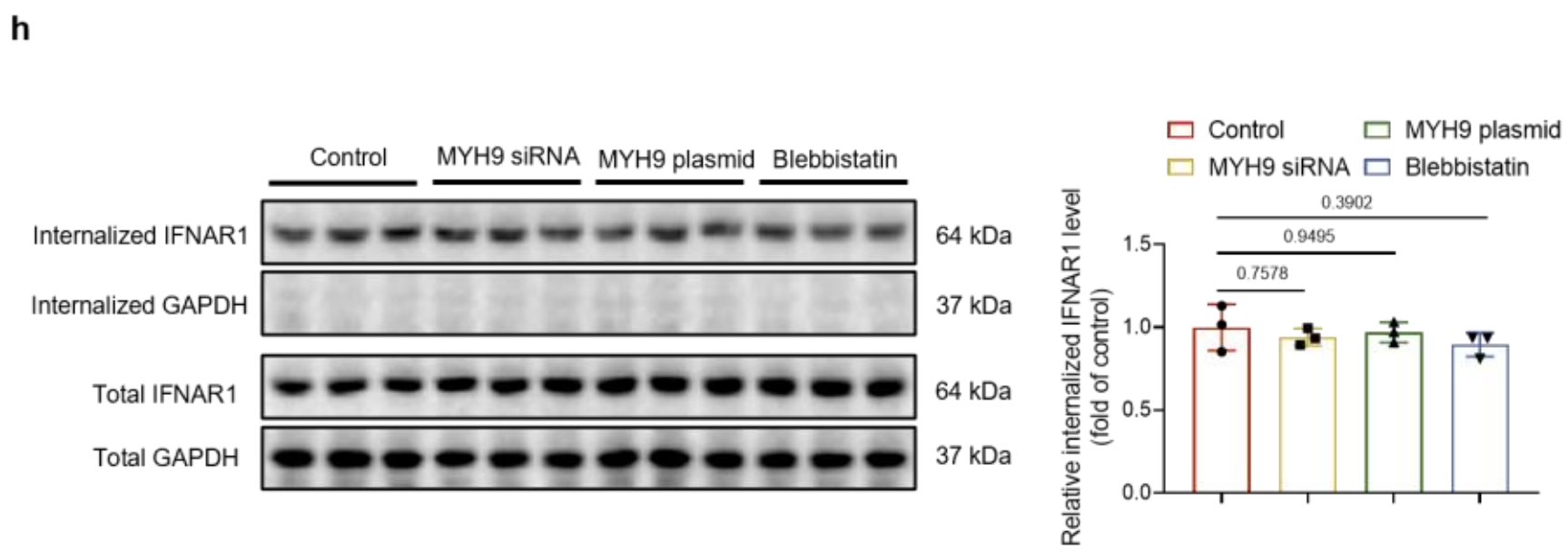

9. IFN-I signaling and feedback loop

The proposed positive feedback loop (IFN-I → STAT1 → USP28 transcription → NMMHC IIA stabilization → IFNAR1 trafficking → amplified IFN-I signaling) is appealing but only partially supported; for example, the dependence of IFNAR1 surface levels and downstream STAT1 activation on USP28/NMMHC IIA in vivo is not rigorously quantified (e.g., phospho-STAT1 and ISG expression in sorted ECs).

Given the complex and sometimes protective roles of IFN-I in sepsis and ARDS, the discussion should better integrate conflicting literature and explicitly clarify that the data primarily address excessive, sustained IFN-I in a specific CLP context rather than all sepsis settings.

Response: The reviewer raises an important point regarding the strength of evidence supporting endothelial-specific regulation within the proposed IFN-I–USP28–NMMHC IIA–IFNAR1 axis. We recognize that direct assessment of signaling changes in endothelial cells in vivo would further reinforce this aspect of the study. To more closely address this, we performed additional validation using primary mouse lung endothelial cells (MLECs). Notably, these primary endothelial cells recapitulated the molecular changes observed in our *in vivo* and *in vitro* experiments, showing consistent regulation patterns in response to IFN-I signaling and USP28 modulation **(updated Supplementary Fig. 6, updated Supplementary Fig. 9, updated Supplementary Fig. 12)**. These results provide additional evidence that the observed effects are intrinsic to endothelial cells rather than secondary to systemic or immune cell-derived influences. Together with our previous results, these data strengthen the evidence for an endothelial-centered feedback loop linking USP28–NMMHC IIA axis and IFN-I signaling. In addition, we have revised the Discussion to better contextualize the role of IFN-I signaling, emphasizing that our findings primarily reflect the effects of dysregulated and sustained IFN-I activation in the CLP-induced sepsis model rather than a universal role of IFN-I across all sepsis settings **(Page 16, Lines 349–359)**.

updated Supplementary Fig. 6

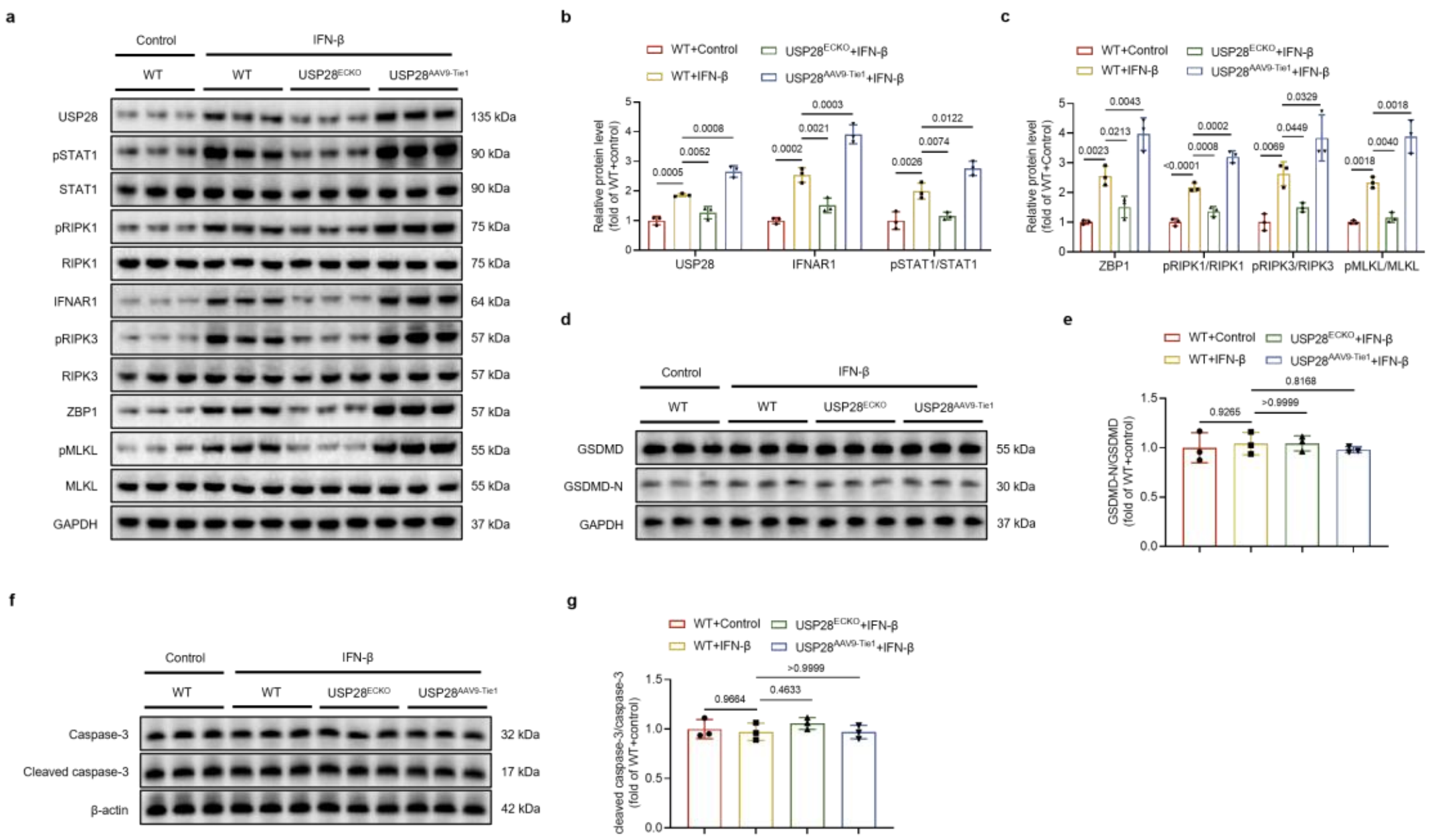

updated Supplementary Fig. 9

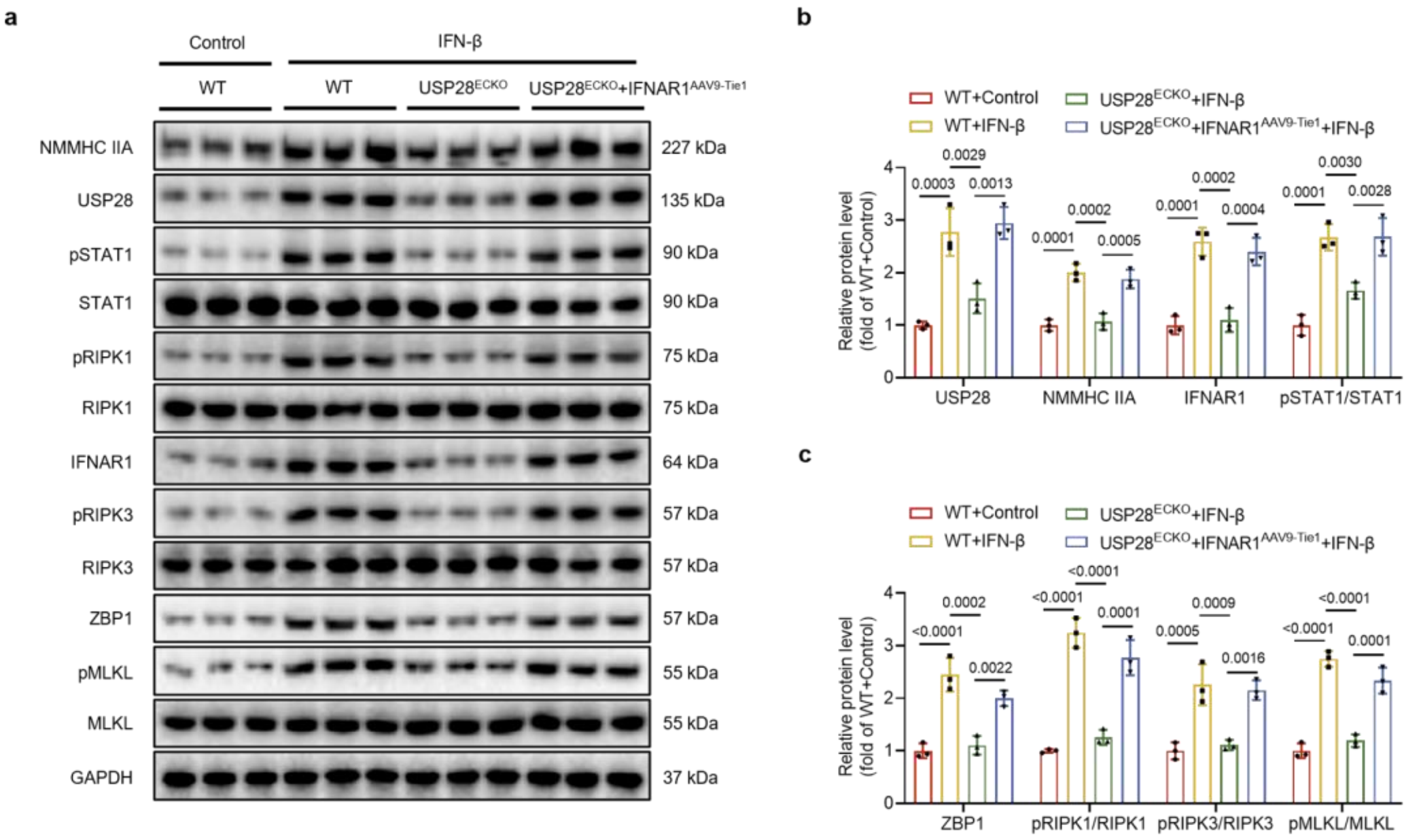

updated Supplementary Fig. 12

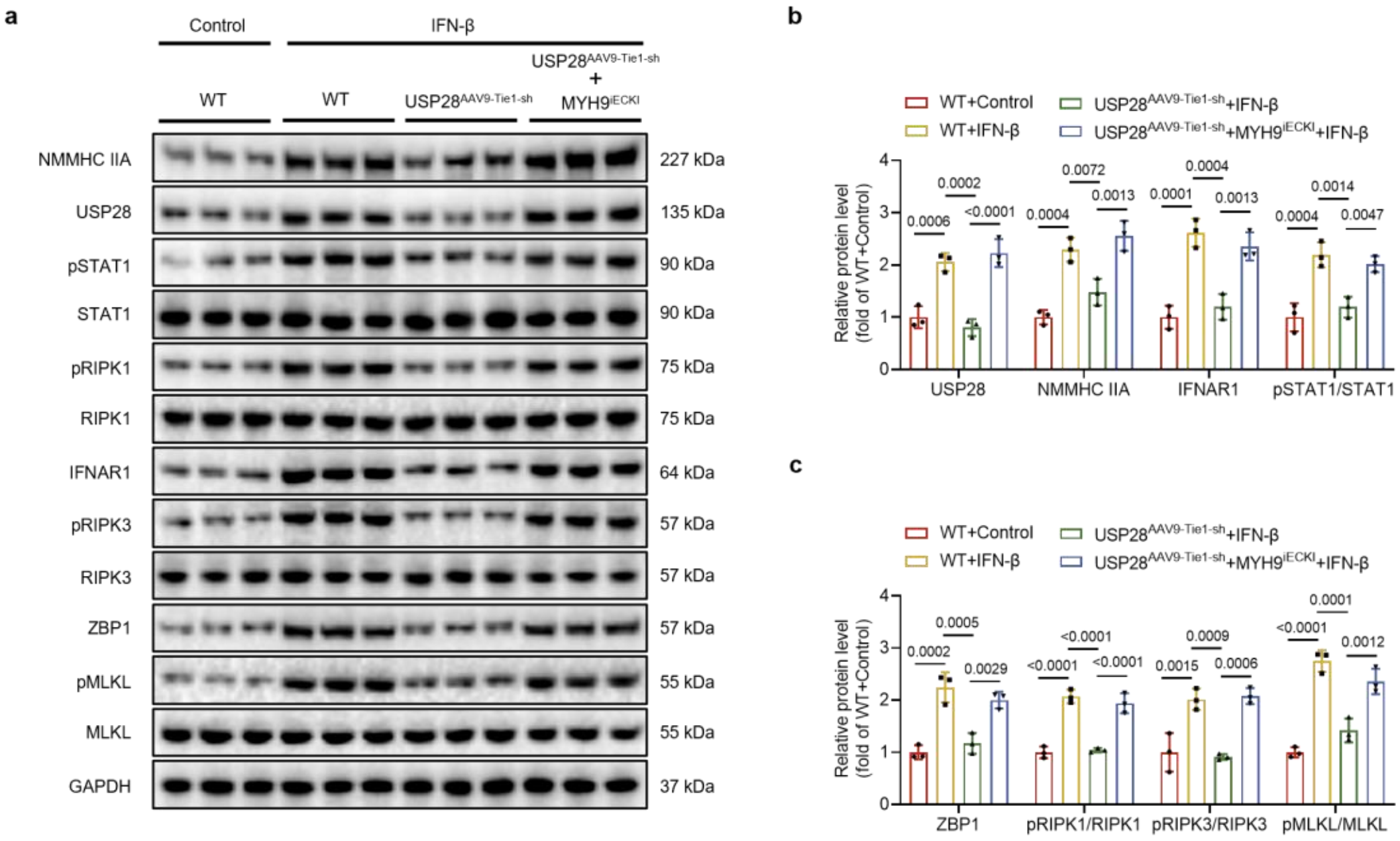

10. Overexpression rescue models and physiological relevance

The EC-specific overexpression of IFNAR1 or NMMHC IIA via AAV9 may produce supra-physiological levels that do not reflect human sepsis; the authors should show absolute or relative expression levels compared to endogenous in septic ECs and discuss whether the phenotypes might partly reflect non-physiologic receptor/myosin load.

In both IFNAR1AAV9-Tie1 and MYH9iECKI rescue experiments, overexpression/knockin appears to fully abrogate the protective effects of USP28

deficiency; the possibility of ceiling effects or pathway saturation should be addressed, and partial-rescue phenotypes or dose–response experiments would be informative.

Response: We sincerely appreciate the reviewer's insightful suggestion regarding the physiological relevance of the overexpression models and the possibility that supra-physiological expression levels may influence the observed phenotypes. To better address this concern, we have now quantified the extent of endothelial-specific overexpression achieved in our models across multiple organs. We found that IFNAR1 expression in endothelial cells was increased by approximately 2.2–4.0-fold across lung, intestine, liver, and kidney, while NMMHC IIA expression was elevated by ~1.7-fold **(updated Supplementary Fig. 8b, updated Supplementary Fig. 11b)**. These levels represent moderate upregulation rather than extreme overexpression. Importantly, consistent with our endothelial-specific USP28 overexpression model **(updated Supplementary Fig. 4)**, endothelial overexpression of IFNAR1 or NMMHC IIA alone did not induce detectable pathological changes in mice in the absence of CLP challenge **(updated Supplementary Fig. 8, updated Supplementary Fig. 11)**. This indicates that increased expression of these proteins is not sufficient to drive tissue injury by itself, but instead requires the presence of a pathological stimulus. In this context, our data suggest that the observed phenotypes arise from the interaction between elevated IFNAR1/NMMHC IIA levels and the dysregulated IFN-I signaling environment induced by CLP, rather than from non-physiological overloading of receptor or cytoskeletal components per se. These findings therefore mitigate the concern that the effects observed in our rescue models are merely artifacts of supra-physiological expression.

With respect to the observation that IFNAR1 or NMMHC IIA overexpression largely abrogates the protective effects of USP28 deficiency, we agree that this strong rescue phenotype may reflect efficient re-engagement of the downstream signaling axis and potentially a ceiling effect at the level of IFN-I signaling. However, these models are primarily intended to establish pathway dependency, demonstrating that restoration of IFNAR1 or NMMHC IIA is sufficient to override the protection conferred by USP28 deficiency. At the same time, we acknowledge that dose–response or partial-rescue experiments would provide additional insight into pathway sensitivity and saturation, and we have noted this as an important direction for future studies.

updated Supplementary Fig. 8

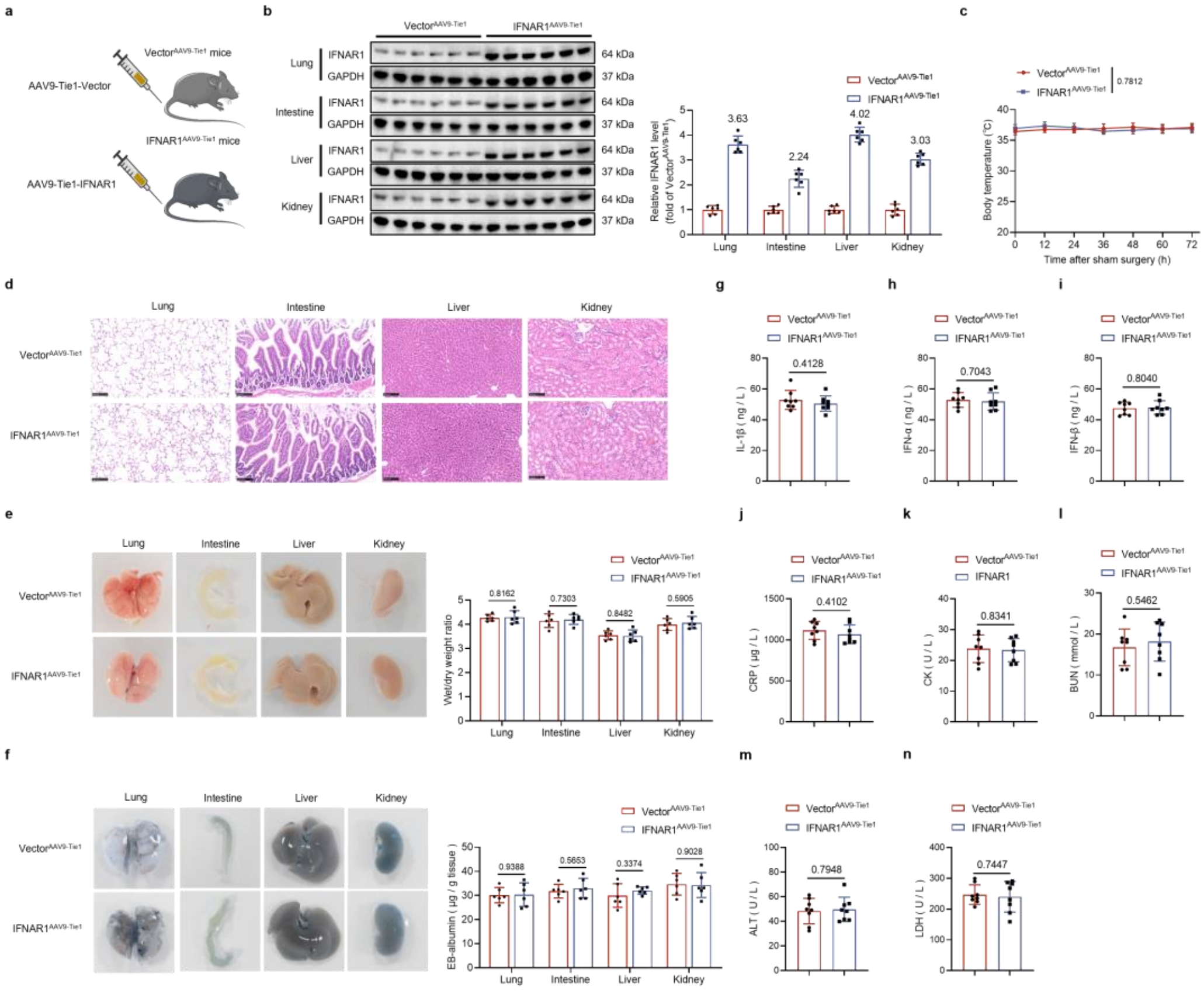

updated Supplementary Fig. 11

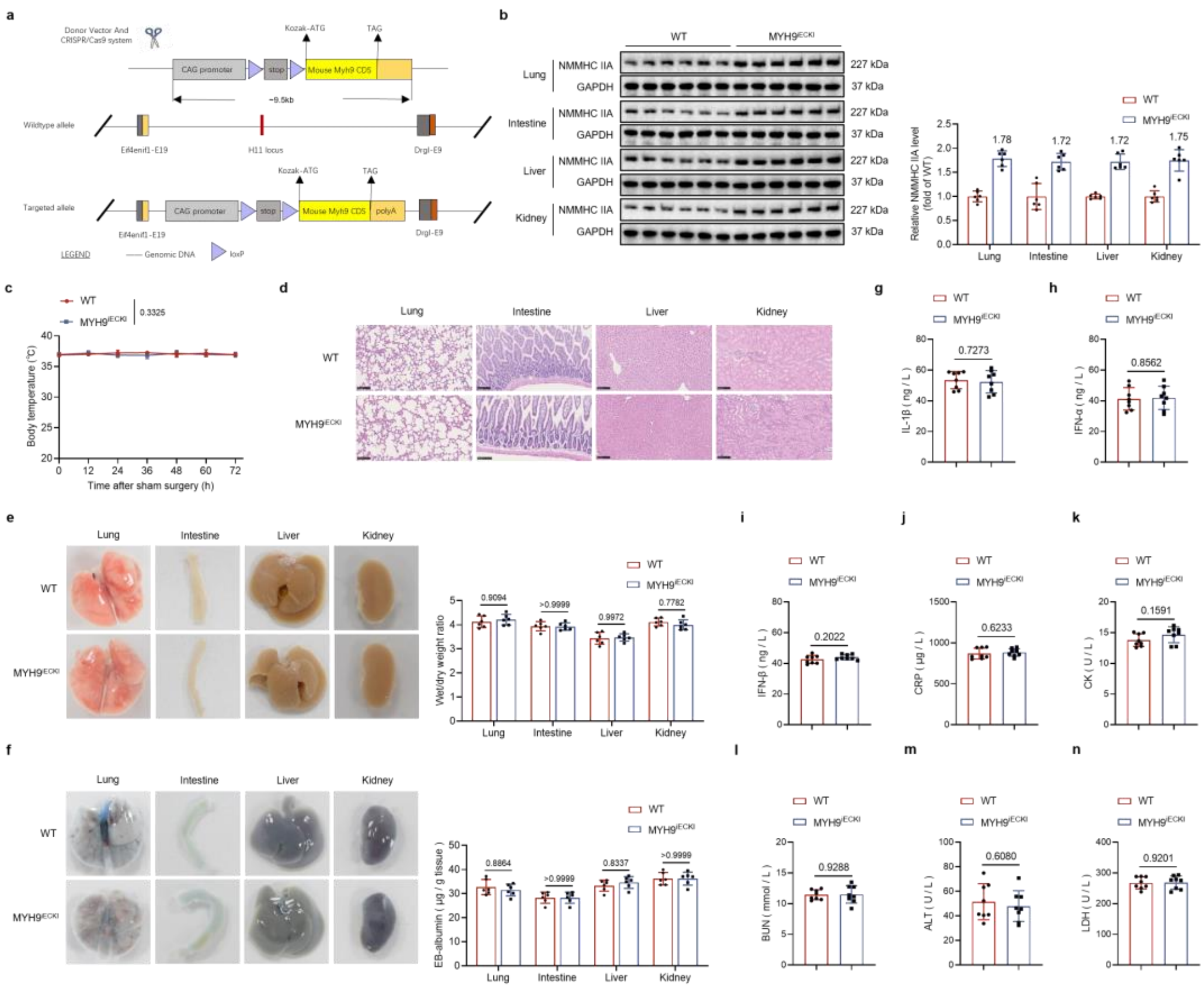

11. Single-cell RNA-seq analysis of lung ECs

The scRNA-seq lung dataset is a major mechanistic anchor, but analysis of endothelial subclusters (arterial, venous, capillary, lymphatic) and their differential response to USP28 deletion is limited; more granular analysis of EC heterogeneity and necroptosis/IFN-I signatures across subtypes would strengthen the mechanistic claims. Methods for quality control, doublet removal, integration, and differential expression in scRNA-seq (e.g., thresholds for mitochondrial reads, minimum genes, batch correction) are not fully detailed and should be expanded, with appropriate statistics and multiple-testing correction clearly stated.

Response: We thank the reviewer for raising an important point regarding both the depth of endothelial heterogeneity analysis and the completeness of the scRNA-seq methodology. To provide a more granular view of endothelial heterogeneity, we re-clustered the endothelial cell population from the lung scRNA-seq dataset and identified distinct subtypes, including arterial, venous, aerocyte capillary (aCAP), general capillary (gCAP), and lymphatic endothelial cells based on established marker genes. We then assessed IFN-I–related and necroptosis-associated transcriptional signatures across these endothelial subclusters. Notably, arterial, venous, aCAP, and gCAP endothelial cells displayed a broadly consistent enrichment pattern in response to USP28 deletion, whereas lymphatic endothelial cells did not show significant changes **(updated Supplementary Fig. 5)**. These results suggest that the effects of USP28 loss on IFN-I signaling and necroptosis are primarily manifested in blood vascular endothelial subsets rather than uniformly across all endothelial cell types, highlighting the functional heterogeneity of endothelial populations in the context of sepsis. We have included these analyses in the revised Supplementary Information.

In parallel, we have expanded the Methods section to provide a more detailed description of the scRNA-seq data processing and analysis pipeline. Specifically, we now include explicit criteria for quality control (gene number thresholds and mitochondrial content), doublet removal using DoubletFinder, batch correction and data integration using Harmony, clustering parameters, and statistical methods for differential expression analysis, including multiple-testing correction **(Pages 28–29, Lines 624–639)**. We believe that these additions improve the transparency and reproducibility of our single-cell analysis.

updated Supplementary Fig. 5

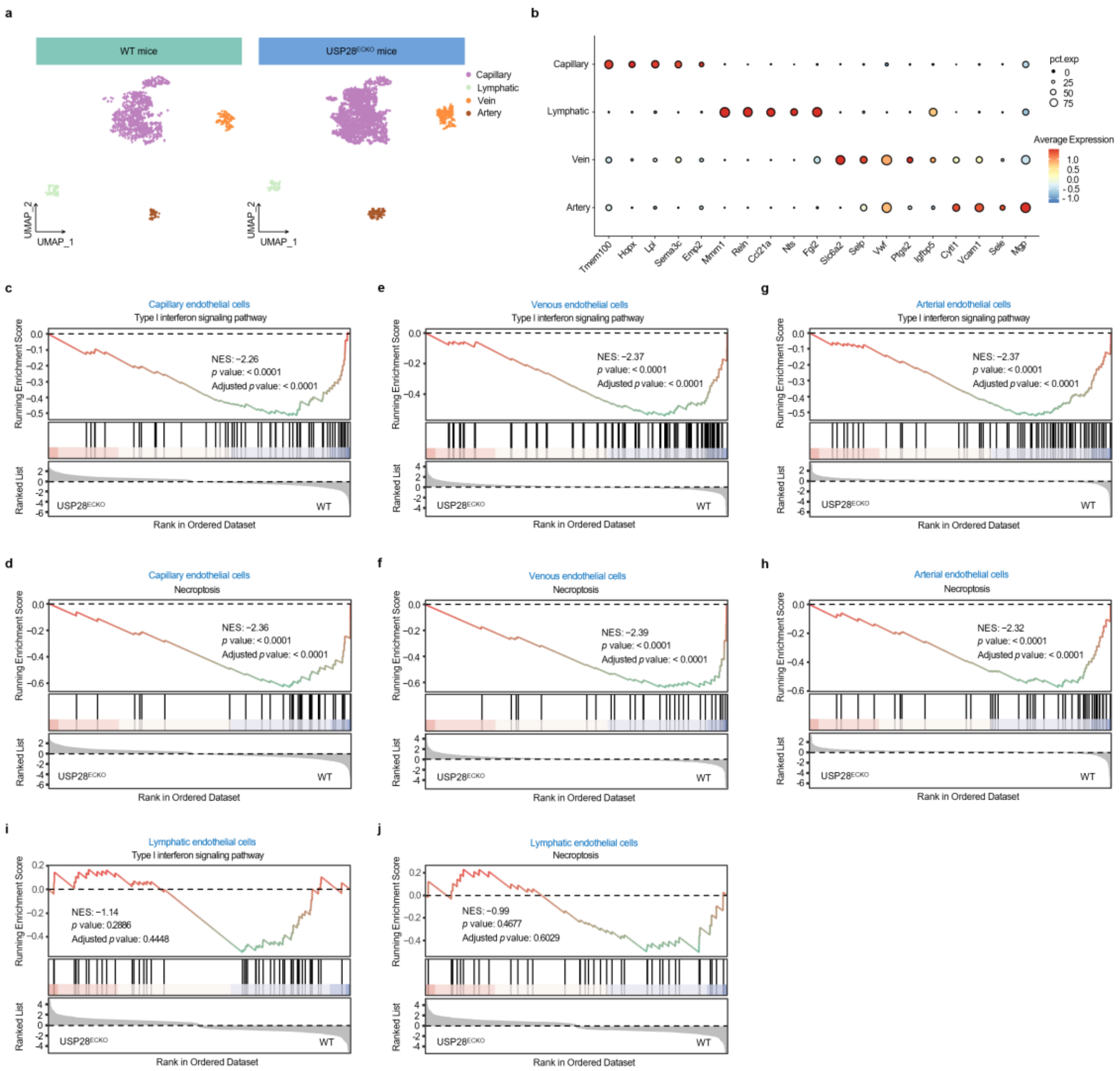

12. Data presentation and quantification

Several key figures rely on representative histology or immunofluorescence with limited quantitative data in the main text; numerical data (e.g., necroptosis markers in lung ECs, vascular leakage, wet/dry ratios) should be fully plotted with sample sizes, effect sizes, and P values clearly indicated, ideally in the main figures or expanded data. For many western blots and co-IP experiments, quantification (densitometry) is not consistently shown, and the number of biological replicates is sometimes unclear; the authors should standardize reporting of n, independent experiments, and statistical tests throughout.

Response: We appreciate the reviewer's request for clarification on the completeness and consistency of data presentation and quantification. We have carefully revised the manuscript to improve both the clarity and rigor of data reporting. Specifically, quantitative data corresponding to key histological and immunofluorescence observations, including necroptosis-related markers in lung endothelial cells, vascular leakage, and wet/dry ratios, have now been fully plotted with individual data points,

sample sizes, and statistical annotations in the revised figures and/or Supplementary Information. Exact *P* values are now explicitly indicated to facilitate transparent interpretation of the results. In addition, for western blot and co-immunoprecipitation experiments, we have standardized the presentation by including densitometric quantification where appropriate and clearly specifying the number of biological replicates. The definition of “n” has been consistently clarified in the Method section **(Page 34, Lines 751–752)**, distinguishing independent animals, independent cell cultures, and patient samples as applicable. We have also unified the statistical reporting throughout the manuscript. All data are now presented as mean ± SD, and statistical analyses were performed using two-tailed Student’s t-test, one- or two-way ANOVA with Tukey post hoc test, or Pearson’s correlation analysis as appropriate. Exact *P* values are provided in the figures, and all underlying numerical data have been made available in the Source Data file. These changes have been consistently incorporated into all figures and figure legends in the revised manuscript.

13. Pharmacological tool AZ-1

The selectivity profile of AZ-1 for USP28 versus other USPs and deubiquitinases is not discussed; the authors should either provide or cite biochemical specificity data and acknowledge potential off-target effects that might contribute to the observed phenotypes.

If feasible, using an independent USP28-targeting modality (e.g., a structurally distinct inhibitor or a genetic knockdown approach in adult mice) would help exclude AZ-1–specific artifacts; otherwise, this limitation should be clearly stated.

Response: We thank the reviewer for raising these critical questions regarding the specificity of the pharmacological inhibitor AZ-1 and the potential contribution of off-target effects. To address this concern, we took advantage of endothelial-specific USP28 knockout mice to determine whether the protective effects of AZ-1 are dependent on USP28 in vivo. Consistent with our original findings, administration of AZ-1 at 1 h after CLP significantly improved survival, reduced pro-inflammatory cytokine levels, and alleviated multi-organ injury in septic mice. Notably, these protective effects were largely abolished in endothelial-specific USP28-deficient mice, indicating that the in vivo efficacy of AZ-1 is critically dependent on endothelial USP28 **(updated Supplementary Fig. 3)**. These results provide genetic evidence that the observed effects of AZ-1 are not merely attributable to non-specific inhibition of other deubiquitinases, but are predominantly mediated through USP28.

14. Ethical, reporting, and ARRIVE considerations

The animal experiments section lacks some important details (randomization, allocation concealment, a priori power calculations, humane endpoints) that are

increasingly expected for high-impact journals; these details should be added and aligned with current ARRIVE guidelines.
For human subjects, the consent process and handling of severely ill or incapacitated patients (e.g., surrogate consent) should be clarified, and the registration of the cohort or biobank, if any, should be indicated.

Response: We appreciate the reviewer's request for clarification regarding compliance with ARRIVE guidelines and ethical reporting standards for both animal and human studies. We have revised the manuscript to provide additional details and improve transparency in these aspects. For animal experiments, we have expanded the Methods section to include information on experimental design and reporting in line with current ARRIVE recommendations **(Page 19, Lines 420–425)**. Specifically, we now state that mice were randomly assigned to experimental groups and that sample sizes were determined based on previous studies and pilot experiments **(doi: 10.1038/s41418-020-0524-1)**. Due to the use of genetically modified mouse models, investigators were not blinded to group allocation; however, data analysis was performed in a blinded manner where feasible to minimize bias. In addition, humane endpoints have now been explicitly defined, and mice were euthanized if they exhibited severe hypothermia, inability to access food or water, or moribund condition. For human subjects, we have clarified the consent procedure to better reflect clinical practice in critically ill patients. Written informed consent was obtained from participants or their legally authorized representatives prior to enrollment. We have also refined the description of sample collection and ethical compliance to ensure consistency with the Declaration of Helsinki and institutional approval requirements **(Page 18, Lines 397–403)**. These revisions improve the clarity, completeness, and compliance of our study with current ethical and reporting standards.

15. Writing, consistency, and minor issues
There are numerous typographical and formatting issues (missing hyphens, inconsistent spacing, ambiguous symbols, occasional apparent word omissions) that impede clarity and should be carefully corrected by thorough language editing.
Some statements in the Discussion seem internally inconsistent with earlier sections (e.g., one sentence suggests USP28 "inhibits sepsis progression" while the overall conclusion is that USP28 is detrimental), and the final paragraphs should be revised to maintain logical consistency and avoid contradictory phrasing.

Response: We apologize for the typographical and formatting issues present in the original manuscript and appreciate the reviewer's careful reading. The manuscript has now undergone thorough language editing to correct inconsistencies in spacing, hyphenation, symbols, and phrasing throughout. In addition, we have carefully revised

the Discussion to ensure logical consistency, particularly with respect to the role of USP28 in sepsis **(Page 18, Lines 390–391)**.

Reviewer #3 (Remarks to the Author):

Thank you for the invitation to review the manuscript. The authors propose a mechanism wherein endothelial USP28 stabilizes NMMHC IIA to promote IFNAR1 trafficking and necroptosis, thereby driving sepsis-induced multi-organ injury. The study presents a substantial amount of work, including clinical correlation, genetic mouse models, and molecular biology assays. However, despite the potential novelty of the proposed USP28-NMMHC IIA axis, I have significant concerns regarding the experimental design, the quality of the data, and the strength of the evidence provided. The current findings are insufficient to support the major conclusions drawn. While the hypothesis is interesting, the study in its current form suffers from fundamental design flaws and a lack of robust evidence. Therefore, I cannot recommend its publication.

Response: We sincerely apologize for the concerns raised regarding the experimental design, data quality, and strength of evidence in our original submission, and we greatly appreciate the reviewer's critical and constructive evaluation. We have carefully considered these comments and undertaken extensive revisions to address the issues raised. In the revised manuscript, we have performed additional experiments, incorporated new data, and refined our analyses to strengthen both the mechanistic depth and the rigor of our conclusions. We have also clarified the study design and improved data presentation throughout the manuscript. We believe that these substantial revisions have significantly improved the quality and robustness of the study, and we hope that the revised version adequately addresses the reviewer's concerns and will now be suitable for reconsideration.

Major concerns:

1. Organ Heterogeneity and Sampling Bias (Figure 1) While the IHC results in Figure 1H suggest ubiquitous expression of USP28 across the lung, intestine, liver, and kidney, the co-localization analysis in Figure 1I is restrictively performed only on pulmonary endothelial cells. Why were other cell types and organs excluded? Given that sepsis-induced dysfunction follows a distinct temporal sequence across different organs, it is scientifically precarious to assume the pulmonary expression pattern represents the systemic condition. Preliminary verification of USP28 in other cell types is required. Furthermore, the immunofluorescence (IF) images provided have an unacceptably small field of view, which introduces significant selection bias. Larger fields of view are necessary to ensure data representativeness.

Response: We appreciate the reviewer's thoughtful comments regarding organ

heterogeneity, cell-type specificity, and potential sampling bias in Figure 1. To better define the cellular distribution of USP28, we have now performed multiplex immunofluorescence staining in lung tissue, including markers for endothelial cells (CD31), macrophages (F4/80), alveolar epithelial cells (SP-C), and smooth muscle/myofibroblasts (α-SMA), together with USP28. These analyses demonstrate that, although USP28 is detectable in multiple cell types, its upregulation following CLP is most prominently observed in endothelial cells, supporting a preferential induction of USP28 in this compartment under septic conditions **(updated Supplementary Fig. 1)**.

updated Supplementary Fig. 1

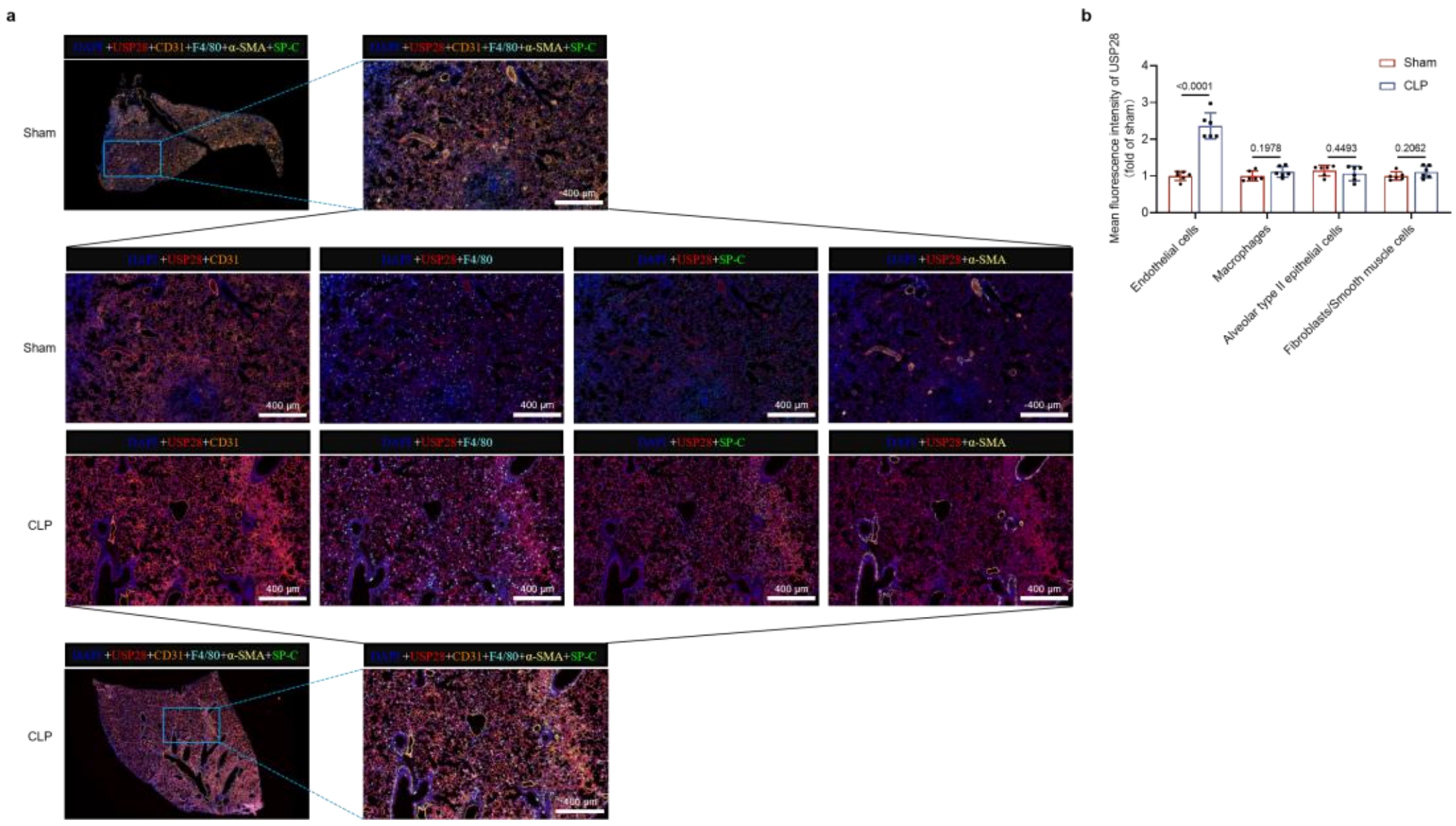

With respect to organ heterogeneity, we would like to clarify that multi-organ expression of USP28 was already evaluated in the original manuscript by immunohistochemistry **(updated Fig. 1k, l)**, which showed increased USP28 expression in the lung, intestine, liver, and kidney after CLP. In our initial analysis, we focused the time-course immunofluorescence experiments on lung tissue, as USP28 upregulation was most pronounced in the lung. To further strengthen the generalizability of our findings, we have now extended our analyses and confirmed that USP28 is similarly upregulated in vascular endothelial cells in the intestine, liver, and kidney at early time points after CLP, although the magnitude of induction remains highest in the lung **(updated Fig. 1m–q)**.

We chose to focus our subsequent mechanistic studies on pulmonary endothelial cells for several reasons. First, our study was initially motivated by the identification of USP28 in circulating endothelial cells (CECs) from patients with sepsis, indicating a direct link to endothelial dysfunction. Second, the lung contains a dense vascular

network and is one of the earliest and most severely affected organs in sepsis, making it a suitable and widely accepted model for studying endothelial injury. Third, our data show that USP28 induction is most robust in pulmonary tissue, further supporting this focus **(updated Fig. 1m–q)**.

updated Fig. 1m–q

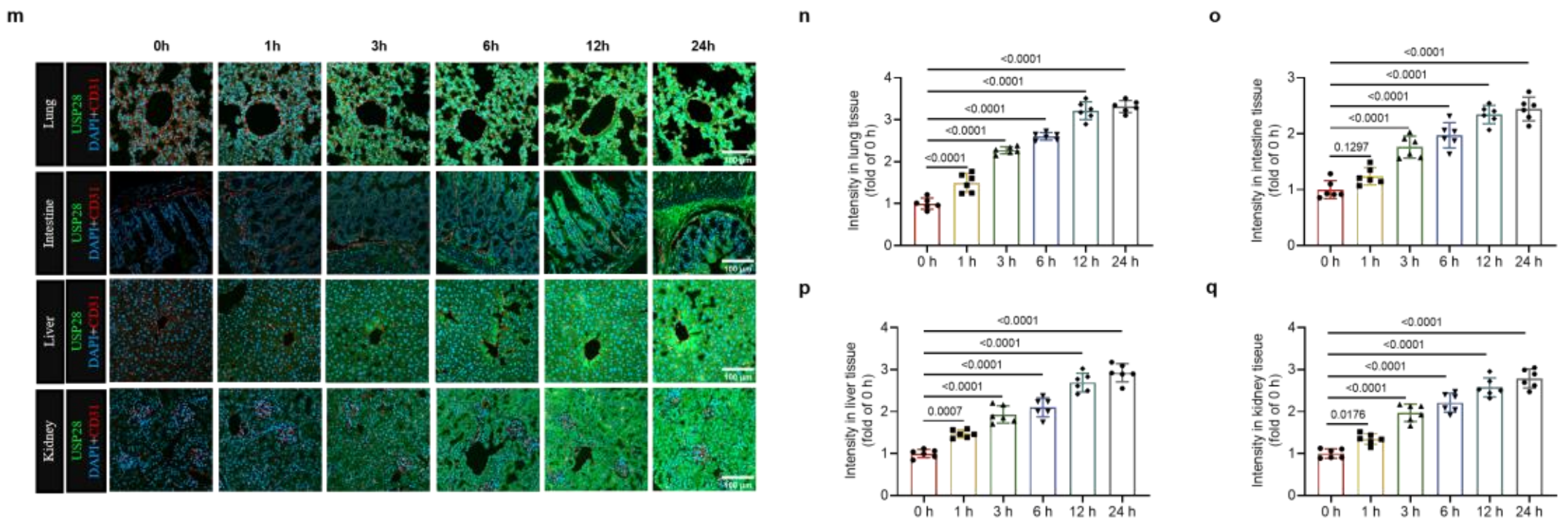

Regarding the concern about the limited field of view in the immunofluorescence images, we agree that this could introduce potential bias. We have therefore replaced the images with larger fields of view and provided corresponding low-magnification images to ensure better representativeness and transparency **(updated Fig. 1m; updated Fig. 3h, i; updated Fig. 4n, o; updated Fig. 7n, o)**.

2. Inadequate Validation of Genetic Models (Figure S1 & S3)
Relying solely on IF (Figure S1B) to validate endothelial-specific knockout or overexpression is methodologically insufficient. The authors must isolate primary pulmonary endothelial cells from the respective mouse models and confirm the efficiency via Western blotting and qPCR. Additionally, the overexpression efficiency shown in Figure S3B is underwhelming (less than 2-fold increase). This raises serious concerns regarding the titer of the AAV virus or the efficacy of the injection protocol, questioning the validity of the subsequent phenotypic observations.

Response: We sincerely appreciate the reviewer's critical comments regarding the validation of our genetic models and the level of overexpression achieved. We agree that relying solely on immunofluorescence is not sufficiently rigorous to validate endothelial-specific knockout or overexpression. To address this, we have now performed validation experiments by isolating primary vascular endothelial cells from the lung, intestine, liver, and kidney of the respective mouse models. The efficiency of all genetically modified mouse models used in this study, including endothelial-specific knockout and overexpression models, was re-assessed by western blotting in these primary endothelial cells. These new data provide more direct and quantitative validation of the genetic manipulations than the original immunofluorescence-based assessment **(updated Fig. 2b; updated Supplementary Fig. 3b; updated**

Supplementary Fig. 4b; updated Supplementary Fig. 8b; updated Supplementary Fig. 11b). With respect to the level of USP28 overexpression, we have re-evaluated its expression and observed a ~1.7–2.3-fold increase in endothelial cells across different organs **(updated Supplementary Fig. 4b)**. While this level of increase may appear modest, we note that USP28 is a relatively large protein (~135 kDa) with a transcript length of approximately 3,252 bp, which approaches the packaging capacity limit of AAV9-Tie1 vectors. This technical constraint likely limits the achievable overexpression efficiency, rather than reflecting insufficient viral titer or injection protocol. Importantly, despite this moderate level of overexpression, our functional data consistently demonstrate that USP28 overexpression is sufficient to exacerbate endothelial dysfunction and multi-organ injury in sepsis models **(updated Supplementary Fig. 4)**.

3. Poor Histological Quality (Lung H&E)
The quality of the lung H&E staining images is substandard and requires immediate replacement. Crucially, the control group exhibits evident structural damage and alveolar collapse, which is biologically implausible for healthy tissue. This suggests either improper sample processing or pre-existing pathology in the control mice.

Response: We thank the reviewer for this important comment regarding the histological quality of the lung H&E staining. We agree that the originally presented images were not of optimal quality, and we have now replaced them with higher-resolution images with improved contrast and standardized presentation. These updated images are provided in the revised manuscript **(updated Fig. 2e; updated Fig. 4c; updated Fig. 7c; updated Supplementary Fig. 2c; updated Supplementary Fig. 3d; updated Supplementary Fig. 4e; updated Supplementary Fig. 8d; updated Supplementary Fig. 10d)**. Regarding the reviewer's concern about the apparent structural alterations in the control group, we would like to clarify our tissue processing procedure. In this study, animals were subjected to systemic cardiac perfusion to ensure consistent and simultaneous fixation across multiple organs (lung, liver, kidney, and intestine), as our primary objective was to evaluate multi-organ injury under a unified experimental framework. Unlike lung-specific studies that employ tracheal instillation fixation to maintain alveolar inflation, cardiac perfusion fixation may lead to partial alveolar collapse or subtle structural distortion due to the extremely thin and delicate nature of alveolar walls. This phenomenon has been reported in previous studies using similar systemic cardiac perfusion protocols **(doi: 10.1038/s41467-024-51209-9; doi: 10.1016/j.apsb.2025.10.020; doi: 10.1021/jacs.6c00209; doi: 10.1038/s41392-026-02592-w; doi: 10.1126/sciadv.adt7589； doi: 10.1172/JCI159400)**. Importantly, histological evaluation (injury score) in this study was not based on alveolar

morphology, but on a comprehensive assessment of inflammatory cell infiltration, interstitial edema, and exudation, which are well-established indicators of lung injury in sepsis models.

4. Anomalous Cytokine Profiles (Figure 2 & S2)
The cytokine data presented is highly questionable for several reasons:
Timing: Measuring cytokines at 24 hours post-CLP is suboptimal, as levels of IL-1β, IL-6, and TNF-α typically peak at 6–12 hours and decline significantly by 24 hours.
Magnitude Discrepancy: Physiologically, serum IL-6 levels (typically ng/mL range) should exceed IL-1β and TNF-α (pg/mL range) in sepsis, yet the reported data contradicts this well-established pattern.
Baseline Levels: The baseline TNF-α level in control mice is reported as ~500 pg/mL (500 ng/L), which is aberrantly high (normal range <30 pg/mL).
Incomplete Data: Why are cytokine and CK data omitted in subsequent figures? This selective reporting undermines the credibility of the study.

Response: We thank the reviewer for the careful evaluation of the cytokine data and for raising these important concerns. We have carefully re-examined and strengthened this part of the study as detailed below.
(1) Timing of cytokine measurement
We agree that certain cytokines, such as IL-6 and TNF-α, may exhibit early peaks following CLP (e.g., 6–12 h). However, in this study, we selected the 24 h time point to align with our primary objective of assessing multi-organ injury, as this is when organ injury–related biochemical markers (e.g., CK, ALT, BUN, LDH) become more pronounced. Indeed, previous studies have shown that organ damage and corresponding biochemical alterations are not fully evident at earlier time points following CLP **(doi: 10.1038/s41418-020-0524-1)**. Importantly, cytokine dynamics in CLP models are highly context-dependent, and several studies have reported that inflammatory mediators, including IL-1β, IL-6, and TNF-α, remain elevated or even increase at later time points (~22–24 h) compared to earlier stages **(doi: 10.1038/s41418-020-0524-1; doi: 10.1038/s41392-026-02592-w)**. Therefore, the 24 h time point represents a widely used and biologically relevant time window for integrated assessment of inflammation and organ injury in sepsis research **(doi: 10.1038/s41392-025-02457-8; doi: 10.1016/j.cmet.2026.01.005; doi: 10.1002/advs.202506209)**.
(2) Magnitude discrepancy of cytokine levels
We appreciate the reviewer’s concern regarding the relative magnitude of cytokines. We recognize that the initially reported values may not fully reflect the expected physiological hierarchy. Upon further investigation, we found that cytokine measurements can vary substantially depending on the assay kits used, including

differences in calibration standards, detection ranges, and antibody specificity. Consistent with this, considerable variability in reported cytokine concentrations has been documented across studies employing different ELISA platforms **(doi: 10.1172/JCI159400; doi: 10.1016/j.apsb.2024.03.025; doi: 10.1038/s41418-020-0524-1; doi: 10.1002/advs.202411652; doi: 10.1016/j.apsb.2025.10.020)**, highlighting the inherent variability in absolute quantification. To address this issue, we have re-measured IL-6 and TNF-α using new ELISA kits (IL-6: NeoBioscience, CAT#EMC004.96; TNF-α: NeoBioscience, CAT#EMC102a.96). The updated results now show a physiologically consistent pattern: IL-6 levels increased from ~40 ng/L in controls to ~2100 ng/L in CLP mice, while TNF-α increased from ~50 ng/L to ~200 ng/L **(updated Fig. 2i, j; updated Supplementary Fig. 2g, h)**, in line with established cytokine profiles in sepsis.

updated Fig. 2i, j

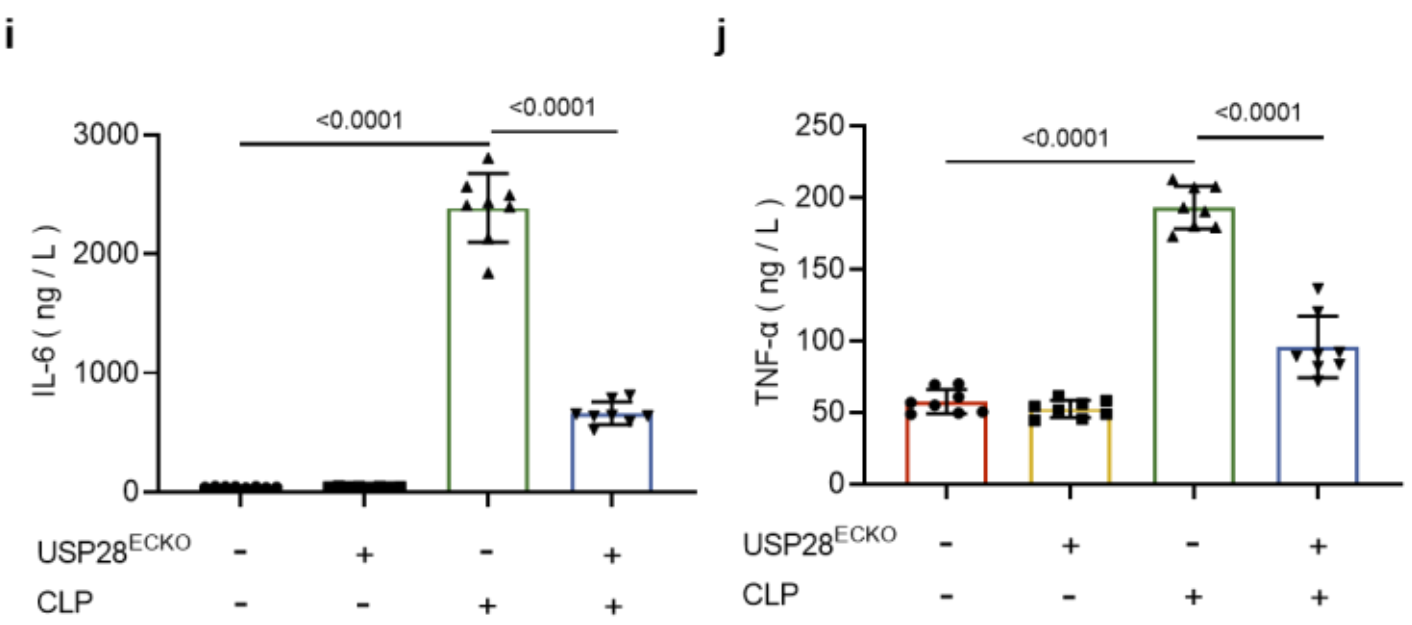

updated Supplementary Fig. 2g, h

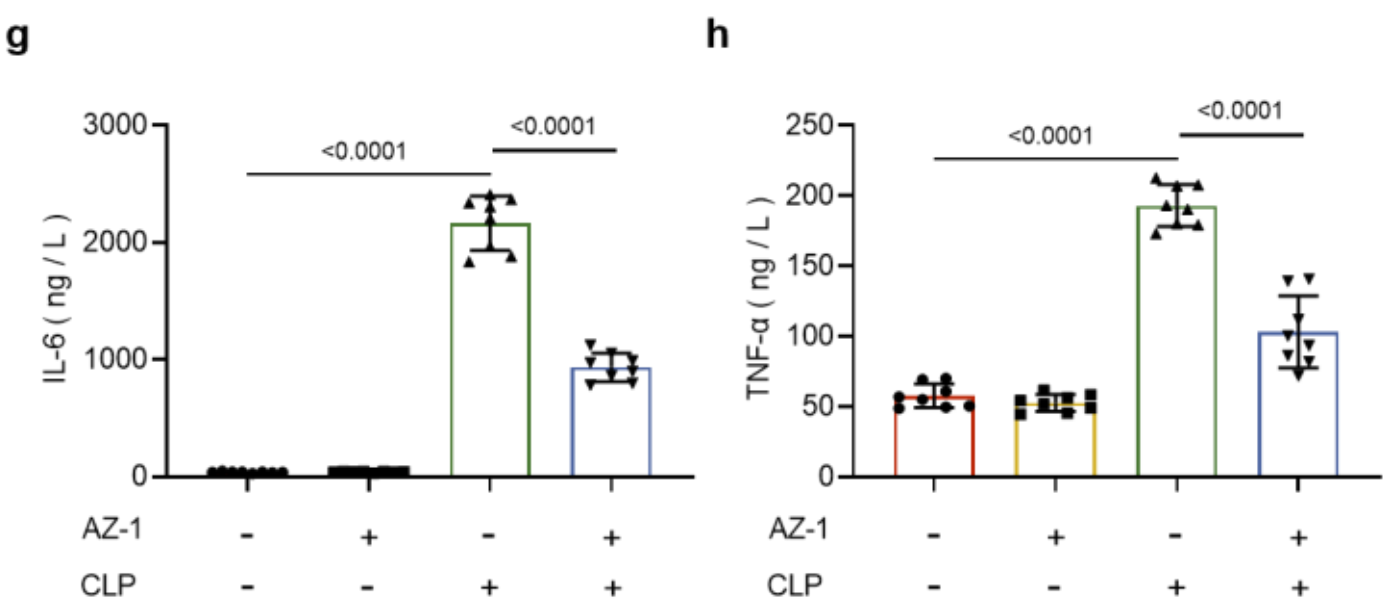

(3) Baseline TNF-α levels in controls

We agree that the initially reported baseline TNF-α levels were higher than expected. This discrepancy was also attributable to differences in assay systems. As described above, after re-evaluation using new ELISA kits, the baseline TNF-α levels in control mice are now within the expected physiological range (~50 ng/L), thereby resolving this issue.

(4) Incomplete reporting of cytokine and CK data

We appreciate the reviewer's concern regarding data completeness. In this study, our primary focus was on multi-organ injury and IFN-I signaling, and due to practical considerations, we prioritized a subset of biomarkers in subsequent experiments. Specifically, later analyses focused on IL-1β, IFN-α, IFN-β, CRP, CK, BUN, ALT, and LDH. To improve transparency and completeness, we have now included all previously omitted data, including IFN-α and CK measurements, in the revised manuscript **(updated Supplementary Fig. 3j, m; updated Supplementary Fig. 4i, l; updated Fig. 4g, j; updated Fig. 7g, j)**. These additions ensure a more comprehensive and consistent presentation of the biochemical and inflammatory profiles across experiments.

updated Supplementary Fig. 3j, m

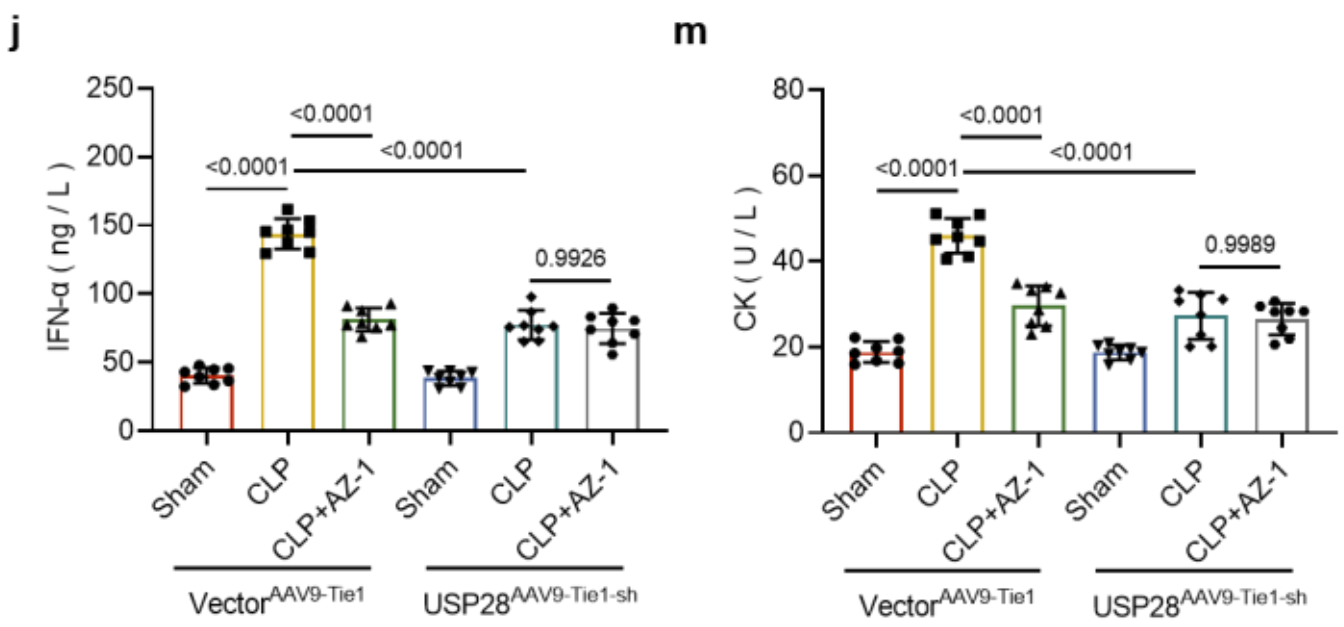

updated Supplementary Fig. 4i, l

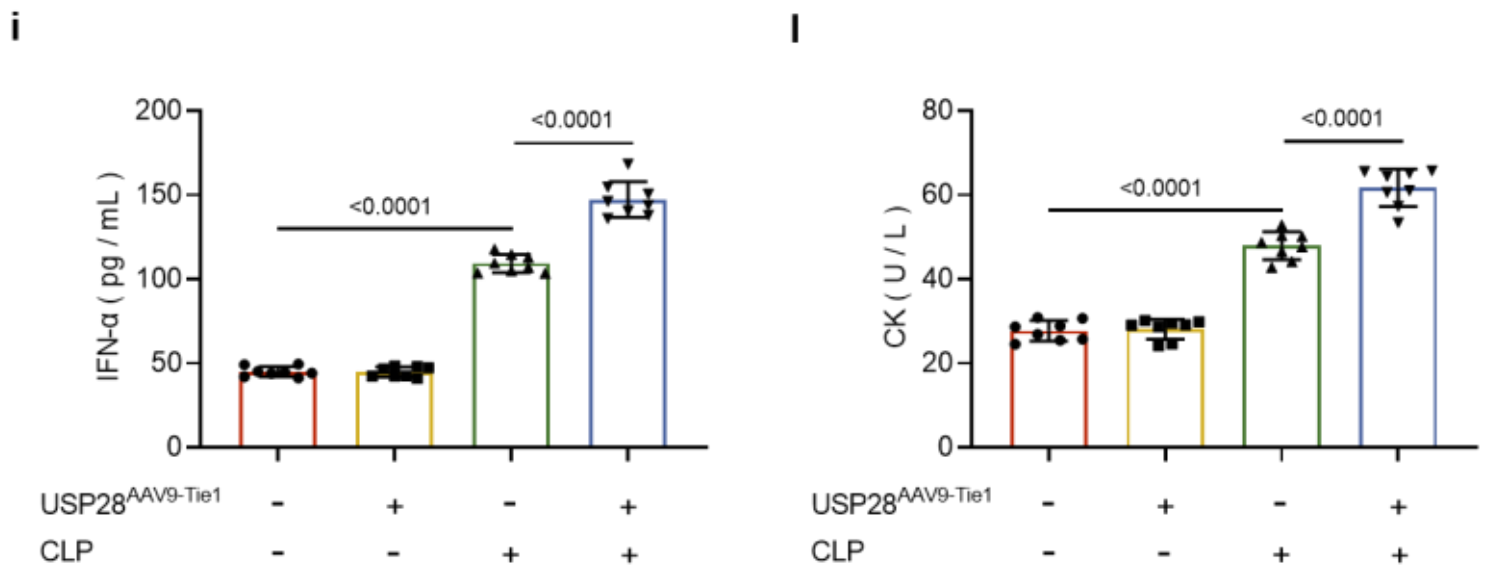

updated Fig. 4g, j

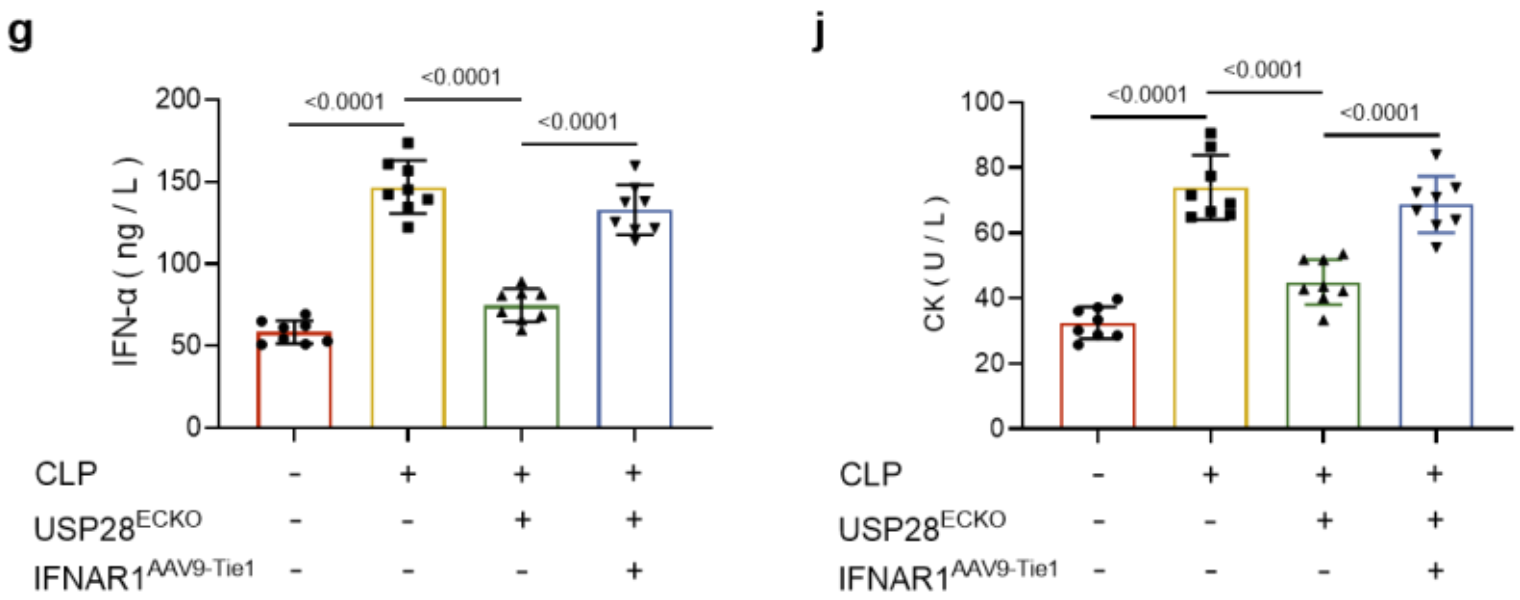

updated Fig. 7g, j

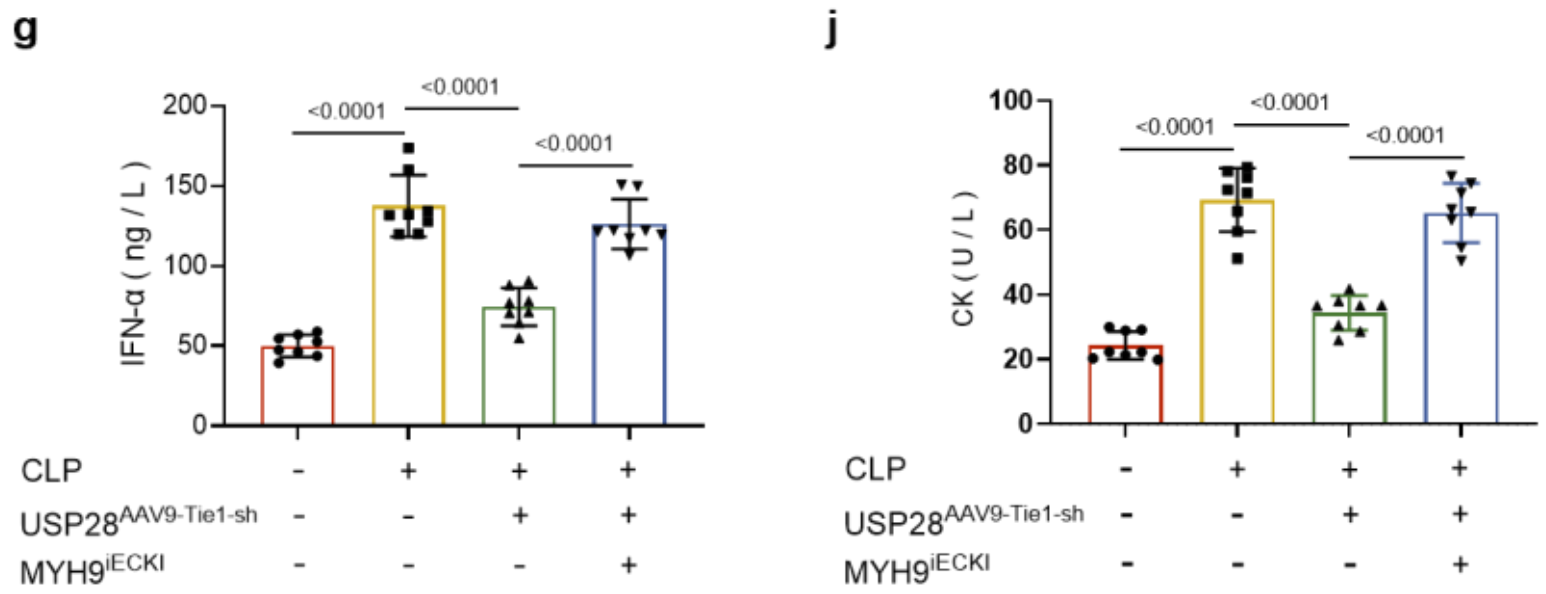

5. Flawed Drug Intervention Strategy

Administering drug intervention prior to CLP surgery violates Minimum Quality Threshold in Pre-Clinical Sepsis Studies (Mqtipss), which require post-insult treatment to mimic clinical reality. Furthermore, AZ-1 is a non-specific inhibitor that also targets USP25, a homolog expressed in endothelial cells (PMID: 40370563). The authors failed to rule out the off-target contribution of USP25 inhibition to the observed protective effects.

Response: We fully agree that prophylactic administration does not adequately reflect clinical scenarios, as highlighted by the Mqtipss guidelines. In the initial design, AZ-1 was administered prior to CLP primarily to evaluate the proof-of-concept role of USP28 in the early pathogenesis of sepsis-induced endothelial dysfunction, rather than to establish a clinically optimized therapeutic regimen. To address this important concern, we have now performed additional experiments using a post-insult treatment paradigm, in which AZ-1 was administered at 1 h after CLP surgery. Notably, delayed administration of AZ-1 still conferred robust protective effects, including improved survival, reduced systemic inflammatory responses, and alleviation of multi-organ injury **(updated Supplementary Fig. S3)**. These findings demonstrate that pharmacological inhibition of USP28 remains effective after sepsis onset, thereby strengthening the translational relevance of our study. We also acknowledge the reviewer's concern regarding the specificity of AZ-1, particularly its reported activity toward USP25. Given that AZ-1 is not a fully selective inhibitor, it is important to determine whether the observed protective effects can be attributed specifically to USP28 inhibition. To address this, we leveraged endothelial-specific USP28-deficient mice (*USP28*$^{\text{AAV9-Tie1-sh}}$) to functionally assess target dependence. Notably, AZ-1 treatment failed to confer additional protection in these mice, as reflected by the absence of further improvements in survival, inflammatory cytokine production, and multi-organ injury **(updated Supplementary Fig. S3)**. These findings indicate that the protective effects of AZ-1 are largely dependent on USP28, rather than arising from off-target inhibition of related deubiquitinases such as USP25.

updated Supplementary Fig 3

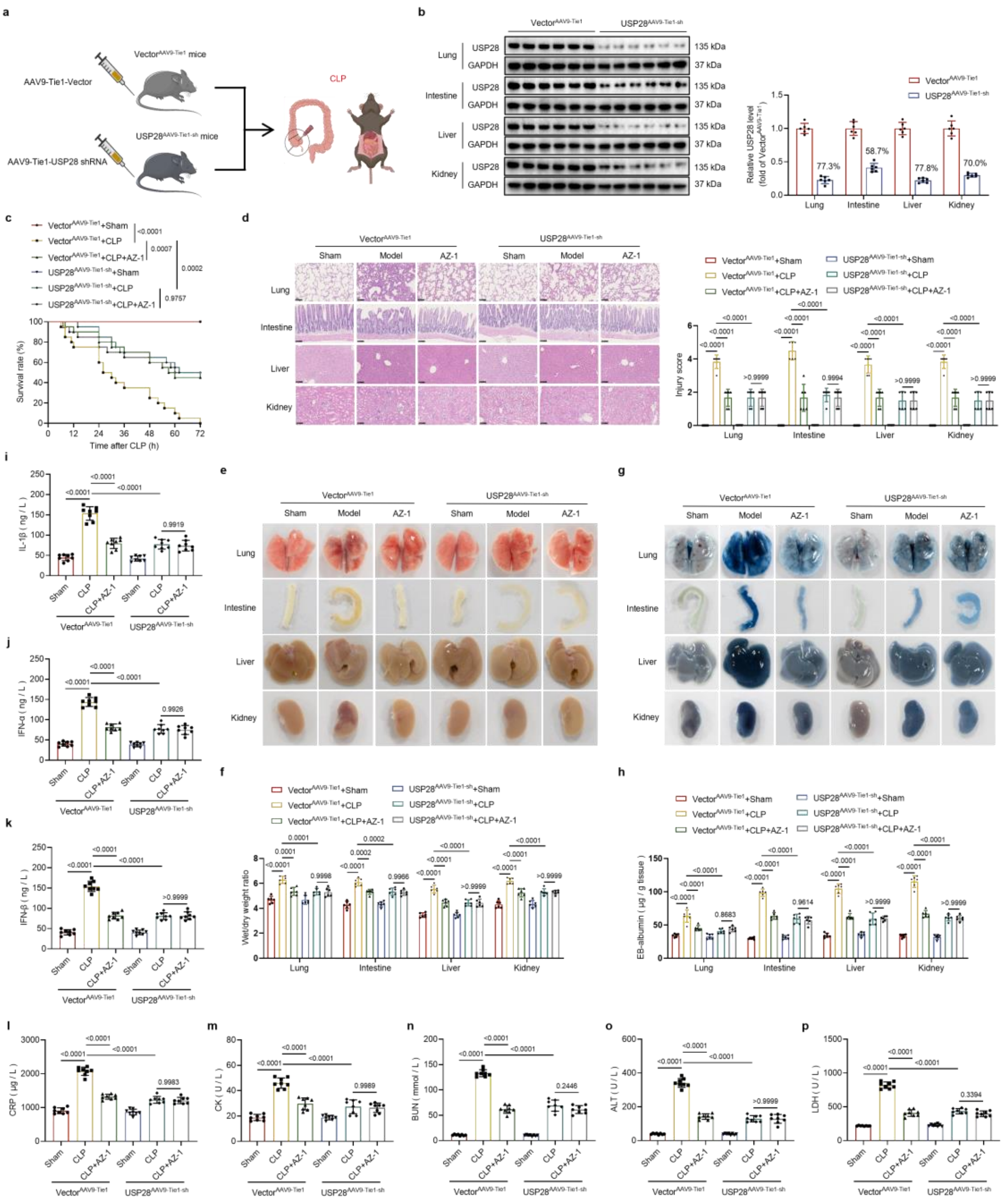

6. Limitations of AAV Tropism (Figure S3)

While AAV9-Tie1 effectively targets the lung and liver via tail vein injection, its transduction efficiency in the kidney and intestine is historically poor. The authors did not assess the overexpression efficiency in these organs. Therefore, attributing the systemic multi-organ dysfunction solely to endothelial USP28 overexpression is speculative without verifying target engagement in the kidney and gut.

Response: We sincerely appreciate the reviewer's insightful comment regarding the tissue tropism of AAV9-Tie1 and the potential variability of transduction efficiency

across organs. To directly address this concern, we have now performed systematic validation of target engagement at the endothelial cell level across multiple organs. Specifically, primary vascular endothelial cells were isolated from the lung, intestine, liver, and kidney of AAV9-Tie1–mediated USP28 overexpression mice, and USP28 expression was quantified by western blotting. The results demonstrate that endothelial USP28 expression was consistently increased across all examined organs, with overexpression efficiencies of approximately 2.27-fold in lung, 1.74-fold in intestine, 2.34-fold in liver, and 1.96-fold in kidney **(updated Supplementary Fig. 4b)**. These data provide direct evidence that AAV9-Tie1 achieves effective endothelial targeting not only in the lung and liver, but also in the intestine and kidney at the level of isolated endothelial cells. We agree that AAV9 tropism may vary across tissues; however, this level of increase is sufficient to drive consistent functional effects on endothelial dysfunction and multi-organ injury, supporting the biological relevance of the model. Moreover, to ensure the rigor and consistency of genetic model validation throughout the study, we similarly isolated primary vascular endothelial cells from the lung, intestine, liver, and kidney of all other genetically modified mouse models used in this work, and re-assessed their knockout or overexpression efficiency by western blotting **(updated Fig. 2b; updated Supplementary Fig. 3b; updated Supplementary Fig. 8b; updated Supplementary Fig. 11b)**.

7. Survival vs. Organ Damage Discrepancy (Figure S3C)
If endothelial USP28 overexpression exacerbates multi-organ damage, why does it fail to accelerate mortality in the survival analysis? This discrepancy suggests a "ceiling effect" caused by an overly severe CLP model. I recommend repeating this experiment using a milder CLP protocol (e.g., fewer punctures or smaller needle size) to determine if USP28 overexpression truly impacts survival.

Response: We thank the reviewer for this insightful comment regarding the apparent discrepancy between survival outcomes and organ injury severity, and we apologize for any confusion caused by insufficient methodological clarification and missing statistical annotations in the original version. We agree with the reviewer that a “ceiling effect” can occur under overly severe CLP conditions, limiting the sensitivity of survival as an endpoint. Indeed, based on our prior experience with the CLP model, we recognized this issue early and therefore employed different CLP severities for different experimental purposes. Specifically, while a more severe CLP protocol was used in experiments designed to induce robust and reproducible multi-organ injury, the survival analysis shown in Figure S3 was already performed using a milder CLP model with approximately 50 % cecal ligation, in order to avoid survival saturation. Under these moderate CLP conditions, endothelial-specific USP28 overexpression significantly

aggravated mortality (p = 0.0481), as shown in **updated Supplementary Fig. 4c**, demonstrating that USP28 overexpression does accelerate death when the survival endpoint is not masked by excessive surgical severity. The lack of statistical significance annotations in the original figures, including survival and body temperature curves, may have contributed to the reviewer's concern. In the revised manuscript, we have now added complete statistical annotations to all survival analyses and body temperature curves, and we have clarified the distinct CLP protocols used in the Methods section **(page 21, lines 460–462)**. In addition, we note that, even under this milder CLP protocol, body temperature measurements still exhibited an apparent "ceiling effect". This likely reflects the fact that profound hypothermia is tightly linked to imminent death in septic mice; mice reaching critically low body temperatures often succumb rapidly and are therefore no longer available for subsequent temperature measurements. As a result, body temperature can underestimate the full extent of pathological deterioration, as it is necessarily recorded only in surviving animals. Taken together, these revisions clarify that the observed survival effects are fully consistent with the organ injury data and that endothelial USP28 overexpression exacerbates sepsis outcomes when assessed under appropriate disease severity. We appreciate the reviewer's comment, which helped us improve the clarity and presentation of this important point.

8. Irrational Vector Control Data (Figure S3)
The "Vector-AAV9-Tie1 + CLP" group exhibits significantly milder tissue injury and lower inflammatory markers compared to the "WT + CLP" group in Figure 2 or the "CLP" group in Figure S2. It is biologically irrational for an empty vector to confer protection against sepsis. This inconsistency indicates serious issues with experimental reproducibility or batch effects.

Response: We thank the reviewer for this important comment and apologize for the confusion caused by insufficient clarification of the experimental conditions in the original manuscript. The apparent discrepancy arises from the use of different CLP severities across experiments, rather than any protective effect of the empty vector. Specifically, the experiments shown in Figure S3 (including the "Vector-AAV9-Tie1 + CLP" group) were performed using a milder CLP protocol (~50% cecal ligation) to avoid ceiling effects and allow adequate evaluation of survival and pathological outcomes. In contrast, the experiments presented in Figure 2 and Figure S2 were conducted using a more severe CLP protocol (~75% ligation) to induce robust and reproducible multi-organ injury. As a result, the reduced tissue injury and inflammatory responses observed in the "Vector-AAV9-Tie1 + CLP" group in Figure S3 **(now in updated Supplementary Fig. 4)** reflect the lower disease severity of the model, rather

than any protective effect of the control vector. We have now clearly indicated the distinct CLP protocols used in each experimental set in the Methods section **(page 21, lines 460–462)** to avoid further misunderstanding.

9. Lack of Biochemical Verification for Signaling (Figures 3, 4, 7, S4, S5)
Relying exclusively on immunofluorescence to demonstrate the impact of USP28 on IFN-I signaling is insufficient. The authors must provide Western blot evidence from isolated mouse pulmonary endothelial cells (in vivo) and HUVEC lysates (in vitro) to quantitatively validate these signaling changes.

Response: We agree that immunofluorescence alone is not sufficient to quantitatively assess IFN-I signaling. In response, we have now supplemented our study with comprehensive western blot analyses to validate the signaling changes at the protein level. Specifically, we performed western blotting using primary pulmonary endothelial cells isolated from mice (*in vivo*) as well as HUVEC lysates (*in vitro*) to assess the impact of USP28 on IFN-I signaling. These new data confirm the changes observed by immunofluorescence, thereby providing further quantitative validation. The corresponding results have been incorporated into the revised manuscript **(updated Supplementary Fig. 6; updated Supplementary Fig. 7f–o; updated Supplementary Fig. 9; updated Supplementary Fig. 10f–h; updated Supplementary Fig. 12)**.

updated Supplementary Fig. 6

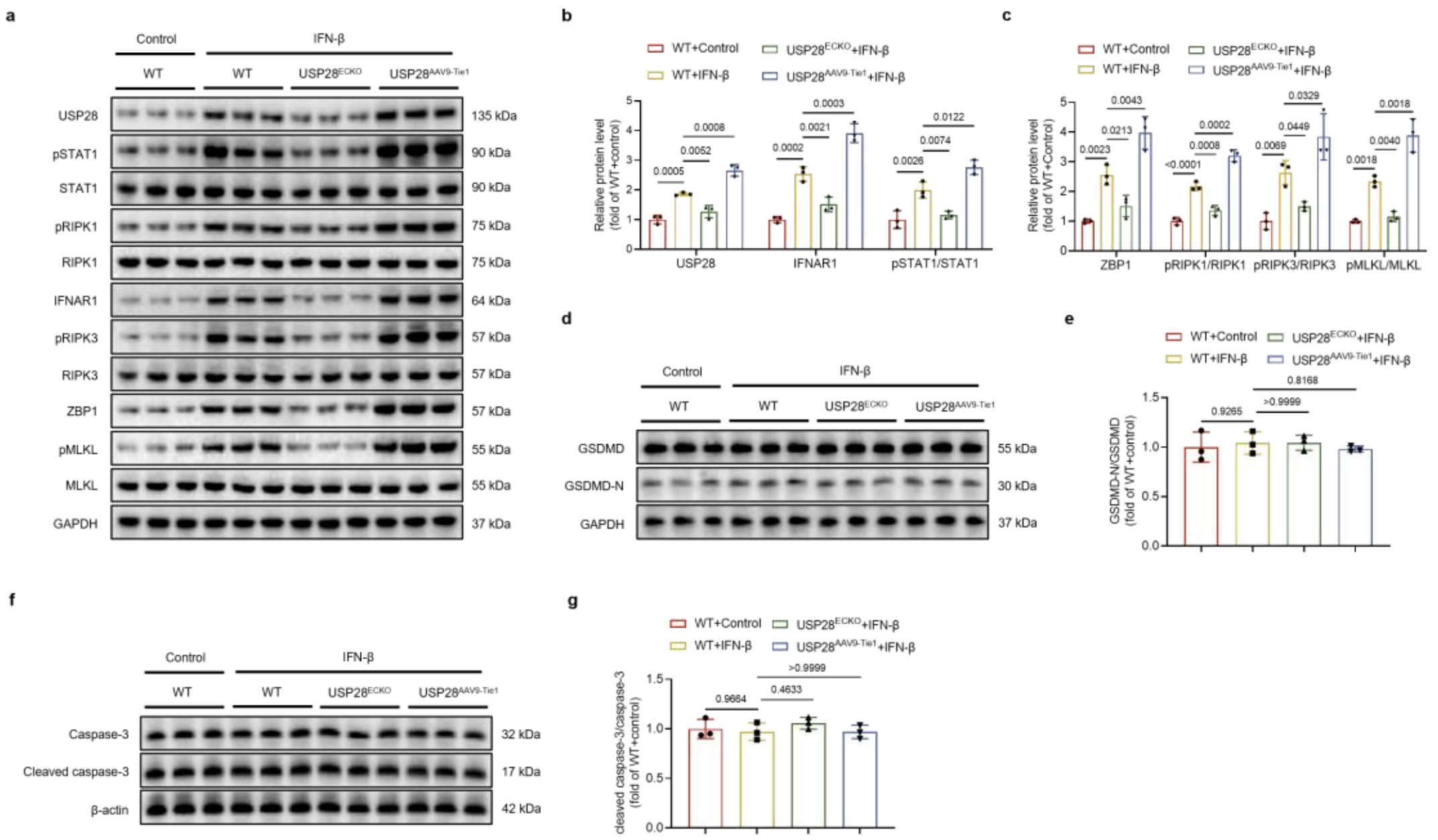

updated Supplementary Fig. 7f–o

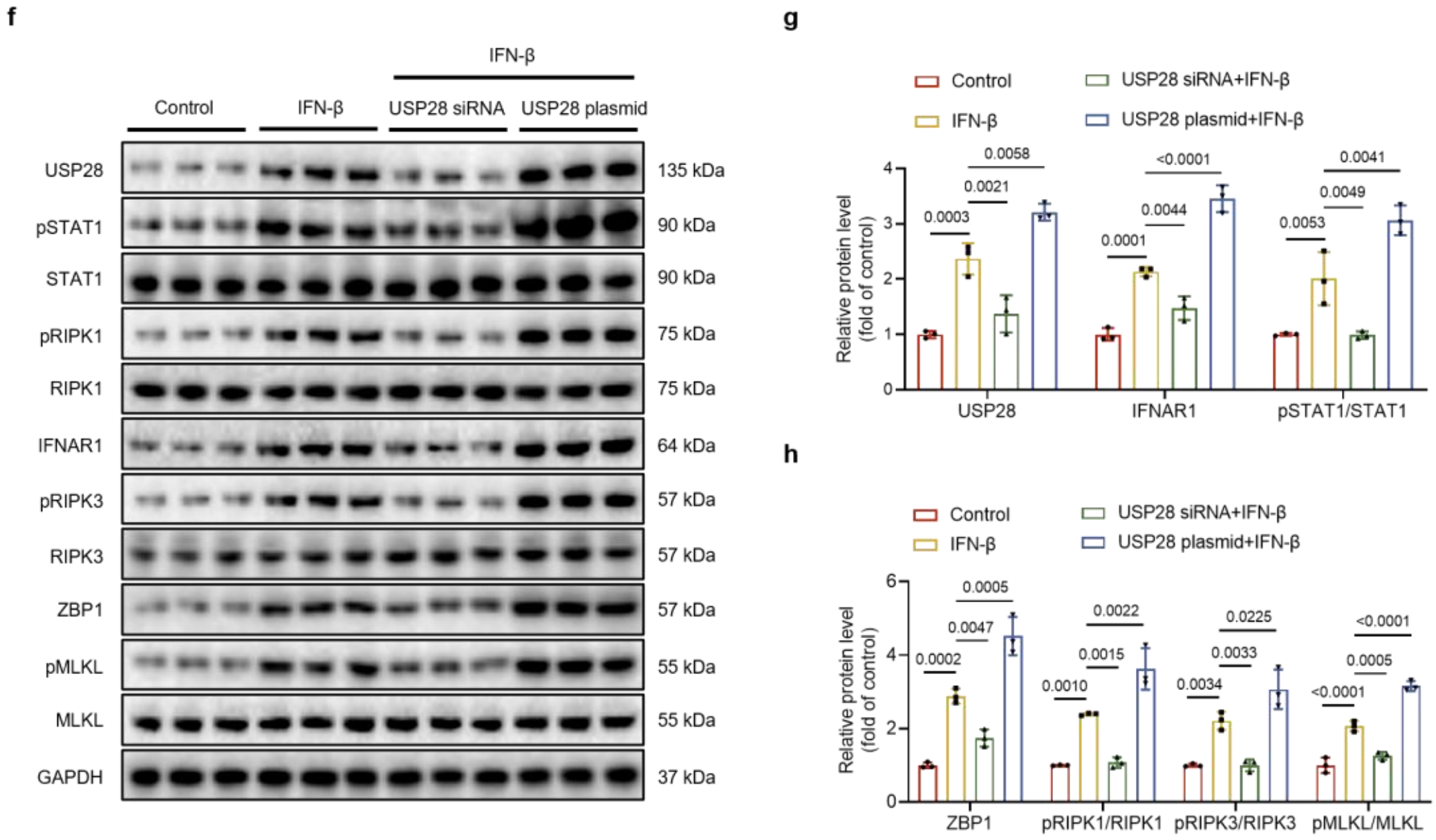

updated Supplementary Fig. 9

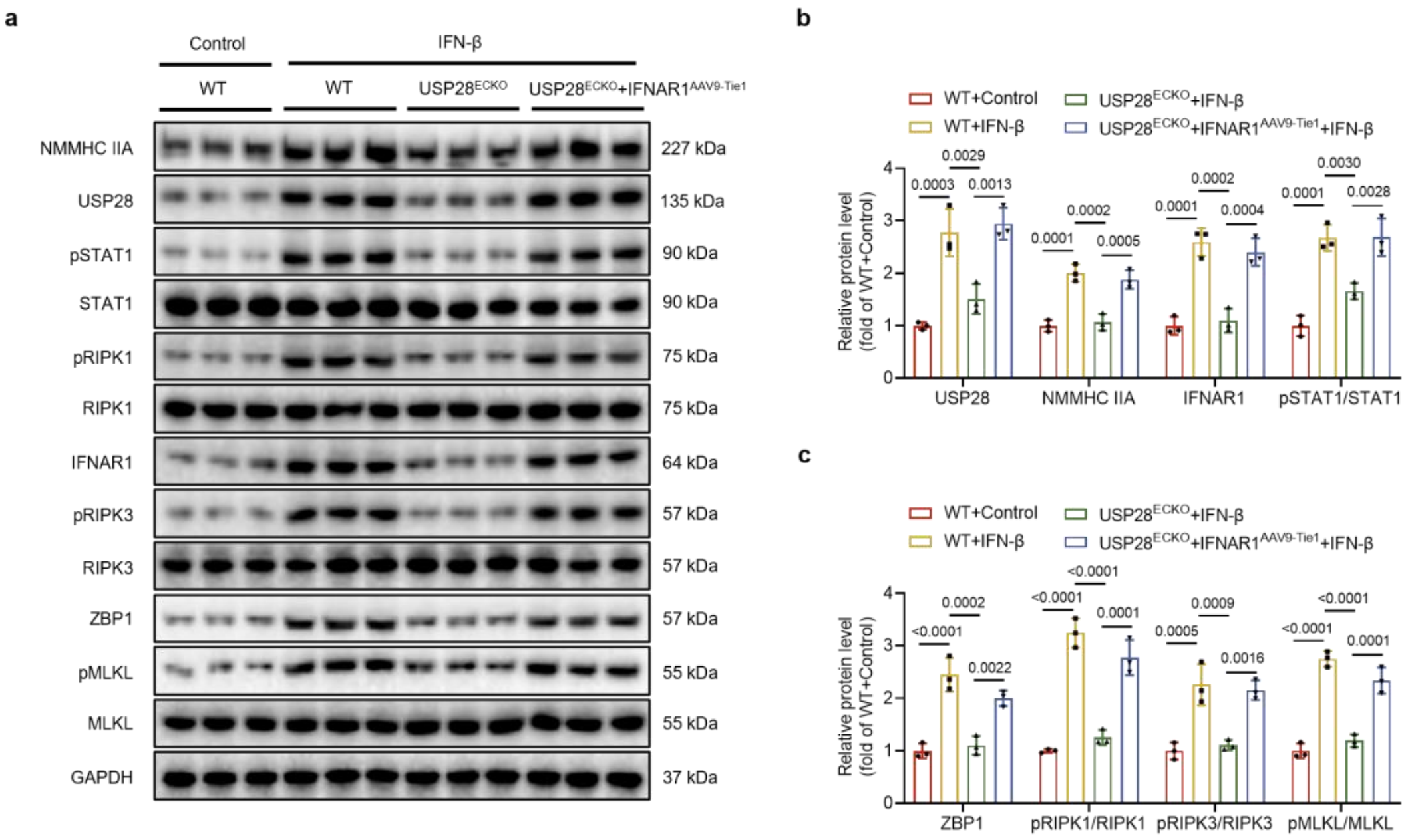

updated Supplementary Fig. 10f–h

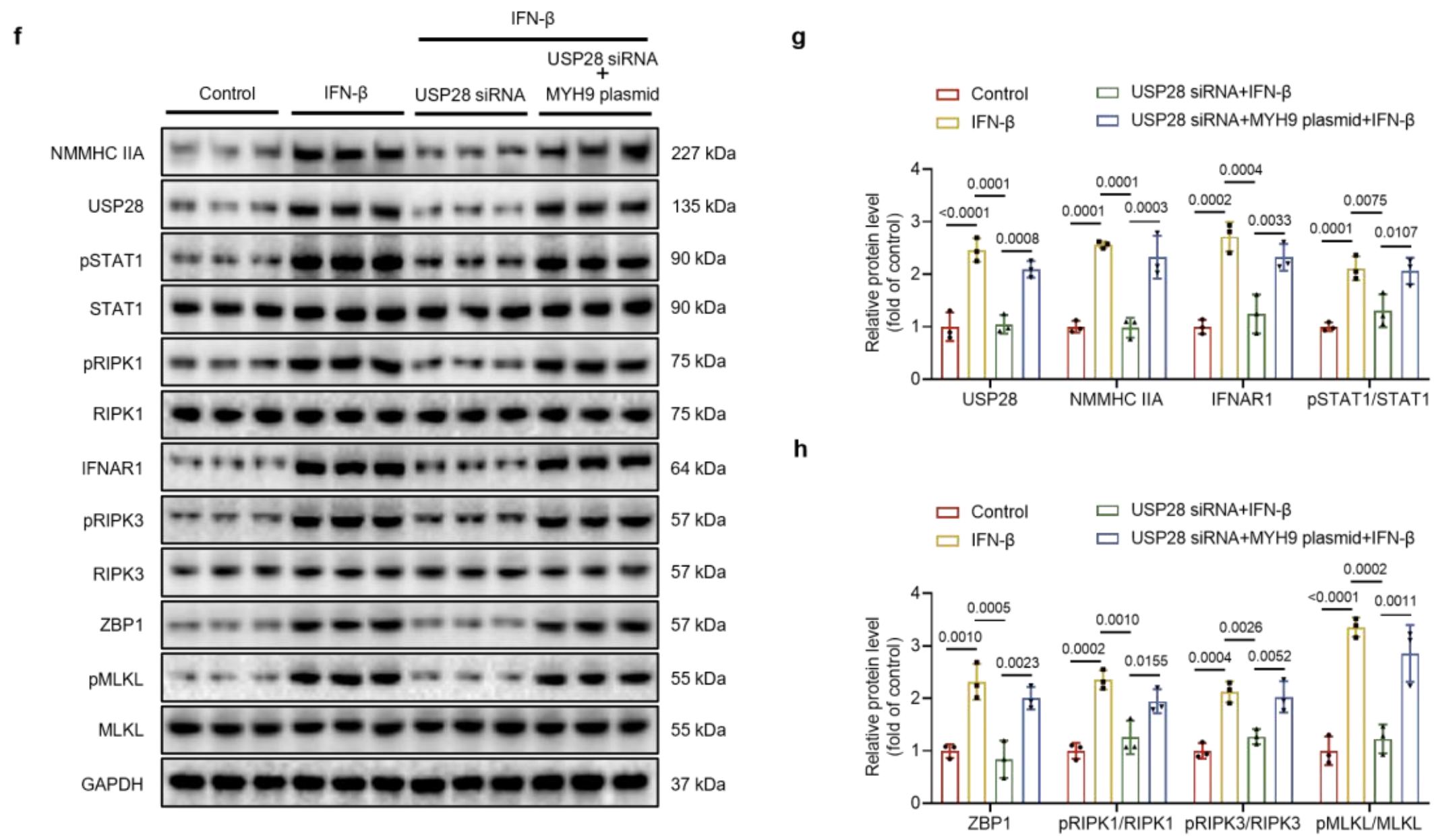

updated Supplementary Fig. 12

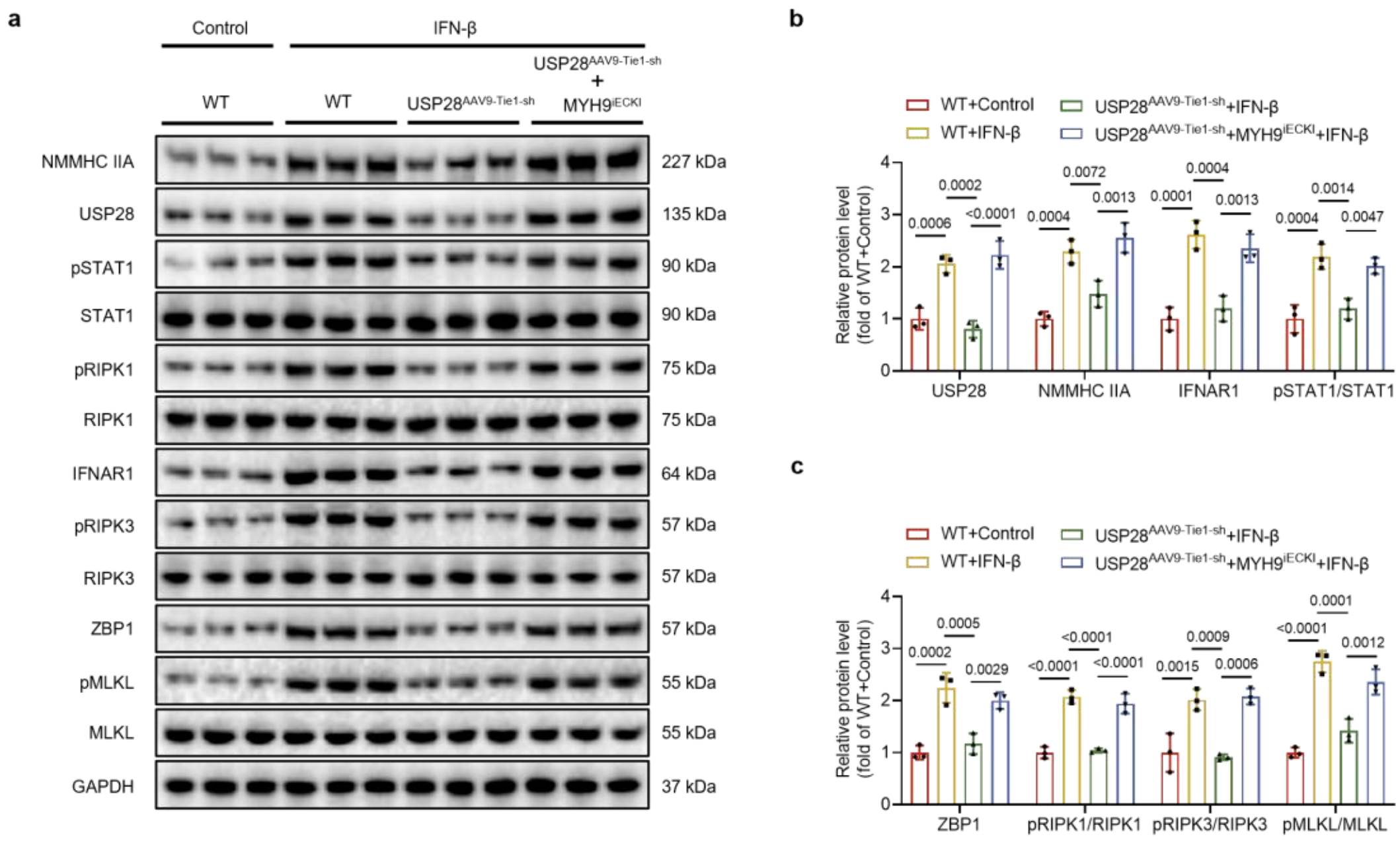

10. p-STAT1 Localization and Imaging (HUVECs)

Single-cell imaging is anecdotal and does not represent the population. Moreover, phosphorylated STAT1 (p-STAT1) is a transcription factor that should translocate to the nucleus. The provided images show predominant cytoplasmic localization, which is anomalous. The authors must explain this observation—is it an antibody specificity issue, a fixation artifact, or a biological peculiarity?

Response: In response to the concern about single-cell imaging, we have now repeated

the immunofluorescence staining and replaced the original images with representative multi-cell images to better reflect the overall population **(updated Supplementary Fig. S7d, e; updated Supplementary Fig. S10d, e)**. With respect to the predominantly cytoplasmic localization of p-STAT1, we sincerely apologize for this oversight. We re-evaluated the antibody used for immunofluorescence and found that its performance in HUVECs was indeed suboptimal. Upon further examination, we determined that this issue was likely due to the fact that the antibody had not been validated for use in human samples, which may have affected its specificity in this context. To address this issue, we replaced it with a validated antibody and repeated the staining experiments, and the updated images have been included in the revised manuscript.

11. Validation of AAV-IFNAR1 Rescue

The authors must demonstrate the transduction efficiency of AAV9-Tie1-IFNAR1 across all relevant organs (lung, liver, kidney, intestine). The experimental grouping in Figure 4 is incomplete; a "IFNAR-AAV9-Tie1 + CLP" group should be compared directly with the "USP28 + IFNAR-AAV9-Tie1 + CLP" group. Additionally, the baseline effect of AAV9-Tie1-IFNAR1 on normal (Sham) mice must be assessed.

Response: We sincerely appreciate the reviewer's insightful comments regarding the validation of AAV9-Tie1–mediated IFNAR1 overexpression and the experimental design of the rescue studies. To address the concern about transduction efficiency across organs, we isolated primary vascular endothelial cells from the lung, intestine, liver, and kidney of *IFNAR1*$^{\text{AAV9-Tie1}}$ mice and performed western blot analysis. The results demonstrate robust endothelial overexpression of IFNAR1 across all examined organs, with fold increases of approximately 3.63 in lung, 2.24 in intestine, 4.04 in liver, and 3.03 in kidney **(updated Supplementary Fig. 8b)**, confirming effective endothelial overexpression across multiple organs.

With respect to the experimental grouping, we agree that inclusion of additional groups such as *IFNAR1*$^{\text{AAV9-Tie1}}$ + Sham and *IFNAR1*$^{\text{AAV9-Tie1}}$ + CLP would further strengthen the completeness of the study. However, the central mechanistic conclusion of our work is derived from the within-cohort comparison between *USP28*^{ECKO} + CLP group and *USP28*^{ECKO} + *IFNAR1*$^{\text{AAV9-Tie1}}$ + CLP group, which were performed within the same experimental batch **(similar reference doi: 10.34133/research.1033)**. This design directly tests whether restoration of endothelial IFNAR1 signaling is sufficient to reverse the protective phenotype caused by endothelial USP28 deficiency. Consistently, re-expression of IFNAR1 in endothelial cells markedly reversed the protective effects observed in USP28-deficient mice, including survival, inflammatory responses, and multi-organ injury, thereby establishing IFNAR1 as a functional downstream effector of USP28 in sepsis. We acknowledge that inclusion of additional experimental groups

would further strengthen the study; however, performing these additional comparisons would require repeating the entire set of experiments under identical conditions, which in turn would require a substantial number of *USP28*^{ECKO} mice and involve a prolonged breeding period. Considering this, and given that the key mechanistic question is already addressed by the current rescue design, we focused on evaluating the baseline effect of *IFNAR1*$^{AAV9\text{-}Tie1}$. Specifically, we performed additional experiments in Sham-operated mice and confirmed that endothelial IFNAR1 overexpression does not significantly affect baseline physiological status **(updated Supplementary Fig. 8)**.

updated Supplementary Fig. 8

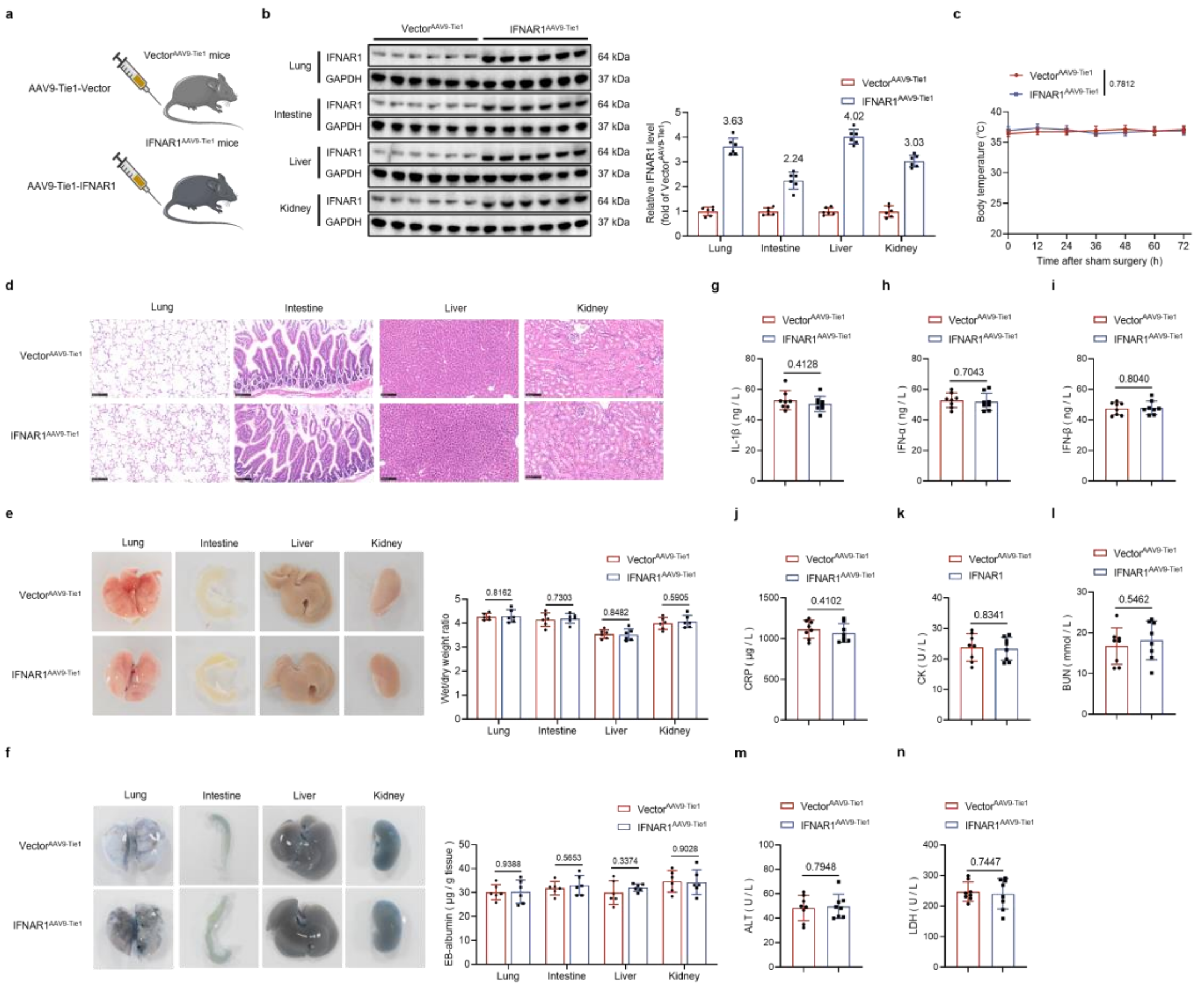

12. Ubiquitination Specificity

Does USP28 exclusively target K48-linked ubiquitination on NMMHC IIA? The authors should investigate whether it affects other linkage types (e.g., K63) to provide a complete mechanistic picture.

Response: We thank the reviewer for raising this important question regarding ubiquitin linkage specificity. We would like to clarify that this issue has already been experimentally addressed in the manuscript, although it was not sufficiently emphasized in the original presentation. We systematically examined the contribution

of multiple ubiquitin linkage types to NMMHC IIA ubiquitination using a comprehensive panel of ubiquitin mutants. Specifically, ubiquitin constructs corresponding to K6, K11, K27, K29, K33, K48, and K63 linkages were individually tested. These experiments demonstrate that modulation of USP28 selectively affects K48-linked ubiquitination of NMMHC IIA, whereas no consistent changes were observed for other linkage types, including K63-linked ubiquitination **(updated Fig. 5l, m)**. Therefore, the conclusion that USP28 preferentially targets K48-linked ubiquitin chains on NMMHC IIA is supported by systematic experimental evaluation across multiple linkage types, rather than by assumption.

updated Fig. 5l, m

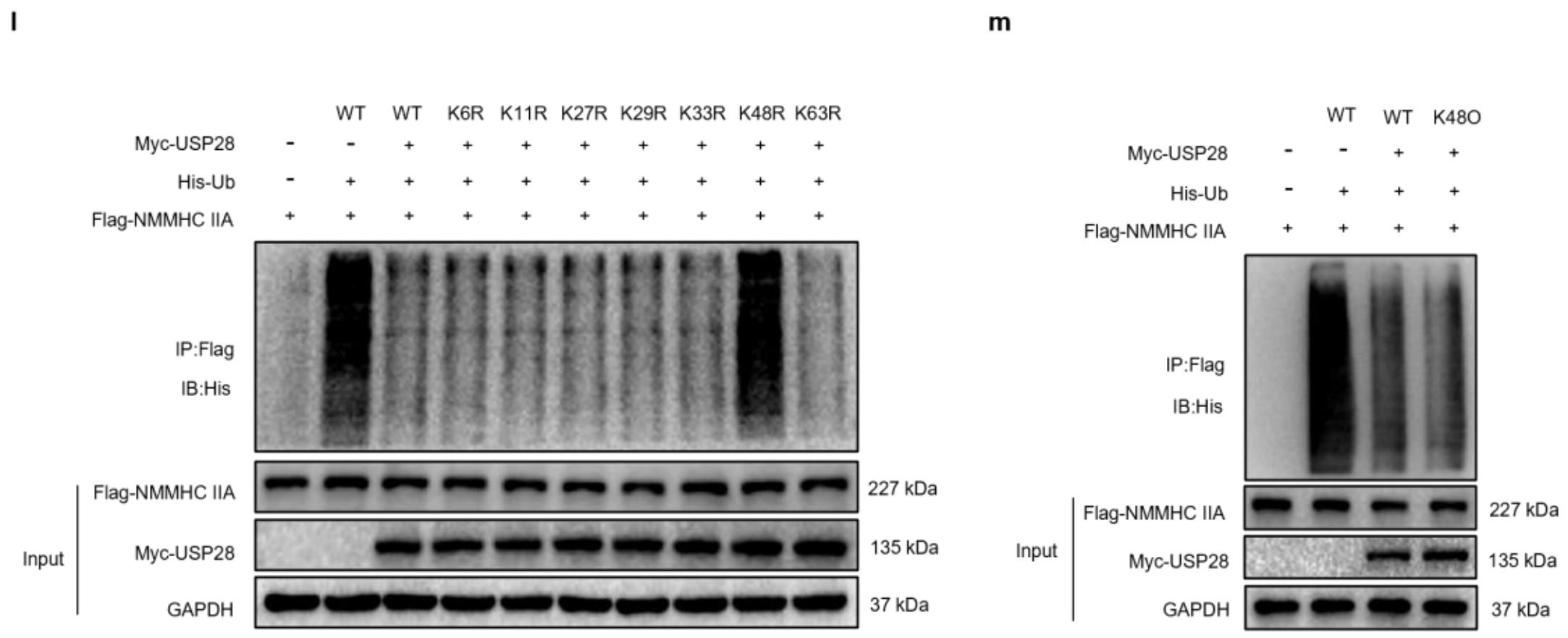

13. Direct Interaction Confirmation (Figures 5 & 6)

Co-IP alone is insufficient to claim "direct" binding. The interaction between USP28 and NMMHC IIA, as well as NMMHC IIA and IFNAR1, must be confirmed using GST-pull down assays (using purified proteins) and Proximity Ligation Assays (PLA) in cells.

Response: We thank the reviewer for this important suggestion to strengthen the evidence supporting the interactions among USP28, NMMHC IIA, and IFNAR1. We agree that co-immunoprecipitation alone cannot distinguish direct binding from indirect association within protein complexes, and that complementary approaches are required to support direct interaction. We note that the interaction between NMMHC IIA and IFNAR1 has already been examined using a proximity ligation assay (PLA), as shown in Fig. 6I **(updated Fig. 6j)**. This assay detects protein proximity at endogenous levels in intact cells and demonstrates close spatial association between NMMHC IIA and IFNAR1, providing in situ evidence consistent with a direct interaction. To further strengthen the evidence for the interaction between USP28 and NMMHC IIA, we have now performed additional PLA experiments to assess their proximity in cells. These

data provide direct in situ support for the association between USP28 and NMMHC IIA, complementing the co-immunoprecipitation results **(updated Fig. 5c, d)**. Regarding in vitro binding assays, we note that NMMHC IIA is a very large (~230 kDa) motor protein with filament-forming properties and complex higher-order assembly, making purification of full-length, properly folded protein technically challenging. In addition, purified full-length NMMHC IIA is not commercially available, which further limits the feasibility of GST pull-down assays in this context. Taken together, the combination of co-immunoprecipitation and in situ PLA analyses provides converging evidence supporting the interactions within the USP28–NMMHC IIA and NMMHC IIA–IFNAR1.

updated Fig. 5c, d

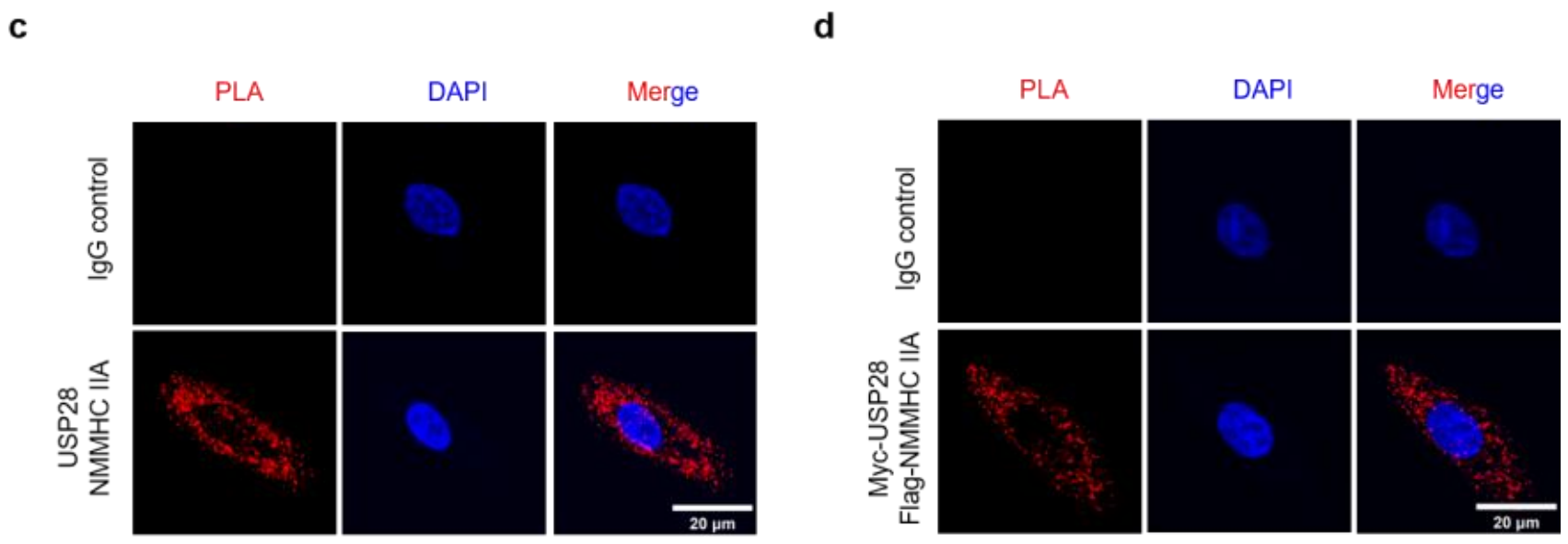

14. Validation of AAV-MYH9 Rescue

Similar to Point 11, the authors must validate the efficiency of AAV9-Tie1-MYH9 in all organs. The grouping in Figure 4 is incomplete (missing "MYH9-AAV + CLP" vs. "USP28 + MYH9-AAV + CLP") and lacks a baseline assessment in normal mice.

Response: As this concern is conceptually similar to that raised in Point 11, we have addressed it using a comparable validation strategy. We would like to clarify that, due to the large coding size of *MYH9* exceeding the packaging capacity of AAV9 vectors, endothelial-specific NMMHC IIA overexpression in this study was not achieved using an AAV9-based approach. Instead, we employed a tamoxifen-inducible endothelial-specific NMMHC IIA knock-in model (*MYH9*^{iECKI}) driven by Cdh5-CreERT2. In this genetic background, endothelial USP28 was selectively suppressed using AAV9-Tie1-shUSP28. To validate the efficiency of these genetic manipulations, we isolated primary vascular endothelial cells from the lung, intestine, liver, and kidney and performed western blot analyses. The results showed that endothelial NMMHC IIA overexpression in *MYH9*^{iECKI} mice was consistently maintained at approximately 1.75-fold across organs **(updated Supplementary Fig. 11b)**. In parallel, the knockdown efficiency of USP28 in *USP28*$^{\mathrm{AAV9\text{-}Tie1\text{-}sh}}$ mice reached 77.3 % in lung, 58.7 % in intestine, 77.8 % in liver, and 70.0 % in kidney **(updated Supplementary Fig. 3b)**.

Regarding the experimental grouping, consistent with our response to Point 11, the central conclusion of this rescue experiment is based on the direct comparison between the *USP28*$^{\text{AAV9-Tie1-sh}}$ + CLP group and the *USP28*$^{\text{AAV9-Tie1-sh}}$ + *MYH9*^{iECKI} + CLP group **(similar reference doi: 10.34133/research.1033)**, which were performed within the same experimental batch, allowing direct assessment of whether NMMHC IIA mediates the downstream effects of USP28. We acknowledge that inclusion of additional groups would further extend the completeness of the study; however, performing such experiments would require re-establishing the full set of experimental cohorts under identical conditions, involving substantial numbers of *MYH9*^{iECKI} mice and a prolonged breeding period. In addition, we have performed supplementary experiments to evaluate the baseline effects of NMMHC IIA overexpression under Sham conditions and found no significant alterations in physiological parameters **(updated Supplementary Fig. 11)**. Together, these data support the robustness of the NMMHC IIA rescue strategy and further reinforce the USP28–NMMHC IIA axis in sepsis.

updated Supplementary Fig. 11

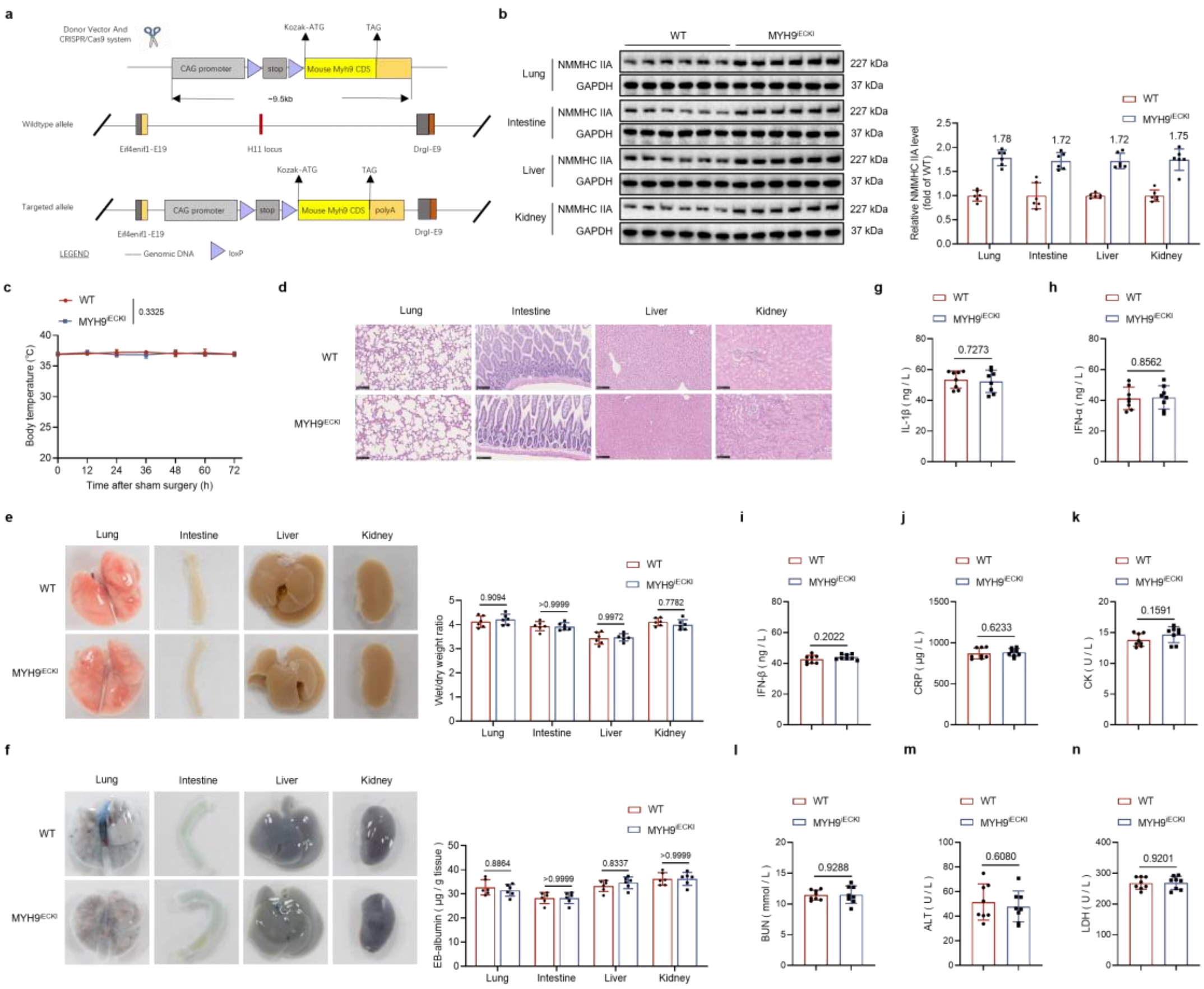

15. Validation of STAT1 Feedback Loop (Figure 8)

Western blotting is required to confirm the knockdown efficiency of si-STAT1 in Figure 8F. To rigorously prove the feedback loop, the authors should also perform a

STAT1 overexpression experiment and measure the resulting changes in USP28 expression and NMMHC IIA ubiquitination levels.

Response: We thank the reviewer for this insightful comment regarding the validation of the STAT1 feedback loop. In response to the concern about knockdown efficiency, we have now performed western blot analyses to confirm the effectiveness of si-STAT1 in HUVECs, and the corresponding data have been included in the revised manuscript **(updated Supplementary Fig. 13a)**. To further strengthen the evidence for the proposed feedback loop, we additionally performed STAT1 overexpression experiments. The results showed that STAT1 overexpression further enhanced IFN-I-induced USP28 and NMMHC IIA expression, and was accompanied by a further reduction in IFN-I-induced ubiquitination of NMMHC IIA **(updated Supplementary Fig. 13b–d)**.

updated Supplementary Fig. 13

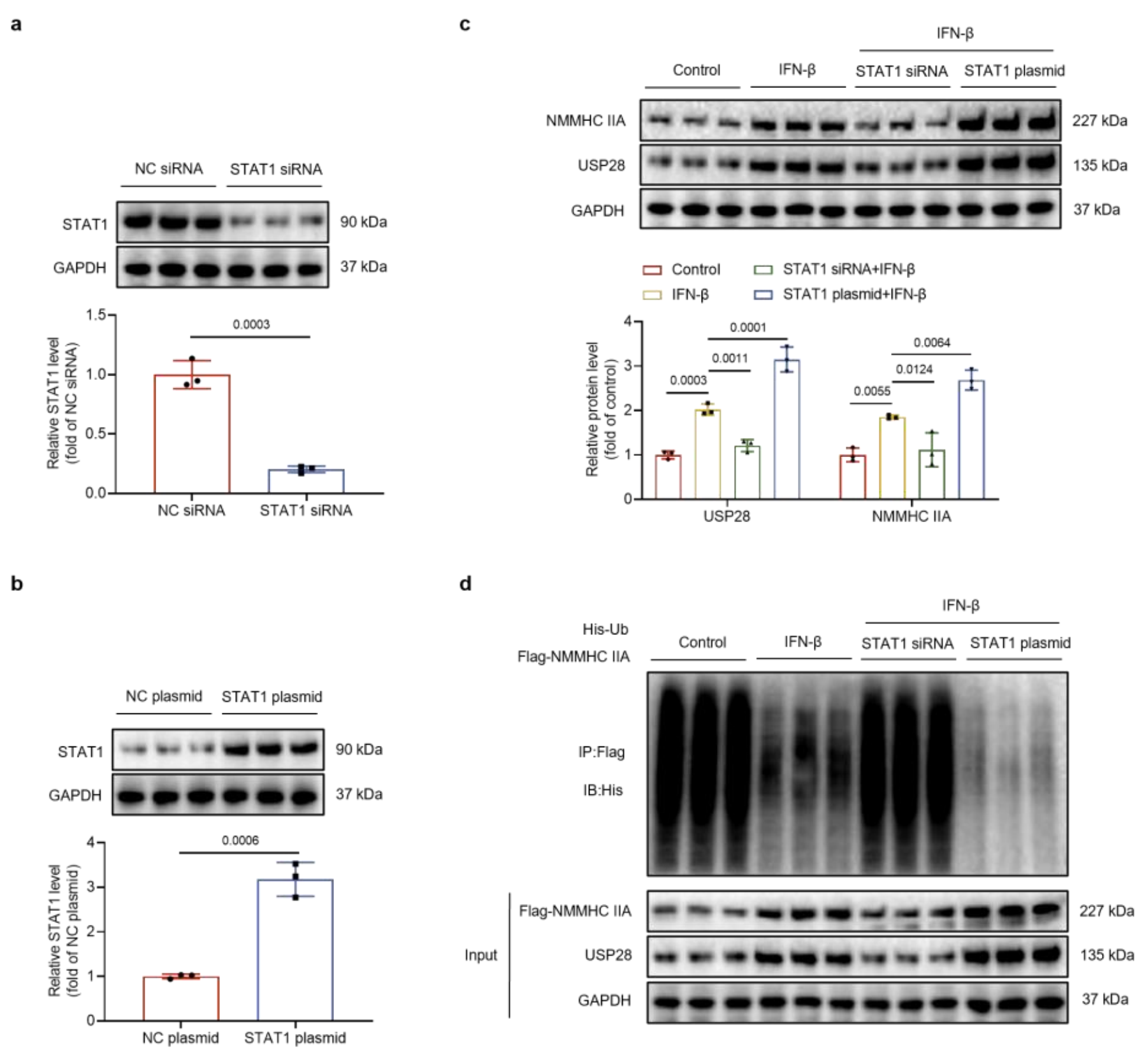

16. Over-interpretation of Lung-Specific Data

The conclusion that USP28 drives septic endothelial necroptosis via IFN-I signaling is predominantly based on scRNA-seq analysis derived exclusively from lung tissue (WT vs. USP28^{ECKO}). However, the authors extrapolate these findings to explain systemic multi-organ dysfunction, a generalization that lacks sufficient logical support.

Endothelial heterogeneity is a fundamental biological principle; both under physiological conditions and during sepsis, the molecular mechanisms and biological behaviors of endothelial cells differ significantly across distinct vascular beds. It is highly probable that the abundance of USP28 and the activation threshold of IFN-I signaling vary among organs, yet the authors only validated this pathway in lung tissue via immunofluorescence, leaving other critical organs unexamined. Furthermore, the use of HUVECs (macrovascular endothelial cells) in vitro fails to adequately represent the specialized phenotype of pulmonary microvascular endothelial cells. Consequently, relying solely on pulmonary and HUVEC data is insufficient to demonstrate the universal applicability of this mechanism in the context of systemic multi-organ injury.

Response: We thank the reviewer for this thoughtful and important comment. We fully agree that endothelial heterogeneity is a fundamental biological principle and that endothelial cells from distinct vascular beds exhibit organ-specific molecular features and activation thresholds under both physiological and septic conditions. In this study, lung tissue was selected as the primary site for single-cell transcriptomic analysis because the lung represents the earliest and most severely affected organ during sepsis and contains a highly extensive and exposed microvascular endothelial network. Accordingly, lung scRNA-seq was used as a discovery platform to identify endothelial pathways potentially involved in sepsis-associated organ injury, rather than to imply uniform endothelial behavior across all organs. At the multi-organ level, we have examined the temporal expression dynamics of endothelial USP28 in the lung, liver, kidney, and intestine under septic conditions using immunofluorescence analysis. These data demonstrate that endothelial USP28 is broadly upregulated across multiple organs during sepsis, supporting the concept that USP28 activation is not restricted to the pulmonary vasculature. In addition, functional readouts of organ injury were consistently assessed across multiple organs throughout the study.

With respect to mechanistic validation, we acknowledge that detailed molecular pathway analyses were primarily conducted in lung tissue. To strengthen the rigor of this aspect, we have now added Western blot validation using primary pulmonary microvascular endothelial cells, confirming the regulation of key components of the USP28–NMMHC IIA–IFN-I axis at the protein level in the relevant endothelial subtype. These additions reinforce that the proposed mechanism is directly supported by pulmonary endothelial cells rather than relying solely on HUVEC-based in vitro systems **(updated Supplementary Fig. 6; updated Supplementary Fig. 9; updated Supplementary Fig. 12)**. Importantly, we do not intend to claim that the magnitude or dynamics of USP28–IFN-I signaling are identical across all organs. To avoid overgeneralization, we have revised the manuscript to clarify this point and have

explicitly acknowledged in the Limitations section that mechanistic validation was performed primarily in lung endothelium, and that future studies will be required to delineate organ-specific regulatory features of this pathway **(page 17, lines 377–380)**. We believe that these clarifications and additions address the reviewer's concern and appropriately define the scope and boundaries of our conclusions.

Minor concerns：

1.Selection Criteria for DEGs (Figures 1C & 1E):

The authors appear to have used a relaxed threshold for identifying differentially expressed genes (DEGs). Conventionally, a cutoff of log2(Fold Change) ≥ 1 is recommended to ensure biological significance. While a lower threshold yields a higher number of candidates, it deviates from standard screening criteria and may include noise.

Response: We agree that a log2(Fold Change) ≥ 1 is often applied as a stringent criterion. In the present study, the nanoproteomics is specifically optimized for low-input samples and is inherently associated with reduced detection depth compared with conventional bulk proteomics. Under such conditions, the use of slightly relaxed fold-change thresholds is a commonly adopted strategy at the discovery stage to avoid missing biologically relevant candidates, particularly for proteins with moderate but functionally important changes **(doi: 10.1007/s00125-022-05849-5; doi: 10.1016/j.scib.2021.12.022)**. Accordingly, we used a cutoff of Fold Change > 1.5 in combination with adjusted *P* value < 0.05 to balance sensitivity and statistical robustness, rather than relying solely on effect size. Importantly, the central candidate highlighted in the manuscript, USP28, exhibited a differential signal with a log2(Fold Change) greater than 1, exceeding commonly used thresholds. Moreover, USP28 was subsequently validated through multiple independent experimental approaches, including protein-level assays, endothelial-specific genetic models, and functional analyses, ensuring that the key conclusions of the study do not rely solely on the initial proteomic screening.

2.Missing Statistical Annotations:

The graphs depicting mouse survival rates and body temperature changes lack statistical significance annotations (e.g., asterisks or p-values). These must be added to allow for proper interpretation of the data.

Response: We thank the reviewer for pointing out this omission. In the revised manuscript, we have added statistical significance annotations to the graphs depicting mouse survival rates and body temperature changes, including exact p values where appropriate. For survival analyses, statistical comparisons were performed using the

log-rank (Mantel–Cox) test, and the corresponding *p* values are now indicated in the figures. For body temperature, quantitative comparisons were performed based on area under the curve analysis followed by multiple-group statistical testing. These revisions improve the clarity and interpretability of the data **(updated Fig. 2c, d; updated Fig. 4a, b; updated Fig. 7a, b; updated Supplementary Fig. 2a, b; updated Supplementary Fig. 3c; updated Supplementary Fig. 4c, d; updated Supplementary Fig. 8c; updated Supplementary Fig. 10c)**.

3.Inconsistency in CRP Levels (Figures 2, S2, & S3):
There is a substantial discrepancy in the average serum CRP levels reported across Figures 2, S2, and S3. The magnitude of these differences is concerning. The authors should verify whether this is due to inconsistencies in the detection assay, batch effects, or variations in the experimental system.

Response: We apologize for our oversight. Upon re-examination of the original data, we identified an error in the calculation of CRP levels in Figure 2. Specifically, the plasma samples used for CRP measurement were diluted three-fold prior to detection; however, the corresponding values were inadvertently not multiplied by the dilution factor when calculating the final concentrations. We have now corrected this error, and the updated data are presented in **update Fig. 2m**. Importantly, we have carefully re-checked the raw data and analysis procedures for CRP measurements in all relevant figures, and no similar issues were identified. The observed differences among Figures 2, S2, and S3 therefore reflect variations in independent experimental batches rather than calculation inconsistencies. These corrections do not affect the overall trends or conclusions of the study.

update Fig. 2m

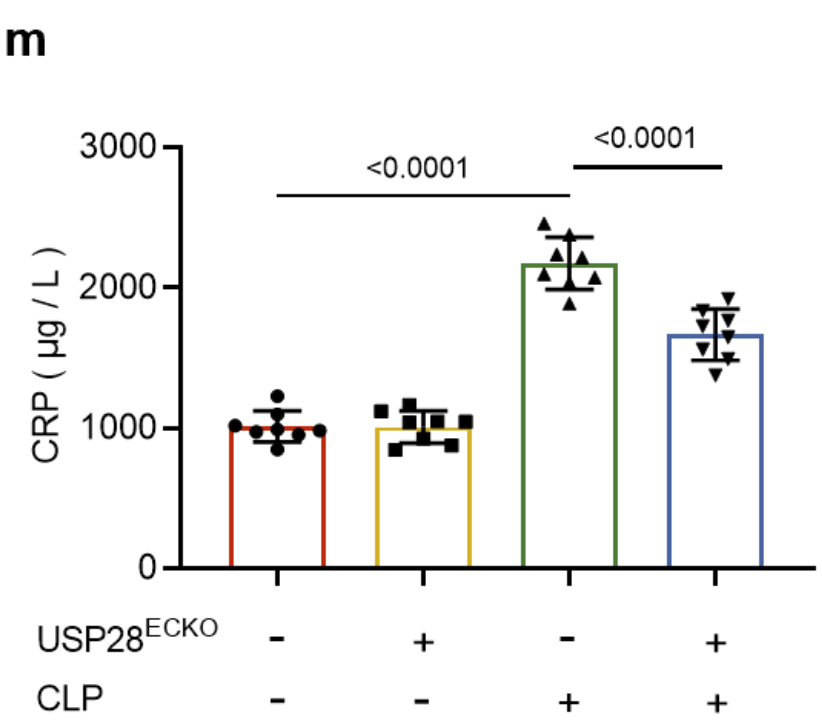

4.Graph Formatting:
The formatting style of the statistical graphs is inconsistent throughout the manuscript. Please standardize the layout, font sizes, and axis styles for a uniform presentation.

Response: We thank the reviewer for pointing out the inconsistency in graph formatting.

In the revised manuscript, we have standardized the formatting of all statistical graphs, including layout, font size, axis style, and labeling conventions. These adjustments were applied consistently across all figures to ensure a coherent and uniform visual presentation.

5.Alternative Explanations for Cell Numbers (Figure 3D):

Regarding Figure 3D, the observed increase in endothelial cell numbers could theoretically result from self-proliferation or differentiation from progenitor cells. Although investigating these specific pathways may be outside the scope of this study, the authors should briefly discuss these possibilities as potential contributing factors.

Response: To further address this possibility, we performed additional immunofluorescence co-staining for CD31 and Ki67 in lung tissues following CLP. Quantitative analysis showed that the proportion of Ki67-positive cells among CD31-positive endothelial cells was not significantly different between WT and *USP28*^{ECKO} mice (**update Fig. 3e**). Consistently, Gene set enrichment analysis (GSEA) of ECs clusters showed no enrichment of proliferation-related signatures, suggesting that the increased ECs proportion is unlikely driven by enhanced proliferation (**update Fig. 3f**). These results suggest that altered endothelial self-proliferation is unlikely to account for the increased endothelial cell abundance observed in the lungs of *USP28*^{ECKO} mice after CLP.

update Fig. 3e, f

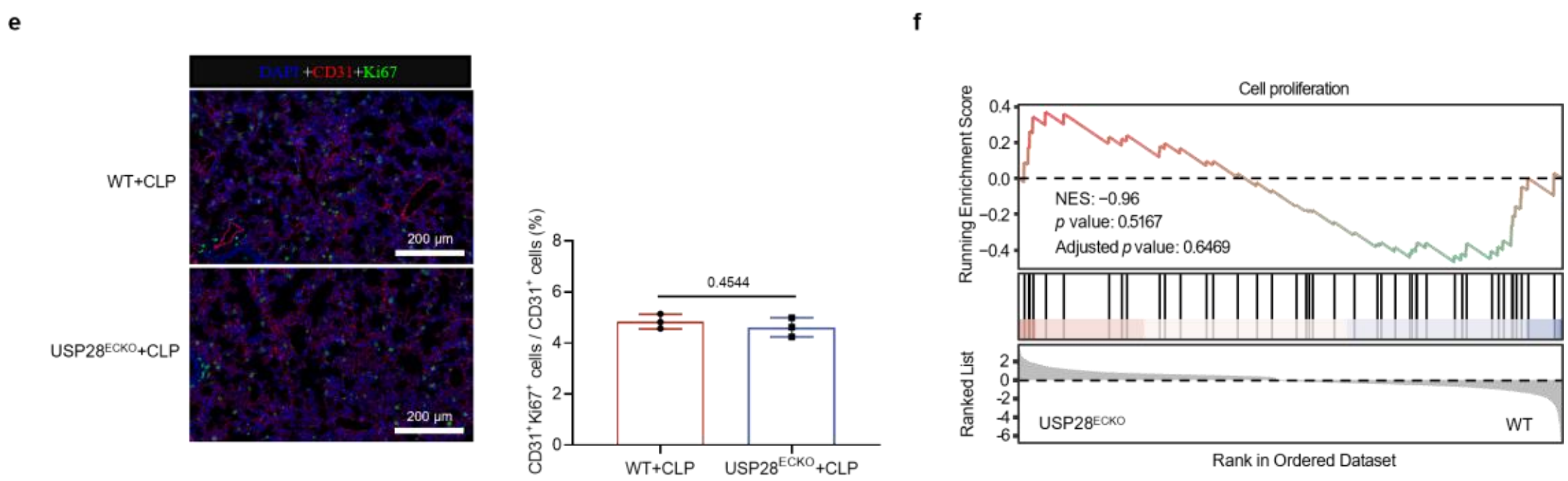

6.Contextualizing NMMHC IIA Function:

The functional role of NMMHC IIA in IFN-I signaling is currently mentioned only in the Abstract. To enhance readability and flow, this background information should also be elaborated upon in the Introduction and explicitly reintroduced in the text corresponding to Result sect.

Response: We thank the reviewer for this helpful suggestion. We would like to clarify that, in the original design of the Introduction, NMMHC IIA was not introduced upfront because its involvement in IFN-I signaling was not previously established and was instead identified as a key finding emerging from our study. Therefore, we chose to

present NMMHC IIA primarily within the Results section as part of the mechanistic discovery process, rather than as a predefined component of the signaling framework. To improve clarity and better guide the reader, we have now revised the manuscript by adding brief contextual information to introduce NMMHC IIA at appropriate points in the text and by explicitly reintroducing its role in IFN-I signaling within the Results and Discussion sections **(pages 16–17, lines 360–373)**. These revisions help improve the narrative continuity while preserving the logic of discovery underlying our study.

Reviewer #4 (Remarks to the Author):

This manuscript presents a comprehensive and mechanistically well-supported study elucidating the role of USP28 in sepsis-induced endothelial necroptosis and multi-organ injury. The study initiated with nanoproteomics from patients to identify USP28 as a key deubiquitinase upregulated in sepsis and demonstrate that endothelial USP28 promotes vascular injury and mortality in a polymicrobial sepsis model. Mechanistically, USP28 stabilizes NMMHC IIA through deubiquitination, thereby sustaining IFNAR1 membrane localization and amplifying IFN-I signaling, which ultimately drives endothelial necroptosis and barrier dysfunction. Functionally, endothelial-specific overexpression of IFNAR1 or NMMHC IIA largely reversed the protective effects of USP28 inhibition on sepsis-induced multi-organ injury. This study provides mechanistic insight into endothelial regulation of IFN-I signaling during polymicrobial sepsis and helps explain how sustained interferon responses contribute to vascular injury in severe infection. As such, these original findings should appeal to a broad audience, including clinicians, infectious diseases researchers, and immunologists. The overall conclusions are well supported by the data, and the study design is elegant. But there some issues should be addressed prior to publication.

Response: We sincerely thank the reviewer for the positive and encouraging evaluation of our study. We are particularly grateful for the recognition of the mechanistic depth, experimental design, and the potential relevance of our findings to both basic research and clinical contexts. We appreciate the reviewer’s thoughtful comments and constructive suggestions. We have carefully addressed each of the points raised and have revised the manuscript accordingly. Our detailed responses are provided below.

1.The study exclusively utilized male mice for all in vivo experiments. The authors are encouraged to briefly justify this experimental choice and to comment on whether sex-specific effects might influence the interpretation of the findings.

Response: We thank the reviewer for this important comment regarding the use of male mice for *in vivo* experiments. With respect to sex, we have now explicitly acknowledged this as a limitation in the revised manuscript **(Page 17, Lines 374–377)**.

In this study, male mice were selected because of their lower variability in the caecal ligation and puncture (CLP) model compared with female mice. Sepsis is characterized by systemic inflammation and immune dysregulation, whereas female mice exhibit cyclic fluctuations in sex hormone levels that can influence inflammatory responses and tolerance to CLP-induced injury, thereby introducing additional variability **(doi: 10.1038/nprot.2008.214; doi: 10.1172/jci.insight.180132)**. In addition, female sex hormones have been reported to exert protective effects in sepsis-associated cardiac injury **(doi: 10.1038/s41598-022-08889-4)**. Therefore, the use of male mice is consistent with the majority of experimental sepsis studies and facilitates more stable and reproducible phenotypic assessment **(doi: 10.1016/j.phrs.2022.106473; doi: 10.1002/ptr.8093)**.

2.In the Methods section, the description of the Evans blue–albumin leakage assay includes a wavelength of “20 nm”, which appears inconsistent with typical UV–Vis absorbance measurements. The authors should check and correct this value.

Response: We thank the reviewer for pointing out this error. We confirm that the reported wavelength of “20 nm” in the description of the Evans blue–albumin leakage assay was a typographical mistake. The correct absorbance wavelength used for Evans blue quantification is 620 nm, and this has now been corrected in the Methods section of the revised manuscript **(Page 33, Line 748)**. We apologize for this oversight and thank the reviewer for helping us improve the accuracy and clarity of the methodological description.

3.In Figure 1H, the immunohistochemical images labeled as liver and kidney appear inconsistent with the expected tissue morphology. Please verify whether the labeling of these panels is correct.

Response: We apologize for our oversight. We thank the reviewer for carefully examining Figure 1H and for pointing out the inconsistency in tissue morphology. We confirm that this issue was due to an inadvertent labeling error in the representative images: the labels for the liver and kidney panels were mistakenly interchanged. This labeling error has now been corrected in **update Fig. 1k**. The underlying images, quantitative analyses, and conclusions of the study remain unchanged. We apologize for this oversight and thank the reviewer for helping us improve the accuracy of figure presentation.

4.In Figure 5B, during the IP/MS-based identification of USP28-interacting proteins, the authors focused on NMMHC IIA for subsequent mechanistic analyses. The authors are encouraged to clarify the criteria used to prioritise this candidate, such as peptide

counts or enrichment fold.

Response: We thank the reviewer for the suggestion to clarify the criteria used to prioritize USP28-interacting candidates identified by IP-MS. The selection of NMMHC IIA for subsequent mechanistic analyses was primarily based on objective criteria derived from the IP-MS dataset. Among all proteins identified in the USP28 immunoprecipitates, NMMHC IIA showed the most abundant peptide enrichment and the highest confidence, reflected by high unique peptide counts and robust enrichment relative to IgG controls. Notably, NMMHC IIA was identified with 205 unique peptides in the IP-MS analysis, supporting the robustness and reliability of this interaction. Accordingly, NMMHC IIA ranked at the top of the USP28-interacting candidates in our IP-MS results **(Page 10, Lines 209–210)**. To enable full transparency, the complete IP-MS search output and ranking information (including peptide/protein identifications and associated metrics), together with the raw mass spectrometry files, have been deposited in a publicly accessible proteomics repository, with accession information provided in the revised manuscript **(Page 34, Lines 762–768)**.

5.In Figure 5L, the identification of K1413 as the key deubiquitination site is convincing. Please clarify whether this site is evolutionarily conserved, which would strengthen the biological significance.

Response: We thank the reviewer for this insightful suggestion. To address this point, we performed sequence alignment analysis using UniProt database resources to examine the conservation of the identified deubiquitination site. The results show that the K1413 residue of NMMHC IIA is highly conserved between human and mouse (**Fig. R1**). This evolutionary conservation supports the functional importance of this site and further strengthens the biological relevance of our findings.

Fig. R1

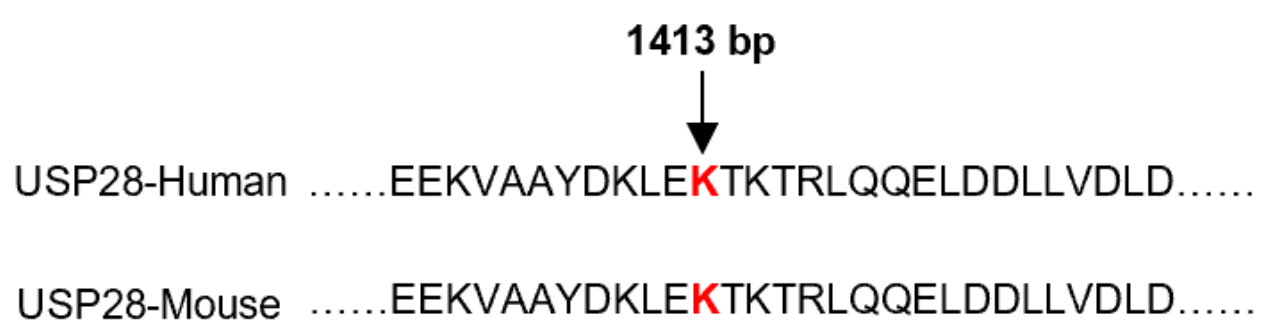

6.In Figure 6G, IFNAR1 membrane expression is presented. Please clarify whether this result was obtained using membrane protein fractionation, as this method does not appear to be explicitly described in the Methods section.

Response: We thank the reviewer for this helpful question regarding the assessment of IFNAR1 membrane. We clarify that the IFNAR1 membrane expression shown in Figure 6G was obtained using membrane protein fractionation. In response to this

comment, we have revised the Methods section to explicitly describe the membrane protein isolation procedure, including the fractionation approach used to separate membrane and cytosolic protein fractions prior to immunoblot analysis **(Page 32, Lines 709–716)**.

7.Cell apoptosis, pyroptosis and other forms of programmed cell death have also been reported to play important roles in the pathogenesis of sepsis. The authors are encouraged to further discuss why the present study specifically focuses on the role of USP28 in endothelial necroptosis.

Response: We thank the reviewer for this important comment regarding the specificity of necroptosis and the need for more rigorous validation of cell death modalities. In response, we have performed additional experiments to more comprehensively characterize the mode of endothelial cell death. Specifically, we examined representative markers of apoptosis and pyroptosis in both primary mouse lung endothelial cells (MLECs) and HUVECs under IFN-I stimulation. We found that IFN-I did not alter the expression of cleaved caspase-3 or GSDMD-N in MLECs, and neither USP28 deficiency nor USP28 overexpression affected the levels of these markers. Consistent results were obtained in HUVECs, in which IFN-I likewise failed to induce cleaved caspase-3 or GSDMD-N, and modulation of USP28 expression did not affect either apoptotic or pyroptotic marker levels. These data indicate that, under our experimental conditions, IFN-I-induced endothelial injury is not accompanied by detectable activation of apoptosis or pyroptosis **(updated Supplementary Fig. S6d–g; updated Supplementary Fig. S7i–l)**. In addition, to provide more direct functional evidence for necroptosis, we further performed loss-of-function experiments targeting key downstream mediators of the necroptotic pathway in HUVECs. Notably, silencing of MLKL or RIPK3 using siRNA almost completely blocked the IFN-I-induced reduction in cell viability, indicating that the endothelial injury observed in this setting is highly dependent on the canonical RIPK3-MLKL necroptotic axis **(updated Supplementary Fig. S7m–o)**. When considered together with our original data showing activation of IFNAR1/STAT1 signaling and increased expression of ZBP1, pRIPK1, pRIPK3, and pMLKL, these new findings provide much stronger evidence that USP28 regulates IFN-I-driven endothelial necroptosis rather than apoptosis or pyroptosis.

8.A few grammatical inconsistencies and typographical errors are present (e.g., “IFANR1” instead of “IFNAR1” in one section) and should be corrected during revision.

Response: We apologize for our oversight. We have thoroughly checked the manuscript

and corrected all such errors, including the typographical error “IFANR1,” which has now been revised to “IFNAR1” **(Page 9, Line 190)**. The manuscript has also been carefully proofread to ensure overall clarity and accuracy.

9.Overall, the study is carefully executed and the data are of high quality. Because of the wealth of data, the arguments are not always easy to follow. A graphical abstract detailing the main molecular findings described in the manuscript would improve clarity for the reader.

Response: We thank the reviewer for this helpful suggestion. To improve the clarity and overall accessibility of the manuscript, we have added a schematic model summarizing the proposed USP28–NMMHC IIA–IFNAR1/STAT1 signaling axis and its role in endothelial necroptosis during sepsis **(updated Supplementary Fig. 14)**. This schematic is intended to provide an integrated overview of the key molecular relationships described throughout the study and to facilitate interpretation of the experimental findings.

Responses to the Reviewers' comments

Title: USP28 potentiates endothelial necroptosis via deubiquitinating NMMHC IIA in sepsis

Manuscript Number: NCOMMS-25-86271

We sincerely thank the reviewers for their continued evaluation of our manuscript and for their valuable and constructive comments. We have carefully considered all remaining concerns raised during this round of review. In response, we further clarified the interpretation of lung tissue immunofluorescence images, re-examined the lung histopathology data with professional pathological input, refined the explanation of the CLP model design, and expanded the legend and annotation of Supplementary Fig. 14 to make the graphical model more self-explanatory. All corresponding modifications have been reflected in the revised manuscript, figure legends, and supplementary materials where appropriate.

The following are our point-by-point responses to each question. The reviewers' comments are displayed below, followed by our responses. We believe that these additional revisions have further improved the clarity, rigor, and reliability of the manuscript.

Reviewer Comments

Reviewer #1 (Remarks to the Author):

The authors should be commended for their thoughtful and thorough revisions. The revised manuscript has addressed all of my concerns and is suitable for publication as is.

Response: We thank the reviewer for the positive comments.

Reviewer #3 (Remarks to the Author):

The authors' previously submitted manuscript suffered from substantial data quality issues, which was my primary reason for raising concerns and recommending against publication. I note significant improvements in the data quality of this revised submission, and the authors' efforts during the revision process are highly commendable. While most of my initial concerns have been addressed with reasonable explanations, several points still require further clarification.

Response: We sincerely thank the reviewer for the careful re-evaluation of our revised manuscript and for recognizing the substantial improvements in data quality. We are grateful for the reviewer's acknowledgment of our revision efforts and for the constructive comments on the remaining issues. We fully appreciate the importance of these concerns and have taken them seriously in this further revision. We have provided additional clarification and made corresponding revisions to improve the accuracy of data interpretation, the transparency of the experimental design, and the clarity of figure presentation. We hope that the responses below adequately address the reviewer's remaining concerns.

1. Issues remain with some of the immunofluorescence (IF) images. Specifically, proteins with distinctly different subcellular localizations—such as ZBP1 (cytoplasm), pRIPK1 (cytoplasm), pRIPK3 (cytoplasm), IFNAR1 (cell membrane and cytoplasm), and pSTAT1 (nucleus)—all appear to be predominantly localized to the nucleus in the IF images of lung tissues. This is highly unusual and anomalous. The authors must address this issue, as it directly impacts the validity and biological rationale of the experimental data. Furthermore, when quantifying these IF results, the authors should evaluate the fluorescence intensity specifically within lung endothelial cells rather than calculating the overall intensity across the entire lung tissue.

Response: We thank the reviewer for this important and insightful comment. We fully agree that ZBP1, pRIPK1, pRIPK3, IFNAR1, and pSTAT1 have distinct expected subcellular distributions, and that tissue immunofluorescence images should not be interpreted in a way that contradicts established biological localization. We would like to clarify that the lung tissue IF images do not indicate that these proteins are predominantly localized in the nucleus. Rather, in some lung cryosections, signals from cytoplasmic or membrane-associated proteins may appear to overlap with DAPI-positive regions in two-dimensional displayed images, thereby creating an apparent nuclear overlap. A likely technical reason is that the lung cryosections used for IF staining were cut at a thickness of 10 μm. Such sections do not necessarily represent a true single-cell optical layer, but may include more than one cellular layer or partially overlapping subcellular structures along the z-axis. Confocal images acquired from tissue sections represent fluorescence signals collected within a defined optical volume. When these signals are displayed as a single two-dimensional image or projection, fluorescence from cytoplasmic or membrane compartments located above or below the nucleus may be superimposed onto the DAPI-positive nuclear region. This is particularly relevant in lung tissue, where endothelial cells, epithelial cells, immune cells, and stromal cells are densely packed and spatially intermingled. Therefore, apparent overlap between non-nuclear markers and DAPI in tissue IF images should

not be interpreted as true nuclear localization. Even under these conditions, some degree of signal overlap may occur because different subcellular compartments can occupy different depths within the same x–y position. In the present study, the cell-based immunofluorescence experiments provide more reliable evidence for subcellular localization, whereas lung tissue IF was mainly used to evaluate target proteins expression changes in endothelial regions in situ, rather than to define precise subcellular localization.

Figure 2I from research of *Advanced Science* (Doi: 10.1002/advs.202407064)

Subcellular localizations: Sydecan-1 (cell membrane), HSPG2 (cytoplasm)

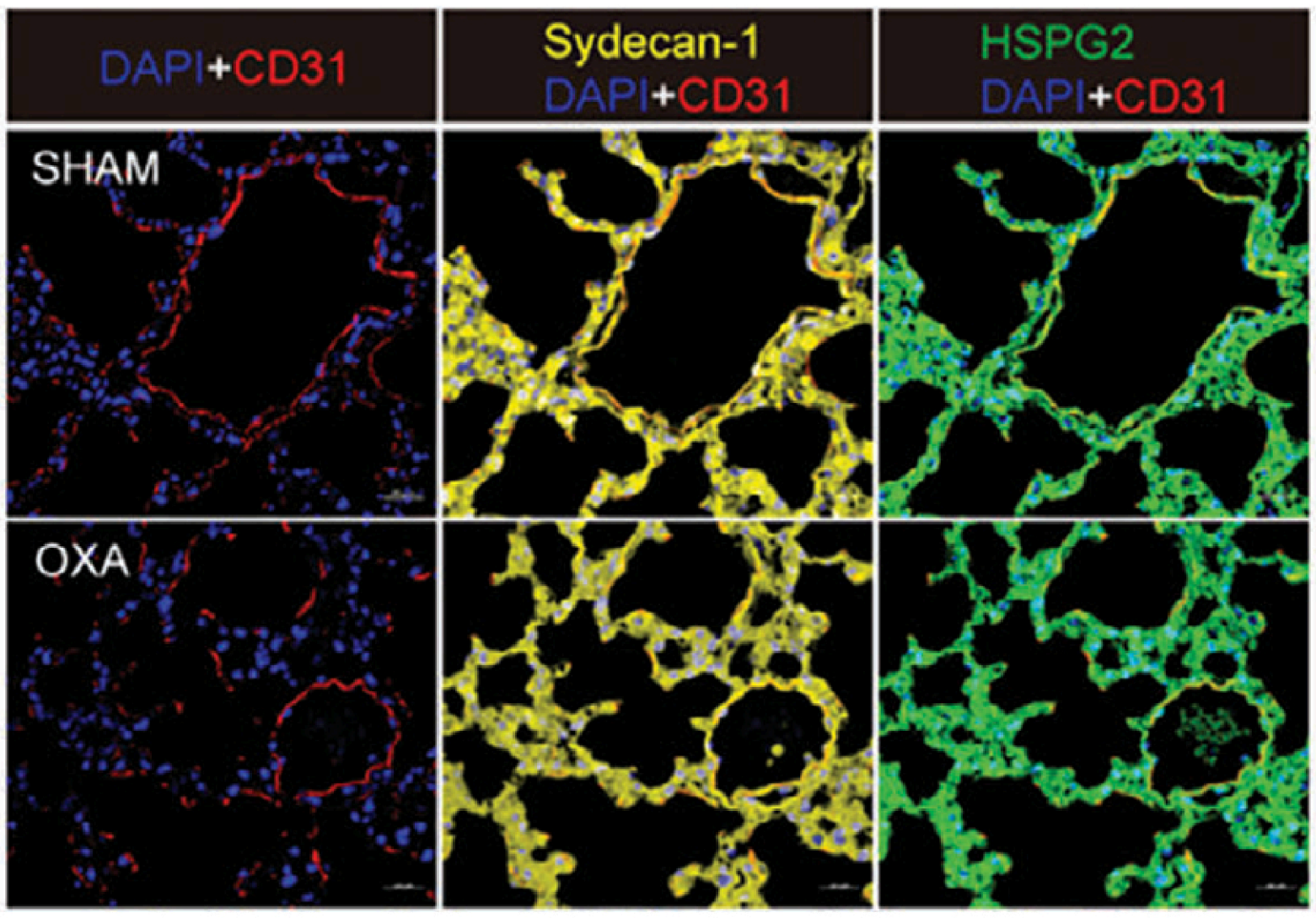

Figure 2L from research of *Circulation Research* (Doi: 10.1161/CIRCRESAHA.124.325855)

Subcellular localizations: Claudin-5 (cell membrane)

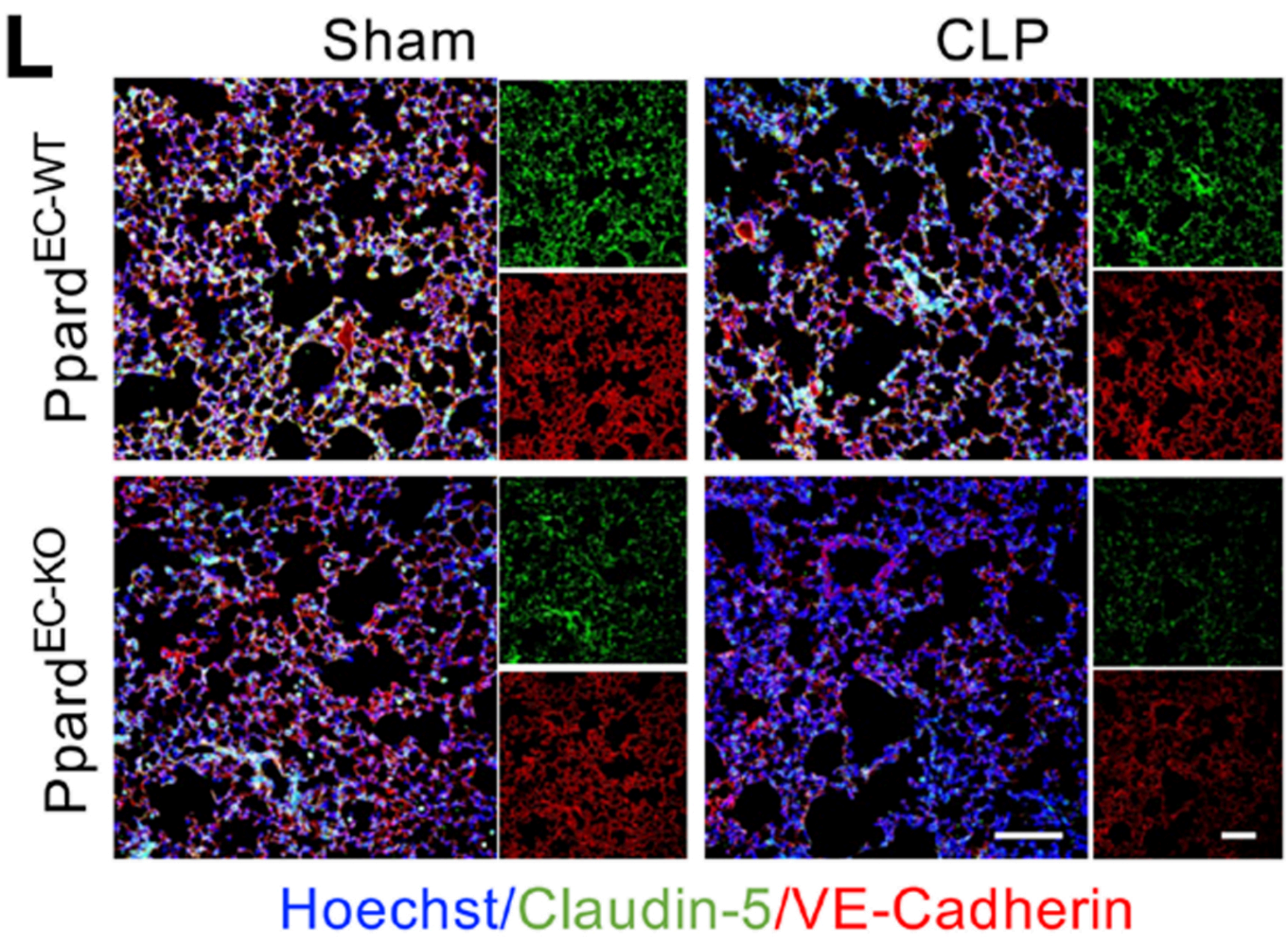

Regarding the reviewer's concern about quantification, we would like to clarify that our original quantification was not based on the overall fluorescence intensity across the entire lung tissue section. As described in the corresponding figure legends, the IF quantification was performed to evaluate the expression of these proteins in lung endothelial cells. Specifically, CD31-positive endothelial regions were used to identify lung endothelial cells, and the fluorescence intensity of target proteins was quantified within these endothelial regions, rather than across the whole lung tissue.

2. Although the authors have resolved the quality issues regarding the tissue section data, it must be clarified that while perfusion can indeed cause alveolar collapse or minor structural deformation, it should not result in massive structural destruction. The lung pathology images presented in the original manuscript were largely unacceptable from the perspective of pulmonary pathologists.

Response: We thank the reviewer for this important clarification. We fully agree that cardiac perfusion and subsequent tissue processing for H&E staining may cause a certain degree of unavoidable structural alteration in lung tissue, particularly because the alveolar wall is fragile. However, such procedure-related changes should generally be mild and should not be used to explain massive destruction of lung architecture. We apologize that the representative lung pathology images shown in the original manuscript were not optimal for pathological interpretation. To address this issue more rigorously, we re-consulted professional pulmonary pathologists and asked them to carefully evaluate our original lung histopathological sections. Based on their assessment, more representative images from the original lung histopathological sections were used for presentation in the revised manuscript **(updated Fig. 2e; updated Fig. 4c; updated Fig. 7c; updated Supplementary Fig. 2c; updated Supplementary Fig. 3d; updated Supplementary Fig. 4e; updated Supplementary Fig. 8d; updated Supplementary Fig. 11d)**. The revised images better reflect the pathological changes induced by CLP while avoiding misleading interpretation caused by suboptimal tissue sections. We appreciate the reviewer's careful and professional evaluation of this issue, which helped us improve the pathological rigor and reliability of the lung injury data.

updated Fig. 2e

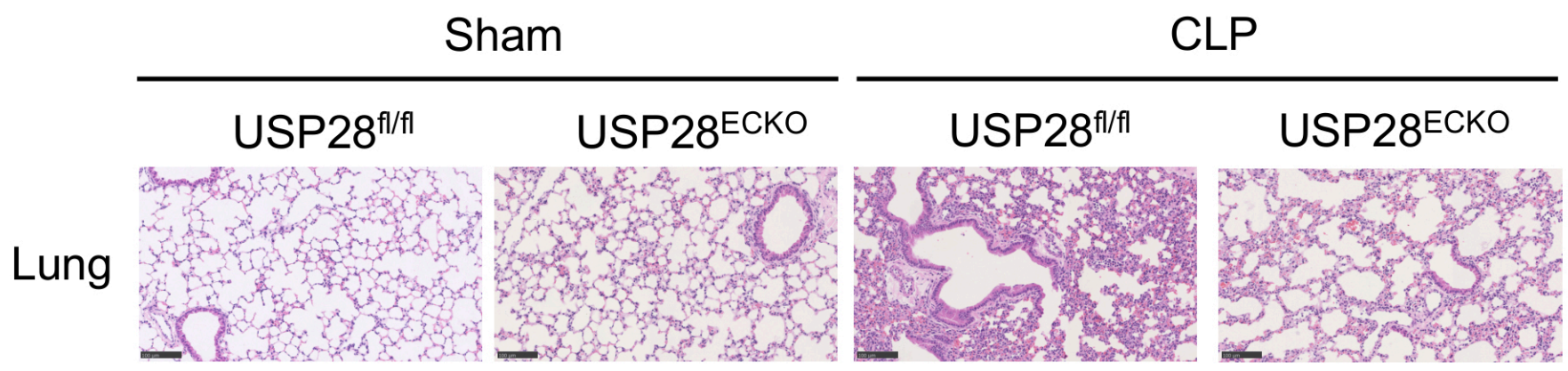

updated Fig. 4c

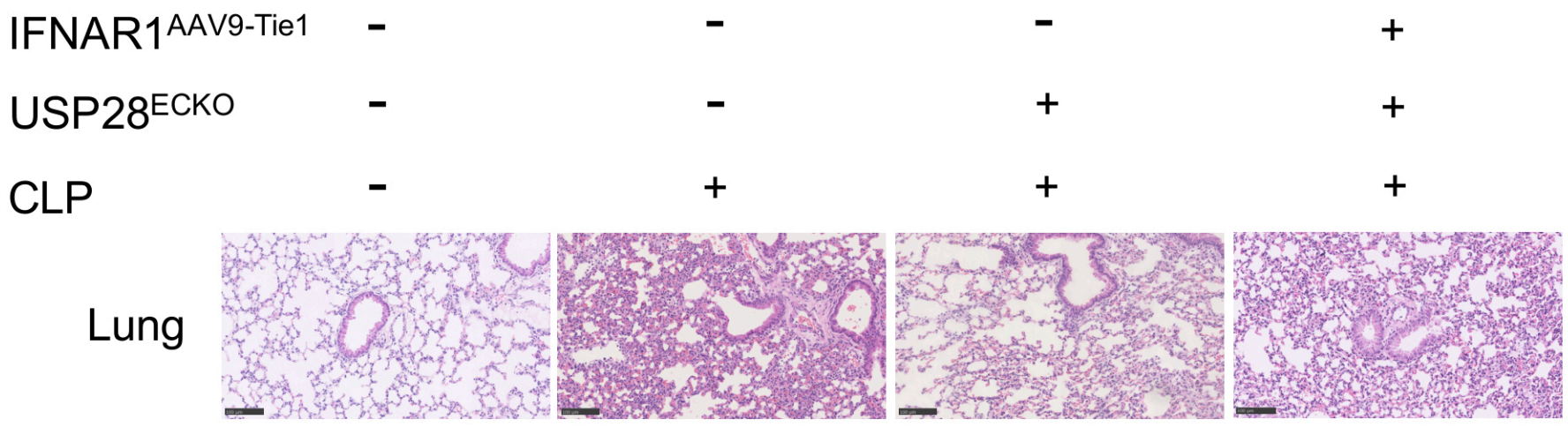

updated Fig. 7c

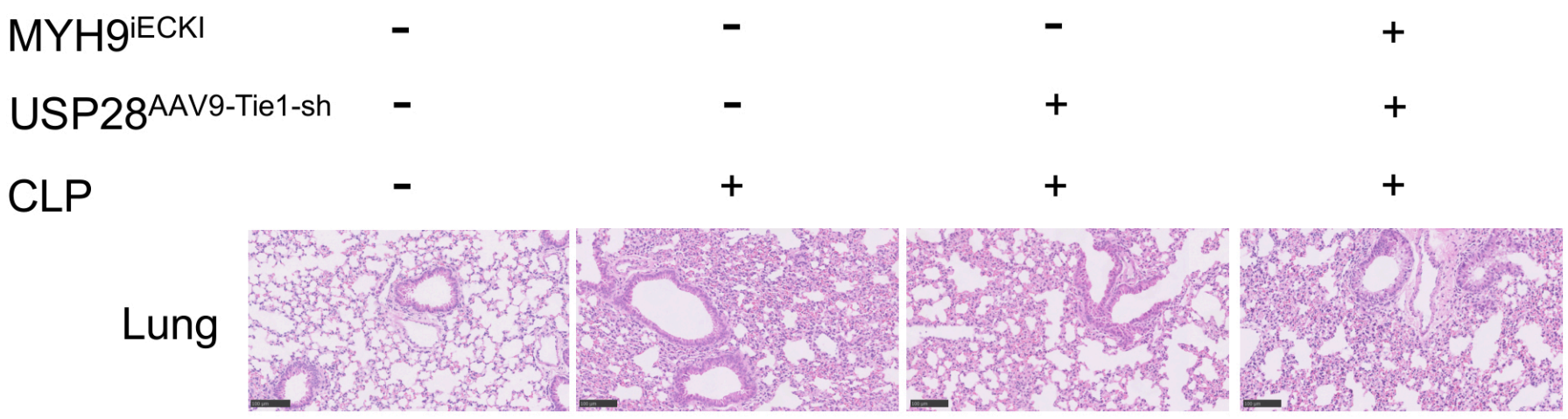

updated Supplementary Fig. 2c

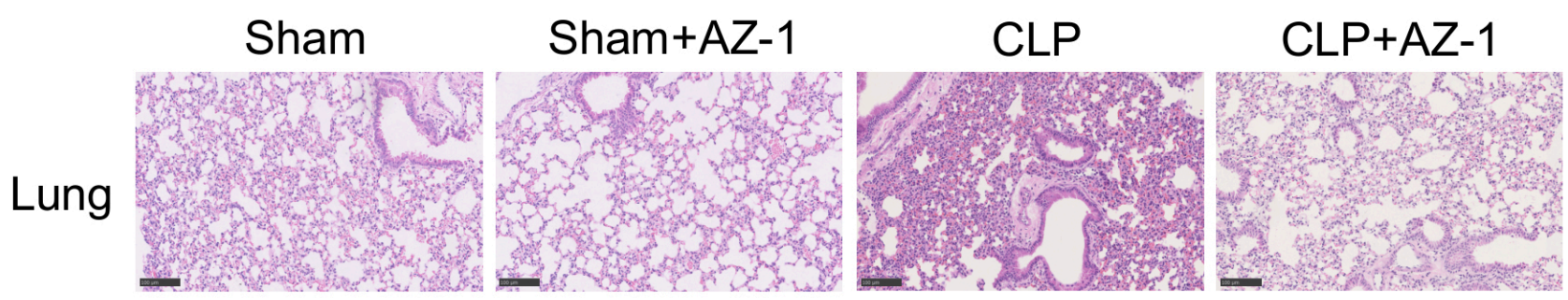

updated Supplementary Fig. 3d

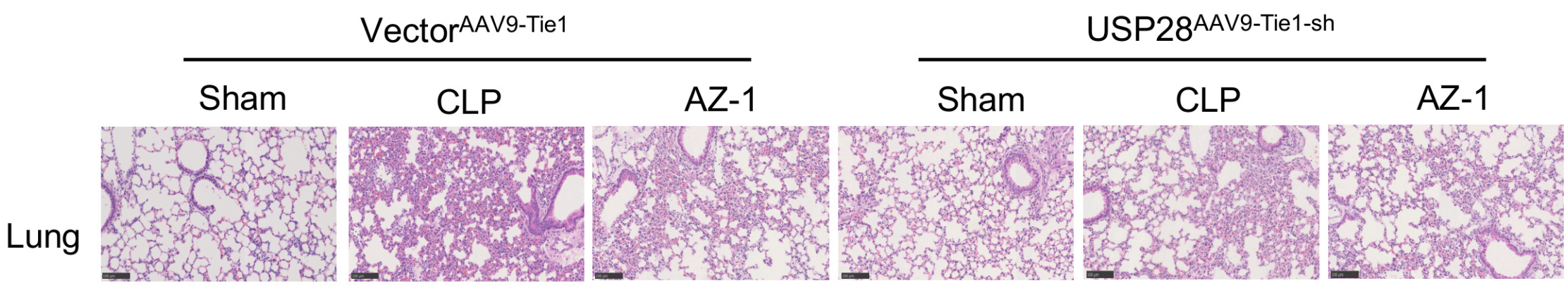

updated Supplementary Fig. 4e

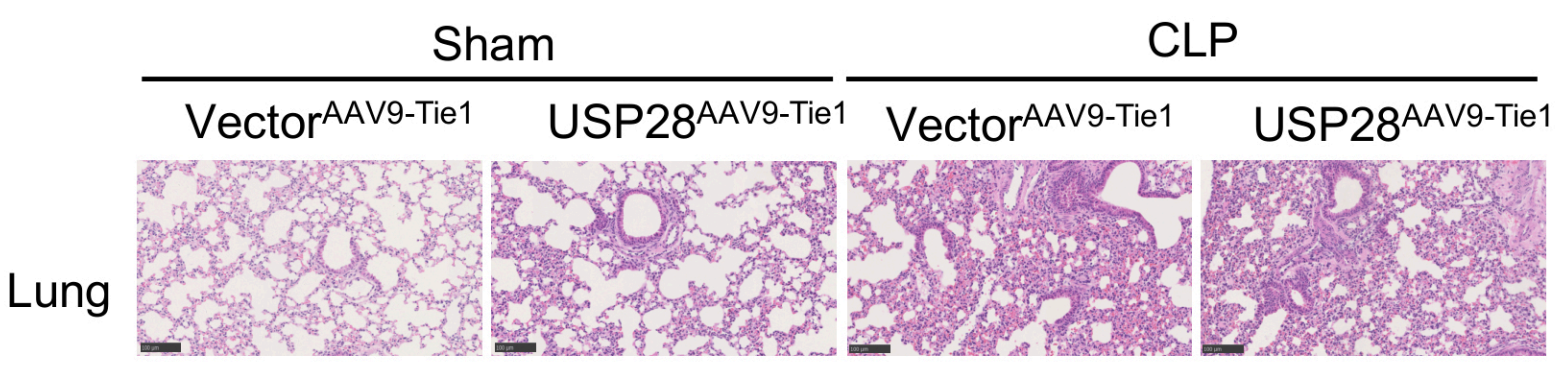

updated Supplementary Fig. 8d

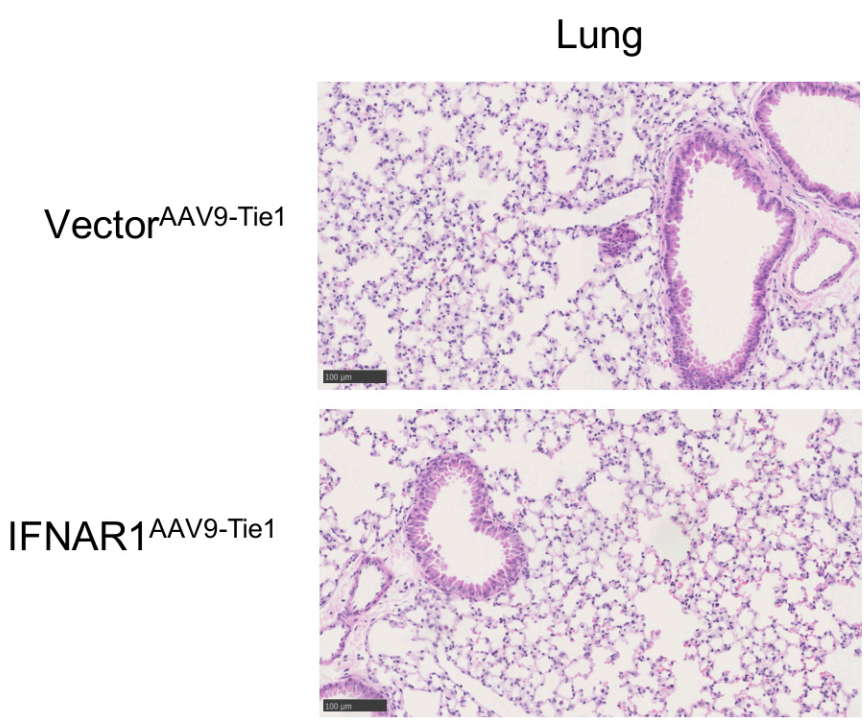

updated Supplementary Fig. 11d

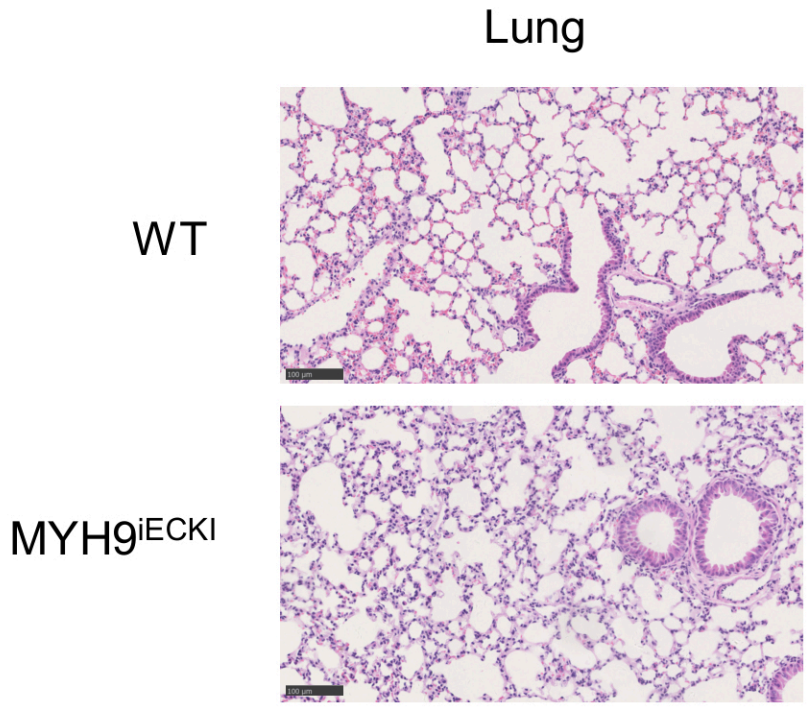

3. While it is common practice to employ CLP models of varying severities when independently assessing survival rates versus organ function, applying different severities of CLP across different experimental groups within the same study/comparison seems quite unusual. Could the authors provide references to other studies or researchers that have utilized a similar experimental design?

Response: We thank the reviewer for raising this important concern. We would like to clarify that different CLP severities were not applied across experimental groups within the same direct comparison. In each individual experiment and statistical comparison, all directly compared groups were subjected to the same CLP protocol. In our study, a milder CLP protocol was used only in the endothelial USP28 overexpression experiments. As Reviewer #2 previously pointed out, under severe CLP conditions, tissue injury, inflammatory responses, and mortality may already be close to their maximal levels, which would create a ceiling effect and reduce the dynamic range needed to detect an additional detrimental effect caused by USP28 overexpression. Thus, the use of a milder CLP protocol in the gain-of-function experiments was intended to preserve the sensitivity of the model for detecting disease aggravation, rather than to compare results directly with those obtained from the severe CLP experiments.

This strategy is consistent with the adjustable nature of the CLP model. Rittirsch et al. from University of Michigan Medical School described CLP as an “immune-design” model in which defined severity grades of sepsis can be generated by modulating the position and extent of cecal ligation, and emphasized that the inflammatory response varies with sepsis severity and is highly dependent on the extent of cecal ligation **(doi: 10.1038/nprot.2008.214)**. In a recent *Nature Communications* study, Oliveira et al. from Universidade do Porto first used a mid-grade CLP protocol to evaluate the susceptibility, bacterial dissemination, inflammatory response, and organ involvement in CD5L-deficient mice, and then used a high-grade CLP protocol to test the protective efficacy of recombinant CD5L treatment under a more severe disease condition **(doi:**

10.1038/s41467-024-48360-8). This provides an example in which CLP severity was selected according to distinct experimental objectives within the same overall study, while direct comparisons were still made within the same severity setting. In addition, Ming et al. used both lethal CLP and nonlethal CLP in a *Journal of Clinical Investigation* study on TREM2 in sepsis **(doi: 10.1172/JCI159400),** and further clarified in their reply that nonlethal CLP was employed for the detection of inflammatory cell infiltration at 72 h **(doi: 10.1172/JCI189219)**. Together, these studies support the general principle that CLP severity can be tailored to the experimental question and endpoint, provided that the severity is kept identical within each direct comparison.

Therefore, in our study, the different CLP severities were not used to create unequal conditions between experimental groups. Rather, they were selected for independent experimental modules with different purposes. Accordingly, we have added supporting references in the revised manuscript **(page 21, lines 462; page 42, lines 921–922)**.

Reviewer #4 (Remarks to the Author):

All concerns have been addressed.

Response: We thank the reviewer for the positive comments.

Reviewer #5 (Remarks to the Author):

All questions of reviewer 2 were adequately answered
Suppl figure 14 is an informative graphical abstract, but I miss what all purple arrows mean. Are all relations positive feedback loops? The legend to suppl figure 14 is to short to be a stand-alone figure. Please expand and explain

Response: We sincerely thank the reviewer for the careful evaluation of our revised manuscript and for acknowledging that the concerns raised by Reviewer #2 have been adequately addressed. We also appreciate the reviewer's helpful suggestion regarding Supplementary Fig. 14. We agree that the previous legend for Supplementary Fig. 14 was too brief to serve as a stand-alone explanation of the graphical model. In particular, the meaning of some arrows was not clearly defined, which could lead to the misunderstanding that each purple arrow represents an independent positive feedback loop. We have therefore revised Supplementary Fig. 14 by adding labels to the arrows in the schematic and have also expanded the figure legend to describe the molecular events shown in the schematic **(updated Supplementary Fig. 14)**.

updated Supplementary Fig. 14

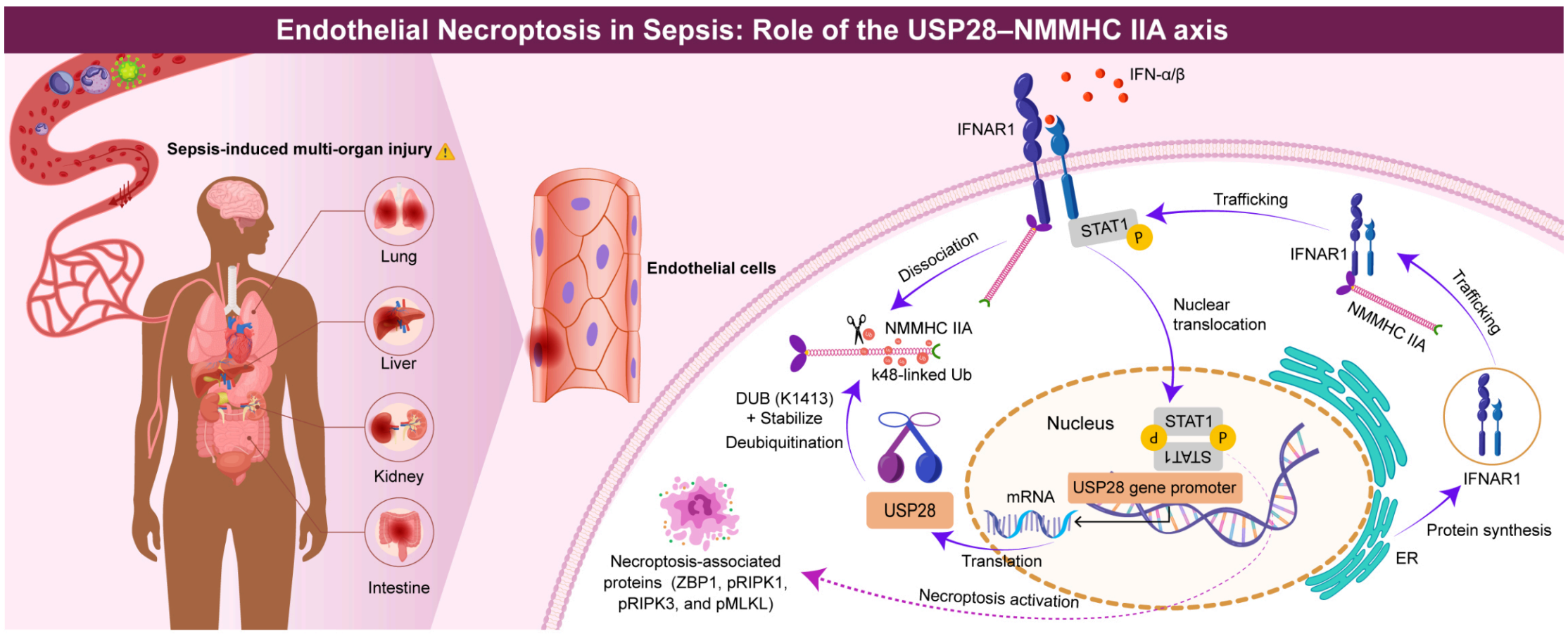

Supplementary Figure 14. Schematic summary of the mechanism by which NMMHC IIA–USP28 axis exacerbates sepsis. In sepsis, excessive and sustained type I interferon activates IFNAR1/STAT1 signaling in endothelial cells. Activated STAT1 translocates into the nucleus and promotes USP28 transcription, leading to increased USP28 expression. Mechanistically, USP28 directly binds to NMMHC IIA and removes K48-linked ubiquitination from NMMHC IIA at the K1413 site through its catalytic ubiquitin-specific protease domain, thereby preventing proteasomal degradation and stabilizing NMMHC IIA. Stabilized NMMHC IIA further interacts with IFNAR1 and facilitates IFNAR1 trafficking and accumulation at the plasma membrane, which enhances IFNAR1/STAT1 signaling. Thus, STAT1-dependent USP28 upregulation and USP28–NMMHC IIA-mediated amplification of IFNAR1/STAT1 signaling form a positive feedback loop. Sustained activation of this loop promotes ZBP1/RIPK1/RIPK3/MLKL-dependent endothelial necroptosis, impairs endothelial barrier integrity, increases vascular leakage, and ultimately aggravates sepsis-induced multi-organ injury.

Responses to the Reviewers' comments

Title: USP28 potentiates endothelial necroptosis via deubiquitinating NMMHC IIA in septic male mice

Manuscript Number: NCOMMS-25-86271

We sincerely thank the reviewers for their careful evaluation of our manuscript and for their valuable comments throughout the review process. No additional reviewer comments were provided in this round. We greatly appreciate the reviewers' time and consideration.
